# Organophotoredox-catalyzed semipinacol rearrangement via radical-polar crossover

**DOI:** 10.1038/s41467-022-30395-4

**Published:** 2022-05-13

**Authors:** Taiga Kodo, Kazunori Nagao, Hirohisa Ohmiya

**Affiliations:** 1 Division of Pharmaceutical Sciences, Graduate School of Medical Sciences, Kanazawa University, Kakuma-machi, Kanazawa, 920-1192 Japan; 2 Institute for Chemical Research, Kyoto University, Gokasho, Uji, Kyoto 611-0011 Japan; 3 JST, PRESTO, 4-1-8 Honcho, Kawaguchi, Saitama 332-0012 Japan

**Keywords:** Synthetic chemistry methodology, Photocatalysis, Organocatalysis

## Abstract

Over the past century, significant progress in semipinacol rearrangement involving 1,2-migration of α-hydroxy carbocations has been made in the areas of catalysis and total synthesis of natural products. To access the α-hydroxy carbocation intermediate, conventional acid-mediated or electrochemical approaches have been employed. However, the photochemical semipinacol rearrangement has been underdeveloped. Herein, we report the organophotoredox-catalyzed semipinacol rearrangement via radical-polar crossover (RPC). A phenothiazine-based organophotoredox catalyst facilitates the generation of an α-hydroxy non-benzylic alkyl radical followed by oxidation to the corresponding carbocation, which can be exploited to undergo the semipinacol rearrangement. As a result, the photochemical approach enables decarboxylative semipinacol rearrangement of β-hydroxycarboxylic acid derivatives and alkylative semipinacol type rearrangement of allyl alcohols with carbon electrophiles, producing α-quaternary or α-tertiary carbonyls bearing *sp*^*3*^-rich scaffolds.

## Abstract (web summary)

Although electrochemical semipinacol rearrangements have been reported, a complementary photochemical route has yet to be developed. Here the authors report an organophotoredox-catalyzed semipinacol rearrangement via radical-polar crossover.

## Introduction

**Fig. 1:**
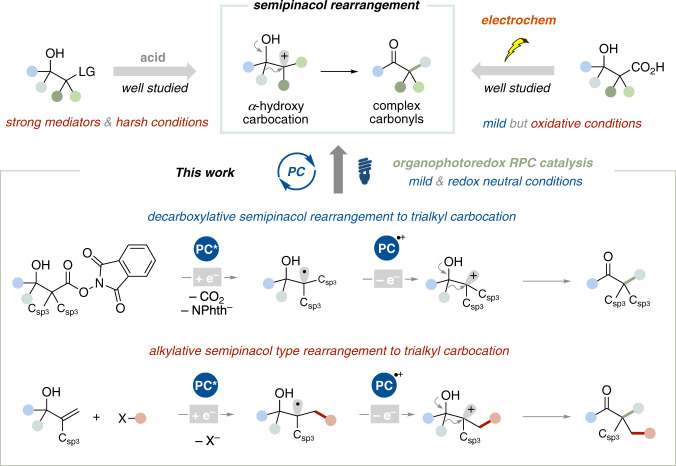
Overview of semipinacol rearrangement. Comparison with previous works such as ionic approach or electrochemical approach and this work based on organophotoredox catalysis.

Rapid and modular synthesis of complex and three-dimensional scaffolds from readily available materials is one of the primary missions of modern organic synthesis^1^. The development of synthetic reactions to meet such demands provides new options for synthetic plans for pharmaceutical drug candidates and natural bioactive molecules. In this context, rearrangements have continuously fascinated synthetic chemists because they allow for the recombination of substituents that are difficult to couple by conventional addition or substitution reactions. Over the past century, semipinacol rearrangement involving 1,2-migration of α-hydroxy carbocations has been broadly utilized as a key step in the total synthesis of natural products^2–5^. Complex carbonyl compounds, such as α-quaternary carbonyls and α-spiroketones can be synthesized through semipinacol rearrangement. However, the reported methods have mainly relied on ionic approaches using strong acids or bases as mediators, which limits the coexistable functional groups and substrate structures (Fig. 1, left top). To overcome these problems, intrinsically different synthetic strategies are required.

Oxidative radical-polar crossover (RPC) has emerged as a non-acidic and mild approach to a carbocation via single electron oxidation of an alkyl radical^6,7^. The reaction design of semipinacol rearrangement via RPC has been pursued in conjunction with the development of electrochemistry which enables the generation and oxidation of the reactive carbon-centered radical species (Fig. 1, right top). Corey and co-workers reported a decarboxylative semipinacol rearrangement of a β-hydroxycarboxylic acid using anodic oxidation^8^. Therein, the first single electron oxidation of the β-hydroxycarboxylate affords the α-hydroxyalkyl radical via decarboxylation. The transient radical is further oxidized to an α-hydroxy carbocation intermediate, resulting in the semipinacol rearrangement. Thus, β-hydroxycarboxylic acids, easily prepared by the reaction between inexpensive and abundant carbonyls and aliphatic carboxylic acids, were added as a new type of semipinacol rearrangement precursor. Although the consecutive single-electron oxidation requires oxidative conditions, the electrochemical semipinacol rearrangement of β-hydroxycarboxylic acids has been extensively studied by many researchers^9,10^. In contrast to the above-mentioned electrochemical methods, a photochemical approach for decarboxylative semipinacol rearrangement of β-hydroxycarboxylic acid derivatives has been scarce^11–13^. Although various photoredox catalysis realizing decarboxylative coupling using aliphatic carboxylic acid derivatives have been presented so far^14–17^, a single electron oxidation of a transient alkyl radical is challenging due to the low concentration of the photooxidant^18–21^. Therefore, a lack of general and versatile RPC-based photochemical approaches has limited the development of decarboxylative semipinacol rearrangements.

Here, we report an organophotoredox-catalyzed decarboxylative semipinacol rearrangement of β-hydroxyesters via RPC (Fig. 1, bottom). The two features of the benzo[*b*]phenothiazine-based organophotoredox catalyst, a strong reductant in the excited state and a persistent and moderate oxidant in the radical cation form, allow it to generate an α-hydroxy non-benzylic alkyl radical. This is followed by oxidation to the corresponding carbocation, which can be exploited in the semipinacol rearrangement to access α-quaternary or α-tertiary carbonyls bearing *sp*^*3*^-rich scaffolds. The photochemical approach also enables alkylative semipinacol type rearrangements of allyl alcohols with carbon electrophiles. The mild, redox neutral and transition metal-free conditions are notable features that cannot be achieved by other methods.

## Results and discussion

### Development of the reaction and screening of conditions

Our group has made ongoing contributions to the development of organophotoredox catalysis enabling redox neutral RPC processes^22–25^. A phenothiazine photoredox catalyst allows the generation of an alkyl radical by single electron reduction of aliphatic carboxylic acid-derived *N*-acyloxyphthalimides under visible light irradiation. The persistent^26–28^ and high oxidation nature of the phenothiazine radical cation converts the transient trialkyl-substituted alkyl radical to an alkylsulfonium intermediate, which reacts with various heteroatom nucleophiles as a carbocation equivalent. To show the potential for C–C bond formation, we initiated a program to develop a RPC-based photochemical semipinacol rearrangement. Various reaction components were screened in the decarboxylative semipinacol rearrangement using **1a** synthesized by the reaction between *p*-anisaldehyde and cyclohexane carboxylic acid. It was found that *N*-phenyl benzo[*b*]phenothiazine (**PTH1**)^29^ as an organophotoredox catalyst promoted the desired rearrangement to afford the α-quaternary aldehyde **2a** in high yield with formation of the H-migrated product **3a** as a byproduct (Table 1, entry 1).

**Table 1:** Screening of reaction conditions.


Entry	Change from standard conditions^a^	Yield (%) of 2a	Yield (%) of 3a
1	None	95 (91)	5
2	**PTH2** instead of **PTH1**	84	7
3	**PTH3** instead of **PTH1**	87	4
4	**PTH4** instead of **PTH1**	88	4
5	**PTH5** instead of **PTH1**	15	0
6	**PTH6** instead of **PTH1**	43	2
7	**PTH7** instead of **PTH1**	66	7
8	**POX1** instead of **PTH1**	84	7
9	**PTH1** (5 mol %) and **LiBF**_**4**_ (5 mol %)	86	5
10	**PTH1** (2 mol %) and **LiBF**_**4**_ (2 mol %)	65	5
11	**4Cz-IPN** (2 mol %) instead of **PTH1**	67	5
12	**Ir(ppy)**_**3**_ (2 mol %) instead of **PTH1**	29	4
13	**Ir[dF(CF**_**3**_**)ppy]**_**2**_**(dtbbpy)PF**_**6**_ (2 mol %) instead of **PTH1** and LiBF_4_	48	8
14	**Ru(bpy)**_**3**_**(PF**_**6**_**)**_**2**_ (2 mol %) instead of **PTH1** and LiBF_4_	0	0
15	Without LiBF_4_	4	0


^*a*^Reaction was carried out with **1a** (0.2 mmol), photocatalyst (0.02 mmol), LiBF_4_ (0.02 mmol), EtOAc (0.6 mL), 24 h under blue LED irradiation. NMR yield (Isolated yield in parentheses).

The screening of organophotoredox catalysts revealed that their structure significantly affected the reaction efficiency. Regardless of the *N*-substituent, benzo[*b*]phenothiazine derivatives consistently provided the desired aldehydes in high yields (entries 2–4). In contrast, a benzo[*a*]phenothiazine scaffold did not work well (entry 5). Simple phenothiazine scaffolds showed moderate reactivities (entries 6 and 7). These results were consistent with our previous studies exploiting the intermediacy of a charge transfer complex of a benzo[*b*]phenothiazine catalyst and a redox active ester fragment. A phenoxazine catalyst developed by the Miyake group^30^ also showed high catalytic activity (entry 8). The reaction also underwent well with 5 or 2 mol% of photoredox catalyst (entries 9 and 10). The semipinacol rearrangement with a representative organic photoredox catalyst, 4Cz-IPN^31,32^, proceeded well (entry 11). The Doyle group disclosed that the homoleptic iridium photoredox catalyst, Ir(ppy)_3_, acted as an excellent RPC catalyst, however, this reaction did not take place efficiently (entry 12). The reaction with other Ir-based photoredox catalyst, Ir[dF(CF_3_)ppy]_2_(dtbbpy)PF_6_ resulted in moderate product yield (entry 13). In contrast, Ru(bpy)_3_(PF_6_)_2_ was not effective in this reaction (entry 14). Without LiBF_4_, the reaction efficiency was significantly decreased (entry 15). LiBF_4_ might stabilize the phenothiazine radical cation and radical anion form of **1a**, respectively and inhibit the back electron transfer^33^.

### Substrate scope of decarboxylative semipinacol rearrangement

**Fig. 2:**
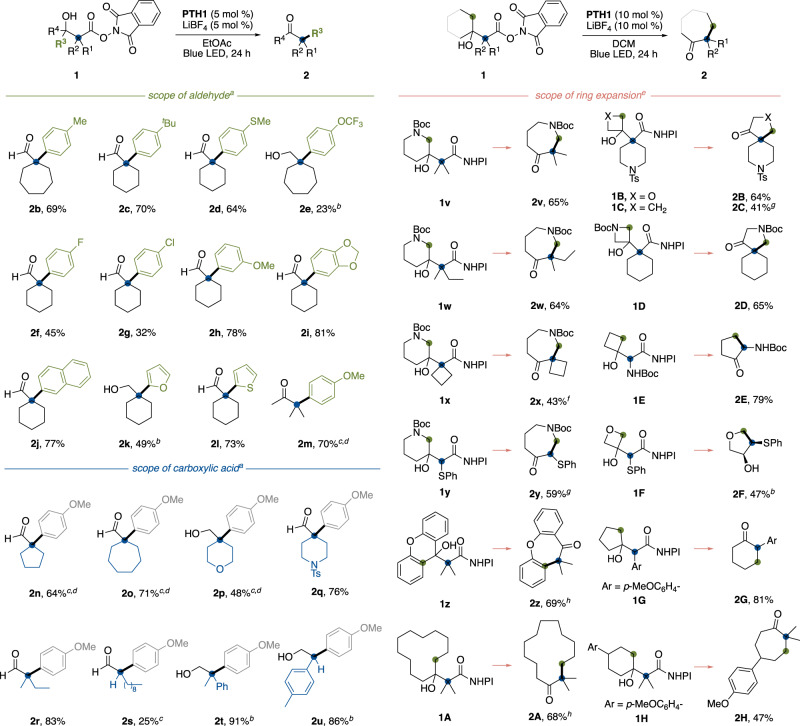
Substrate scope of decarboxylative semipinacol rearrangement. **a** Reaction was carried out with **1** (0.2 mmol), **PTH1** (0.01 mmol), LiBF_4_ (0.01 mmol), EtOAc (0.6 mL), 24 h under blue LED irradiation. **b** The product was isolated as the alcohol through reduction by NaBH_4_ (0.4 mmol) in MeOH (5.0 mL). **c** **PTH1** (0.02 mmol) and LIBF4 (0.02 mmol) were used. **d** MeCN (0.6 mL) was used instead of EtOAc. **e** Reaction was carried out with **1** (0.2 mmol), **PTH1** (0.02 mmol), LiBF_4_ (0.02 mmol), DCM (0.6 mL), 24 h under blue LED irradiation. **f** 12 h instead of 24 h. **g** EtOAc (0.6 mL) was used instead of DCM. **h** MeCN (0.6 mL) was used instead of DCM.

We explored the scope of β-hydroxyesters prepared by the reaction between aromatic aldehydes and aliphatic carboxylic acids (Fig. 2). First, various migrating aryl groups originating from aldehydes were investigated. As with the *p*-anisyl group, electron-donating groups, alkyl and thioether substituents at *para* positions, promoted the desired reaction efficiently (**2b**–**2d**). On the other hand, an electron-withdrawing group decreased the reactivity (**2e**). Halogen substituents were tolerated (**2** **f** and **2** **g**). An ether functional group at the *meta* position did not affect the reaction (**2** **h** and **2i**). Naphthalene or electron-rich heteroaromatic rings participated in this reaction (**2j**–**2** **l**). Notably, when an acetophenone-derived substrate **1** **m** was used, the aromatic ring selectively migrated to the adjacent carbon center over the methyl group (**2** **m**). This phenomenon is consistent with the conventional acid-mediated semipinacol rearrangement.

Subsequently, aliphatic scaffolds stemming from aliphatic carboxylic acids were studied (Fig. 2). Various cycloalkane moieties could be installed at the α-position of aldehyde products (**2n** and **2o**). The 4-position of tetrahydropyran or piperizine scaffolds could be utilized as a migration site (**2p** and **2q**). This protocol could generate tertiary and secondary acyclic carbocation species, which induced the semipinacol rearrangement. For example, benzylic or non-benzylic aliphatic carboxylic acids produced α-quaternary or α-tertiary aldehydes (**2r**–**2** **u**).

Next, we examined the ring expansion-type semipinacol rearrangement with β-hydroxyesters, which were prepared by the reaction between cyclic ketones and aliphatic carboxylic acids (Fig. 2). The reactions with esters **1v**–**y** derived from inexpensive 3-piperidone afforded the 3-functionalized 4-azepanone derivatives **2v**–**y**, which are potential synthetic precursors of opioid receptor agonists, such as meptazinol. For these substrates, the α-aminoalkyl group preferentially migrated to the adjacent carbocation center over the primary alkyl one. The formal dimethylative ring expansion of xanthone and cyclododecanone proceeded well to produce the α-quaternary ketones in high yields (**2z** and **2** **A**). Our protocol also could construct the heteroatom-containing spiroketone scaffolds (**2B**–**D**). Carbocations stabilized by heteroatoms were able to trigger the desired rearrangements (**2E** and **2** **F**). Simple cyclopentanol and cyclohexanol scaffolds were efficiently converted to the corresponding cyclohexanone and cycloheptanone derivatives with α-functionalization by our reaction (**2** **G** and **2H**). Notably, most of the products obtained by our protocol could not be accessed by conventional enolate alkylation of ketones with complete regioselectivity.

### Alkylative semipinacol type rearrangement

Next, we attempted to develop an alkylative semipinacol type rearrangement utilizing this organophotoredox catalysis. Recently, several groups have reported photoredox protocols enabling the alkylative semipinacol type rearrangement of allyl alcohols with carbon or heteroatom electrophiles^5,34–38^. In common with reported methods, the catalytic process consists of the addition of a reductively-generated alkyl or heteroatom radical to the alkene moiety of allyl alcohols, followed by the oxidation of the prolonged carbon-centered radical to the carbocation, which induces the semipinacol rearrangement. Although a broad range of electrophiles have been utilized, the following limitations existed: (1) the available RPC sites are limited to the benzylic position, (2) usable photoredox catalysts are limited to metal-based ones. We envisioned that our organophotoredox protocol could provide solutions to these problems to construct *sp*^*3*^-rich scaffolds in an environmentally benign manner.

**Fig. 3:**
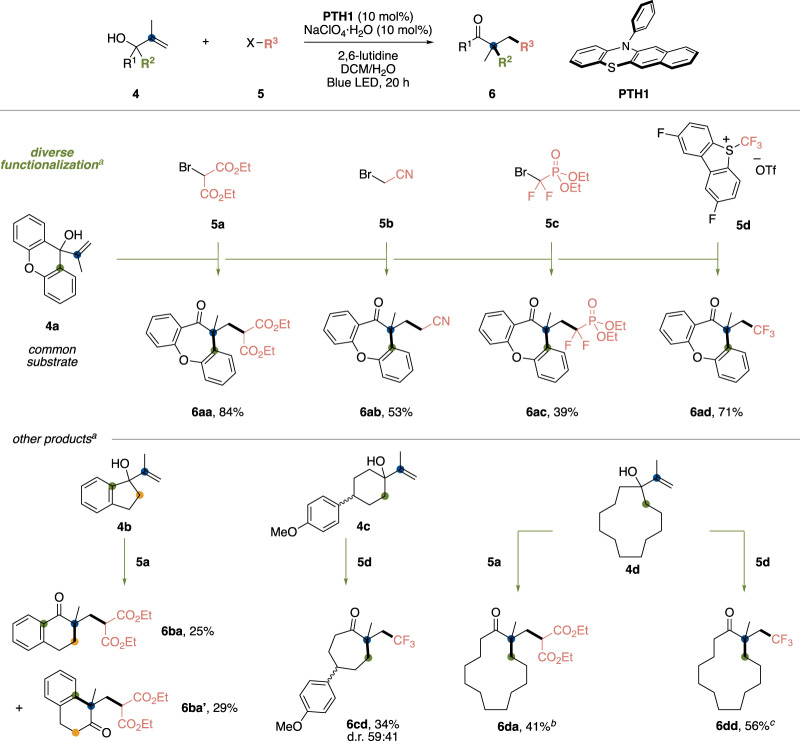
Substrate scope of alkylative semipinacol type rearrangement. **a** Reaction was carried out with **4** (0.2 mmol), **5** (0.3 mmol), **PTH1** (0.02 mmol), NaClO_4_∙H_2_O (0.02 mmol) and lutidine (0.4 mmol) in DCM (562.5 μL) and water (37.5 μL) for 20 h under blue LED irradiation. **b** Hexafluoroisopropanol (37.5 μL) was used instead of water. **c** TBABF_4_ (0.02 mmol) and MeCN (562.5 μL) were used instead of NaClO_4_∙H_2_O and DCM, respectively.

After a slight modification of the reaction conditions, we achieved the desired alkylative semipinacol type rearrangements between allylic alcohols and various carbon electrophiles (Fig. 3)^39,40^. For example, allylic alcohol **4a** prepared by the reaction between xanthone and isopropenyl Grignard reagent could react with diethyl bromomalonate **5a** with a catalytic amount of **PTH1** and sodium perchlorate salt and a stoichiometric amount of lutidine to afford the alkylative ring expansion product **6aa** in high yield. Notably, we found that other carbon electrophiles, such as bromoacetonitrile **5b**, diethyl bromodifluoromethanephosphonate **5c** and Umemoto’s reagent II **5d** were competent coupling partners, allowing access to diverse product scaffolds from a single substrate. Allylic alcohol substrates **4b**–**4d** derived from cyclic ketones also engaged with this catalysis to undergo alkylative ring expansion reactions (**6ba**, **6cd**, **6da** and **6dd**).

### Possible pathways

**Fig. 4:**
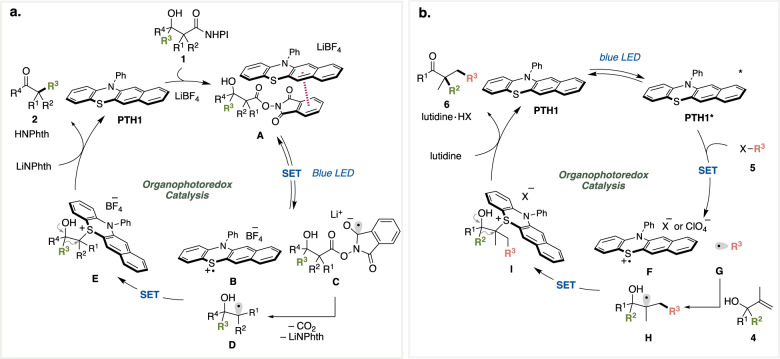
Possible pathways. **a** A proposed catalytic cycle for decarboxylative semipinacol rearrangement. **b** A proposed catalytic cycle for alkylative semipinacol type rearrangement.

Based on our previous reports^22–25^ on the phenothiazine-based organophotoredox catalysis, plausible pathways are outlined in Fig. 4. For decarboxylative semipinacol rearrangement (Fig. 4a), a charge transfer complex **A** consisting of the **PTH1** catalyst and a redox active ester moiety of **1** undergoes a single electron transfer to generate the radical cation form of **PTH1** (**B**) and the radical anion form of redox active ester (**C**). **C** collapses to afford an α-hydroxyalkyl radical (**D**) with carbon dioxide and a phthalimide anion. Next, a recombination event between **B** and **D** occurs to provide an alkylsulfonium intermediate (**E**) as the α-hydroxy carbocation equivalent. In the presence of a catalytic amount of lithium phthalimide anion, the semipinacol rearrangement produces the desired carbonyl product (**2**) with regeneration of the phenothiazine catalyst.

On the other hand, the result of UV-Vis absorption experiments indicated that a charge transfer complex between **PTH1** and an alkyl electrophile **5** was not involved in the alkylative semipinacol type rearrangement (Fig. 4b). Instead, blue LED irradiation converts the phenothiazine catalyst **PTH1** to the corresponding excited state PTH1* bearing high reducing ability. A single electron reduction of **5** by PTH1* produces the cation radical form of **PTH1** (**F**) and an alkyl radical **G**, which undergoes addition across the carbon–carbon double bond of allyl alcohol (**4**) to generate the α-hydroxyalkyl radical (**H**). The recombination of **H** and **F** and semipinacol rearrangement of the alkylsulfonium intermediate (**I**) occur sequentially to afford the desired product **6**. Our preliminary DFT calculation revealed that the alkylsulfonium is more stable than the corresponding discrete carbocation by 8.0 kcal/mol. The result suggested the intermediacy of alkylsulfonium although the reaction pathway involving the formation of discrete carbocation cannot be ruled out (Supplementary Figs. 7 and 8).

In summary, we can now add an organophotoredox RPC protocol to the synthetic toolbox for semipinacol rearrangement. This protocol accesses the α-hydroxy carbocation equivalent from β-hydroxyesters using inexpensive and naturally abundant materials under mild, redox neutral and transition metal-free conditions. The persistent and high oxidation nature of the phenothiazine cation radical enables RPC on a transient trialkyl-substituted carbon center to broaden the accessible *sp*^*3*^-rich scaffolds. The radical relay process including radical addition to alkenes can be incorporated in organophotoredox catalysis. Studies for expanding the substrate scope are currently underway in our laboratory.

## Methods

### The reaction to produce 2a in Table 1, entry 1 is representative

In a glovebox, to an oven-dried vial with a stirring bar was added **PTH1** (3.3 mg, 0.01 mmol), lithium tetrafluoroborate (0.9 mg, 0.01 mmol) and β-hydroxyester **1a** (81.9 mg, 0.2 mmol). Ethyl acetate (600 µL) was added to the reaction mixture. The reaction was stirred and irradiated with a 34 W blue LED (0.5 cm away) with a cooling fan to keep the temperature around 40 °C. After 24 h, the reaction was quenched by 0.5 M NaOH aq. solution (1.5 mL). The aqueous layer was extracted three times with dichloromethane (500 μL × 3) and then the combined organic layer was dried over Na_2_SO_4_ and filtered. After volatiles were removed under reduced pressure, purification by flash column chromatography on silica gel (100:0–90:10, hexane/EtOAc) gave the rearrangement product **2a** (39.7 mg, 0.18 mmol, 91% yield).

### The reaction to produce 6aa in Fig. 3 is representative

In a glovebox, to an oven-dried vial with a stirring bar was added **PTH1** (6.5 mg, 0.02 mmol), sodium perchlorate monohydrate (2.8 mg, 0.02 mmol) and allylic alcohol **4a** (47.7 mg, 0.2 mmol). Dichloromethane (562.5 µL), water (37.5 µL) and ethyl bromomalonate (50.5 µL, 0.3 mmol) were added to the reaction mixture. The reaction was stirred and irradiated with a 34 W blue LED (0.5 cm away) with a cooling fan to keep the temperature around 40 °C. After 20 h, the reaction was quenched by sat. NH_4_Cl aq. solution (1.5 mL). The aqueous layer was extracted three times with dichloromethane (500 μL × 3), and then the combined organic layer was dried over Na_2_SO_4_ and filtered. After volatiles were removed under reduced pressure, purification by flash column chromatography on silica gel (Biotage Selekt, 99:1–90:10, hexane/EtOAc) gave the rearrangement product **6aa** (66.6 mg, 0.17 mmol, 84% yield).

## Supplementary information

Supplementary Information

## Data Availability

The authors declare that the data supporting the findings of this study are available within the paper or its Supplementary Information files and from the corresponding author upon request.
