# Supplementary material for: Organophotoredox-catalyzed semipinacol rearrangement via radical-polar crossover
Source: Nat Commun. 2022 May 13;13:2684. doi: 10.1038/s41467-022-30395-4 (PMC9106707; doi:10.1038/s41467-022-30395-4)
Supplement: Supplementary file 1 — Supplementary Information [file 41467_2022_30395_MOESM1_ESM.pdf]

Supplementary Information for

Organophotoredox-Catalyzed Semipinacol Rearrangement via Radical-Polar Crossover

Taiga Kodo,[†] Kazunori Nagao,*[†] and Hirohisa Ohmiya*[‡§]

[†]Division of Pharmaceutical Sciences, Graduate School of Medical Sciences, Kanazawa University, Kakuma-machi, Kanazawa 920-1192, Japan

[‡] Institute for Chemical Research, Kyoto University, Gokasho, Uji, Kyoto 611-0011, Japan

[§]JST, PRESTO, 4-1-8 Honcho, Kawaguchi, Saitama, 332-0012, Japan

*E-mail: Kazunori Nagao : nkazunori@p.kanazawa-u.ac.jp
Hirohisa Ohmiya: ohmiya@scl.kyoto-u.ac.jp

Table of Contents

■ **Supplementary Methods** ■

1. Instrumentation and Chemicals S2
2. General Procedure for Synthesis of β–Hydroxy Esters S3
3. Characterization Data for β–Hydroxy Esters S4–S17
4. General Procedures for Organophotoredox–Catalyzed Semipinacol Rearrangement S18
5. Characterization Data for Rearrangement Products S19–S29
6. Characterization Data for Allylic Alcohols S30
7. General Procedure for Alkylative Semipinacol Type Rearrangement S31
8. Characterization Data for Alkylative Semipinacol Type Rearrangement Products S31–S34
9. Effects of Reaction Components on Alkylative Semipinacol Type Rearrangement S35
10. UV-Vis Absorption Spectra of Reaction Mixtures S36
11. Computational Study S37–S40
12. Reaction Mechanism via A Discrete Carbocation Intermedaite S40

■ **Supplementary References** ■ S41–S42

■ **NMR Spectra** ■ S43–S208

■ **Supplementary Methods** ■

1. Instrumentation and Chemicals

NMR spectra were recorded on a JNM-ECS400, operating at 400 MHz for ^{1}H NMR and 100.5 MHz for ^{13}C NMR, JNM-ECA600, operating at 600 MHz for ^{1}H NMR and 150.9 MHz for ^{13}C NMR, and Bruker Avance NEO 400N, operating at 400 MHz for ^{1}H NMR and 100.6 MHz for ^{13}C NMR, 376.5 MHz for ^{19}F NMR and 162.0 MHz for ^{31}P NMR. Chemical shifts were reported in δ ppm. Chloroform-d_1 ($CDCl_3$) containing 0.03% tetramethylsilane (TMS) (>99.8%D, Cambridge Isotope Laboratories, Inc., Cat. No. DLM-7), and dimethyl sulfoxide-d_6 (99.9%D, Cambridge Isotope Laboratories, Inc., Cat. No. DLM-10) were used as solvents for NMR measurements at ambient temperature. Chemical shifts (δ) for ^{1}H NMR are given in parts per million (ppm) relative to TMS (δ 0.00 ppm in $CDCl_3$), or residual dimethyl sulfoxide (δ 2.50 ppm). Chemical shifts (δ) for ^{13}C NMR are given in ppm relative to $CDCl_3$ (δ 77.0 ppm) or residual dimethyl sulfoxide (δ 39.5 ppm). Chemical shifts (δ) for ^{19}F NMR are given in ppm relative to α,α,α−trifluorotoluene (δ −63.0 ppm in $CDCl_3$) used as the external standard. Chemical shifts (δ) for ^{31}P NMR are given in ppm relative to $BF_3{\cdot}OEt_2$ (δ 0.0 ppm in $CDCl_3$) used as the external standard. The abbreviations s, d, t, q, h, br s, and m signify singlet, doublet, triplet, quartet, heptet, broad singlet, and multiplet, respectively. Mass spectra were obtained with JMS-T100TD (DART and ESI). TLC analyses were performed on commercial glass plates bearing 0.25-mm layer of Merck Silica gel 60F_{254}. Silica gel (Wakosil® 60, 64~210 μm) were used for column chromatography. Gel Permeation Chromatography (GPC) was performed with LaboACE LC−5060 using UV Detector and Refractive Index Detector. IR spectra were measured with a Thermo Scientific iD7 ATR Accessory for the Thermo Scientific Nicolet iS5 FT-IR Spectrometer. Melting points were measured on a Stanford Research System MPA100 (OptiMelt) apparatus. CV measurements were recorded with a Hokuto Denko HZ-7000 potentiostat. UV-Vis absorption spectra were recorded on a Shimadzu UV-1900. Fluorescence spectra were recorded on a Shimadzu RF-6000. Kessil A160W Tuna Blue (highest blue and intensity setting) was used as a light source. TEKNOS MG9 was used as a fan.

All reactions were carried out under nitrogen atmosphere. Materials were obtained from commercial suppliers or prepared according to standard procedures unless otherwise noted. $LiBF_4$ was purchased from Aldrich Chemical Co., stored under nitrogen, and used as received. Dichloromethane, acetonitrile, ethyl acetate and acetone were purchased from FUJIFILM Wako Pure Chemical Co., stored under nitrogen, and used as received. Allylic alcohols were prepared by the reported procedure.[1] **PTH1, PTH2, PTH3, PTH4, PTH5, PTH6, PTH7** and **PTH8** were prepared by the reported procedure.[2]

2. General Procedure for Synthesis of β–Hydroxy Esters (*Note: These conditions are not fully optimized.)*

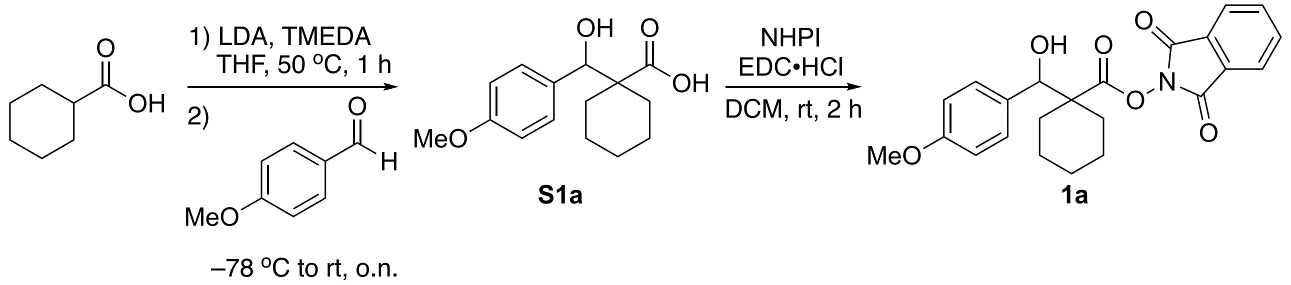

Supplementary Figure 1. Synthesis of substrate

Synthesis of 1a as a representative (Supplementary Figure 1). β–Hydroxy acids were prepared according to the modified conditions of the literature[3]. To a solution of diisopropylamine (2.36 mL, 16.8 mmol) and *N,N,N′,N′*–tetramethylethylenediamine (2.54 mL, 16.8 mmol) in THF (72 mL), cooled to –78 °C, *n*–butyllithium (10.6 mL of a 1.58 M solution in hexanes, 16.8 mmol) was added dropwise. After this mixture had been stirred at –78 °C for 30 min, the solution of cyclohexanecarboxylic acid (1.03 g, 8.0 mmol) in THF (8 mL) was added in one portion. The mixture was heated at 50 °C for 1 h and then cooled to –78 °C. Subsequently, *p*–anisaldehyde (1.22 mL, 10 mmol) was added dropwise. The reaction mixture was stirred at room temperature overnight. After quenching with water (80 mL), the aqueous phase was washed with diethyl ether (3 × 50 mL), then acidified with 2 M HCl to pH = 3. The aqueous phase was subsequently extracted with ethyl acetate (3 × 50 mL). The combined organic layers were dried over sodium sulfate, and filtered, after which the filtrate was concentrated in vacuo. The crude product **S1a** was used for the next condensation reaction without further purification.

The β–hydroxy esters were prepared according to the modified conditions of the literature.[4] To a solution of β–hydroxy acids **S1a** (10 mmol) and *N*–hydroxyphtalimide (NHPI, 1.70 g, 10.4 mmol) in THF (40 mL), cooled to 0 °C, 1-(3-dimethylaminopropyl)-3-ethylcarbodiimide hydrochloride (EDC・HCl, 1.99 g, 10.4 mmol) was added portionwise. The reaction mixture was stirred 2 h at room temperature. After quenching with 2 M HCl (20 mL), the aqueous phase was extracted with dichloromethane (3 × 40 mL). The combined organic layers were dried over sodium sulfate and filtered. After volatiles were removed under reduced pressure, purification by flash column chromatography on silica gel (Biotage Selekt, 95:5–70:30, hexane/EtOAc) gave the desired β–hydroxy ester **1a** as a white solid (1.88 g, 4.58 mmol, 57%).

3. Characterization Data for *β*–Hydroxy Esters

1,3-Dioxoisoindolin-2-yl 1-[Hydroxy(4-methoxyphenyl)methyl]cyclohexane-1-carboxylate (1a)

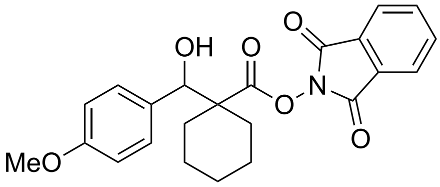

The product **1a** was prepared from *p*–anisaldehyde (1.22 mL, 10 mmol) and cyclohexanecarboxylic acid (1.03 g, 8.0 mmol). The crude residue was purified by flash chromatography on silica gel (Biotage Selekt, 95:5–70:30, hexane/EtOAc) (1.88 g, 4.58 mmol, 57% isolated yield). White solid. **M.p.** 144–146 °C. **IR** (neat) 1048, 1175, 1248, 1373, 1467, 1513, 1737, 1781, 2937, 3515 cm^{-1}. **^{1}H NMR** (400 MHz, $CDCl_3$) δ 7.90–7.89 (m, 2H), 7.81–7.80 (m, 2H), 7.33 (d, J = 7.6 Hz, 2H), 6.89 (d, J = 7.6 Hz, 2H), 4.95 (m, 1H), 3.82 (s, 3H), 3.04 (brs, 1H), 2.38 (d, J = 13.2 Hz, 1H), 2.25 (d, J = 13.2 Hz, 1H), 1.75–1.46 (m, 5H), 1.27–1.13 (m, 2H), 1.03 (m, 1H). **^{13}C NMR** (100.6 MHz, $CDCl_3$) δ 171.5, 162.6, 159.4, 134.8, 131.0, 129.0, 129.0, 124.0, 113.2, 79.4, 55.2, 54.4, 32.1, 27.9, 25.6, 23.4, 22.6. **HRMS–DART** (*m/z*): $[M+NH_4]^+$ calcd for $C_{23}H_{27}N_2O_6^+$, 427.1864; found, 427.1864.

1,3-Dioxoisoindolin-2-yl 1-[Hydroxy(*p*-tolyl)methyl]cycloheptane-1-carboxylate (1b)

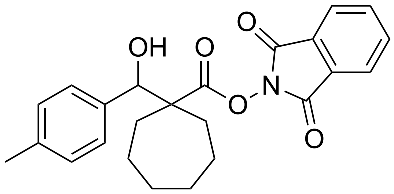

The product **1b** was prepared from *p*–tolualdehyde (736.2 μL, 6.0 mmol) and cycloheptanecarboxylic acid (711.0 mg, 5.0 mmol). The crude residue was purified by flash chromatography on silica gel (Biotage Selekt, 95:5–80:20, hexane/EtOAc) (758.1 mg, 1.86 mmol, 37% isolated yield). White solid. **M.p.** 161–164 °C. **IR** (neat) 518, 696, 877, 1003, 1372, 1735, 1785, 2860, 2931, 3548 cm^{-1}. **^{1}H NMR** (400 MHz, $CDCl_3$) δ 7.89 (brs, 2H), 7.80 (brs, 2H), 7.32 (d, J = 7.6 Hz, 2H), 7.16 (d, J = 7.6 Hz, 2H), 5.08 (s, 1H), 3.27 (s, 1H), 2.35–2.30 (m, 5H), 1.72–1.49 (m, 7H), 1.36–1.34 (m, 3H). **^{13}C NMR** (100.6 MHz, $CDCl_3$) δ 172.7, 162.3, 137.7, 136.1, 134.8, 129.0, 128.5, 127.7, 124.0, 79.5, 56.8, 33.9, 29.9, 29.0, 28.7, 23.8, 23.7, 21.1. **HRMS–DART** (*m/z*): $[M+NH_4]^+$ calcd for $C_{24}H_{29}N_2O_5^+$, 425.2071; found, 425.2071.

1,3-Dioxoisoindolin-2-yl 1-{[4-(*tert*-Butyl)phenyl](hydroxy)methyl}cyclohexane-1-carboxylate (1c)

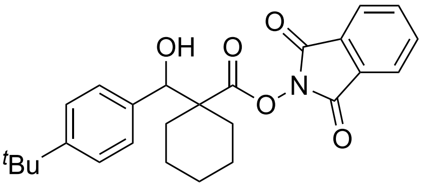

The product **1c** was prepared from 4-*tert*-butylbenzaldehyde (1.00 mL, 6.0 mmol) and cyclohexanecarboxylic acid (640.9 mg, 5.0 mmol). The crude residue was purified by flash chromatography on silica gel (Biotage Selekt, 90:10–70:30, hexane/EtOAc) (874.7 mg, 2.01 mmol,

40% isolated yield). White solid. **M.p.** 179–182 °C. **IR** (neat) 696, 878, 971, 1047, 1373, 1736, 1782, 2866, 2941, 3514 cm^{-1}. **^{1}H NMR** (400 MHz, $CDCl_3$) δ 7.91–7.90 (m, 2H), 7.80–7.79 (m, 2H), 7.38–7.32 (m, 4H), 4.97 (s, 1H), 3.07 (brs, 1H), 2.40 (d, J = 13.2 Hz, 1H), 2.27 (d, J = 13.2 Hz, 1H), 1.76–1.48 (m, 5H), 1.33–1.16 (m, 11H), 1.09–1.00 (m, 1H). **^{13}C NMR** (100.6 MHz, $CDCl_3$) δ 171.5, 162.5, 150.9, 135.9, 134.8, 129.0, 127.6, 124.7, 124.0, 79.7, 54.3, 34.5, 32.1, 31.3, 27.9, 25.5, 23.4, 22.6. **HRMS–DART** (*m/z*): $[M+NH_4]^+$ calcd for $C_{26}H_{33}N_2O_5^+$, 453.2384; found, 453.2384.

1,3-Dioxoisoindolin-2-yl 1-{Hydroxy[4-(methylthio)phenyl]methyl}cyclohexane-1-carboxylate (1d)

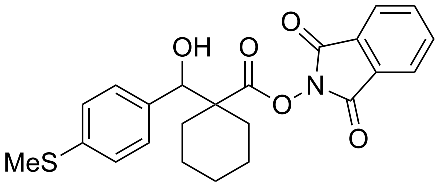

The product **1d** was prepared from 4-(methylthio)benzaldehyde (780.6 µL, 6.0 mmol) and cyclohexanecarboxylic acid (640.9 mg, 5.0 mmol). The crude residue was purified by flash chromatography on silica gel (Biotage Selekt, 95:5–70:30, hexane/EtOAc) (613.9 mg, 1.44 mmol, 29% isolated yield). Yellow solid. **M.p.** 146–150 °C. **IR** (neat) 728, 904, 1046, 1138, 1373, 1734, 1781, 2862, 2936, 3509 cm^{-1}. **^{1}H NMR** (400 MHz, $CDCl_3$) δ 7.88 (brs, 2H), 7.80 (brs, 2H), 7.33 (d, J = 7.6 Hz, 2H), 7.23 (d, J = 7.6 Hz, 2H), 4.96 (s, 1H), 3.18 (m, 1H), 2.49 (s, 3H), 2.37 (d, J = 13.2 Hz, 1H), 2.23 (d, J = 13.2 Hz, 1H), 1.75–1.46 (m, 5H), 1.27–1.12 (m, 2H), 1.02 (m, 1H). **^{13}C NMR** (100.6 MHz, $CDCl_3$) δ 171.3, 162.6, 138.3, 135.7, 134.8, 128.9, 128.3, 125.7, 124.0, 79.3, 54.3, 32.0, 27.9, 25.5, 23.3, 22.5, 15.6. **HRMS–DART** (*m/z*): $[M+ NH_4]^+$ calcd for $C_{23}H_{27}N_2O_5S^+$, 443.1635; found, 443.1631.

1,3-Dioxoisoindolin-2-yl 1-{Hydroxy[4-(trifluoromethoxy)phenyl]methyl}cycloheptane-1-carboxylate (1e)

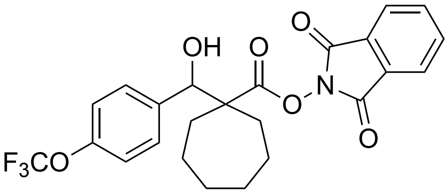

The product **1e** was prepared from 4-(trifluoromethoxy)benzaldehyde (857.7 µL, 6.0 mmol) and cycloheptanecarboxylic acid (711.0 mg, 5.0 mmol). The crude residue was purified by flash chromatography on silica gel (Biotage Selekt, 100:0–80:20, hexane/EtOAc) (1.02 g, 2.131 mmol, 43% isolated yield). White solid. **M.p.** 100–103 °C. **IR** (neat) 878, 1016, 1161, 1221, 1257, 1737, 1784, 2861, 2928, 3516 cm^{-1}. **^{1}H NMR** (400 MHz, $CDCl_3$) δ 7.90–7.89 (m, 2H), 7.82–7.80 (m, 2H), 7.48 (d, J = 7.8 Hz, 2H), 7.21 (d, J = 7.8 Hz, 2H), 5.13 (d, J = 3.2 Hz, 1H), 3.47 (d, J = 3.2 Hz, 1H), 2.35–2.30 (m, 2H), 1.71–1.50 (m, 7H), 1.39–1.35 (m, 3H). **^{13}C NMR** (100.6 MHz, $CDCl_3$) δ 172.3, 162.3, 148.9 (m), 137.8, 134.9, 129.3, 128.9, 124.1, 120.4 (q, $J_{C–F}$ = 257.3 Hz), 120.2, 78.9, 56.9, 33.7, 30.0, 28.9, 28.6, 23.8, 23.7. **^{19}F NMR** (376 MHz, $CDCl_3$) δ -57.8. **HRMS–DART** (*m/z*): $[M+NH_4]^+$ calcd for $C_{24}H_{26}F_3N_2O_6^+$, 495.1737; found, 495.1740.

1,3-Dioxoisoindolin-2-yl 1-[(4-Fluorophenyl)(hydroxy)methyl]cyclohexane-1-carboxylate (1f)

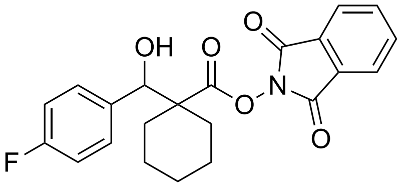

The product **1f** was prepared from 4-fluorobenzaldehyde (631.1 μL, 6.0 mmol) and cyclohexanecarboxylic acid (640.9 mg, 5.0 mmol). The crude residue was purified by flash chromatography on silica gel (Biotage Selekt, 95:5–70:30, hexane/EtOAc) (762.4 mg, 1.92 mmol, 38% isolated yield). White solid. **M.p.** 153–157 °C. **IR** (neat) 696, 970, 1047, 1373, 1509, 1733, 1781, 2864, 2938, 3514 cm^{-1}. **^{1}H NMR** (400 MHz, $CDCl_3$) δ 7.90 (brs, 2H), 7.81 (brs, 2H), 7.40–7.37 (m, 2H), 7.05 (t, J = 8.4 Hz, 2H), 4.99 (s, 1H), 3.23 (brs, 1H), 2.38 (d, J = 13.2 Hz, 1H), 2.25 (d, J = 13.2 Hz, 1H), 1.76–1.46 (m, 5H), 1.27–0.98 (m, 3H). **^{13}C NMR** (100.6 MHz, $CDCl_3$) δ 171.3, 162.5 (d, $J_{C\text{–}F}$ = 246.3 Hz), 162.3, 134.8, 134.6, 129.5 (d, $J_{C\text{–}F}$ = 8.2 Hz), 128.9, 124.0, 114.7 (d, $J_{C\text{–}F}$ = 21.3 Hz), 79.1, 54.3, 31.9, 28.0, 25.5, 23.3, 22.5. **^{19}F NMR** (376 MHz, $CDCl_3$) δ -114.2. **HRMS–DART** (*m/z*): $[M+NH_4]^+$ calcd for $C_{22}H_{24}FN_2O_5^+$, 415.1664; found, 415.1666.

1,3-Dioxoisoindolin-2-yl 1-[(4-Chlorophenyl)(hydroxy)methyl]cyclohexane-1-carboxylate (1g)

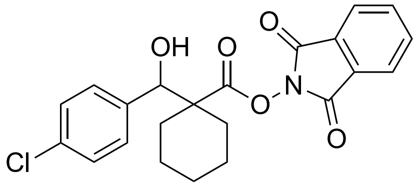

The product **1g** was prepared from 4-chlorobenzaldehyde (843.4 mg, 6.0 mmol) and cyclohexanecarboxylic acid (640.9 mg, 5.0 mmol). The crude residue was purified by flash chromatography on silica gel (Biotage Selekt, 95:5–70:30, hexane/EtOAc) (800.6 mg, 1.93 mmol, 39% isolated yield). White solid. **M.p.** 126–130 °C. **IR** (neat) 727, 877, 904, 970, 1046, 1372, 1734, 1781, 2938, 3510 cm^{-1}. **^{1}H NMR** (400 MHz, $CDCl_3$) δ 7.89 (brs, 2H), 7.81 (brs, 2H), 7.36–7.32 (m, 4H), 4.98 (brs, 1H), 3.25 (m, 1H), 2.37 (d, J = 13.2 Hz, 1H), 2.25 (d, J = 13.2 Hz, 1H), 1.76–1.49 (m, 5H), 1.23 (t, J = 13.2 Hz, 1H), 1.15–0.98 (m, 2H). **^{13}C NMR** (100.6 MHz, $CDCl_3$) δ 171.2, 162.3, 137.4, 134.9, 133.8, 129.2, 128.9, 128.0, 124.0, 79.0, 54.3, 31.9, 27.9, 25.5, 23.3, 22.5. **HRMS–DART** (*m/z*): $[M+NH_4]^+$ calcd for $C_{22}H_{24}ClN_2O_5^+$, 431.1368; found, 431.1364.

1,3-Dioxoisoindolin-2-yl 1-[Hydroxy(3-methoxyphenyl)methyl]cyclohexane-1-carboxylate (1h)

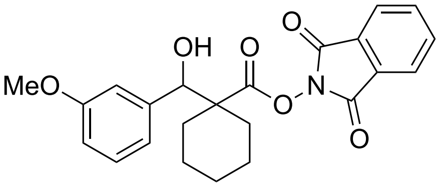

The product **1h** was prepared from *m*-anisaldehyde (729.4 μL, 6.0 mmol) and cyclohexanecarboxylic acid (640.9 mg, 5.0 mmol). The crude residue was purified by flash chromatography on silica gel (Biotage Selekt, 95:5–70:30, hexane/EtOAc) (301.8 mg, 0.74 mmol, 15% isolated yield). White solid. **M.p.** 117–119 °C. **IR** (neat) 695, 877, 970, 1043, 1257, 1585, 1734, 1781, 2938, 3512 cm^{-1}. **^{1}H NMR** (400 MHz, $CDCl_3$) δ 7.88 (brs, 2H), 7.79 (brs, 2H), 7.25 (m, 1H),

6.97 (s, 2H), 6.85 (m, 1H), 4.98 (brs, 1H), 3.81 (s, 3H), 3.24 (m, 1H), 2.38 (d, J = 13.2 Hz, 1H), 2.28 (d, J = 13.2 Hz, 1H), 1.75–1.47 (m, 5H), 1.32–1.15 (m, 2H), 1.02 (m, 1H). **^{13}C NMR** (100.6 MHz, $CDCl_3$) δ 171.4, 162.3, 159.1, 140.6, 134.8, 128.9, 128.7, 124.0, 120.4, 113.5, 113.5, 79.5, 55.2, 54.3, 32.1, 27.9, 25.5, 23.4, 22.6. **HRMS–DART** (*m/z*): $[M+ NH_4]^+$ calcd for $C_{23}H_{27}N_2O_6^+$, 427.1864; found, 427.1864.

1,3-Dioxoisoindolin-2-yl 1-[Benzo[*d*][1,3]dioxol-5-yl(hydroxy)methyl]cyclohexane-1-carboxylate (1i)

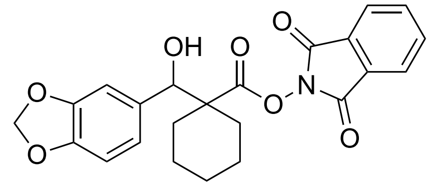

The product **1i** was prepared from piperonal (900.8 mg, 6.0 mmol) and cyclohexanecarboxylic acid (640.9 mg, 5.0 mmol). The crude residue was purified by flash chromatography on silica gel (Biotage Selekt, 90:10–70:30, hexane/EtOAc) (1.37 g, 3.25 mmol, 65% isolated yield). White solid. **M.p.** 147–150 °C. **IR** (neat) 729, 907, 1037, 1242, 1373, 1488, 1734, 1781, 2938, 3516 cm^{-1}. **^{1}H NMR** (400 MHz, $CDCl_3$) δ 7.89 (brs, 2H), 7.81 (brs, 2H), 6.96 (s, 1H), 6.84 (d, J = 8.0 Hz, 1H), 6.78 (d, J = 8.0 Hz, 1H), 5.97 (s, 2H), 4.92 (brs, 1H), 3.11 (m, 1H), 2.35 (d, J = 13.2 Hz, 1H), 2.25 (d, J = 13.2 Hz, 1H), 1.76–1.46 (m, 5H), 1.30–1.00 (m, 3H). **^{13}C NMR** (100.6 MHz, $CDCl_3$) δ 171.4, 162.3, 147.3, 134.8, 132.8, 129.0, 124.0, 121.5, 108.3, 107.5, 101.0, 79.5, 54.4, 32.1, 28.1, 25.6, 23.4, 23.3, 22.6. **HRMS–DART** (*m/z*): $[M+NH_4]^+$ calcd for $C_{23}H_{25}N_2O_7^+$, 441.1656; found, 441.1654.

1,3-Dioxoisoindolin-2-yl 1-[Hydroxy(naphthalen-2-yl)methyl]cyclohexane-1-carboxylate (1j)

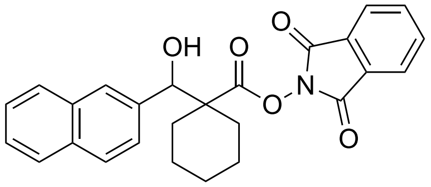

The product **1j** was prepared from 2-naphthaldehyde (937.1 mg, 6.0 mmol) and cyclohexanecarboxylic acid (640.9 mg, 5.0 mmol). The crude residue was purified by GPC (257.5 mg, 0.60 mmol, 12% isolated yield). White solid. **M.p.** 163–167 °C. **IR** (neat) 696, 906, 971, 1049, 1373, 1737, 1781, 2863, 2938, 3512 cm^{-1}. **^{1}H NMR** (400 MHz, $CDCl_3$) δ 7.89–7.80 (m, 8H), 7.56 (d, J = 8.4 Hz, 1H), 7.50–7.49 (m, 2H), 5.19 (s, 1H), 3.27 (brs, 1H), 2.46 (d, J = 13.2 Hz, 1H), 2.36 (d, J = 13.2 Hz, 1H), 1.75–1.47 (m, 5H), 1.35–1.18 (m, 2H), 0.99 (m, 1H). **^{13}C NMR** (100.6 MHz, $CDCl_3$) δ 171.5, 162.7, 136.5, 134.8, 133.2, 132.8, 129.0, 128.2, 127.6, 127.4, 127.1, 126.1 (× 2C), 125.8, 124.0, 79.8, 54.6, 32.2, 27.9, 25.5, 23.4, 22.6. **HRMS–DART** (*m/z*): $[M+ NH_4]^+$ calcd for $C_{26}H_{27}N_2O_5^+$, 447.1915; found, 447.1915.

1,3-Dioxoisoindolin-2-yl 1-[Furan-2-yl(hydroxy)methyl]cyclohexane-1-carboxylate (1k)

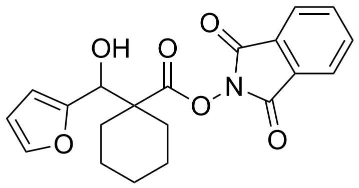

The product **1k** was prepared from furfural (497.0 μL, 6.0 mmol) and cyclohexanecarboxylic acid (640.9 mg, 5.0 mmol). The crude residue was purified by flash chromatography on silica gel (Biotage Selekt, 95:5–70:30, hexane/EtOAc) (497.6 mg, 1.35 mmol, 27% isolated yield). Purple solid. **M.p.** 120–122 °C. **IR** (neat) 695, 729, 970, 1049, 1372, 1734, 1781, 2862, 2936, 3511 cm^{-1}. **^{1}H NMR** (400 MHz, $CDCl_3$) δ 7.89 (brs, 2H), 7.80 (brs, 2H), 7.41 (s, 1H), 6.43 (s, 1H), 6.38 (s, 1H), 4.96 (d, J = 6.8 Hz, 1H), 3.22 (m, 1H), 2.42 (d, J = 12.8 Hz, 1H), 2.25 (d, J = 12.8 Hz, 1H), 1.56 (m, 7H), 1.17 (m, 1H). **^{13}C NMR** (100.6 MHz, $CDCl_3$) δ 171.0, 162.3, 152.6, 142.1, 134.8, 128.9, 124.0, 110.2, 108.4, 74.0, 53.7, 31.6, 28.8, 25.5, 23.1, 22.6. **HRMS–DART** (*m/z*): $[M+NH_4]^+$ calcd for $C_{20}H_{23}N_2O_6^+$, 387.1551; found, 387.1553.

1,3-Dioxoisoindolin-2-yl 1-[Hydroxy(thiophen-2-yl)methyl]cyclohexane-1-carboxylate (1l)

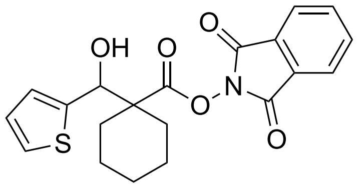

The product **1l** was prepared from 2-thiophenecarboxaldehyde (547.1 μL, 6.0 mmol) and cyclohexanecarboxylic acid (640.9 mg, 5.0 mmol). The crude residue was purified by flash chromatography on silica gel (Biotage Selekt, 95:5–70:30, hexane/EtOAc) (492.7 mg, 1.35 mmol, 26% isolated yield). Pale yellow solid. **M.p.** 133–137 °C. **IR** (neat) 694, 729, 970, 1044, 1373, 1732, 1781, 2862, 2937, 3510 cm^{-1}. **^{1}H NMR** (400 MHz, $CDCl_3$) δ 7.88 (brs, 2H), 7.80 (brs, 2H), 7.29 (m, 1H), 7.10 (s, 1H), 7.02 (m, 1H), 5.28 (s, 1H), 3.42 (brs, 1H), 2.34 (t, J = 12.0 Hz, 2H), 1.78–1.32 (m, 7H), 1.09 (m, 1H). **^{13}C NMR** (100.6 MHz, $CDCl_3$) δ 171.2, 162.5, 142.3, 134.8, 128.9, 126.4, 125.9, 125.1, 124.0, 76.0, 54.3, 31.5, 28.0, 25.5, 23.2, 22.6. **HRMS–DART** (*m/z*): $[M+ NH_4]^+$ calcd for $C_{20}H_{23}N_2O_5S^+$, 403.1322; found, 403.1319.

1,3-Dioxoisoindolin-2-yl 3-Hydroxy-3-(4-methoxyphenyl)-2,2-dimethylbutanoate (1m)

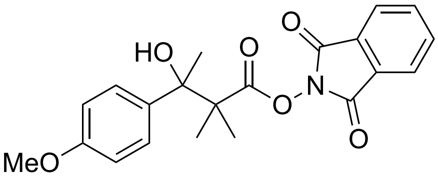

The product **1m** was prepared from 4'-methoxyacetophenone (901.1 mg, 6.0 mmol) and isobutyric acid (463.7 μL, 5.0 mmol). The crude residue was purified by flash chromatography on silica gel (Biotage Selekt, 90:10–70:30, hexane/EtOAc) (387.4 mg, 1.01 mmol, 20% isolated yield). White solid. **M.p.** 145–148 °C. **IR** (neat) 697, 878, 1039, 1184, 1368, 1512, 1738, 1779, 2983, 3541 cm^{-1}. **^{1}H NMR** (400 MHz, $CDCl_3$) δ 7.92–7.89 (m, 2H), 7.82–7.80 (m, 2H), 7.46 (d, J = 8.8 Hz, 2H), 6.89 (d, J = 8.8 Hz, 2H), 3.82 (s, 3H), 3.15 (brs, 1H), 1.81 (s, 3H), 1.35 (s, 3H), 1.30 (s, 3H). **^{13}C NMR**

(100.6 MHz, $CDCl_3$) δ 173.6, 162.3, 158.7, 134.8, 134.5, 128.9, 128.4, 124.0, 112.7, 76.7, 55.2, 51.1, 25.8, 22.0, 21.2. **HRMS–DART** (*m/z*): $[M+NH_4]^+$ calcd for $C_{21}H_{25}N_2O_6^+$, 401.1707; found, 401.1705.

1,3-Dioxoisoindolin-2-yl 1-[Hydroxy(4-methoxyphenyl)methyl]cyclopentane-1-carboxylate (1n)

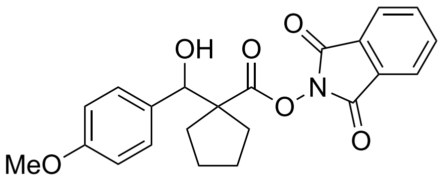

The product **1n** was prepared from *p*–anisaldehyde (729.4 µL, 6.0 mmol) and cyclopentanecarboxylic acid (543.5 µL, 5.0 mmol). The crude residue was purified by GPC (355.9 mg, 0.90 mmol, 18% isolated yield). White solid. **M.p.** 112–113 °C. **IR** (neat) 727, 975, 1184, 1247, 1372, 1512, 1735, 1781, 2958, 3514 cm^{-1}. **^{1}H NMR** (600 MHz, $CDCl_3$) δ 7.91–7.89 (m, 2H), 7.81–7.79 (m, 2H), 7.42–7.40 (m, 2H), 6.92–6.89 (m, 2H), 5.20 (d, *J* = 4.8 Hz, 1H), 3.82 (s, 3H), 2.92 (d, *J* = 4.8 Hz, 1H), 2.33–2.28 (m, 2H), 1.97–1.87 (m, 2H), 1.73–1.64 (m, 2H), 1.51–1.44 (m, 2H). **^{13}C NMR** (100.6 MHz, $CDCl_3$) δ 173.2, 162.3, 159.4, 134.9, 132.1, 129.0, 128.9, 124.1, 113.5, 77.4, 60.3, 55.3, 33.7, 31.1, 25.0, 24.8. **HRMS–DART** (*m/z*): $[M+NH_4]^+$ calcd for $C_{22}H_{25}N_2O_6^+$, 413.1707; found, 413.1706.

1,3-Dioxoisoindolin-2-yl 1-[Hydroxy(4-methoxyphenyl)methyl]cycloheptane-1-carboxylate (1o)

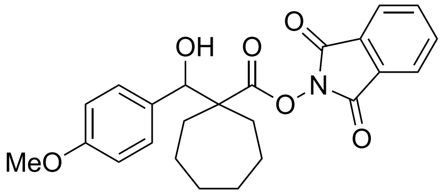

The product **1o** was prepared from *p*–anisaldehyde (729.4 µL, 6.0 mmol) and cycloheptanecarboxylic acid (711.0 mg, 5.0 mmol). The crude residue was purified by GPC (366.7 mg, 0.87 mmol, 17% isolated yield). White solid. **M.p.** 134–136 °C. **IR** (neat) 697, 878, 1003, 1249, 1374, 1512, 1737, 1782, 2927, 3512 cm^{-1}. **^{1}H NMR** (400 MHz, $CDCl_3$) δ 7.90–7.87 (m, 2H), 7.81–7.77 (m, 2H), 7.36 (d, *J* = 8.8 Hz, 2H), 6.88 (d, *J* = 8.8 Hz, 2H), 5.07 (d, *J* = 4.8 Hz, 1H), 3.81 (s, 3H), 3.31 (m, 1H), 2.34–2.29 (m, 2H), 1.76–1.28 (m, 10H). **^{13}C NMR** (100.6 MHz, $CDCl_3$) δ 172.7, 162.7, 162.3, 159.3, 134.8, 131.2, 128.9, 128.9, 124.0, 113.2, 79.2, 56.9, 55.2, 33.9, 29.8, 29.1, 28.7, 23.8, 23.6. **HRMS–DART** (*m/z*): $[M+NH_4]^+$ calcd for $C_{24}H_{29}N_2O_6^+$, 441.2020; found, 441.2022.

1,3-Dioxoisoindolin-2-yl 4-[Hydroxy(4-methoxyphenyl)methyl]tetrahydro-2H-pyran-4-carboxylate (1p)

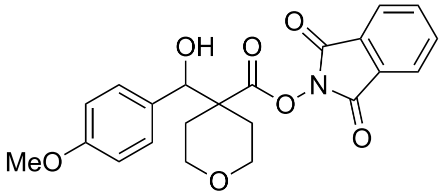

The product **1p** was prepared from *p*–anisaldehyde (729.4 μL, 6.0 mmol) and tetrahydropyran-4-carboxylic acid (650.7 mg, 5.0 mmol). The crude residue was purified by flash chromatography on silica gel (Biotage Selekt, 90:10–70:30, hexane/EtOAc) (592.5 mg, 1.44 mmol, 29% isolated yield). Off white solid. **M.p.** 74–78 °C. **IR** (neat) 727, 908, 1041, 1107, 1248, 1512, 1736, 1781, 2962, 3512 cm^{-1}. **^{1}H NMR** (400 MHz, $CDCl_3$) δ 7.93–7.91 (m, 2H), 7.83–7.81 (m, 2H), 7.34 (d, *J* = 8.8 Hz, 2H), 6.91 (d, *J* = 8.8 Hz, 2H), 4.98 (d, *J* = 4.4 Hz, 1H), 3.96–3.89 (m, 2H), 3.82 (s, 3H), 3.70–3.58 (m, 2H), 2.90 (d, *J* = 4.4 Hz, 1H), 2.28 (dd, *J* = 13.6, 2.0 Hz, 1H), 2.12 (dd, *J* = 13.6, 2.0 Hz, 1H), 1.76–1.63 (m, 2H). **^{13}C NMR** (100.6 MHz, $CDCl_3$) δ 170.9, 162.2, 159.6, 134.9, 130.4, 128.9, 128.8, 124.0, 113.4, 78.9, 65.0, 55.2, 52.1, 31.6, 28.4. **HRMS–DART** (*m/z*): $[M+NH_4]^+$ calcd for $C_{22}H_{25}N_2O_7^+$, 429.1656; found, 429.1658.

1,3-Dioxoisoindolin-2-yl 4-[Hydroxy(4-methoxyphenyl)methyl]-1-tosylpiperidine-4-carboxylate (1q)

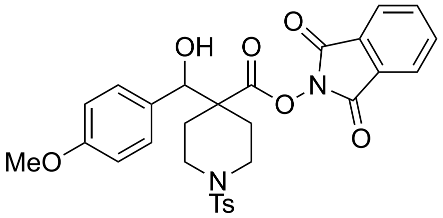

The product **1q** was prepared from *p*–anisaldehyde (607.8 μL, 5.0 mmol) and 1-[(4-methylphenyl)sulfonyl]-4-piperidinecarboxylic acid (1.13 g, 4.0 mmol). The crude residue was purified by GPC (212.4 mg, 0.376 mmol, 9% isolated yield). White solid. **M.p.** 192–195 °C. **IR** (neat) 725, 878, 1039, 1156, 1250, 1342, 1741, 1781, 2936, 3515 cm^{-1}. **^{1}H NMR** (400 MHz, $CDCl_3$) δ 7.84–7.77 (m, 4H), 7.60 (d, *J* = 8.0 Hz, 2H), 7.32–7.27 (m, 4H), 6.89 (d, *J* = 8.0 Hz, 2H), 4.96 (d, *J* = 4.0 Hz, 1H), 3.82 (m, 4H), 3.75 (m, 1H), 2.98 (m, 1H), 2.59–2.49 (m, 2H), 2.44 (s, 3H), 2.38 (dd, *J* = 13.6, 2.0 Hz, 1H), 2.25 (dd, *J* = 13.6, 2.0 Hz, 1H), 1.71-1.59 (m, 2H). **^{13}C NMR** (100.6 MHz, $CDCl_3$) δ 170.3, 161.9, 159.7, 143.4, 134.9, 133.2, 129.9, 129.7, 128.8, 128.8, 127.4, 124.0, 113.6, 78.5, 55.3, 52.2, 43.6, 43.3, 30.7, 27.1, 21.5. **HRMS–DART** (*m/z*): $[M+H]^+$ calcd for $C_{29}H_{29}N_2O_8S^+$, 565.1639; found, 565.1635.

1,3-Dioxoisoindolin-2-yl 2-[Hydroxy(4-methoxyphenyl)methyl]-2-methylbutanoate (1r)

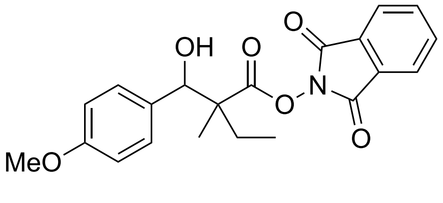

The product **1r** was prepared from *p*–anisaldehyde (607.8 μL, 5.0 mmol) and DL-2-methylbutyric acid (434.6 μL, 4.0 mmol). The crude residue was purified by flash chromatography on silica gel (100:0–95:5, toluene/EtOAc) (788.3 mg, 2.06 mmol, 51% isolated yield). The diastereomeric ratio is 53:47 determined by ^{1}H NMR. White solid. **M.p.** 149–153 °C. **IR** (neat) 834, 1019, 1065, 1186, 1241, 1376, 1511, 1730, 1784, 3516 cm^{-1}. ^{1}**H NMR** (400 MHz, $CDCl_3$) δ 7.88 (brs, 2H), 7.80 (brs, 2H), 7.61 (d, J = 8.0 Hz, 1H), 7.32 (d, J = 8.0 Hz, 1H), 6.89 (d, J = 8.0 Hz, 2H), 5.20 (s, 0.47 × 1H), 5.07 (s, 0.53 × 1H), 3.81 (s, 3H), 3.27 (m, 0.47 × 1H), 2.97 (s, 0.53 × 1H), 2.10 (m, 1H), 1.50 (sextet, J = 7.2 Hz, 0.5 × 1H), 1.28–1.18 (m, 0.53 × 3H + 0.47 × 4H), 1.04 (q, J = 7.2 Hz, 3H). Signals for both diastereomers were given: ^{13}**C NMR** (100.6 MHz, $CDCl_3$) δ 172.4, 172.1, 162.2, 159.3, 134.8, 134.8, 131.2, 131.0, 128.9, 124.0, 123.9, 113.2, 113.2, 78.4, 78.1, 55.2, 55.2, 53.9, 52.8, 29.7, 26.4, 17.7, 14.1, 8.9, 8.7 (Only observed peaks). **HRMS–DART** (*m/z*): $[M+NH_4]^+$ calcd for $C_{21}H_{25}N_2O_6^+$, 401.1707; found, 401.1706.

1,3-Dioxoisoindolin-2-yl 2-[Hydroxy(4-methoxyphenyl)methyl]undecanoate (1s)

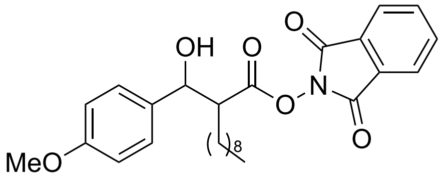

The product **1s** was prepared from *p*–anisaldehyde (607.8 μL, 5.0 mmol) and undecanoic acid (745.2 mg, 4.0 mmol). The crude residue was purified by flash chromatography on silica gel (Biotage Selekt, 93:7–70:30, hexane/EtOAc) (1.20 g, 2.56 mmol, 64% isolated yield). The diastereomeric ratio is 56:44 determined by ^{1}H NMR. Colorless oil. **IR** (neat) 878, 1034, 1248, 1371, 1513, 1740, 1784, 2854, 2924, 3521 cm^{-1}. ^{1}**H NMR** (400 MHz, $CDCl_3$) δ 7.90–7.77 (m, 4H), 7.35 (t, J = 7.6 Hz, 2H), 6.92 (d, J = 7.6 Hz, 2H), 5.15 (brs, 0.44 × 1H), 4.86 (d, J = 9.2 Hz, 0.56 × 1H), 3.82 (s, 3H), 3.08 (m, 1H), 2.87 (brs, 0.56 × 1H), 2.67 (brs, 0.44 × 1H), 1.89–1.47 (m, 2H), 1.24–1.22 (m, 14H), 0.88–0.85 (m, 3H). Signals for both diastereomers were given: ^{13}**C NMR** (100.6 MHz, $CDCl_3$) δ 171.2, 170.6, 162.0, 161.8, 159.6, 159.3, 134.7, 134.7, 133.0, 132.8, 128.9, 128.8, 128.0, 127.4, 124.0, 123.9, 114.0, 113.8, 75.4, 73.6, 55.2, 52.3, 51.2, 31.8, 31.8, 29.5, 29.4, 29.4, 29.3, 29.2, 29.2, 29.2, 27.2, 26.8, 26.7, 22.6, 14.1 (Only observed peaks). **HRMS–DART** (*m/z*): $[M+NH_4]^+$ calcd for $C_{27}H_{37}N_2O_6^+$, 485.2646; found, 485.2647.

1,3-Dioxoisoindolin-2-yl 3-Hydroxy-3-(4-methoxyphenyl)-2-methyl-2-phenylpropanoate (1t)

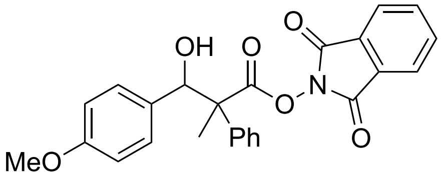

The product **1t** was prepared from *p*–anisaldehyde (607.8 μL, 5.0 mmol) and DL-2-phenylpropionic acid (546.1 μL, 4.0 mmol). The crude residue was purified by flash chromatography on silica gel (Biotage Selekt, 95:5–70:30, hexane/EtOAc) (558.2 mg, 1.29 mmol, 32% isolated yield). White solid. **M.p.** 145–147 °C. **IR** (neat) 730, 1023, 1176, 1248, 1372, 1512, 1736, 1780, 2999, 3522 cm^{-1}. ^{1}**H NMR** (400 MHz, $CDCl_3$) δ 7.89 (brs, 2H), 7.80 (brs, 2H), 7.33–7.26 (m, 5H), 6.74 (d, J =

8.0 Hz, 2H), 6.64 (d, J = 8.0 Hz, 2H), 5.49 (s, 1H), 3.73 (s, 3H), 3.45 (brs, 1H), 1.77 (s, 3H). **^{13}C NMR** (100.6 MHz, $CDCl_3$) δ 173.2, 161.9, 158.9, 137.5, 134.8, 129.6, 128.9, 128.8, 128.4, 128.0, 127.3, 124.0, 112.6, 77.9, 56.7, 55.1, 14.8. **HRMS–DART** (*m/z*): $[M+NH_4]^+$ calcd for $C_{25}H_{25}N_2O_6^+$, 449.1707; found, 449.1709.

1,3-Dioxoisoindolin-2-yl 3-Hydroxy-3-(4-methoxyphenyl)-2-(*p*-tolyl)propanoate (1u)

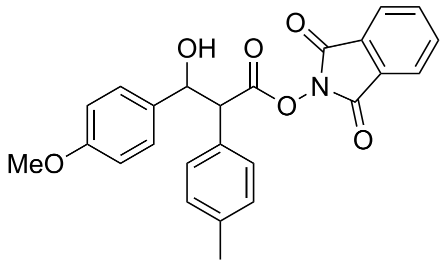

The product **1u** was prepared from *p*–anisaldehyde (607.8 µL, 5.0 mmol) and *p*–tolylacetic acid (600.7 mg, 4.0 mmol). The crude residue was purified by flash chromatography on silica gel (Biotage Selekt, 100:0–97:3, DCM/EtOAc) (741.3 mg, 1.72 mmol, 43% isolated yield). White solid. **M.p.** 189–192 °C. **IR** (neat) 696, 878, 1019, 1065, 1241, 1376, 1511, 1730, 1784, 3000, 3516 cm^{-1}. **^{1}H NMR** (400 MHz, DMSO-d_6) δ 7.94 (brs, 4H), 7.14 (d, J = 8.8 Hz, 2H), 7.10 (d, J = 8.0 Hz, 2H), 7.02 (d, J = 8.0 Hz, 2H), 6.73 (d, J = 8.8 Hz, 2H), 5.89 (d, J = 4.4 Hz, 1H), 5.08 (dd, J = 10.4, 4.4 Hz, 1H), 4.27 (d, J = 10.4 Hz, 1H), 3.96 (s, 3H), 2.21 (s, 3H). **^{13}C NMR** (100.6 MHz, DMSO-d_6) δ 169.6, 161.6, 158.4, 136.8, 135.5, 133.8, 130.9, 129.0, 128.7, 128.4, 128.2, 123.9, 113.1, 74.4, 56.1, 54.9, 20.6. **HRMS–DART** (*m/z*): $[M+NH_4]^+$ calcd for $C_{25}H_{25}N_2O_6^+$, 449.1707; found, 449.1708.

***tert*-Butyl 3-{1-[(1,3-Dioxoisoindolin-2-yl)oxy]-2-methyl-1-oxopropan-2-yl}-3-hydroxypiperidine-1-carboxylate (1v)**

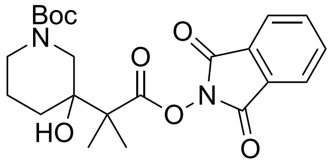

The product **1v** was prepared from 1-(*tert*-butoxycarbonyl)-3-piperidone (2.59 g, 13.0 mmol) and isobutyric acid (927.5 µL, 10.0 mmol). The crude residue was purified by flash chromatography on silica gel (Biotage Selekt, 90:10–60:40, hexane/EtOAc) (1.31 g, 3.02 mmol, 30% isolated yield). White solid. **M.p.** 43–47 °C. **IR** (neat) 727, 907, 1059, 1152, 1276, 1426, 1673, 1740, 1778, 2978 cm^{-1}. Signals for two rotamers (51:49) were given: **^{1}H NMR** (400 MHz, $CDCl_3$) δ 7.91–7.87 (m, 2H), 7.82–7.78 (m, 2H), 4.24–4.11 (m, 2H), 3.10 (s, 0.51 × 1H), 3.06 (s, 0.49 × 1H), 2.73–2.69 (m, 2H), 1.98–1.86 (m, 2H), 1.74–1.54 (m, 3H), 1.48–1.47 (m, 15H). Signals for both rotamers were given: **^{13}C NMR** (100.6 MHz, $CDCl_3$) δ 172.4, 161.9, 156.6, 134.7, 128.7, 123.8, 79.7, 72.9, 49.4, 49.2, 44.7, 43.7, 30.0, 28.2, 21.0, 20.2, 20.1 (Only observed peaks). **HRMS–DART** (*m/z*): $[M+H]^+$ calcd for $C_{22}H_{29}N_2O_7^+$, 433.1969; found, 433.1968.

tert-Butyl 3-{1-[(1,3-Dioxoisoindolin-2-yl)oxy]-2-methyl-1-oxobutan-2-yl}-3-hydroxypiperidine-1-carboxylate (1w)

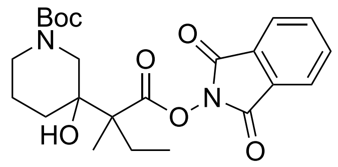

The product **1w** was prepared from 1-(_tert_-butoxycarbonyl)-3-piperidone (996.3 mg, 5.0 mmol) and DL-2-methylbutyric acid (434.6 μL, 4.0 mmol). The crude residue was purified by flash chromatography on silica gel (Biotage Selekt, 90:10–70:30, hexane/EtOAc) (Fig. 2, 178.6 mg, 0.40 mmol, 10% isolated yield). White solid. **M.p.** 56–60 °C. **IR** (neat) 728, 907, 986, 1154, 1365, 1672, 1740, 1777, 2975, 3467 cm^{-1}. Signals for two rotamers (72:28) were given: **^{1}H NMR** (400 MHz, $CDCl_3$) δ 7.89–7.87 (m, 2H), 7.81–7.80 (m, 2H), 4.27–4.12 (m, 2H), 3.17 (d, J = 13.6 Hz, 0.72 × 1H), 2.97 (d, J = 13.6 Hz, 0.28 × 1H), 2.71 (brs, 2H), 2.28 (m, 1H), 2.12–1.55 (m, 5H), 1.47–1.47 (m, 9H), 1.41 (brs, 3H), 1.11–1.05 (m, 3H). Signals for both rotamers were given: **^{13}C NMR** (100.6 MHz, $CDCl_3$) δ 172.0, 171.8, 162.0, 156.8, 155.7, 134.7, 128.9, 123.9, 79.9, 73.6, 54.5, 54.4, 49.5, 45.0, 43.9, 30.3, 28.3, 25.8, 25.6, 21.2, 16.0, 15.6, 9.2 (Only observed peaks). **HRMS–DART** (_m/z_): $[M+H]^+$ calcd for $C_{23}H_{31}N_2O_7^+$, 447.2126; found, 447.2126.

tert-Butyl 3-(1-{[(1,3-Dioxoisoindolin-2-yl)oxy]carbonyl}cyclobutyl)-3-hydroxypiperidine-1-carboxylate (1x)

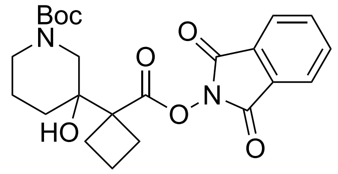

The product **1x** was prepared from 1-(_tert_-butoxycarbonyl)-3-piperidone (996.3 mg, 5.0 mmol) and cyclobutanecarboxylic acid (377.8 μL, 4.0 mmol). The crude residue was purified by flash chromatography on silica gel (Biotage Selekt, 93:7–60:40, hexane/EtOAc) (592.1 mg, 1.332 mmol, 33% isolated yield). White solid. **M.p.** 57–61 °C. **IR** (neat) 727, 906, 1005, 1155, 1366, 1427, 1670, 1740, 1777, 2975 cm^{-1}. Signals for two rotamers (50:50) were given: **^{1}H NMR** (400 MHz, $CDCl_3$) δ 7.92–7.88 (m, 2H), 7.83–7.79 (m, 2H), 4.09 (brs, 2H), 3.14 (s, 0.50 × 1H), 3.10 (s, 0.50 × 1H), 2.76–2.56 (m, 6H), 2.08 (m, 1H), 1.98–1.80 (m, 3H), 1.69–1.58 (m, 2H), 1.48 (s, 9H). Signals for both rotamers were given: **^{13}C NMR** (100.6 MHz, $CDCl_3$) δ 172.5, 161.9, 156.6, 134.7, 128.7, 123.7, 79.7, 71.4, 54.4, 48.9, 44.7, 29.6, 28.1, 26.3, 20.9, 16.3 (Only observed peaks). **HRMS–DART** (_m/z_): $[M+H]^+$ calcd for $C_{23}H_{29}N_2O_7^+$, 445.1969; found, 445.1969.

tert-Butyl 3-{2-[(1,3-Dioxoisoindolin-2-yl)oxy]-2-oxo-1-(phenylthio)ethyl}-3-hydroxypiperidine-1-carboxylate (1y)

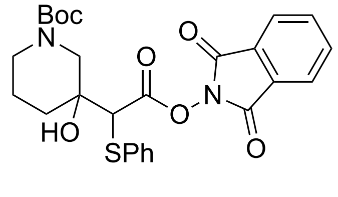

The product **1y** was prepared from 1-(*tert*-butoxycarbonyl)-3-piperidone (996.3 mg, 5.0 mmol) and (phenylthio)acetic acid (673.0 mg, 4.0 mmol). The crude residue was purified by flash chromatography on silica gel (Biotage Selekt, 90:10–70:30, hexane/EtOAc) (1.05 g, 2.06 mmol, 51% isolated yield). Yellow solid. **M.p.** 62–65 °C. **IR** (neat) 908, 967, 1057, 1147, 1271, 1425, 1674, 1741, 1784, 2975 cm^{-1}. Signals for two rotamers were given: ^{1}**H NMR** (400 MHz, $CDCl_3$) δ 7.90–7.87 (m, 2H), 7.82–7.70 (m, 4H), 7.41–7.36 (m, 3H), 4.20–2.76 (m, 6H), 2.24–1.74 (m, 3H), 1.60 (m, 1H), 1.48–1.43 (m, 9H). Signals for both rotamers were given: ^{13}**C NMR** (100.6 MHz, $CDCl_3$) δ 168.4, 167.9, 161.5, 155.4, 134.9, 133.9, 132.7, 129.4, 129.3, 129.0, 128.9, 128.8, 128.7, 124.0, 80.2, 80.0, 71.9, 59.5, 57.5, 50.5, 43.9, 34.6, 32.8, 28.32, 28.31, 21.5, 20.9 (Only observed peaks). **HRMS–DART** (*m/z*): $[M+NH_4]^+$ calcd for $C_{26}H_{32}N_3O_7S^+$, 530.1956; found, 530.1960.

1,3-Dioxoisoindolin-2-yl 2-(9-Hydroxy-9H-xanthen-9-yl)-2-methylpropanoate (1z)

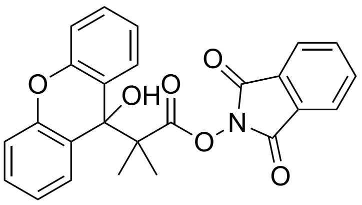

The product **1z** was prepared from xanthone (1.18 g, 6.0 mmol) and isobutyric acid (463.7 µL, 5.0 mmol). The crude residue was purified by GPC (Fig. 2, 458.0 mg, 1.07 mmol, 21% isolated yield). White solid. **M.p.** 136–139 °C. **IR** (neat) 727, 760, 907, 1047, 1238, 1447, 1735, 1777, 2983, 3502 cm^{-1}. ^{1}**H NMR** (400 MHz, $CDCl_3$) δ 7.92–7.89 (m, 2H), 7.85–7.79 (m, 4H), 7.39–7.35 (m, 2H), 7.26–7.17 (m, 4H), 3.79 (m, 1H), 1.25 (s, 6H). ^{13}**C NMR** (100.6 MHz, $CDCl_3$) δ 172.7, 161.9, 151.7, 134.7, 129.3, 128.9, 127.6, 123.9, 123.9, 123.2, 116.2, 73.0, 53.8, 21.1. **HRMS–DART** (*m/z*): $[M+NH_4]^+$ calcd for $C_{25}H_{23}N_2O_6^+$, 447.1551; found, 447.1550.

1,3-Dioxoisoindolin-2-yl 2-(1-Hydroxycyclododecyl)-2-methylpropanoate (1A)

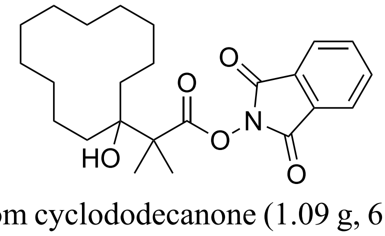

The product **1A** was prepared from cyclododecanone (1.09 g, 6.0 mmol) and isobutyric acid (463.7 µL, 5.0 mmol). The crude residue was purified by flash chromatography on silica gel (Biotage Selekt, 97:3–80:20, hexane/EtOAc) (910.4 mg, 2.19 mmol, 44% isolated yield). White solid. **M.p.** 107–109 °C. **IR** (neat) 727, 906, 1038, 1370, 1468, 1738, 1778, 1804, 2862, 2930 cm^{-1}. ^{1}**H NMR** (400 MHz, $CDCl_3$) δ 7.89–7.87 (m, 2H), 7.80–7.78 (m, 2H), 2.51 (s, 1H), 1.81–1.66 (m, 4H), 1.53–1.40 (m, 24H). ^{13}**C NMR** (100.6 MHz, $CDCl_3$) δ 173.5, 162.0, 134.7, 128.8, 123.9, 77.3, 51.4, 31.9, 26.9, 26.1, 22.7, 22.1, 21.4, 20.5. **HRMS–DART** (*m/z*): $[M+NH_4]^+$ calcd for $C_{24}H_{37}N_2O_5^+$, 433.2697; found, 433.2697.

1,3-Dioxoisoindolin-2-yl 4-(3-Hydroxyoxetan-3-yl)-1-tosylpiperidine-4-carboxylate (1B)

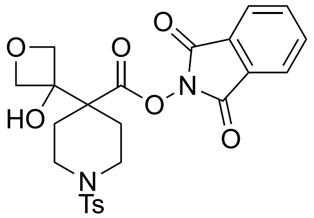

The product **1B** was prepared from 3-oxetanone (310.6 μL, 5.0 mmol) and 1-[(4-methylphenyl)sulfonyl]-4-piperidinecarboxylic acid (1.13 g, 4.0 mmol). The crude residue was purified by flash chromatography on silica gel (Biotage Selekt, 100:0–80:20, DCM/EtOAc) (416.4 mg, 0.83 mmol, 21% isolated yield). White solid. **M.p.** 192–194 °C. **IR** (neat) 727, 876, 937, 1039, 1163, 1327, 1740, 1783, 2933, 3336 cm^{-1}. **^{1}H NMR** (400 MHz, $CDCl_3$) δ 7.86–7.83 (m, 2H), 7.82–7.79 (m, 2H), 7.67 (d, J = 8.0 Hz, 2H), 7.35 (d, J = 8.0 Hz, 2H), 4.83 (d, J = 8.0 Hz, 2H), 4.56 (d, J = 8.0 Hz, 2H), 3.97–3.93 (m, 2H), 3.57 (brs, 1H), 2.56 (td, J = 12.8, 2.0 Hz, 2H), 2.48–2.45 (m, 5H), 1.89 (td, J = 12.8, 4.4 Hz, 2H). **^{13}C NMR** (100.6 MHz, $CDCl_3$) δ 169.4, 161.9, 143.8, 135.1, 133.3, 129.8, 128.6, 127.5, 124.2, 79.1, 78.7, 50.5, 43.4, 27.6, 21.5. **HRMS–DART** (*m/z*): $[M+NH_4]^+$ calcd for $C_{24}H_{28}N_3O_8S^+$, 518.1592; found, 518.1588.

1,3-Dioxoisoindolin-2-yl 4-(1-Hydroxycyclobutyl)-1-tosylpiperidine-4-carboxylate (1C)

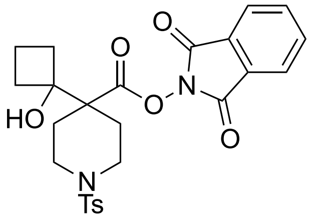

The product **1C** was prepared from cyclobutanone (376.8 μL, 5.0 mmol) and 1-[(4-methylphenyl)sulfonyl]-4-piperidinecarboxylic acid (1.13 g, 4.0 mmol). The crude residue was purified by flash chromatography on silica gel (Biotage Selekt, 100:0–90:10, toluene/EtOAc) (138.6 mg, 0.28 mmol, 7% isolated yield). White solid. **M.p.** 179–182 °C. **IR** (neat) 548, 723, 908, 1034, 1158, 1342, 1737, 1780, 2947, 3510 cm^{-1}. **^{1}H NMR** (400 MHz, $CDCl_3$) δ 7.85–7.82 (m, 2H), 7.81–7.78 (m, 2H), 7.72 (d, J = 8.0 Hz, 2H), 7.35 (d, J = 8.0 Hz, 2H), 3.92–3.88 (m, 2H), 2.81 (s, 1H), 2.52–2.34 (m, 9H), 2.15 (m, 1H), 1.97–1.90 (m, 2H), 1.82–1.68 (m, 3H). **^{13}C NMR** (100.6 MHz, $CDCl_3$) δ 169.8, 161.9, 143.5, 134.9, 133.1, 129.7, 128.5, 127.4, 123.9, 80.0, 52.4, 43.6, 30.7, 27.5, 21.4, 13.3. **HRMS–DART** (*m/z*): $[M+NH_4]^+$ calcd for $C_{25}H_{30}N_3O_7S^+$, 516.1799; found, 516.1796.

***tert*-Butyl 3-(1-{[(1,3-Dioxoisoindolin-2-yl)oxy]carbonyl}cyclohexyl)-3-hydroxyazetidine-1-carboxylate (1D)**

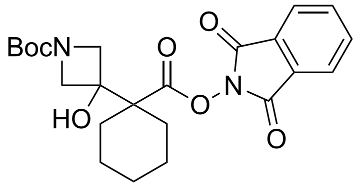

The product **1D** was prepared from 1-(*tert*-Butoxycarbonyl)-3-azetidinone (856.0 mg, 5.0 mmol) and cyclohexanecarboxylic acid (512.7 mg, 4.0 mmol). The crude residue was purified by flash chromatography on silica gel (Biotage Selekt, 90:10–70:30, hexane/EtOAc) (450.2 mg, 1.01 mmol,

25% isolated yield). White solid. **M.p.** 79–82 °C. **IR** (neat) 697, 970, 1061, 1160, 1415, 1677, 1741, 1811, 2935, 3381 cm^{-1}. **^{1}H NMR** (400 MHz, $CDCl_3$) δ 7.89 (brs, 2H), 7.82 (brs, 2H), 4.24 (d, J = 10.0 Hz, 2H), 3.80 (d, J = 10.0 Hz, 2H), 3.51 (brs, 1H), 2.43 (d. J = 13.2 Hz, 2H), 1.84–1.77 (m, 3H), 1.59–1.15 (m, 14H). **^{13}C NMR** (100.6 MHz, $CDCl_3$) δ 170.4, 162.4, 156.2, 135.0, 128.8, 124.2, 79.8, 75.2, 58.4, 57.6, 53.3, 28.3 (×2C), 25.3, 22.9. **HRMS–DART** (*m/z*): [M+H]$^+$ calcd for $C_{23}H_{29}N_2O_7^+$, 445.1969; found, 445.1969.

1,3-Dioxoisoindolin-2-yl 2-[(*tert*-Butoxycarbonyl)amino]-2-(1-hydroxycyclobutyl)acetate (1E)

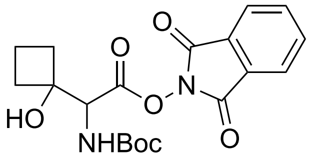

The product **1E** was prepared from cyclobutanone (376.8 μL, 5.0 mmol) and *N*-(*tert*-butoxycarbonyl)glycine (700.7 mg, 4.0 mmol). The crude residue was purified by flash chromatography on silica gel (Biotage Selekt, 90:10–70:30, hexane/EtOAc) (410.0 mg, 1.05 mmol, 26% isolated yield). White solid. **M.p.** 128–130 °C. **IR** (neat) 966, 1049, 1159, 1367, 1501, 1710, 1740, 1788, 2979, 3433 cm^{-1}. Signals for two rotamers (77:23) were given: **^{1}H NMR** (400 MHz, $CDCl_3$) δ 7.90–7.88 (m, 2H), 7.82–7.80 (m, 2H), 5.48 (m, 0.77 × 1H), 5.25 (brs, 0.23 × 1H), 4.83 (d, J = 9.6 Hz, 0.77 × 1H), 4.51 (m, 0.23 × 1H), 3.09 (brs, 1H), 2.59 (m, 1H), 2.26–1.96 (m, 4H), 1.79 (m, 1H), 1.55–1.44 (m, 9H). Signals for both rotamers were given: **^{13}C NMR** (100.6 MHz, $CDCl_3$) δ 167.3, 161.8, 155.7, 135.0, 128.7, 124.1, 81.7, 80.5, 76.6, 75.5, 60.5, 59.0, 33.5, 33.0, 32.6, 28.2, 28.0, 12.3 (Only observed peaks). **HRMS–DART** (*m/z*): [M+NH$_4$]$^+$ calcd for $C_{19}H_{26}N_3O_7^+$, 408.1765; found, 408.1767.

1,3-Dioxoisoindolin-2-yl 2-(3-Hydroxyoxetan-3-yl)-2-(phenylthio)acetate (1F)

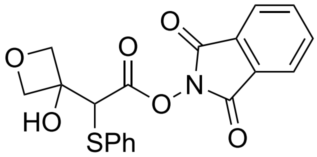

The product **1F** was prepared from 3-oxetanone (310.6 μL, 5.0 mmol) and (phenylthio)acetic acid (673.0 mg, 4.0 mmol). The crude residue was washed with $CHCl_3$ to yield pure product (867.3 mg, 2.25 mmol, 56% isolated yield). White solid. **M.p.** 159–161 °C. **IR** (neat) 695, 877, 973, 1185, 1371, 1468, 1741, 1785, 2951, 3368 cm^{-1}. **^{1}H NMR** (400 MHz, $CDCl_3$) δ 7.92–7.89 (m, 2H), 7.84–7.81 (m, 2H), 7.75–7.73 (m, 2H), 7.41–7.40 (m, 2H), 4.83–4.72 (m, 4H), 4.39 (s, 1H), 3.53 (brs, 1H). **^{13}C NMR** (100.6 MHz, $CDCl_3$) δ 168.3, 161.5, 135.1, 134.3, 131.8, 129.6, 129.4, 128.7, 124.2, 81.3, 81.0, 75.0, 56.0. **HRMS–DART** (*m/z*): [M+NH$_4$]$^+$ calcd for $C_{19}H_{19}N_2O_6S^+$, 403.0958; found, 403.0957.

1,3-Dioxoisoindolin-2-yl 2-(1-Hydroxycyclopentyl)-2-(4-methoxyphenyl)acetate (1G)

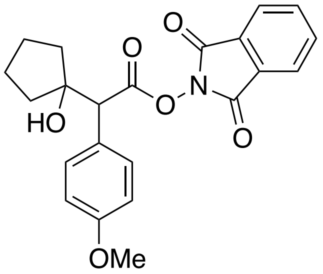

The product **1G** was prepared from cyclopentanone (442.7 μL, 5.0 mmol) and 4-methoxyphenylacetic acid (664.7 mg, 4.0 mmol). The crude residue was purified by flash chromatography on silica gel (Biotage Selekt, 97:3–70:30, hexane/EtOAc) (825.4 mg, 2.09 mmol, 52% isolated yield). Clear sticky oil. **IR** (neat) 877, 969, 1064, 1182, 1249, 1358, 1512, 1737, 1783, 2958, 3525 cm^{-1}. **^{1}H NMR** (400 MHz, $CDCl_3$) δ 7.89–7.86 (m, 2H), 7.82–7.77 (m, 2H), 7.43–7.39 (m, 2H), 6.93–6.90 (m, 2H), 4.05 (s, 1H), 3.82 (s, 3H), 2.47 (s, 1H), 1.94–1.49 (m, 8H). **^{13}C NMR** (100.6 MHz, $CDCl_3$) δ 169.7, 161.9, 159.4, 134.8, 130.6, 128.8, 125.9, 124.0, 113.9, 83.2, 56.3, 55.2, 39.3, 37.6, 23.6, 23.3. **HRMS–DART** (*m/z*): $[M+NH_4]^+$ calcd for $C_{23}H_{27}N_2O_6^+$, 427.1864; found, 427.1864.

1,3-Dioxoisoindolin-2-yl 2-[1-Hydroxy-4-(4-methoxyphenyl)cyclohexyl]-2-methylpropanoate (1H)

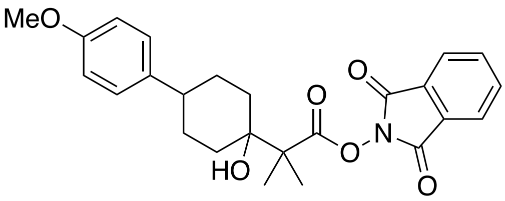

The product **1H** was prepared from 4-(4-methoxyphenyl)cyclohexanone (766.0 mg, 3.75 mmol) and isobutyric acid (278.2 μL, 3.0 mmol). The crude residue was purified by flash chromatography on silica gel (Biotage Selekt, 97:3–70:30, hexane/EtOAc) (714.6 mg, 1.63 mmol, 54% isolated yield). White solid. **M.p.** 156–158 °C. **IR** (neat) 831, 1041, 1182, 1246, 1369, 1512, 1738, 1778, 2934, 3537 cm^{-1}. **^{1}H NMR** (400 MHz, $CDCl_3$) δ 7.92–7.88 (m, 2H), 7.82–7.78 (m, 2H), 7.20–7.17 (m, 2H), 6.87–6.83 (m, 2H), 3.79 (s, 3H), 2.50–2.42 (m, 2H), 2.00–1.71 (m, 8H), 1.49 (s, 6H). **^{13}C NMR** (100.6 MHz, $CDCl_3$) δ 173.5, 162.2, 157.8, 139.2, 134.9, 128.9, 127.7, 124.0, 113.7, 73.7, 55.2, 51.0, 42.9, 31.6, 29.4, 20.7. **HRMS–DART** (*m/z*): $[M+NH_4]^+$ calcd for $C_{23}H_{27}N_2O_6^+$, 427.1864; found, 427.1864.

4. General Procedures for Organophotoredox–Catalyzed Semipinacol Rearrangement

The reaction to produce 2a in Table 1, entry 1 is representative. In a glovebox, to an oven-dried vial with a stirring bar was added **PTH1** (3.3 mg, 0.01 mmol), lithium tetrafluoroborate (0.9 mg, 0.01 mmol), *β*-hydroxyester **1a** (81.9 mg, 0.2 mmol). Then, ethyl acetate (600 μL) was added to the reaction mixture. After sealing the vial with a cap and removed from the glove box, the reaction was stirred and irradiated with a 34W blue LED (0.5 cm away) with a cooling fan to keep the temperature around 40 °C (Supplementary Figure 2). After 24 h, the reaction was quenched by 0.5 M NaOH aq. solution (1.5 mL). The aqueous layer was thrice extracted with dichloromethane (500 μL), dried over Na_2SO_4 and then, filtered. After volatiles were removed under reduced pressure, purification by flash column chromatography on silica gel (100:0–90:10, hexane/EtOAc) gave the rearrangement product **2a** (39.6 mg, 0.182 mmol, 91% isolated yield).

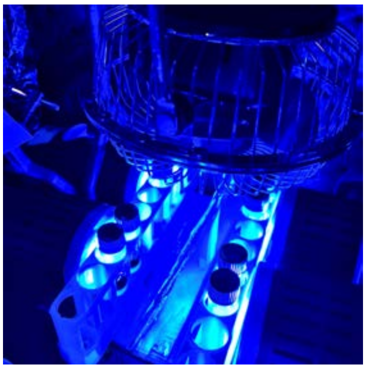

Supplementary Figure 2. Light set up

The reaction to produce 2v in Fig. 2, right is representative (ring expansion). In a glovebox, to an oven-dried vial with a stirring bar was added **PTH1** (6.6 mg, 0.02 mmol), lithium tetrafluoroborate (1.9 mg, 0.02 mmol), *β*-hydroxyester **1v** (86.5 mg, 0.2 mmol). Then, dichloromethane (600 μL) was added to the reaction mixture. After sealing the vial with a cap and removed from the glove box, the reaction was stirred and irradiated with a 34W blue LED (0.5 cm away) with a cooling fan to keep the temperature around 40 °C (Supplementary Figure 2). After 24 h, the reaction was quenched by 0.5 M NaOH aq. solution (1.5 mL). The aqueous layer was thrice extracted with dichloromethane (500 μL), dried over Na_2SO_4 and then, filtered. After volatiles were removed under reduced pressure, purification by flash column chromatography on silica gel (100:0–80:20, hexane/EtOAc) gave the rearrangement product **2v** (31.4 mg, 0.130 mmol, 65% isolated yield).

1 mmol scale reaction of 2y. In a glovebox, to an oven-dried vial with a stirring bar was added **PTH1** (32.3 mg, 0.10 mmol), lithium tetrafluoroborate (9.4 mg, 0.10 mmol), *β*-hydroxyester **1y** (512.6 mg, 1.0 mmol). Then, ethyl acetate (3.0 mL) was added to the reaction mixture. After sealing the vial with a cap and removed from the glove box, the reaction was stirred and irradiated with a 34W blue LED (0.5 cm away) with a cooling fan to keep the temperature around 40 °C (Supplementary Figure 2). After 24 h, the reaction was quenched by 0.5 M NaOH aq. solution (5.0 mL). The aqueous layer was thrice extracted with dichloromethane (2.5 mL), dried over Na_2SO_4 and then, filtered. After volatiles were removed under reduced pressure, purification by flash column chromatography on silica gel (Biotage Selekt, 97:3–85:15, hexane/EtOAc) gave the rearrangement product **2y** (184.5 mg, 0.574 mmol, 57% isolated yield).

5. Characterization Data for Rearrangement Products

1-(4-Methoxyphenyl)cyclohexane-1-carbaldehyde (2a)

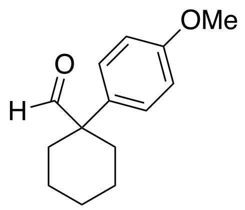

The product **2a** was purified by flash chromatography on silica gel (100:0–90:10, hexane/EtOAc) (Table 1, entry 1; 39.6 mg, 0.18 mmol, 91% isolated yield). The spectrum data of product **2a** was consistent with the literature.[5]

1-(*p*-Tolyl)cycloheptane-1-carbaldehyde (2b)

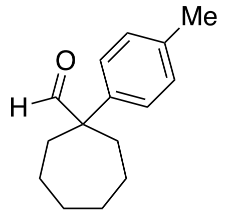

The product **2b** was purified by flash chromatography on silica gel (Biotage Selekt, 100:0–97:3, hexane/EtOAc) (Fig. 2, 29.9 mg, 0.138 mmol, 69% isolated yield). Colorless oil. **IR** (neat) 536, 721, 810, 904, 1463, 1512, 1724, 1970, 2857, 2922 cm^{-1}. **^{1}H NMR** (600 MHz, $CDCl_3$) δ 9.33 (s, 1H), 7.19–7.15 (m, 4H), 2.33 (s, 3H), 2.23–2.19 (m, 2H), 2.08–2.04 (m, 2H), 1.64–1.53 (m, 8H). **^{13}C NMR** (150.9 MHz, $CDCl_3$) δ 201.7, 138.0, 136.7, 129.6, 127.2, 57.4, 32.9, 29.9, 23.0, 20.9. **HRMS–DART** (*m/z*): $[M+NH_4]^+$ calcd for $C_{15}H_{24}NO^+$, 234.1852; found, 234.1851.

1-[4-(*tert*-Butyl)phenyl]cyclohexane-1-carbaldehyde (2c)

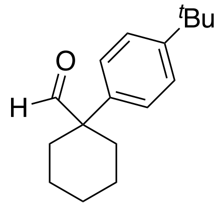

The product **2c** was purified by flash chromatography on silica gel (100:0–97:3, hexane/EtOAc) (Fig. 2, 34.3 mg, 0.140 mmol, 70% isolated yield). The spectrum data of product **2c** was consistent with the literature.[6]

1-[4-(Methylthio)phenyl]cyclohexane-1-carbaldehyde (2d)

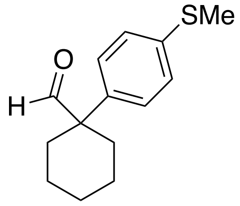

The product **2d** was purified by flash chromatography on silica gel (100:0–97:3, hexane/EtOAc) (Fig. 2, 30.0 mg, 0.128 mmol, 64% isolated yield). The spectrum data of product **2d** was consistent with the literature.[5]

{1-[4-(Trifluoromethoxy)phenyl]cyclohexyl}methanol (2e)

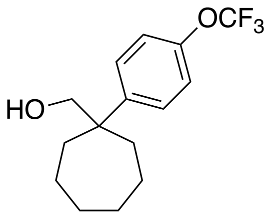

The rearrangement product was isolated as the alcohol **2e** through reduction by $NaBH_4$ (0.4 mmol) in MeOH (5.0 mL). The product **2e** was purified by flash chromatography on silica gel (100:0–95:5, hexane/EtOAc) (Fig. 2, 13.5 mg, 0.047 mmol, 23% isolated yield). Colorless oil. **IR** (neat) 1018, 1061, 1161, 1213, 1259, 1472, 1509, 2859, 2924, 3340 cm^{-1}. **^{1}H NMR** (400 MHz, $CDCl_3$) δ 7.40–7.37 (m, 2H), 7.19 (d, J = 8.0 Hz, 2H), 3.50 (s, 2H), 2.16–2.10 (m, 2H), 1.79–1.73 (m, 2H), 1.68–1.42 (m, 8H). **^{13}C NMR** (100.6 MHz, $CDCl_3$) δ 147.4 (m), 144.4, 128.6, 120.7, 120.5 (q, J_{C-F} = 256.9 Hz), 73.1, 46.9, 35.1, 29.8, 23.2. **^{19}F NMR** (376 MHz, $CDCl_3$) δ –57.8. **HRMS–DART** (*m/z*): $[M+NH_4]^+$ calcd for $C_{15}H_{23}F_3NO_2$, 306.1675; found, 306.1677.

1-(4-Fluorophenyl)cyclohexane-1-carbaldehyde (2f)

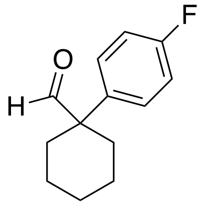

The product **2f** was purified by flash chromatography on silica gel (100:0–97:3, hexane/EtOAc) (Fig. 2, 18.5 mg, 0.090 mmol, 45% isolated yield). The spectrum data of product **2f** was consistent with the literature.[7]

1-(4-Chlorophenyl)cyclohexane-1-carbaldehyde (2g)

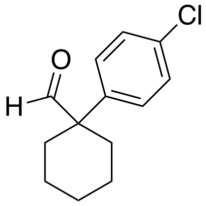

The product **2g** was purified by flash chromatography on silica gel (100:0–97:3, hexane/EtOAc) (Fig. 2, 14.4 mg, 0.065 mmol, 32% isolated yield). The spectrum data of product **2g** was consistent with the literature.[7]

1-(3-Methoxyphenyl)cyclohexane-1-carbaldehyde (2h)

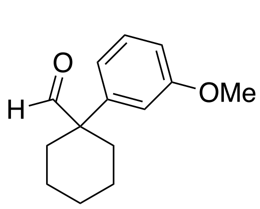

The product **2h** was purified by flash chromatography on silica gel (100:0–95:5, hexane/EtOAc) (Fig. 2, 34.2 mg, 0.157 mmol, 78% isolated yield). The spectrum data of product **2h** was consistent with the literature.[5]

1-(Benzo[*d*][1,3]dioxol-5-yl)cyclohexane-1-carbaldehyde (2i)

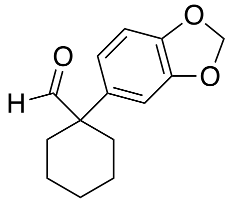

The product **2i** was purified by flash chromatography on silica gel (100:0–97:3, hexane/EtOAc) (Fig. 2, 37.5 mg, 0.162 mmol, 81% isolated yield). Colorless oil. **IR** (neat) 808, 936, 1040, 1239, 1451, 1486, 1565, 1721, 2857, 2933 cm^{-1}. **^{1}H NMR** (600 MHz, $CDCl_3$) δ 9.30 (s, 1H), 6.81 (d, J = 1.8 Hz, 1H), 6.80 (s, 1H), 6.77 (m, 1H), 5.95 (s, 2H), 2.25–2.23 (m, 2H), 1.80–1.75 (m, 2H), 1.69–1.63 (m, 2H), 1.60 (m, 1H), 1.49–1.42 (m, 2H), 1.29 (m, 1H). **^{13}C NMR** (150.9 MHz, $CDCl_3$) δ 202.0, 148.3, 146.7, 133.5, 120.6, 108.5, 107.6, 101.1, 54.0, 31.5, 25.6, 22.8. **HRMS–DART** (*m/z*): $[M+NH_4]^+$ calcd for $C_{14}H_{20}NO_3^+$, 250.1438; found, 250.1437.

1-(Naphthalen-2-yl)cyclohexane-1-carbaldehyde (2j)

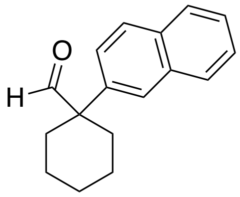

The product **2j** was purified by flash chromatography on silica gel (100:0–95:5, hexane/EtOAc) (Fig. 2, 36.8 mg, 0.155 mmol, 77% isolated yield). Colorless oil. **IR** (neat) 746, 852, 960, 1450, 1597, 1720, 2855, 2932, 3056 cm^{-1}. **^{1}H NMR** (600 MHz, $CDCl_3$) δ 9.43 (s, 1H), 7.84–7.80 (m, 3H), 7.78 (s, 1H), 7.50–7.46 (m, 2H), 7.43(dd, J = 8.4, 1.8 Hz, 1H), 2.42–2.39 (m, 2H), 1.99–1.94 (m, 2H), 1.74–1.69 (m, 2H), 1.64 (m, 1H), 1.57–1.50 (m, 2H), 1.35 (m, 1H). **^{13}C NMR** (150.9 MHz, $CDCl_3$) δ 202.4, 137.1, 133.4, 132.3, 128.5, 128.0, 127.5, 126.3, 126.2, 126.2, 124.9, 54.5, 31.3, 25.6, 22.8. **HRMS–DART** (*m/z*): $[M+NH_4]^+$ calcd for $C_{17}H_{22}NO^+$, 256.1696; found, 256.1698.

[1-(Furan-2-yl)cyclohexyl]methanol (2k)

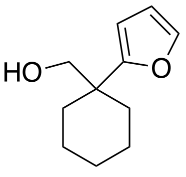

The rearrangement product was isolated as the alcohol **2k** through reduction by $NaBH_4$ (0.4 mmol) in MeOH (5.0 mL). The product **2k** was purified by flash chromatography on silica gel (97:3–80:20, hexane/Et_2O) (Fig. 2, 17.5 mg, 0.097 mmol, 49% isolated yield). Colorless oil. **IR** (neat) 729, 902, 1013, 1041, 1156, 1449, 1504, 2856, 2930, 3363 cm^{-1}. **^{1}H NMR** (400 MHz, $CDCl_3$) δ 7.37 (s, 1H), 6.34 (s, 1H), 6.15 (s, 1H), 3.53 (s, 2H), 2.08–2.05 (m, 2H), 1.56–1.45 (m, 4H), 1.41–1.29 (m, 4H). **^{13}C NMR** (100.6 MHz, $CDCl_3$) δ 158.4, 141.4, 110.0, 106.8, 70.9, 42.5, 31.6, 26.2, 22.1. **HRMS–DART** (*m/z*): $[M+H]^+$ calcd for $C_{11}H_{17}O_2^+$, 181.1223; found, 181.1225.

1-(Thiophen-2-yl)cyclohexane-1-carbaldehyde (2l)

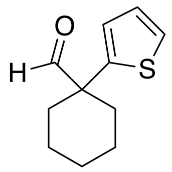

The product **2l** was purified by flash chromatography on silica gel (100:0–95:5, hexane/EtOAc) (Fig. 2, 28.5 mg, 0.147 mmol, 73% isolated yield). Colorless oil. **IR** (neat) 698, 953, 1056, 1240, 1449, 1723, 2699, 2798, 2855, 2932 cm^{-1}. **^{1}H NMR** (400 MHz, $CDCl_3$) δ 9.36 (s, 1H), 7.29 (m, 1H), 7.02 (m, 1H), 6.91 (m, 1H), 2.26–2.22 (m, 2H), 1.96–1.89 (m, 2H), 1.71–1.46 (m, 5H), 1.38 (m, 1H). **^{13}C NMR** (150.9 MHz, $CDCl_3$) δ 199.9, 144.6, 127.4, 125.3, 125.0, 53.0, 32.3, 25.4, 22.5. **HRMS–DART** (*m/z*): $[M+NH_4]^+$ calcd for $C_{11}H_{18}NOS^+$, 212.1104; found, 212.1101.

3-(4-Methoxyphenyl)-3-methylbutan-2-one (2m)

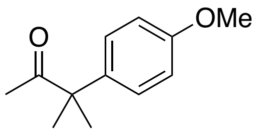

The reaction was carried out with **PTH1** (10 mol %), $LiBF_4$ (10 mol %) and MeCN (0.6 mL) instead of EtOAc. The product **2m** was purified by flash chromatography on silica gel (100:0–90:10, hexane/EtOAc) (Fig. 2, 27.0 mg, 0.140 mmol, 70% isolated yield). Yellow oil. **IR** (neat) 1034, 1184, 1251, 1301, 1352, 1464, 1512, 1609, 1705, 2972 cm^{-1}. **^{1}H NMR** (600 MHz, $CDCl_3$) δ 7.19–7.17 (m, 2H), 6.90–6.87 (m, 2H), 3.81 (s, 3H), 1.91 (m, 3H), 1.46 (s, 6H). **^{13}C NMR** (150.9 MHz, $CDCl_3$) δ 211.5, 158.4, 136.1, 127.0, 114.0, 55.2, 51.7, 25.3, 25.2. **HRMS–DART** (*m/z*): $[M+NH_4]^+$ calcd for $C_{12}H_{20}NO_2^+$, 210.1489; found, 210.1486.

1-(4-Methoxyphenyl)cyclopentane-1-carbaldehyde (2n)

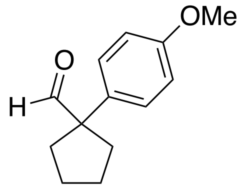

The reaction was carried out with **PTH1** (10 mol %), $LiBF_4$ (10 mol %) and MeCN (0.6 mL) instead of EtOAc. The product **2n** was purified by flash chromatography on silica gel (100:0–95:5, hexane/EtOAc) (Fig. 2, 26.1 mg, 0.128 mmol, 64% isolated yield). Colorless oil. **IR** (neat) 540, 826, 1033, 1181, 1248, 1509, 1608, 1717, 2870, 2952 cm^{-1}. **^{1}H NMR** (400 MHz, $CDCl_3$) δ 9.36 (s, 1H),

7.17 (d, J = 8.8 Hz, 2H), 6.89 (d, J = 8.8 Hz, 2H), 3.80 (s, 3H), 2.52–2.47 (m, 2H), 1.88–1.59 (m, 6H). **^{13}C NMR** (100.6 MHz, $CDCl_3$) δ 200.7, 158.7, 132.2, 128.7, 114.1, 62.9, 55.3, 32.4, 24.2. **HRMS–DART** (*m/z*): $[M+NH_4]^+$ calcd for $C_{13}H_{20}NO_2^+$, 222.1489; found, 222.1489.

1-(4-Methoxyphenyl)cycloheptane-1-carbaldehyde (2o)

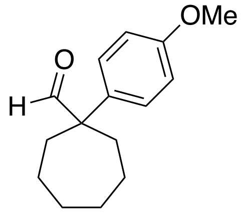

The reaction was carried out with **PTH1** (10 mol %), $LiBF_4$ (10 mol %) and MeCN (0.6 mL) instead of EtOAc. The product **2o** was purified by flash chromatography on silica gel (100:0–95:5, hexane/EtOAc) (Fig. 2, 33.2 mg, 0.143 mmol, 71% isolated yield). Yellow oil. **IR** (neat) 1034, 1185, 1252, 1463, 1511, 1608, 1719, 2702, 2855, 2922 cm^{-1}. **^{1}H NMR** (400 MHz, $CDCl_3$) δ 9.30 (s, 1H), 7.21–7.17 (m, 2H), 6.92–6.88 (m, 2H), 3.80 (s, 3H), 2.23–2.17 (m, 2H), 2.08–2.02 (m, 2H), 1.61–1.53 (m, 8H). **^{13}C NMR** (100.6 MHz, $CDCl_3$) δ 201.6, 158.5, 132.8, 128.5, 114.2, 57.0, 55.2, 33.0, 29.9, 23.0. **HRMS–DART** (*m/z*): $[M+NH_4]^+$ calcd for $C_{15}H_{24}NO_2^+$, 250.1802; found, 250.1803.

4-(4-methoxyphenyl)tetrahydro-2H-pyran-4-carbaldehyde (2p)

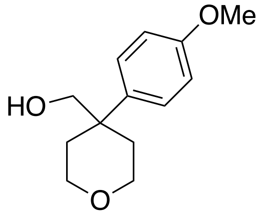

The reaction was carried out with **PTH1** (10 mol %), $LiBF_4$ (10 mol %) and MeCN (0.6 mL) instead of EtOAc. The product **2p** was purified by flash chromatography on silica gel (100:0–80:20, hexane/EtOAc) (Fig. 2, 21.2 mg, 0.096 mmol, 48% isolated yield). Yellow solid. **M.p.** 58–59 °C. **IR** (neat) 1107, 1185, 1253, 1301, 1441, 1511, 1606, 1718, 2836, 2955. cm^{-1}. **^{1}H NMR** (400 MHz, $CDCl_3$) δ 9.36 (s, 1H), 7.22–7.18 (m, 2H), 6.95–6.91 (m, 2H), 3.90 (dt, J = 11.6, 4.0 Hz, 2H), 3.81 (s, 3H), 3.58 (td, J = 11.6, 2.4 Hz, 2H), 2.38–2.35 (m, 2H), 2.09–2.01 (m, 2H). **^{13}C NMR** (100.6 MHz, $CDCl_3$) δ 200.7, 159.0, 130.2, 128.0, 114.5, 64.9, 55.3, 51.4, 31.1. **HRMS–DART** (*m/z*): $[M+NH_4]^+$ calcd for $C_{13}H_{20}NO_3^+$, 238.1438; found, 238.1441.

4-(4-Methoxyphenyl)-1-tosylpiperidine-4-carbaldehyde (2q)

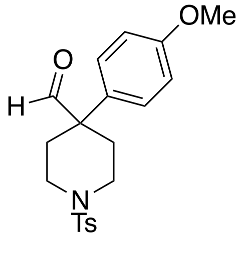

The product **2q** was purified by flash chromatography on silica gel (90:10–75:25, hexane/EtOAc) (Fig. 2, 56.7 mg, 0.152 mmol, 76% isolated yield). Pale yellow solid. **M.p.** 128–130 °C **IR** (neat) 719, 1092, 1162, 1257, 1342, 1512, 1606, 1718, 2838, 2927 cm^{-1}. **^{1}H NMR** (600 MHz, $CDCl_3$) δ 9.19 (s, 1H), 7.61 (d, J = 8.4 Hz, 2H), 7.31 (d, J = 8.4 Hz, 2H), 7.15–7.12 (m, 2H), 6.91–6.89 (m, 2H), 3.79 (s, 3H), 3.67–3.65 (m, 2H), 2.54–2.43 (m, 7H), 2.12–2.07 (m, 2H). **^{13}C NMR** (150.9 MHz, $CDCl_3$) δ 200.3, 159.2, 143.7, 132.9, 129.7, 129.3, 127.9, 127.6, 114.6, 55.3, 51.5, 43.5, 30.3, 21.5. **HRMS–DART** (*m/z*): $[M+NH_4]^+$ calcd for $C_{20}H_{27}N_2O_4S^+$, 391.1686; found, 391.1684.

2-(4-Methoxyphenyl)-2-methylbutanal (2r)

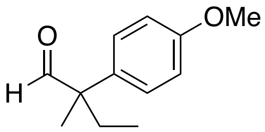

The product **2r** was purified by flash chromatography on silica gel (100:0–95:5, hexane/EtOAc) (Fig. 2, 31.9 mg, 0.166 mmol, 83% isolated yield). The spectrum data of product **2r** was consistent with the literature.[8]

2-(4-Methoxyphenyl)undecanal (2s)

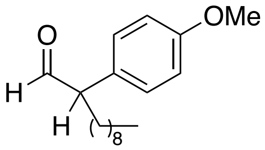

The reaction was carried out with **PTH1** (10 mol %) and $LiBF_4$ (10 mol %). The product **2s** was purified by flash chromatography on silica gel (100:0–97:3, hexane/EtOAc) (Fig. 2, 13.9 mg, 0.050 mmol, 25% isolated yield). Pale yellow oil. **IR** (neat) 546, 828, 1035, 1178, 1249, 1464, 1511, 1722, 2853, 2923 cm^{-1}. **^{1}H NMR** (400 MHz, $CDCl_3$) δ 9.62 (d, J = 2.0 Hz, 1H), 7.12–7.09 (m, 2H), 6.92–6.89 (m, 2H), 3.80 (s, 3H), 3.43 (ddd, J = 8.4, 6.4, 2.0 Hz, 1H), 2.02 (m, 1H), 1.69 (m, 1H), 1.26–1.23 (m, 14H), 0.87 (t, J = 6.8 Hz, 3H). **^{13}C NMR** (100.6 MHz, $CDCl_3$) δ 201.1, 159.0, 129.8, 128.3, 114.4, 58.3, 55.2, 31.8, 29.6, 29.5, 29.4, 29.4, 29.2, 27.0, 22.6, 14.1. **HRMS–DART** (*m/z*): $[M+NH_4]^+$ calcd for $C_{18}H_{32}NO_2^+$, 294.2428; found, 294.2430.

2-(4-Methoxyphenyl)-2-phenylpropan-1-ol (2t)

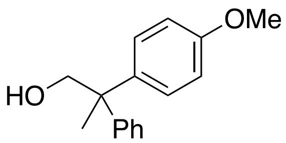

The rearrangement product was isolated as the alcohol **2t** through reduction by $NaBH_4$ (0.4 mmol) in MeOH (5.0 mL). The product **2t** was purified by flash chromatography on silica gel (95:5–80:20, hexane/EtOAc) (Fig. 2, 43.9 mg, 0.181 mmol, 91% isolated yield). The spectrum data of product **2t** was consistent with the literature.[9]

2-(4-Methoxyphenyl)-2-(*p*-tolyl)ethan-1-ol (2u)

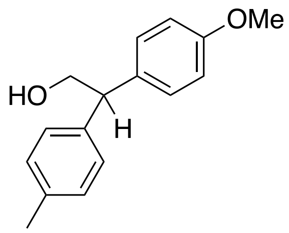

The rearrangement product was isolated as the alcohol **2u** through reduction by $NaBH_4$ (0.4 mmol) in MeOH (5.0 mL). The product **2u** was purified by flash chromatography on silica gel (95:5–80:20, hexane/EtOAc) (Fig. 2, 41.9 mg, 0.173 mmol, 86% isolated yield). Yellow oil. **IR** (neat) 1032, 1061, 1178, 1247, 1302, 1510, 1610, 2835, 2930, 3371 cm^{-1}. **^{1}H NMR** (400 MHz, $CDCl_3$) δ 7.19–7.15 (m, 2H), 7.14–7.11 (m, 4H), 6.87–6.83 (m, 2H), 4.12–4.11 (m, 3H), 3.78 (s, 3H), 2.31 (s, 3H). **^{13}C NMR** (100.6 MHz, $CDCl_3$) δ 158.4, 138.6, 136.3, 133.6, 129.4, 129.2, 128.1, 114.1, 66.3, 55.2, 52.4, 21.0. **HRMS–DART** (*m/z*): $[M+NH_4]^+$ calcd for $C_{16}H_{22}NO_2^+$, 260.1645; found, 260.1643.

***tert*-Butyl 3,3-dimethyl-4-oxoazepane-1-carboxylate (2v)**

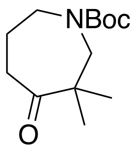

The product **2v** was purified by flash chromatography on silica gel (100:0–80:20, hexane/EtOAc) (Fig. 2, 31.4 mg, 0.130 mmol, 65% isolated yield). Pale yellow oil. **IR** (neat) 934, 1090, 1160, 1278 1366, 1414, 1465, 1691, 2932, 2974 cm^{-1}. Signals for two rotamers were given: **^{1}H NMR** (400 MHz, $CDCl_3$) δ 3.57–3.52 (m, 2H), 3.45 (s, 2H), 2.64 (t, J = 5.6 Hz, 2H), 1.77–1.75 (m, 2H), 1.45 (s, 9H), 1.11 (s, 6H). Signals for both rotamers were given: **^{13}C NMR** (100.6 MHz, $CDCl_3$) δ 215.7, 215.4, 155.3, 155.1, 80.1, 79.9, 55.5, 54.8, 49.7, 49.1, 39.8, 28.4, 28.3, 26.0, 23.7 (Only observed peaks). **HRMS–DART** (*m/z*): $[M+H]^+$ calcd for $C_{13}H_{24}NO_3^+$, 242.1751; found, 242.1751.

***tert*-Butyl 3-Ethyl-3-methyl-4-oxoazepane-1-carboxylate (2w)**

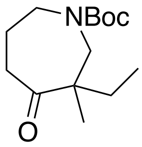

The product **2w** was purified by flash chromatography on silica gel (100:0–90:10, hexane/EtOAc) (Fig. 2, 32.5 mg, 0.127 mmol, 64% isolated yield). Yellow oil. **IR** (neat) 940, 1089, 1158, 1252, 1273, 1365, 1413, 1464, 1688, 2973 cm^{-1}. Signals for two rotamers were given: **^{1}H NMR** (400 MHz, $CDCl_3$) δ 4.05–3.66 (m, 2H), 3.25–3.09 (m, 2H), 2.74 (td, J = 11.6, 2.8 Hz, 1H), 2.50 (m, 1H), 1.81 (brs, 1H), 1.72–1.45 (m, 12H), 1.09 (s, 3H), 0.84 (t, J = 7.6 Hz, 3H). Signals for both rotamers were given: **^{13}C NMR** (100.6 MHz, $CDCl_3$) δ 215.4, 215.0, 155.5, 155.2, 80.1, 79.8, 54.5, 53.8, 53.5, 53.3, 49.8, 49.2, 40.0, 29.7, 28.3, 26.1,19.9, 8.4 (Only observed peaks). **HRMS–DART** (*m/z*): $[M+NH_4]^+$ calcd for $C_{14}H_{29}N_2O_3^+$, 273.2173; found, 273.2174.

tert-Butyl 10-Oxo-6-azaspiro[3.6]decane-6-carboxylate (2x)

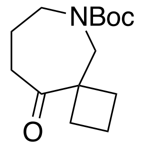

The reaction was carried out for 12 h. The product **2x** was purified by flash chromatography on silica gel (97:3–85:15, hexane/EtOAc) (Fig. 2, 21.8 mg, 0.086 mmol, 43% isolated yield). Colorless oil. **IR** (neat) 935, 1077, 1158, 1283, 1365, 1411, 1688, 2864, 2933, 2974 cm^{-1}. Signals for two rotamers were given: **^{1}H NMR** (400 MHz, $CDCl_3$) δ 3.68–3.66 (m, 2H), 3.49–3.43 (m, 2H), 2.56 (brs, 2H), 2.34 (brs, 2H), 1.99–1.81 (m, 6H), 1.47 (s, 9H). Signals for both rotamers were given: **^{13}C NMR** (100.6 MHz, $CDCl_3$) δ 213.3, 212.6, 155.4, 154.9, 80.2, 79.8, 55.0, 53.4, 52.7, 48.8, 48.6, 39.3, 38.9, 28.4, 28.3, 28.2, 24.8, 24.7, 15.0, 14.9 (Only observed peaks). **HRMS–DART** (*m/z*): $[M+NH_4]^+$ calcd for $C_{14}H_{27}N_2O_3^+$, 271.2016; found, 271.2026.

tert-Butyl 4-oxo-3-(phenylthio)azepane-1-carboxylate (2y)

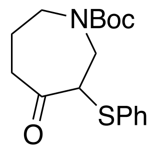

The reaction was carried out with ethyl acetate (600 μl) instead of dichloromethane. The product **2y** was purified by flash chromatography on silica gel (97:3–85:15, hexane/EtOAc) (Fig. 2, 38.1 mg, 0.119 mmol, 59% isolated yield). Pale yellow oil. **IR** (neat) 892, 1091, 1145, 1163, 1239, 1366, 1411, 1466, 1694, 2974 cm^{-1}. Signals for two rotamers (55:45) were given: **^{1}H NMR** (400 MHz, $CDCl_3$) δ 7.45–7.43 (m, 2H), 7.32–7.27 (m, 3H), 4.42 (dd, J = 14.4, 6.4 Hz, 0.45 × 1H), 4.26–4.17 (m, 0.55 × 2H), 4.08–4.03 (m, 0.45 × 2H), 3.89 (m, 0.55 × 1H), 3.01 (m, 0.55 × 1H), 2.89–2.76 (m, 0.45 × 1H + 2H), 2.44 (m, 1H), 1.80–1.70 (m, 2H), 1.43–1.42 (m, 9H). Signals for both rotamers were given: **^{13}C NMR** (100.6 MHz, $CDCl_3$) δ 207.0, 206.9, 154.2, 154.0, 132.4, 132.4, 129.2, 129.1, 128.1, 128.0, 80.6, 80.4, 55.5, 54.5, 50.3, 49.3, 48.5, 48.2, 39.0, 38.8, 28.3, 27.4, 26.6 (Only observed peaks). **HRMS–DART** (*m/z*): $[M+NH_4]^+$ calcd for $C_{17}H_{27}N_2O_3S^+$, 339.1737; found, 339.1737.

11,11-Dimethyldibenzo[_b,f_]oxepin-10(11H)-one (2z)

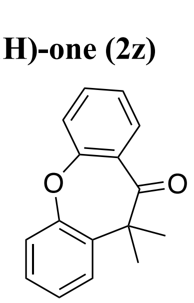

The reaction was carried out with MeCN (600 μl) instead of dichloromethane. The product **2z** was purified by flash chromatography on silica gel (100:0–95:5, hexane/EtOAc) (Fig. 2, 32.9 mg, 0.138 mmol, 69% isolated yield). White solid. **M.p.** 98–100 °C **IR** (neat) 759, 887, 1104, 1231, 1289, 1444, 1472, 1599, 1676, 2982 cm^{-1}. **^{1}H NMR** (400 MHz, $CDCl_3$) δ 8.06 (dd, J = 8.0, 1.6 Hz, 1H), 7.54 (m, 1H), 7.40 (m, 1H), 7.34 (m, 1H), 7.28–7.16 (m, 4H), 1.67 (s, 6H). **^{13}C NMR** (100.6 MHz, $CDCl_3$) δ

195.3, 157.8, 156.4, 134.3, 132.6, 131.5, 128.6, 127.2, 125.9, 124.7, 123.3, 121.6, 120.8, 51.0, 23.3. **HRMS–DART** (*m/z*): $[M+H]^+$ calcd for $C_{16}H_{15}O_2^+$, 239.1067; found, 239.1068.

2,2-Dimethylcyclotridecan-1-one (2A)

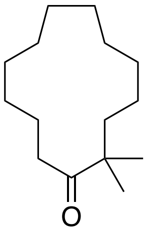

The reaction was carried out with MeCN (600 μl) instead of dichloromethane. The product **2A** was purified by flash chromatography on silica gel (100:0–95:5 hexane/EtOAc) (Fig. 2, 30.5 mg, 0.136 mmol, 68% isolated yield). Colorless crystal. **M.p.** 34–35 °C **IR** (neat) 729, 1022, 1107, 1365, 1386, 1407, 1461, 1702, 2860, 2928 cm^{-1}. **^{1}H NMR** (400 MHz, $CDCl_3$) δ 2.54–2.51 (m, 2H), 1.66 (quintet, J = 6.0 Hz, 2H), 1.54–1.49 (m, 2H), 1.32–1.26 (m, 14H), 1.18–1.12 (m, 8H). **^{13}C NMR** (100.6 MHz, $CDCl_3$) δ 216.1, 47.8, 40.8, 35.7, 26.9, 26.6, 26.6, 25.4, 25.2, 24.7, 24.5, 24.4, 22.2, 21.8. **HRMS–DART** (*m/z*): $[M+NH_4]^+$ calcd for $C_{15}H_{32}NO^+$, 242.2478; found, 242.2480.

8-Tosyl-2-oxa-8-azaspiro[4.5]decan-4-one (2B)

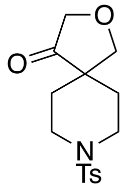

The product **2B** was purified by flash chromatography on silica gel (90:10–70:30, hexane/EtOAc) (Fig. 2, 39.5 mg, 0.128 mmol, 64% isolated yield). Pale yellow solid. **M.p.** 118–120 °C **IR** (neat) 549, 919, 1065, 1160, 1323, 1342, 1597, 1751, 2852, 2924 cm^{-1}. **^{1}H NMR** (400 MHz, $CDCl_3$) δ 7.64 (d, J = 8.0 Hz, 2H), 7.34 (d, J = 8.0 Hz, 2H), 3.97 (s, 2H), 3.90 (s, 2H), 3.41–3.36 (m, 2H), 2.88–2.82 (m, 2H), 2.45 (s, 3H), 1.92–1.85 (m, 2H), 1.70–1.64 (m, 2H). **^{13}C NMR** (100.6 MHz, $CDCl_3$) δ 216.7, 143.7, 133.2, 129.8, 127.6, 76.6, 71.1, 45.5, 42.5, 29.5, 21.6. **HRMS–DART** (*m/z*): $[M+H]^+$ calcd for $C_{15}H_{20}NO_4S^+$, 310.1108; found, 310.1111.

8-Tosyl-8-azaspiro[4.5]decan-1-one (2C)

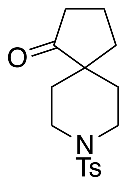

The reaction was carried out with ethyl acetate (600 μl) instead of dichloromethane. The product **2C** was purified by flash chromatography on silica gel (100:0–90:10, toluene/EtOAc) (Fig. 2, 25.2 mg, 0.082 mmol, 41% isolated yield). Colorless oil. **IR** (neat) 724, 817, 931, 1053, 1093, 1162, 1341, 1597, 1732, 2945 cm^{-1}. **^{1}H NMR** (400 MHz, $CDCl_3$) δ 7.64 (d, J = 8.0 Hz, 2H), 7.32 (d, J = 8.0 Hz, 2H), 3.40–3.35 (m, 2H), 2.86–2.80 (m, 2H), 2.44 (s, 3H), 2.25 (t, J = 7.6 Hz, 2H), 1.89–1.73 (m, 6H), 1.51–1.45 (m, 2H). **^{13}C NMR** (100.6 MHz, $CDCl_3$) δ 221.2, 143.5, 133.4, 129.6, 127.6, 46.0, 42.4,

37.6, 34.8, 31.4, 21.5, 18.5. **HRMS–DART** (*m/z*): $[M+H]^+$ calcd for $C_{16}H_{22}NO_3S^+$, 308.1315; found, 308.1320.

***tert*-Butyl 4-Oxo-2-azaspiro[4.5]decane-2-carboxylate (2D)**

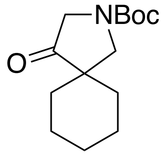

The product **2D** was purified by flash chromatography on silica gel (100:0–92:8 hexane/EtOAc) (Fig. 2, 32.8 mg, 0.129 mmol, 65% isolated yield). Yellow crystal. **M.p.** 63–66 °C **IR** (neat) 772, 883, 1110, 1164, 1401, 1452, 1697, 1751, 2857, 2930 cm^{-1}. Signals for two rotamers were given: ^{1}**H NMR** (400 MHz, $CDCl_3$) δ 3.82 (brs, 2H), 3.62–3.59 (m, 2H), 1.74–1.72 (m, 2H), 1.65 (brs, 1H), 1.57–1.49 (m, 13H), 1.37–1.26 (m, 3H). Signals for both rotamers were given: ^{13}**C NMR** (100.6 MHz, $CDCl_3$) δ 215.4, 214.8, 154.6, 80.2, 53.7, 53.1, 52.5, 52.2, 50.4, 49.9, 31.3, 28.4, 25.1, 22.1 (Only observed peaks). **HRMS–DART** (*m/z*): $[M+NH_4]^+$ calcd for $C_{14}H_{27}N_2O_3^+$, 271.2016; found, 271.2017.

***tert*-Butyl (2-Oxocyclopentyl)carbamate (2E)**

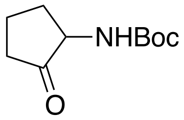

The product **2E** was purified by flash chromatography on silica gel (95:5–80:20, hexane/EtOAc) (Fig. 2, 31.5 mg, 0.158 mmol, 79% isolated yield). Pale brown solid. **M.p.** 74–76 °C. **IR** (neat) 1056, 1160, 1248, 1282, 1366, 1508, 1688, 1751, 2974, 3341 cm^{-1}. ^{1}**H NMR** (400 MHz, $CDCl_3$) δ 4.99 (brs, 1H), 3.92 (brs, 1H), 2.59 (brs, 1H), 2.40 (m, 1H), 2.21–2.01 (m, 2H), 1.89–1.58 (m, 2H), 1.44 (s, 9H). ^{13}**C NMR** (150.9 MHz, $CDCl_3$) δ 215.1, 155.6, 79.8, 59.0, 34.8, 30.4, 28.3, 17.8. **HRMS–DART** (*m/z*): $[M + H]^+$ calcd for $C_{10}H_{18}NO_3^+$, 200.1281; found, 200.1282.

***cis*-4-(Phenylthio)tetrahydrofuran-3-ol (2F)**

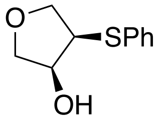

The rearrangement product was isolated as the alcohol **2F** through reduction by $NaBH_4$ (0.4 mmol) in MeOH (5.0 mL). The relative cofiguration and ratio (*cis*/*trans* >99:1) was deretemined by ^{1}H-NMR analysis.[10] The product **2F** was purified by flash chromatography on silica gel (85:15–65:35, hexane/EtOAc) (Fig. 2, 18.4 mg, 0.094 mmol, 47% isolated yield). Pale yellow crystal. **M.p.** 55–58 °C. **IR** (neat) 740, 895, 1051, 1309, 1438, 1480, 1583, 2871, 2946, 3395 cm^{-1}. ^{1}**H NMR** (400 MHz, $CDCl_3$) δ 7.45–7.42 (m, 2H), 7.35–7.25 (m, 3H), 4.31 (m, 1H), 4.20 (t, J = 6.8 Hz, 1H), 4.01–3.92 (m, 2H), 3.76–3.67 (m, 2H), 2.78 (m, 1H). ^{13}**C NMR** (100.6 MHz, $CDCl_3$) δ 133.7, 131.1, 129.3, 127.5, 74.7, 70.6, 70.0, 53.3. **HRMS–DART** (*m/z*): $[M+NH_4]^+$ calcd for $C_{10}H_{16}NO_2S^+$, 214.0896; found, 214.0895.

2-(4-Methoxyphenyl)cyclohexan-1-one (2G)

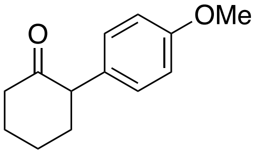

The reaction was carried out with ethyl acetate (600 μl) instead of dichloromethane. The product **2G** was purified by flash chromatography on silica gel (Biotage Selekt, 98:2–90:20, hexane/EtOAc) (Fig. 2; 32.9 mg, 0.161 mmol, 81% isolated yield). The spectrum data of product **2G** was consistent with the literature.[11]

5-(4-Methoxyphenyl)-2,2-dimethylcycloheptan-1-one (2H)

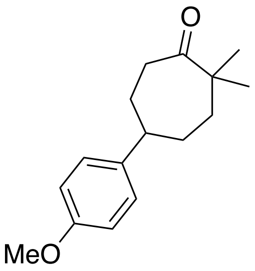

The product **2H** was purified by flash chromatography on silica gel (Biotage Selekt, 100:0–94:6, hexane/EtOAc) (Fig. 2, 23.0 mg, 0.093 mmol, 47% isolated yield). Yellow oil. **IR** (neat) 828, 940, 1036, 1178, 1245, 1459, 1512, 1700, 2860, 2926 cm^{-1}. **^{1}H NMR** (400 MHz, $CDCl_3$) δ 7.09–7.05 (m, 2H), 6.84–6.80 (m, 2H), 3.78 (s, 3H), 2.93 (ddd, J = 12.8, 10.8, 2.0 Hz, 1H), 2.67 (tt, J = 12.0, 3.2 Hz, 1H), 2.39 (ddd, J = 11.2, 7.2, 2.0 Hz, 1H), 2.04 (m, 1H), 1.90–1.75 (m, 3H), 1.63–1.44 (m, 2H), 1.15 (s, 3H), 1.13 (s, 3H). **^{13}C NMR** (100.6 MHz, $CDCl_3$) δ 218.0, 157.9, 139.2, 127.5, 113.8, 55.2, 48.0, 47.8, 39.2, 39.0, 34.4, 32.8, 27.7, 23.4. **HRMS–DART** (*m/z*): $[M+NH_4]^+$ calcd for $C_{15}H_{24}NO^+$, 234.1852; found, 234.1851.

6. Characterization Data for Allylic Alcohols

9-(Prop-1-en-2-yl)-9H-xanthen-9-ol (4a)

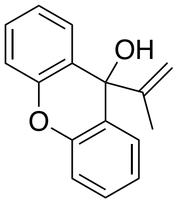

White solid. **M.p.** 97–99 °C. **IR** (neat) 1038, 1241, 1315, 1446, 1476, 1573, 1602, 3038, 3452, 3548 cm^{-1}. **^{1}H NMR** (400 MHz, $CDCl_3$) δ 7.55–7.53 (m, 2H), 7.35–7.30 (m, 2H), 7.16–7.12 (m, 4H), 5.65 (s, 1H), 5.10 (s, 1H), 2.36 (s, 1H), 1.37 (s, 3H). **^{13}C NMR** (100.6 MHz, $CDCl_3$) δ 150.2, 147.9, 129.3, 127.7, 124.4, 123.4, 116.4, 110.5, 71.2, 20.0. **HRMS–DART** (*m/z*): $[M]^+$ calcd for $C_{16}H_{14}O_2^+$, 238.0988; found, 238.0986.

***trans*-4-(4-Methoxyphenyl)-1-(prop-1-en-2-yl)cyclohexan-1-ol (4c)**

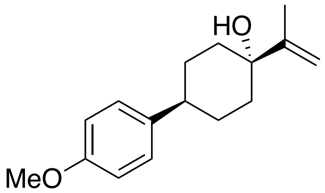

White solid. **M.p.** 69–71 °C. **IR** (neat) 1037, 1128, 1178, 1245, 1441, 1512, 1611, 2856, 2928, 3448 cm^{-1}. **^{1}H NMR** (400 MHz, $CDCl_3$) δ 7.20–7.16 (m, 2H), 6.87–6.83 (m, 2H), 5.06 (m, 1H), 4.83 (m, 1H), 3.79 (s, 3H), 2.45 (m, 1H), 1.96–1.73 (m, 11H). **^{13}C NMR** (100.6 MHz, $CDCl_3$) δ 157.8, 152.4, 139.4, 127.6, 113.7, 108.9, 72.9, 55.2, 42.9, 36.0, 29.5, 19.0. **HRMS–DART** (*m/z*): $[M]^+$ calcd for $C_{16}H_{22}O_2^+$, 246.1614; found, 246.1615.

***cis*-4-(4-Methoxyphenyl)-1-(prop-1-en-2-yl)cyclohexan-1-ol (4c')**

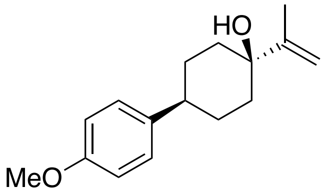

Pale yellow oil. **IR** (neat) 1035, 1054, 1178, 1243, 1450, 1511, 1610, 2861, 2933, 3410 cm^{-1}. **^{1}H NMR** (400 MHz, $CDCl_3$) δ 7.11 (d, J = 7.2 Hz, 2H), 6.82 (d, J = 7.2 Hz, 2H), 5.11 (s, 1H), 5.06 (s, 1H), 3.78 (s, 3H), 2.58 (m, 1H), 2.22 (d, J = 12.8 Hz, 2H), 1.87–1.84 (m, 5H), 1.66–1.47 (m, 4H), 1.41 (s, 1H). **^{13}C NMR** (100.6 MHz, $CDCl_3$) δ 157.7, 146.9, 138.5, 127.6, 113.6, 113.2, 73.4, 55.2, 42.5, 36.3, 31.4, 18.8. **HRMS–DART** (*m/z*): $[M]^+$ calcd for $C_{16}H_{22}O_2^+$, 246.1614; found, 246.1617.

7. General Procedure for Alkylative Semipinacol Type Rearrangement

The reaction to produce 6aa in Fig. 3 is representative. In a glovebox, to an oven-dried vial with a stirring bar was added **PTH1** (6.5 mg, 0.02 mmol), sodium perchlorate monohydrate (2.8 mg, 0.02 mmol), allylic alcohol **4a** (47.7 mg, 0.2 mmol), diethyl bromomalonate **5a** (50.5 µL, 0.3 mmol), 2, 6-lutidine (46.6 µL, 0.4 mmol), dichloromethane (562.5 µL) and H_2O (37.5 µL). After sealing the vial with a cap and removed from the glove box, the reaction was stirred and irradiated with a 34W blue LED (0.5 cm away) with a cooling fan to keep the temperature around 40 °C (Supplementary Figure 2). After 20 h, the reaction was quenched by sat. NH_4Cl aq. solution (1.5 mL). The aqueous layer was thrice extracted with dichloromethane (500 µL), dried over Na_2SO_4 and then, filtered. After volatiles were removed under reduced pressure, purification by flash column chromatography on silica gel (Biotage Selekt, 99:1–90:10, hexane/EtOAc) gave **6aa** (66.5 mg, 0.168 mmol) in 84% isolated yield.

8. Characterization Data for Alkylative Semipinacol Type Rearrangement Products

Diethyl 2-[(10-Methyl-11-oxo-10,11-dihydrodibenzo[*b,f*]oxepin-10-yl)methyl]malonate (6aa)

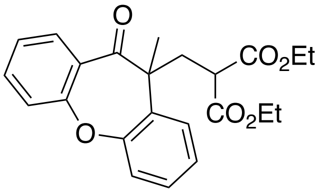

The product **6aa** was purified by flash chromatography on silica gel (Biotage Selekt, 99:1–90:10, hexane/EtOAc) (Fig. 3, 66.5 mg, 0.168 mmol, 84% isolated yield). Pale yellow oil. **IR** (neat) 762, 1028, 1148, 1227, 1277, 1442, 1472, 1674, 1729, 2983 cm^{-1}. **^{1}H NMR** (400 MHz, $CDCl_3$) δ 8.03 (dd, J = 7.6, 1.2 Hz, 1H), 7.55 (t, J = 7.6 Hz, 1H), 7.38–7.35 (m, 2H), 7.29–7.17 (m, 4H), 4.07 (q, J = 7.2 Hz, 2H), 4.00–3.88 (m, 2H), 3.24 (t, J = 6.0 Hz, 1H), 3.01 (dd, J = 14.4, 6.0 Hz, 1H), 2.57 (dd, J = 14.4, 6.0 Hz, 1H), 1.68 (s, 3H), 1.17 (t, J = 7.2 Hz, 6H). **^{13}C NMR** (100.6 MHz, $CDCl_3$) δ 193.8, 169.2, 169.0, 157.6, 156.5, 134.6, 131.6, 129.8, 129.1, 128.1, 126.0, 124.6, 123.5, 121.8, 120.8, 61.5, 61.5, 54.3, 48.8, 32.9, 19.9, 13.9, 13.8. **HRMS–DART** (*m/z*): [M+H]$^+$ calcd for $C_{23}H_{25}O_6^+$, 397.1646; found, 397.1644.

3-(10-Methyl-11-oxo-10,11-dihydrodibenzo[*b,f*]oxepin-10-yl)propanenitrile (6ab)

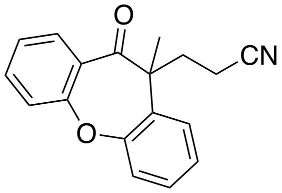

The product **6ab** was purified by flash chromatography on silica gel (Biotage Selekt, 98:2–85:15, hexane/EtOAc) (Fig. 3, 29.3 mg, 0.106 mmol, 53% isolated yield). Colorless oil. **IR** (neat) 759, 887, 1103, 1278, 1288, 1443, 1471, 1673, 2247, 2990 cm^{-1}. **^{1}H NMR** (400 MHz, $CDCl_3$) δ 8.04 (dd, J = 8.8, 1.6 Hz, 1H), 7.59 (m, 1H), 7.38–7.30 (m, 4H), 7.28–7.21 (m, 2H), 2.71 (m, 1H), 2.27–2.17 (m, 3H), 1.73 (s, 3H). **^{13}C NMR** (100.6 MHz, $CDCl_3$) δ 193.3, 157.6, 156.4, 134.9, 131.7, 129.5, 129.3,

127.9, 126.5, 124.4, 123.9, 122.0, 121.0, 119.3, 54.3, 30.8, 19.9, 13.4. **HRMS–DART** (*m/z*): $[M+NH_4]^+$ calcd for $C_{18}H_{19}N_2O_2^+$, 295.1441; found, 295.1441.

Diethyl [1,1-Difluoro-2-(10-methyl-11-oxo-10,11-dihydrodibenzo[*b,f*]oxepin-10-yl)ethyl]phosphonate (6ac)

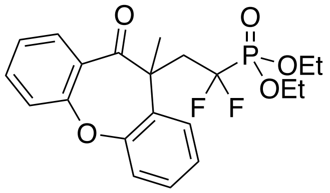

The product **6ac** was purified by flash chromatography on silica gel (Biotage Selekt, 95:5–70:30, hexane/EtOAc) (Fig. 3, 33.1 mg, 0.078 mmol, 39% isolated yield). Pale yellow oil. **IR** (neat) 764, 975, 1022, 1067, 1229, 1277, 1444, 1473, 1681, 2991 cm^{-1}. **^{1}H NMR** (400 MHz, $CDCl_3$) δ 8.00 (dd, J = 8.0, 2.0 Hz, 1H), 7.55 (ddd, J = 8.0, 7.2, 2.0 Hz, 1H), 7.44 (m, 1H), 7.35 (dd, J = 8.0, 0.8 Hz, 1H), 7.30–7.23 (m, 3H), 7.20 (m, 1H), 4.18–3.97 (m, 4H), 3.25 (ddt, J = 38.0, 16.0, 4.0 Hz, 1H), 2.75 (m, 1H), 1.94 (s, 3H), 1.26–1.20 (m, 6H). **^{13}C NMR** (100.6 MHz, $CDCl_3$) δ 192.7, 157.0, 156.2, 134.3, 131.9, 130.1, 129.1, 127.4, 126.4, 124.8, 123.6, 121.8, 120.8 (td, $J_{C–F,\ C–P}$ = 263.2, 216.2 Hz), 120.4, 64.5 (dd, $J_{C–F}$ = 52.0, 6.8 Hz), 52.7 (d, $J_{C–P}$ = 9.4 Hz), 36.9 (td, $J_{C–F,\ C–P}$ = 18.8, 15.1 Hz), 20.5, 16.2 (dd, $J_{C–F}$ = 5.4, 1.8 Hz). **^{19}F NMR** (376 MHz, $CDCl_3$) δ –107.5 (dd, $J_{F–F}$ = 297.0 Hz, $J_{F–P}$ = 109.5 Hz), –110.7 (dd, $J_{F–F}$ = 297.0 Hz, $J_{F–P}$ = 105.0 Hz). **^{31}P NMR** (162 MHz, $CDCl_3$) δ 5.82 (dd, $J_{P–F}$ = 109.5, 105.0 Hz). **HRMS–DART** (*m/z*): $[M+H]^+$ calcd for $C_{21}H_{24}F_2O_5P^+$, 425.1324; found, 425.1323.

11-Methyl-11-(2,2,2-trifluoroethyl)dibenzo[*b,f*]oxepin-10(11H)-one (6ad)

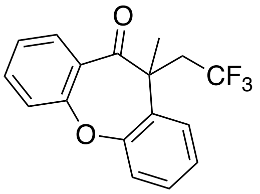

The product **6ad** was purified by flash chromatography on silica gel (Biotage Selekt, 100:0–95:5, hexane/EtOAc) (Fig. 3, 43.2 mg, 0.141 mmol, 71% isolated yield). Colorless oil. **IR** (neat) 1098, 1230, 1258, 1282, 1366, 1444, 1473, 1601, 1681, 3001 cm^{-1}. **^{1}H NMR** (400 MHz, $CDCl_3$) δ 8.20 (dd, J = 8.0, 1.6 Hz, 1H), 7.58 (m, 1H), 7.42–7.36 (m, 2H), 7.31–7.20 (m, 4H), 3.09–2.88 (m, 2H), 1.88 (s, 3H). **^{13}C NMR** (100.6 MHz, $CDCl_3$) δ 192.0, 157.2, 156.4, 134.8, 131.8, 129.4, 129.3, 127.4, 126.5, 126.0 (q, $J_{C–F}$ = 278.9 Hz), 124.4, 123.9, 121.9, 120.8, 51.8 (m), 38.1 (q, $J_{C–F}$ = 27.5 Hz), 19.8 (m). **^{19}F NMR** (376 MHz, $CDCl_3$) δ –59.3. **HRMS–DART** (*m/z*): $[M+NH_4]^+$ calcd for $C_{17}H_{17}F_3NO_2^+$, 324.1206; found, 324.1208.

Diethyl 2-[(1-methyl-2-oxo-1,2,3,4-tetrahydronaphthalen-1-yl)methyl]malonate (6ba)
Diethyl 2-[(2-methyl-1-oxo-1,2,3,4-tetrahydronaphthalen-2-yl)methyl]malonate (6ba')

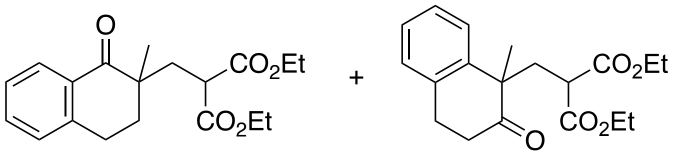

6ba **6ba'**

The product **6ba** and **6ba'** was purified by flash chromatography on silica gel (Biotage Selekt, 100:0–90:10, hexane/EtOAc) (Fig. 3, total 35.9 mg, 0.108 mmol, 54% isolated yield. **6ba**: 25%; **6ba'**: 29% determined by ^{1}H NMR). Pale yellow oil. **IR** (neat) 762, 1028, 1148, 1227, 1277, 1442, 1472, 1674, 1729, 2983 cm^{-1}.

6ba: **^{1}H NMR** (400 MHz, $CDCl_3$) δ 8.01 (dd, J = 7.6, 0.8 Hz, 1H), 7.46 (td, J = 7.6, 1.2 Hz, 1H), 7.32-7.16 (m, 2H), 4.22–4.06 (m, 4H), 3.51 (t, J = 6.4 Hz, 1H), 3.05–2.94 (m, 2H), 2.34 (d, J = 6.4 Hz, 2H), 2.06 (m, 1H), 1.95 (m, 1H), 1.27–1.19 (m, 9H). **^{13}C NMR** (100.6 MHz, $CDCl_3$) δ 201.2, 169.6, 169.2, 140.3, 136.3, 131.4, 128.0, 127.0, 126.7, 61.6, 61.5, 50.6, 44.1, 37.6, 34.3, 28.4, 25.2, 14.0, 13.9.

6ba': **^{1}H NMR** (400 MHz, $CDCl_3$) δ 7.32–7.16 (m, 4H), 4.22–4.06 (m, 2H), 3.95–3.80 (m, 2H), 3.25 (m, 1H), 3.12 (t, J = 6.4 Hz, 1H), 3.00 (m, 1H), 2.72–2.67 (m, 3H), 2.45 (dd, J = 14.4, 6.4 Hz, 1H), 1.46 (s, 3H), 1.27–1.19 (m, 3H), 1.14 (t, J = 7.2 Hz, 3H). **^{13}C NMR** (100.6 MHz, $CDCl_3$) δ 213.3, 169.9, 169.0, 142.9, 133.2, 128.6, 128.2, 126.9, 126.8, 61.5, 61.4, 48.9, 48.1, 37.3, 35.0, 27.3, 21.9, 13.9, 13.8.

HRMS–DART (*m/z*): $[M+H]^+$ calcd for $C_{19}H_{25}O_5^+$, 333.1697; found, 333.1695.

5-(4-Methoxyphenyl)-2-methyl-2-(2,2,2-trifluoroethyl)cycloheptan-1-one (6cd)

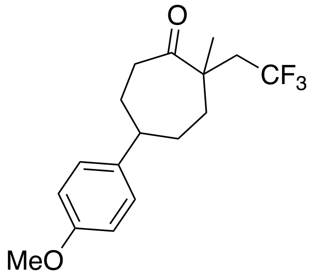

Only **4c** was used as substrate.The product **6cd** was purified by flash chromatography on silica gel (Biotage Selekt, 100:0–90:10, hexane/EtOAc) (Fig. 3, 21.6 mg, 0.069 mmol, 34% isolated yield). The diastereomeric ratio is 59:41determined by ^{1}H NMR. Colorless oil. **IR** (neat) 829, 1034, 1131, 1178, 1249, 1374, 1513, 1611, 1704, 2937 cm^{-1}. **^{1}H NMR** (400 MHz, $CDCl_3$) δ 7.08–7.04 (m, 2H), 6.84–6.81 (m, 2H), 3.78 (s, 3H), 2.99 (m, 0.59 × 1H), 2.88 (td, J = 12.0, 2.4 Hz, 0.41 × 1H), 2.78–2.65 (m, 1H + 0.59 × 1H), 2.56–2.24 (m, 3H), 2.12–2.01 (m, 1H + 0.41 × 1H), 1.96–1.77 (m, 2H), 1.72–1.35 (m, 2H), 1.27 (s, 0.41 × 3H), 1.26 (s, 0.59 × 3H). Signals for both diastereomers were given: **^{13}C NMR** (100.6 MHz, $CDCl_3$) δ 214.7, 214.6, 158.1, 158.1, 138.7, 138.5, 127.5 (×2C), 127.3 (q, J_{C-F} = 278.0 Hz), 126.0 (q, J_{C-F} = 278.9 Hz), 113.9 (×2C), 55.2 (×2C), 48.8, 48.1, 47.9, 47.8, 43.9 (q, J_{C-F} = 27.4 Hz), 40.0, 38.1, 36.8 (q, J_{C-F} = 27.1 Hz), 36.4, 34.5, 34.3, 33.8, 32.3, 32.0, 24.2 (m), 22.5.

^{19}F NMR (376 MHz, $CDCl_3$) δ –58.9, –59.8. **HRMS–DART** (*m/z*): $[M+NH_4]^+$ calcd for $C_{17}H_{25}F_3NO_2^+$, 332.1832; found, 332.1832.

Diethyl 2-[(1-Methyl-2-oxocyclotridecyl)methyl]malonate (6da)

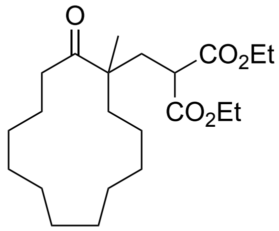

The reaction was carried out with hexafluoroisopropanol (37.5 μl) instead of water. The product **6da** was purified by flash chromatography on silica gel (Biotage Selekt, 100:0–95:5, hexane/EtOAc) (Fig. 3, 31.0 mg, 0.081 mmol, 41% isolated yield). Colorless oil. **IR** (neat) 857, 1031, 1147, 1368, 1463, 1700, 1730, 1751, 2860, 2931 cm^{-1}. **^{1}H NMR** (400 MHz, $CDCl_3$) δ 4.22–4.13 (m, 4H), 3.23 (t, *J* = 6.4 Hz, 1H), 2.60 (m, 1H), 2.50–2.41 (m, 2H), 2.02 (dd, *J* = 14.4, 6.4 Hz, 1H), 1.74 (m, 1H), 1.65–1.39 (m, 4H), 1.36–1.24 (m, 21H), 1.11 (s, 3H). **^{13}C NMR** (100.6 MHz, $CDCl_3$) δ 214.5, 170.0, 169.7, 61.5, 61.5, 50.6, 48.6, 40.1, 36.3 (× 2C), 26.8, 26.6, 26.3, 25.3, 25.0, 24.3, 24.2, 21.7, 21.5, 20.1, 14.0, 13.9. **HRMS–DART** (*m/z*): $[M+ H]^+$ calcd for $C_{22}H_{39}O_5^+$, 383.2792; found, 383.2793.

2-Methyl-2-(2,2,2-trifluoroethyl)cyclotridecan-1-one (6dd)

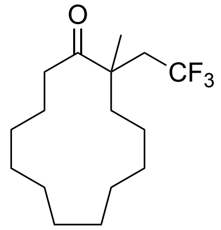

The reaction was carried out with $TBABF_4$ (0.02 mmol) and MeCN (562.5 μL) instead of $NaClO_4{\cdot}H_2O$ and DCM, respectively. The product **6dd** was purified by flash chromatography on silica gel (Biotage Selekt, 100:0–95:5, hexane/EtOAc) (Fig. 3, 32.7 mg, 0.112 mmol, 56% isolated yield). White Solid. **M.p.** 62–63 °C. **IR** (neat) 1065, 1122, 1167, 1259, 1378, 1410, 1459, 1697, 2857, 2928 cm^{-1}. **^{1}H NMR** (400 MHz, $CDCl_3$) δ 2.90–2.70 (m, 2H), 2.38 (ddd, *J* = 18.6, 6.4, 3.2 Hz, 1H)., 2.05 (dq, *J* = 15.2, 11.2 Hz, 1H), 1.94 (m, 1H), 1.59–1.41 (m, 3H), 1.36–1.11 (m, 19H). **^{13}C NMR** (100.6 MHz, $CDCl_3$) δ 212.9, 126.6 (q, $J_{C–F}$ = 278.2 Hz), 48.1, 40.8 (q, $J_{C–F}$ = 27.2 Hz), 40.4, 36.3, 26.6, 26.2, 26.2, 25.2, 24.7, 23.8, 23.4, 21.7, 21.1, 20.5. **^{19}F NMR** (376 MHz, $CDCl_3$) δ –59.5. **HRMS–DART** (*m/z*): $[M+NH_4]^+$ calcd for $C_{16}H_{31}F_3NO^+$, 310.2352; found, 310.2352.

9. Effects of Reaction Components on Alkylative Semipinacol Type Rearrangement

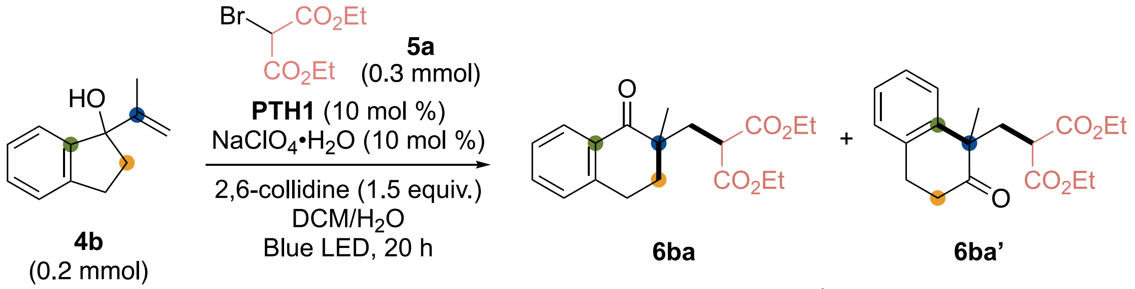

entry	change from standard conditions	^{1}H-NMR yield (isolated) 6ba	6ba'
1	none	14%	16%
2	2,6-lutidine (2.0 equiv.)	27% (25%)	33% (29%)
3	2,6-lutidine (3.0 equiv.)	26%	30%
4	$LiBF_4$ instead of $NaClO_4$•H_2O	14%	16%
5	Bu_4NBF_4 instead of $NaClO_4$•H_2O	24%	29%
6	DCM (600 μL) instead of DCM/H_2O	25%	26%

Supplementary Figure 3. Effects of Reaction Components.

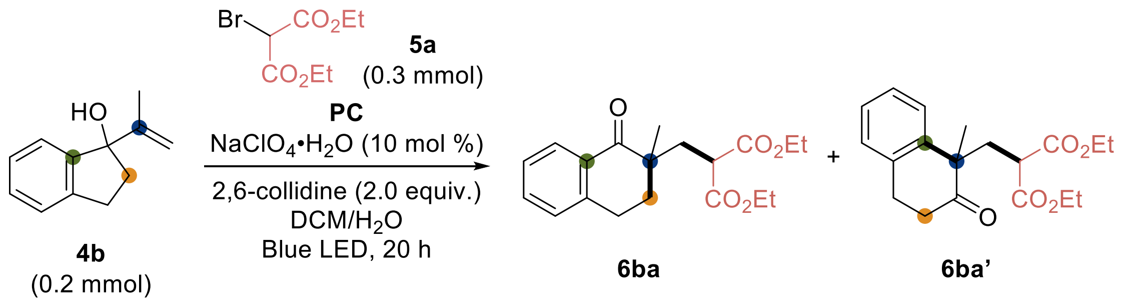

PTH (10 mol%)

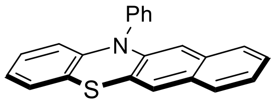

PTH1
6ba: 27%, 6ba': 33%

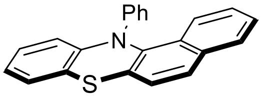

PTH5
6ba: 14%, **6ba'**: 16%

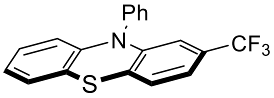

PTH7
6ba: 14%, **6ba'**: 16%

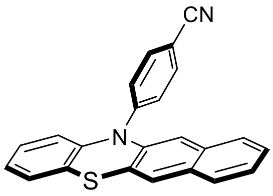

PTH8
6ba: 23%, **6ba'**: 23%

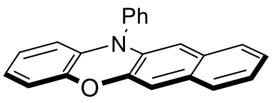

POX2
6ba: 18%, **6ba'**: 21%

PC (2 mol%)

$Ir(ppy)_3$	$[Ru(bpy)_3](PF_6)_2$	$[Ir[dF(CF_3)ppy]_2(dtbbpy)]PF_6$	4CzIPN
**6ba**: 29%	**6ba**: 7%	**6ba**: 8%	**6ba**: 7%
**6ba'**: 32%	**6ba'**: 7%	**6ba'**: n.d.	**6ba'**: 11%

Supplementary Figure 4. Effects of Photoredox Catalysts.

10. UV-Vis Absorption Spectra of Reaction Mixtures

UV-Vis spectra of mixed solution (Diethyl Bromomalonate **5a** : **PTH1** : $NaClO_4 \cdot H_2O$ = 1:1:1) were measured with 40 μM DCM solution (Supplementary Figure 4).

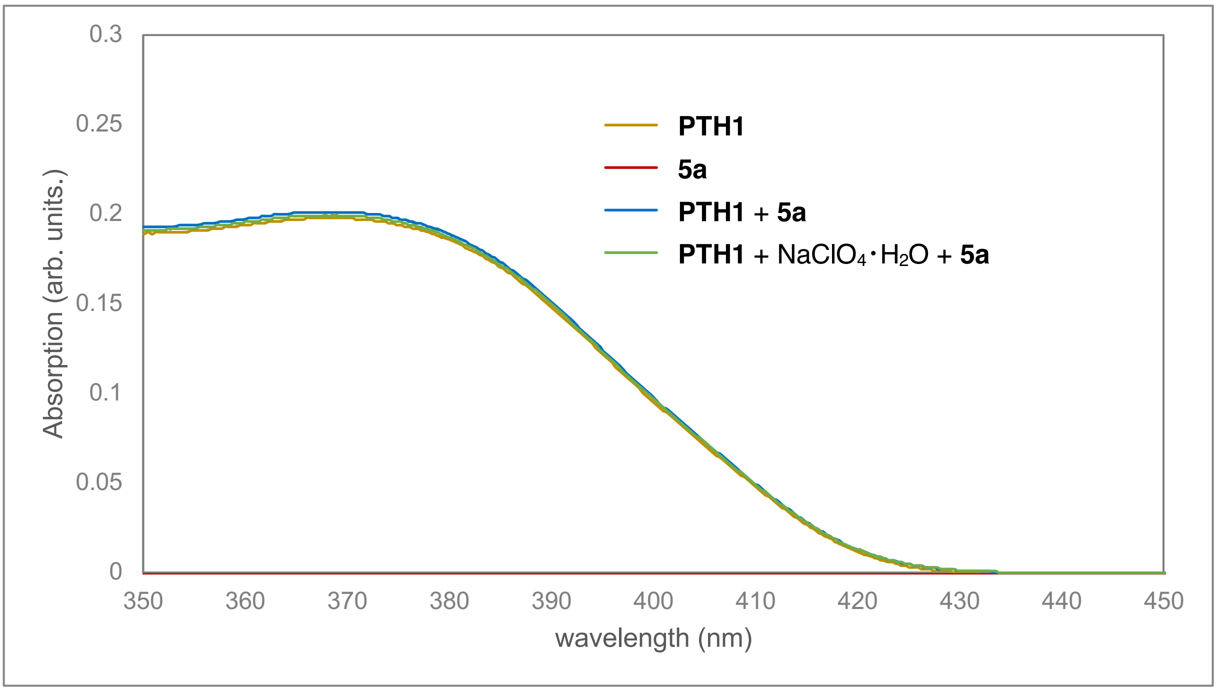

Supplementary Figure 5. UV-Vis absorption spectra of the reaction mixture (40 μM DCM solution).

UV-Vis spectra of the reation mixture were measured under identical concentration to the catalytic reaction conditions [Diethyl Bromomalonate **5a** (0.3 mmol), **PTH1** (10 mol%) and $NaClO_4 \cdot H_2O$ (10 mol%) in DCM (600 μL) solution].

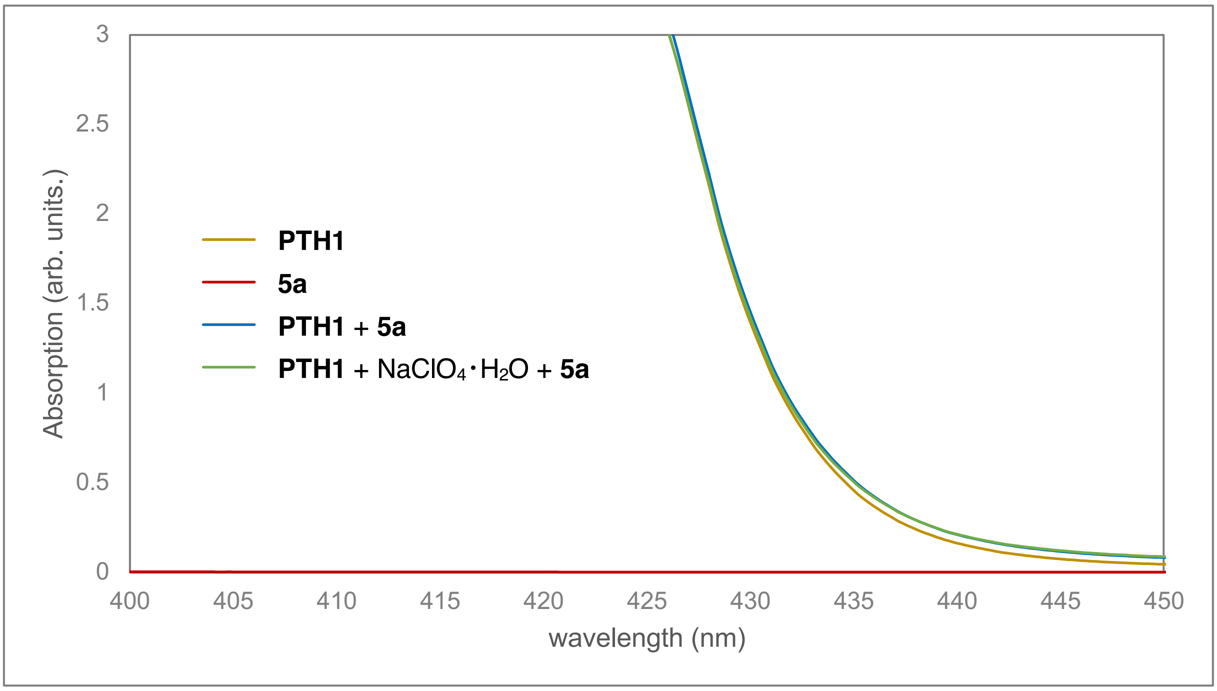

Supplementary Figure 6. UV-Vis spectra of the reaction mixture (identical concentration to the catalytic reaction condition).

11. Computational Study

To consider the intermediacy of alkylsulfonium species, the free energy gap between a free a-hydroxcarbocation and the corresponding alkylsulfonium were compared by DFT calculations. All density functional theory (DFT) calculations were performed with the Gaussian 16 package.[12] The method B3LYP functional with the 6-31G(d) basis set was used for the geometry optimization and normal vibrational mode analysis. The dispertion-corrected method BP86[13]-D3[14] functional with the 6-311+G(d,p) basis set was used to calculate single-point energies with SMD[15] model (dichloromethane).

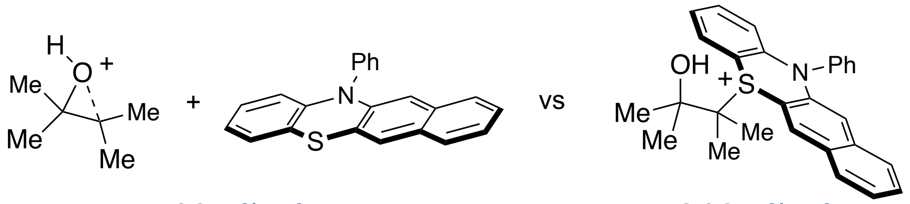

Supplementary Figure 7. Comparison of gibbs free energies. BP86-D3/6-311+G(d,p)// B3LYP/6-31G(d)

	α-hydroxycarbocation	N-Phenyl Bnezo[*b*]phenothiazine	Alkylsulfonium
E (hartree)	-311.5882712	-1300.6608	-1612.2881
Gcorr(hartree)	0.148283	0.259198	0.433796
Gibbs free energy (hartree)<br>Gibbs free energy (kcal/mol)	-311.4399882<br>-195431.707	-1300.401602<br>-816015.009	-1611.854304<br>-1011454.694

α-Hydroxycarbocaiton

C	-0.74482135	-0.00649490	-0.08054782
C	-1.52771065	-1.28054521	-0.26380144
H	-1.67816124	-1.43563933	-1.33920300
H	-1.02835902	-2.15877702	0.14285481
H	-2.51229748	-1.18970542	0.20223081
C	-1.54416225	1.25029512	-0.31647353
H	-2.47178733	1.21987604	0.26257075
H	-1.01376600	2.17703093	-0.09292282
H	-1.82336167	1.27747200	-1.37666140
C	0.74482265	-0.00649810	-0.08054687
C	1.54416812	1.25029190	-0.31646344
H	1.01378334	2.17702503	-0.09287017
H	2.47180741	1.21985085	0.26255640
H	1.82333545	1.27750069	-1.37665853
C	1.52770840	-1.28054799	-0.26380692
H	1.02833309	-2.15879518	0.14278609

H	1.67820913	-1.43560047	-1.33920856
H	2.51227503	-1.18973157	0.20227211
O	-0.00000498	-0.02635991	1.32609338
H	-0.00000035	0.87136774	1.72334657

N-phenyl Benzo[*b*]phenothiazine

C	3.97488050	-2.64323141	-0.77899187
C	2.76006840	-3.05829354	-0.23505044
C	1.77365571	-2.12818005	0.09753492
C	1.96334430	-0.75510382	-0.15555199
C	3.18907007	-0.35296616	-0.71159669
C	4.18412006	-1.28466233	-1.00614469
C	-0.42944937	-0.14788740	0.10801062
C	-0.85037528	-1.49012072	0.37432184
C	-2.17825602	-1.84311651	0.29771578
H	-2.46642405	-2.87404201	0.48765238
C	-3.18561496	-0.88633624	0.01078759
C	-2.78149365	0.46593862	-0.21566712
C	-1.40173031	0.79644961	-0.17473287
H	2.57288450	-4.11168384	-0.04549359
H	3.37120413	0.69660373	-0.90605096
H	-1.11933296	1.82478487	-0.36634171
N	0.94836187	0.18514486	0.16293644
S	0.32435450	-2.68651185	0.96273807
H	5.12350688	-0.93698196	-1.42706411
H	4.74257216	-3.37269301	-1.01912759
C	-3.77957531	1.43724700	-0.49431345
C	-5.11096683	1.08548565	-0.54881951
H	-5.86316115	1.83978981	-0.76402548
C	-5.50821879	-0.25480199	-0.32543032
H	-6.56098862	-0.51970555	-0.37128193
C	-4.56319401	-1.21943885	-0.05039016
H	-4.86136496	-2.25071338	0.12418350
H	-3.47506968	2.46713959	-0.66591105
C	1.30421136	1.57983947	0.20283505
C	1.59732434	2.16447065	1.43746656
C	1.35943481	2.35099929	-0.96472506
C	1.94565096	3.51451279	1.50567701
C	1.70799060	3.70056836	-0.89282565
C	2.00143053	4.28372000	0.34199269
H	2.17305881	3.96401563	2.46830194

H	1.75026866	4.29517294	-1.80128566
H	2.27271365	5.33442391	0.39626243
H	1.12905306	1.88841718	-1.92019476
H	1.54903164	1.55309307	2.33339906

Alkylsulfonium

C	2.79562757	2.42712099	-2.45780728
C	2.60695563	1.09649613	-2.11873705
C	1.53848349	0.72479920	-1.28673293
C	0.57430695	1.66012860	-0.84529910
C	0.81748436	3.01299395	-1.16995900
C	1.90518172	3.38110464	-1.94998561
C	-1.11384614	-0.02719573	-0.23573863
C	-0.30802280	-1.14063054	-0.63202110
C	-0.84620562	-2.39556105	-0.85779173
H	-0.20379749	-3.20549985	-1.19203856
C	-2.21398884	-2.64923017	-0.62901008
C	-3.02268361	-1.56876550	-0.14498572
C	-2.45256416	-0.28497320	0.02757588
H	3.28755500	0.33319101	-2.48463850
H	0.12839607	3.77262718	-0.82548608
H	-3.09946532	0.52183087	0.34842105
N	-0.57996581	1.27832164	-0.14871412
S	1.44908385	-0.97803223	-0.80628439
H	2.04574543	4.43156328	-2.18628728
H	3.62079083	2.71953103	-3.09801076
C	2.17756560	-1.23152601	1.00662002
C	1.64509842	-2.61876202	1.39217573
H	1.90304824	-3.39698879	0.66790818
H	2.10678898	-2.89029755	2.34384801
H	0.56134156	-2.60611393	1.52384036
C	1.64112221	-0.16523660	1.95646716
H	0.56440942	-0.27886217	2.10529676
H	2.13550152	-0.30966662	2.92280799
H	1.84838944	0.85168901	1.61626566
C	-4.39769036	-1.81591202	0.11930214
C	-4.93459299	-3.06528106	-0.08967070
H	-5.98626625	-3.24214345	0.11538807
C	-4.13153813	-4.13145986	-0.57249071
H	-4.57732784	-5.10835028	-0.73194813
C	-2.79809734	-3.92888054	-0.83680444

H	-2.17590560	-4.74026803	-1.20509453
H	-5.01842339	-1.00295693	0.48564790
C	-1.42728028	2.32054582	0.39827125
C	-1.28661821	2.67653997	1.74052692
C	-2.37330498	2.95835859	-0.41148532
C	-2.09732621	3.67916045	2.27668822
C	-3.17998798	3.95954593	0.13068158
C	-3.04246682	4.31998758	1.47371493
H	-1.98865591	3.95792881	3.32046334
H	-3.91394885	4.45713727	-0.49605506
C	3.76208970	-1.23028059	0.91938042
H	-3.67130116	5.09971887	1.89283321
H	-2.47240381	2.67060462	-1.45428223
H	-0.54840003	2.17272243	2.35682972
C	4.38077177	0.17381629	0.82602227
H	4.12017539	0.78007816	1.69649631
H	5.47305999	0.07704770	0.80342758
H	4.08893430	0.71519459	-0.07645587
C	4.32478740	-2.13230224	-0.19325040
H	3.92021933	-3.14599857	-0.14246843
H	4.14614863	-1.73322130	-1.19752407
H	5.41045943	-2.20608282	-0.06824200
O	4.10613605	-1.79364418	2.19511135
H	5.06070060	-1.67260308	2.32577547

12. Reaction Mechanism via A Discrete Carbocation Intermedaite

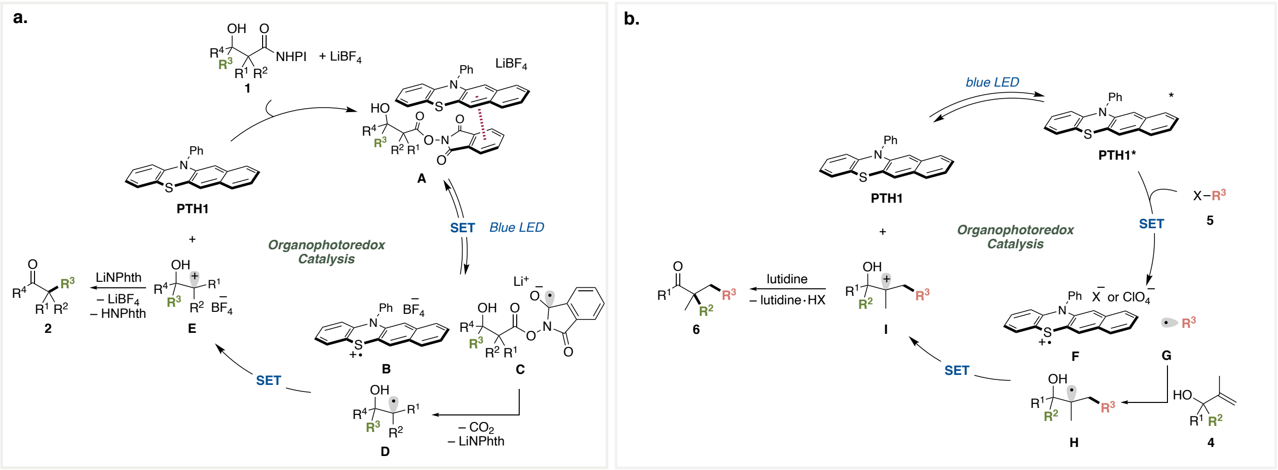

Supplementary Figure 8. Alternative reaction mechanisms.

■ References■

1) Touney, E. E., Foy, N. J., Pronin, S. V. Catalytic Radical–Polar Crossover Reactions of Allylic Alcohols. *J. Am. Chem. Soc.* **140,** 16982–16987 (2018).

2) (a) Shibutani, S., Nagao, K., Ohmiya, H. Organophotoredox-Catalyzed Three-Component Coupling of Heteroatom Nucleophiles, Alkenes and Aliphatic Redox Active Esters. *Org. Lett.* **23**, 1798–1803 (2021). (b) Shibutani, S., Kodo, T., Takeda, M., Nagao, K., Tokunaga, N., Sasaki, Y., Ohmiya, H. Organophotoredox-Catalyzed Decarboxylative C(sp^3)–O Bond Formation. *J. Am. Chem. Soc.* **142**, 1211–1216 (2020). (c) Watanabe, M., Hagiwara, H., Iribe, A., Ogata, Y., Shiomi, K., Staykov, A., Ida, S., Tanaka, K., Ishihara, T. Spacer Effects in Metal-free Organic Dyes for Visible-light-driven Dye-sensitized Photocatalytic Hydrogen Production. *J. Mater. Chem. A*, **2**, 12952–12961 (2014). (d) Chen, D. -G., Chen, Y., Wu, C. -H., Chen, Y. -A., Chen, M. -C., Lin, J. -A., Huang, C. -Y., Su, J., Tian, H., Chou, P. -T.

3) van Beek, R., Zoombelt, A. P., Jenneskens, L. W., van Walree, C. A., de Mello Donegá, C., Veldman, D., Janssen, R. A. J. Side Chain Mediated Electronic Contact between a Tetrahydro-4H-thiopyran-4-ylidene-Appended Polythiophene and CdTe Quantum Dots. *Chem. Eur. J.* **12**. 8075–8083 (**2006**).

4) (a)Qin, T., Cornella, J., Li, C., Malins, L. R., Edwards, J. T., Kawamura, S., Maxwell, B. D., Eastgate, M. D., Baran, P. S. A general alkyl-alkyl cross-coupling enabled by redox-active esters and alkylzinc reagents *Science.* **352**, 801–805 (2016). (b) Suzuki, N., Hofstra, J. L., Poremba, K. E., Reisman, S. E. Nickel-Catalyzed Enantioselective Cross-Coupling of *N*-Hydroxyphthalimide Esters with Vinyl Bromides. *Org. Lett.* **19**, 8571–8582 (2017).

5) Martin, R., Buchwald, S. L. An Improved Protocol for the Pd-Catalyzed α-Arylation of Aldehydes with Aryl Halides. *Org. Lett.* **10**, 4561–4564 (2008).

6) Nareddy, P., Mazet, C. α-Arylation, α-Arylative Esterification, or Acylation: A Stoichiometry-Dependent Trichotomy in the Pd-Catalyzed Cross-Coupling between Aldehydes and Aryl Bromides. *Chem. Asian. J.* **8**, 2579–2583 (2013).

7) Gutiérrez-Bonet, Á., Flores-Gaspar, A., Martin, R. Fe-Catalyzed Regiodivergent [1,2]-Shift of α-Aryl Aldehydes. *J. Am. Chem. Soc.* **135**, 12576–12579 (2013).

8) Vo, G. D., Hartwig, J. F. Palladium-Catalyzed α-Arylation of Aldehydes with Bromo- and Chloroarenes Catalyzed by [{Pd(allyl)Cl}2] and dppf or Q-phos. *Angew. Chem. Int. Ed.* **47**, 2127–2130 (2008).

9) Sonawane, R. P., Jheengut, V., Rabalakos, C., Larouche-Gauthier, R., Scott, H. K., Aggarwal, V. K. Enantioselective Construction of Quaternary Stereogenic Centers from Tertiary Boronic Esters: Methodology and Applications. *Angew. Chem. Int. Ed.* **50**, 3760–3763 (2011).

10) Ogasawara, K., Yamada, O. *Trans*-3-Hydroxy-4-Phenyltheioetheiotetrahydrofuran And Its Production Patent No. JP3060915B2, (1997)

11) Iwama, T., Rawal, V. H. Palladium-Catalyzed Regiocontrolled α-Arylation of Trimethylsilyl Enol Ethers with Aryl Halides. *Org. Lett.* **8**, 5725–5728 (2006).

12) Gaussian 16, Revision C.01, Frisch, M. J.; Trucks, G. W.; Schlegel, H. B.; Scuseria, G. E.; Robb, M. A.; Cheeseman, J. R.; Scalmani, G.; Barone, V.; Petersson, G. A.; Nakatsuji, H.; Li, X.; Caricato, M.; Marenich, A. V.; Bloino, J.; Janesko, B. G.; Gomperts, R.; Mennucci, B.; Hratchian, H. P.; Ortiz,

J. V.; Izmaylov, A. F.; Sonnenberg, J. L.; Williams-Young, D.; Ding, F.; Lipparini, F.; Egidi, F.; Goings, J.; Peng, B.; Petrone, A.; Henderson, T.; Ranasinghe, D.; Zakrzewski, V. G.; Gao, J.; Rega, N.; Zheng, G.; Liang, W.; Hada, M.; Ehara, M.; Toyota, K.; Fukuda, R.; Hasegawa, J.; Ishida, M.; Nakajima, T.; Honda, Y.; Kitao, O.; Nakai, H.; Vreven, T.; Throssell, K.; Montgomery, J. A., Jr.; Peralta, J. E.; Ogliaro, F.; Bearpark, M. J.; Heyd, J. J.; Brothers, E. N.; Kudin, K. N.; Staroverov, V. N.; Keith, T. A.; Kobayashi, R.; Normand, J.; Raghavachari, K.; Rendell, A. P.; Burant, J. C.; Iyengar, S. S.; Tomasi, J.; Cossi, M.; Millam, J. M.; Klene, M.; Adamo, C.; Cammi, R.; Ochterski, J. W.; Martin, R. L.; Morokuma, K.; Farkas, O.; Foresman, J. B.; Fox, D. J. Gaussian, Inc., Wallingford CT, 2016.

13) (a) Becke, A. D. *Phys Rev A Gen Phys* **38**, 3098–3100, (1988). (b) Perdew, J. P. *Phys Rev B Condens Matter* **33**, 8822–8824, (1986).

14) Grimme, S., Ehrlich, S., Goerigk, L. *J. Comput. Chem.* **32**, 1456–1465, (2011).

15) Marenich, A. V., Cramer, C. J., Truhlar, D. G. *J. Phys. Chem. B*, **113**, 6378–6396, (2009).

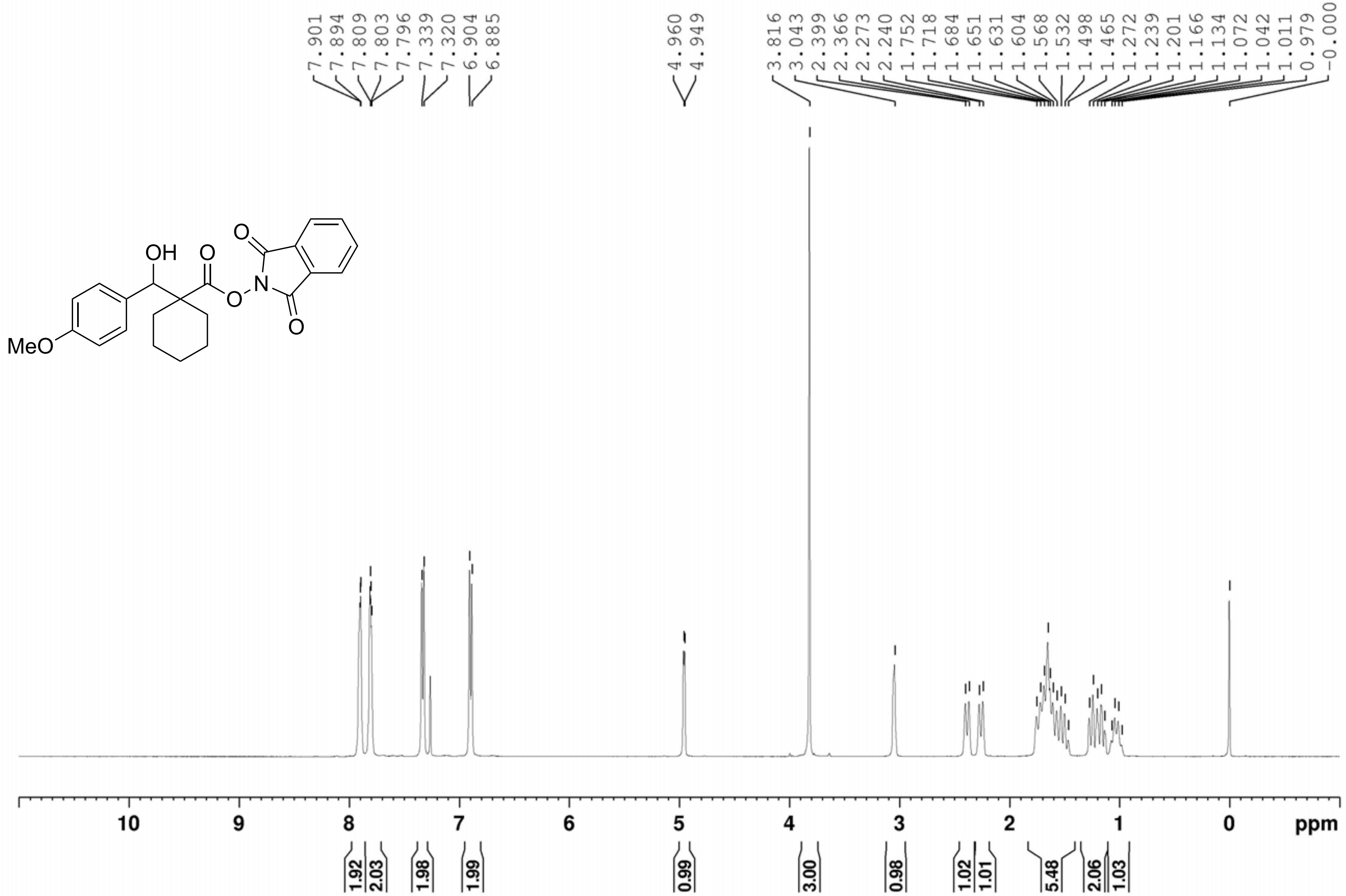

Supplementary Figure 9. ^{1}H NMR spectrum of **1a** (400 MHz, $CDCl_3$)

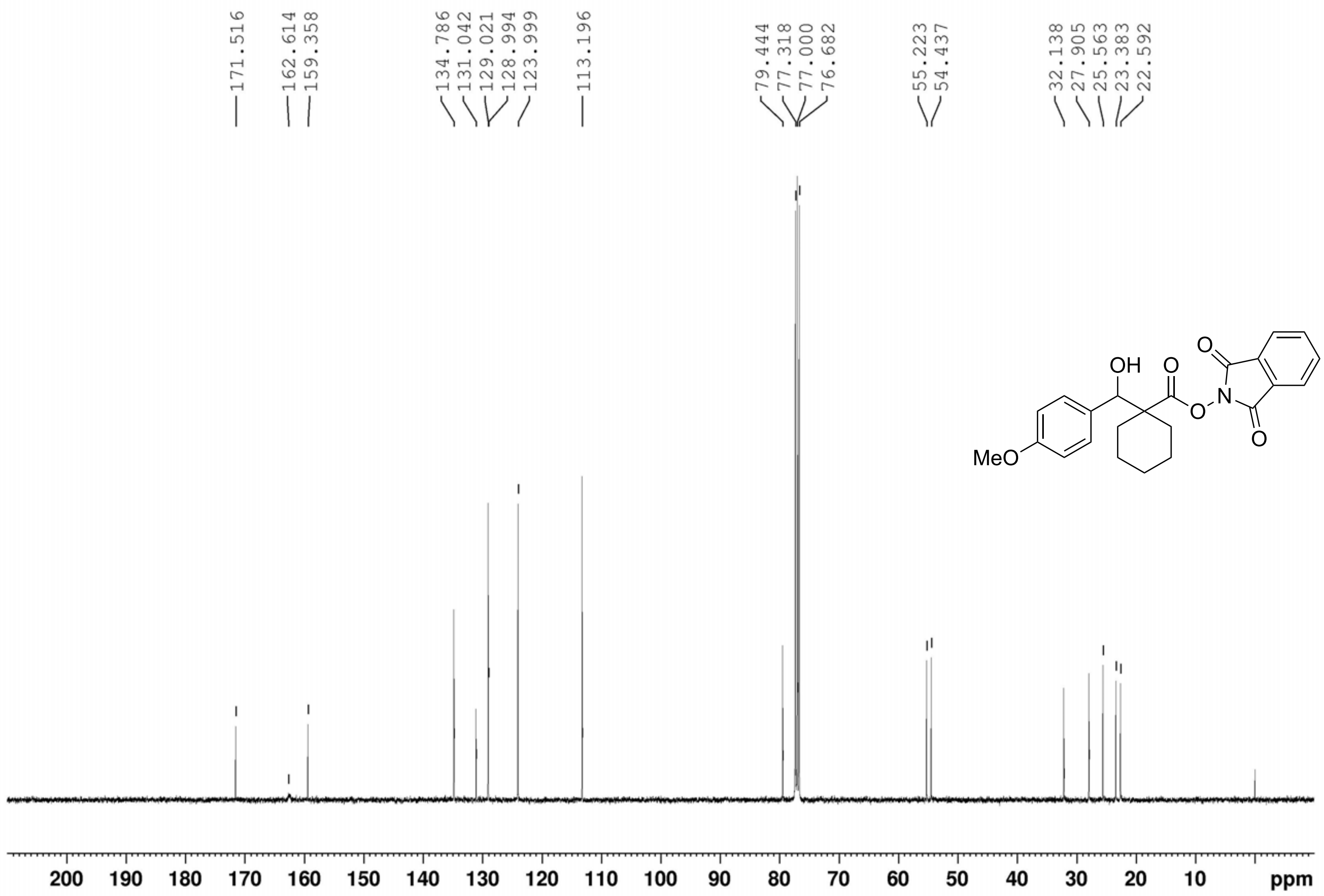

Supplementary Figure 10. ^{13}C NMR spectrum of **1a** (100.6 MHz, $CDCl_3$)

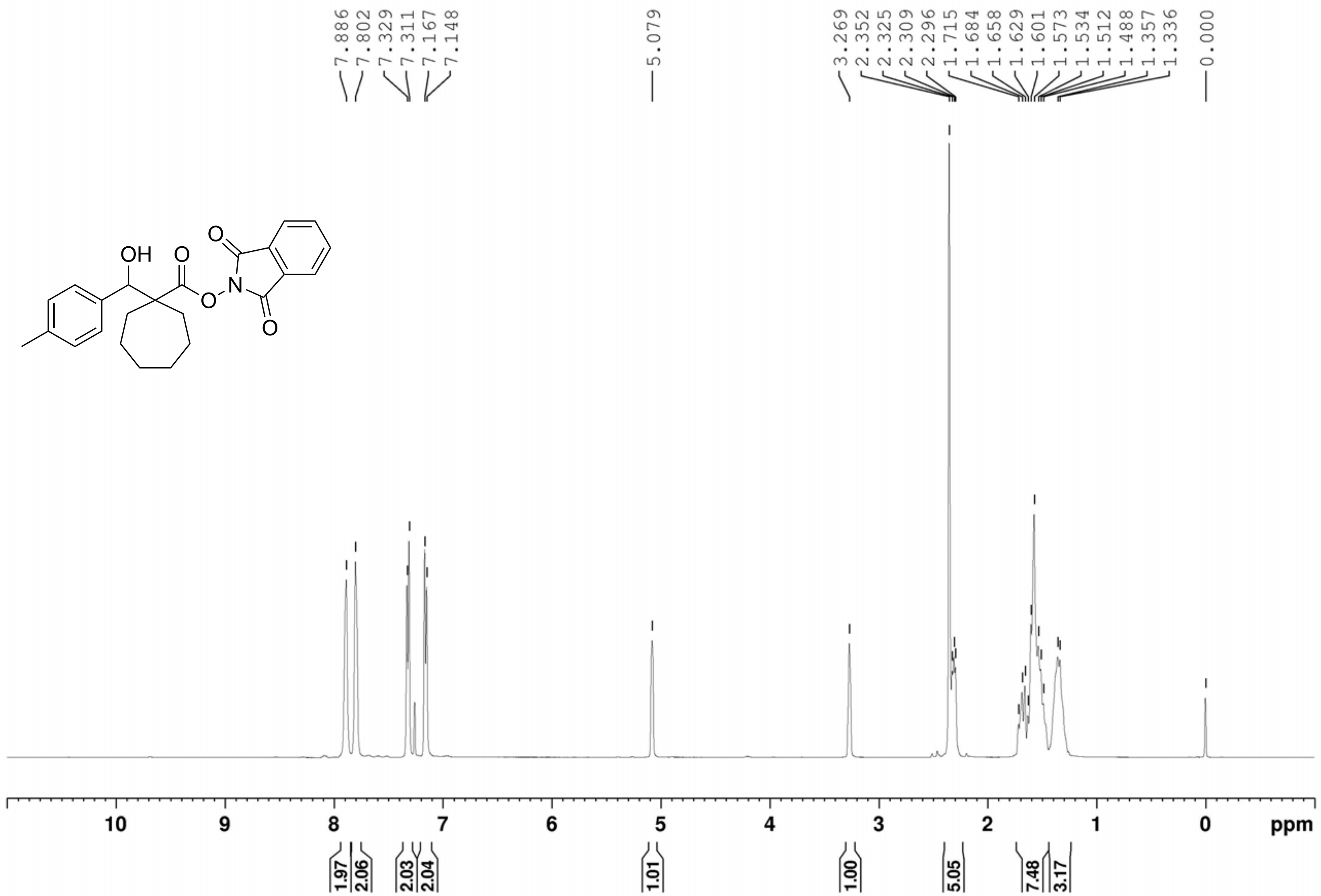

Supplementary Figure 11. ^{1}H NMR spectrum of **1b** (400 MHz, $CDCl_3$)

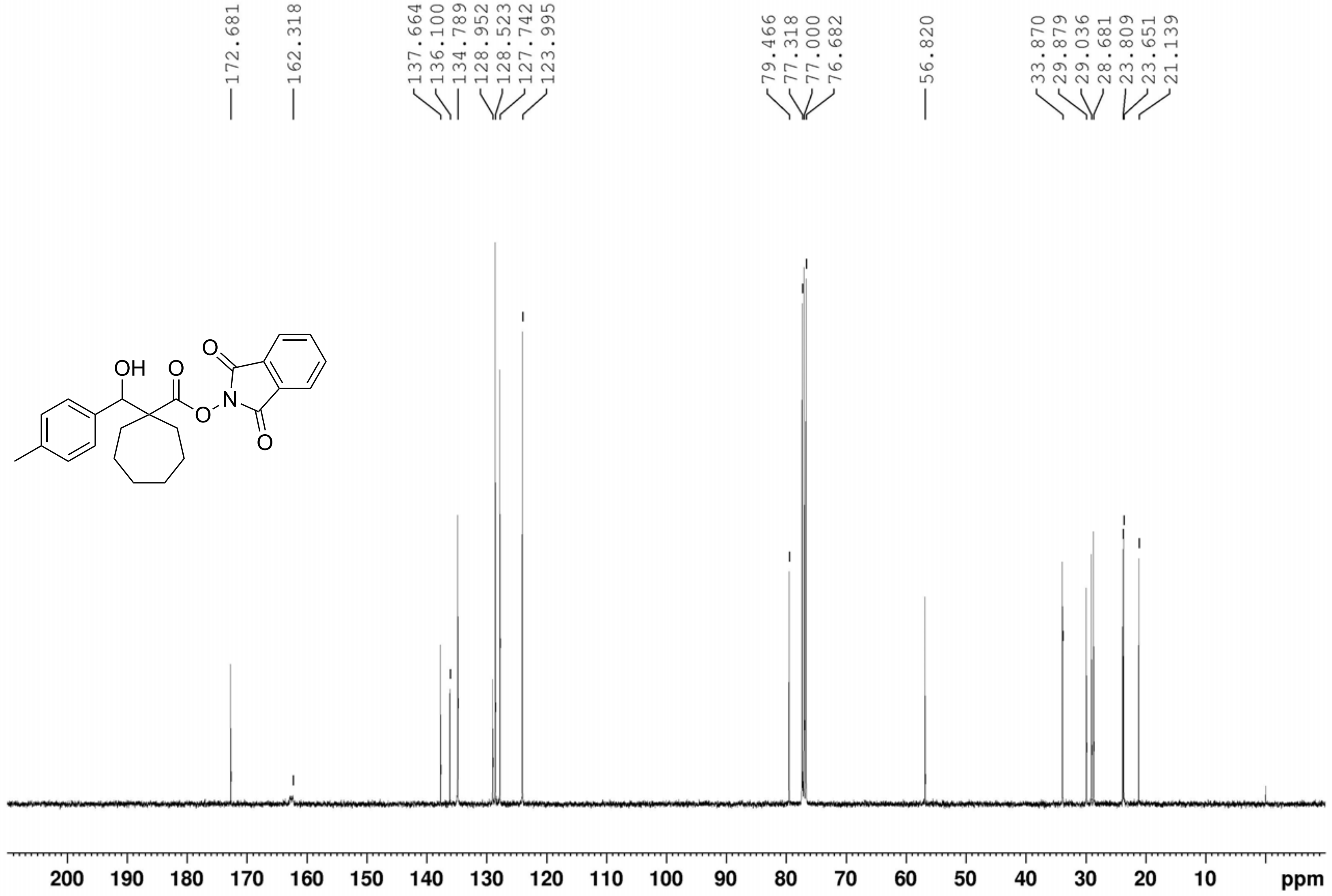

Supplementary Figure 12. ^{13}C NMR spectrum of **1b** (100.6 MHz, $CDCl_3$)

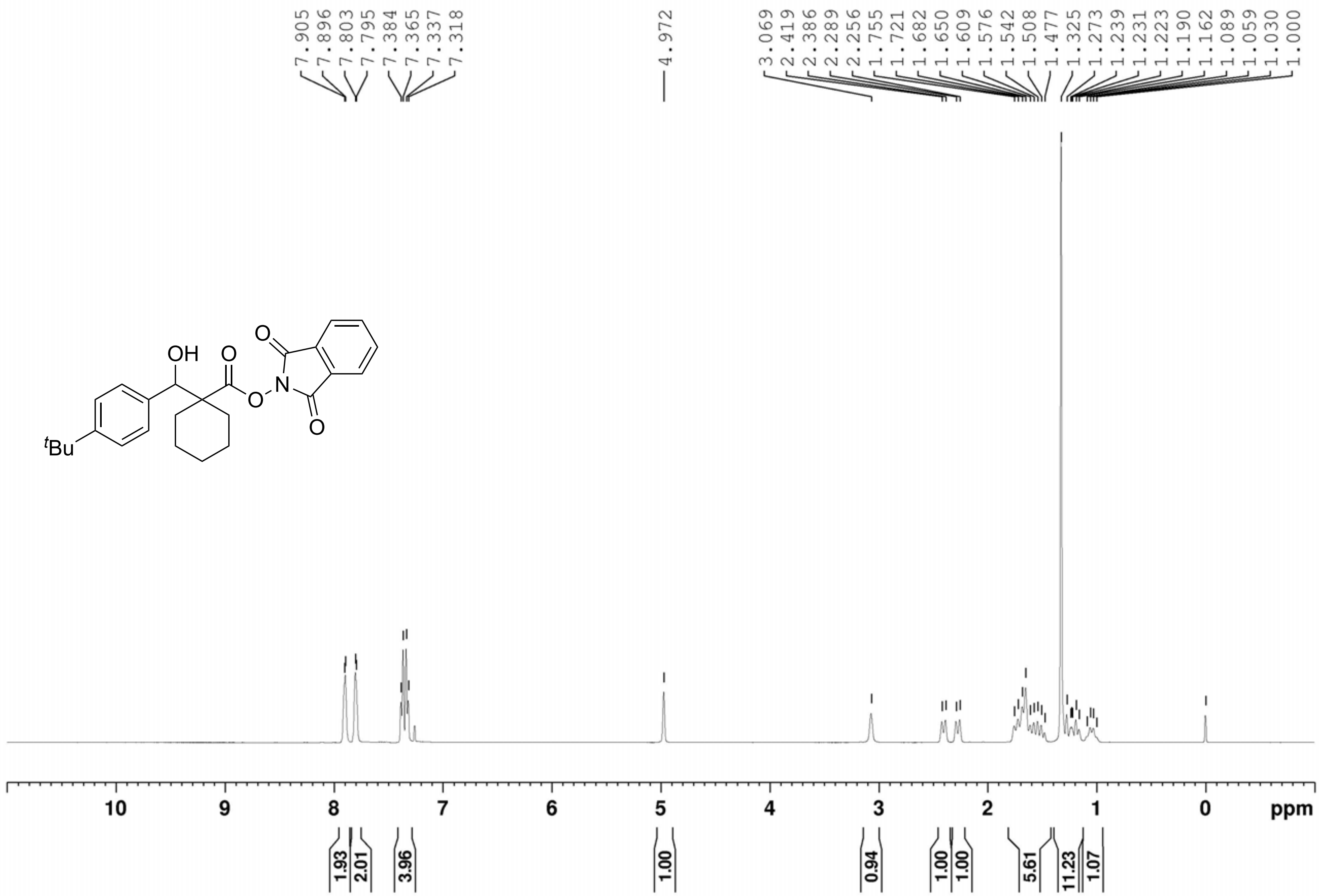

Supplementary Figure 13. ^{1}H NMR spectrum of **1c** (400 MHz, $CDCl_3$)

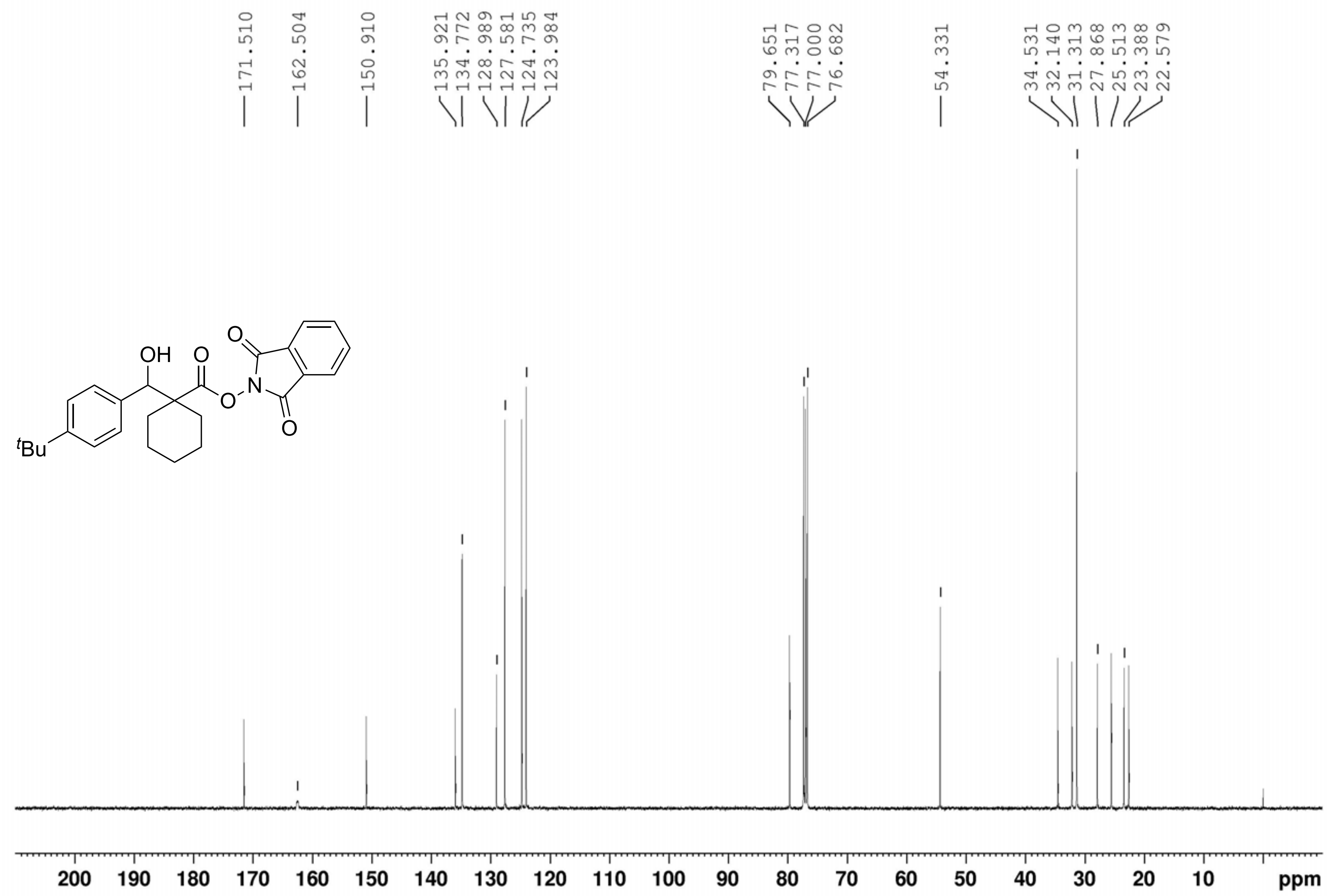

Supplementary Figure 14. ^{13}C NMR spectrum of **1c** (100.6 MHz, $CDCl_3$)

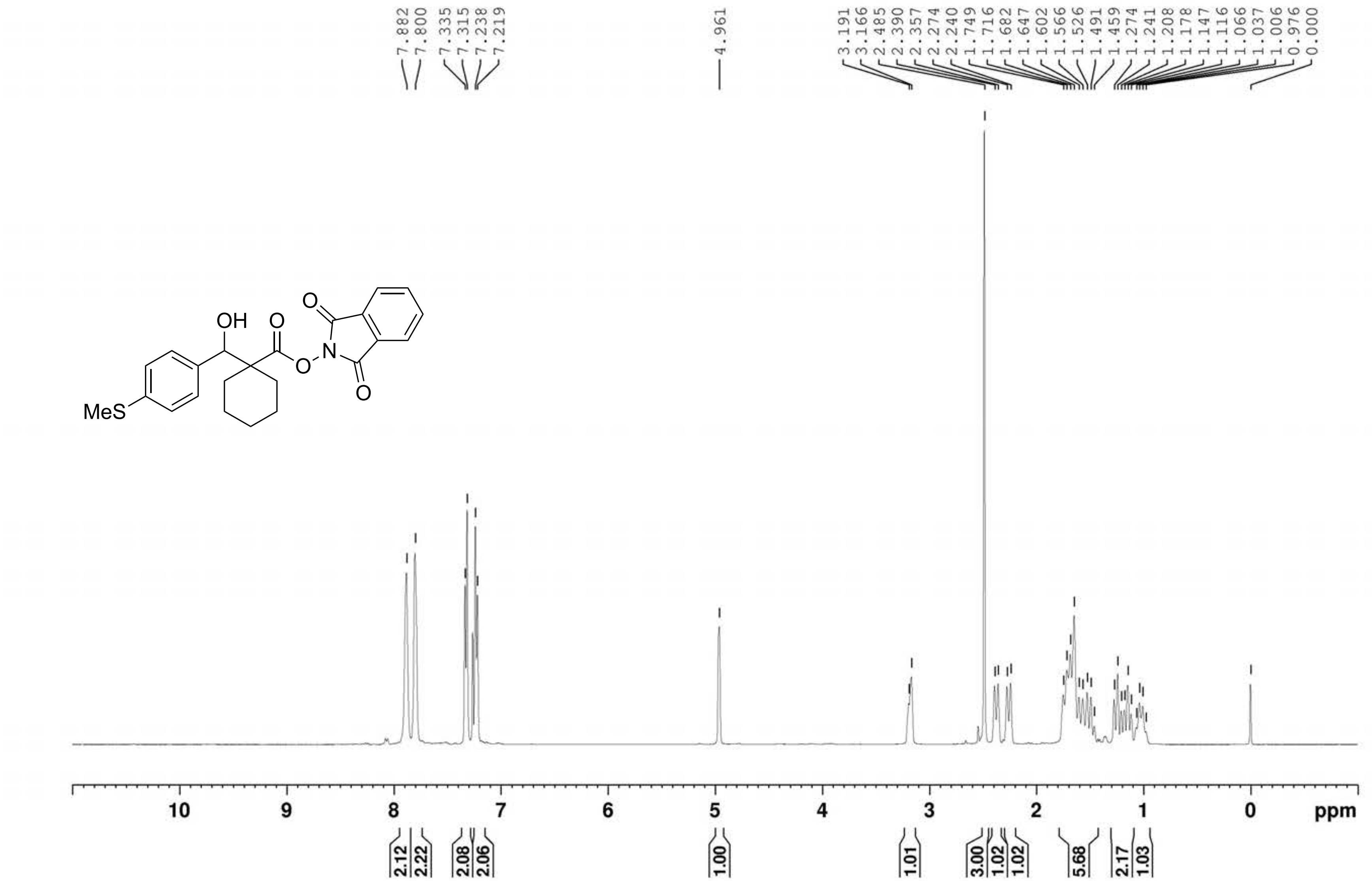

Supplementary Figure 15. ^{1}H NMR spectrum of **1d** (400 MHz, $CDCl_3$)

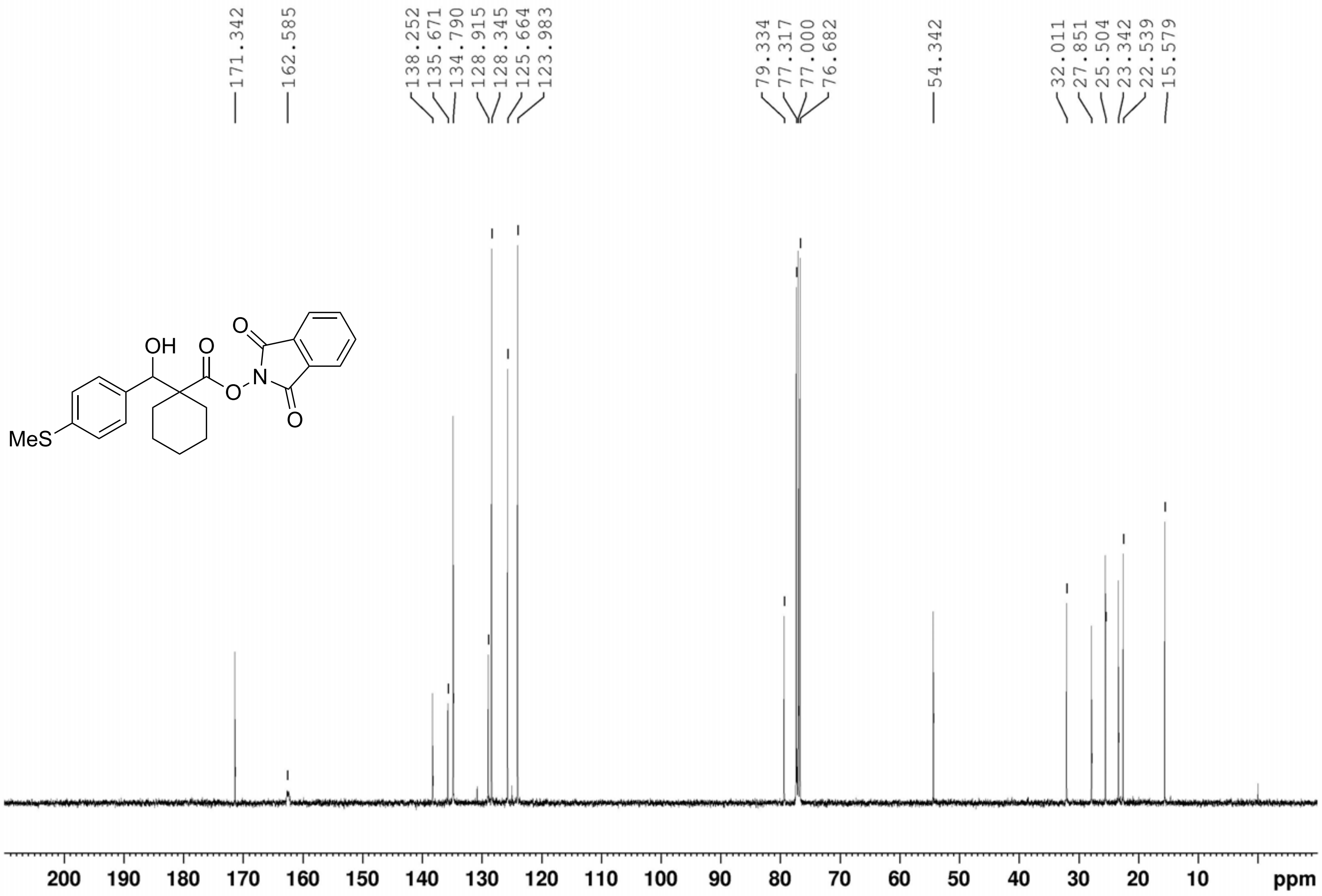

Supplementary Figure 16. ^{13}C NMR spectrum of **1d** (100.6 MHz, $CDCl_3$)

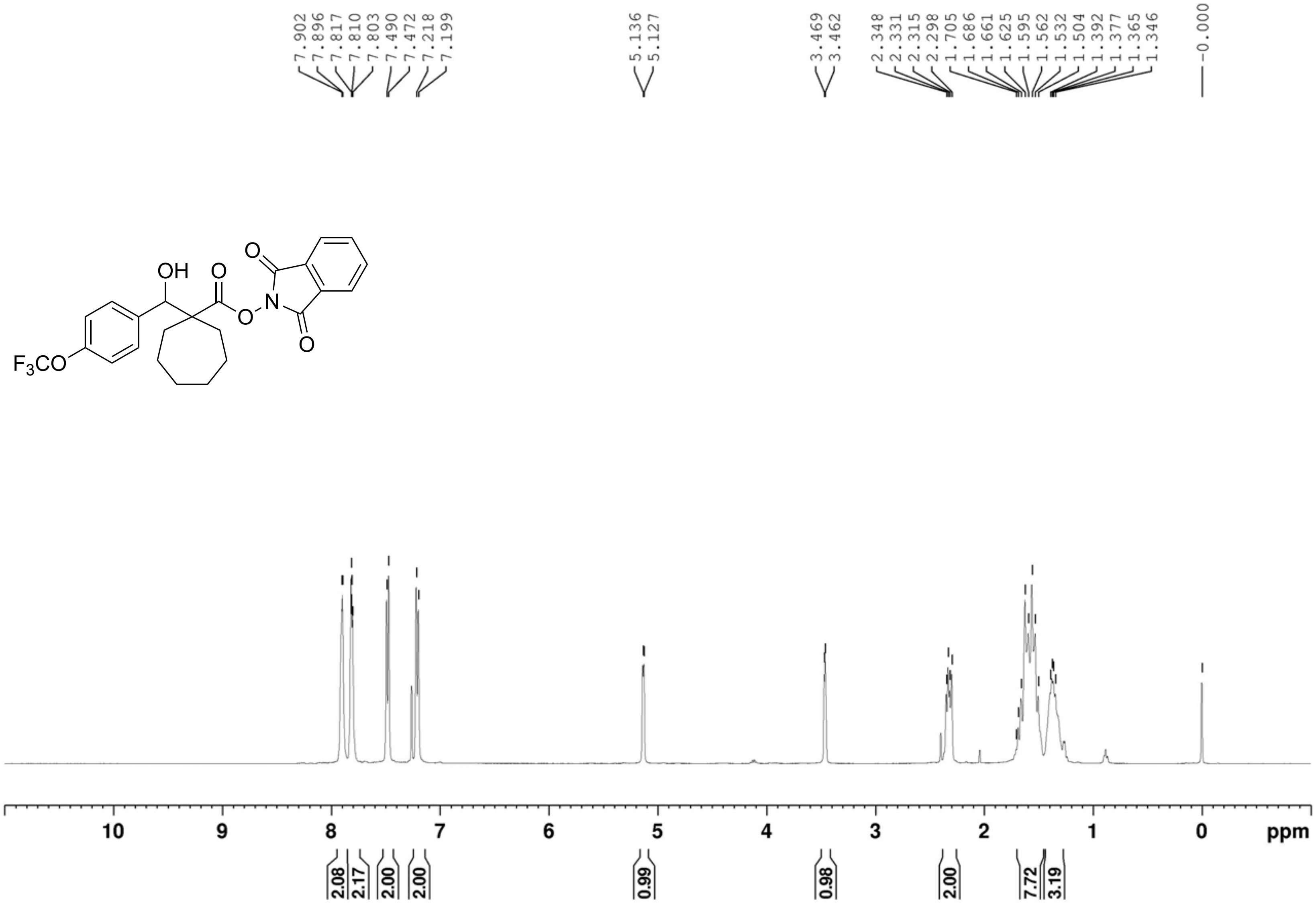

Supplementary Figure 17. ^{1}H NMR spectrum of **1e** (400 MHz, $CDCl_3$)

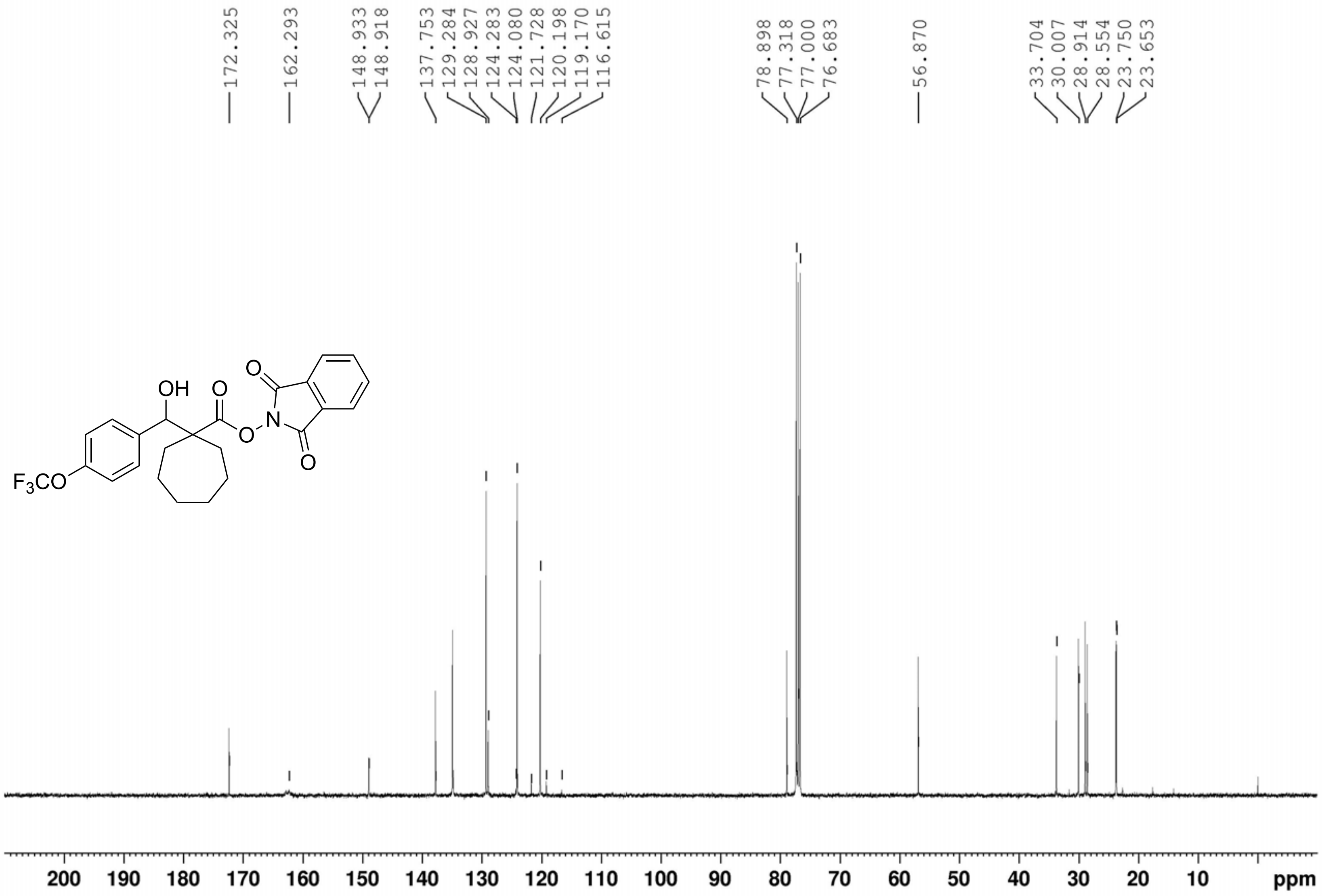

Supplementary Figure 18. ^{13}C NMR spectrum of **1e** (100.6 MHz, $CDCl_3$)

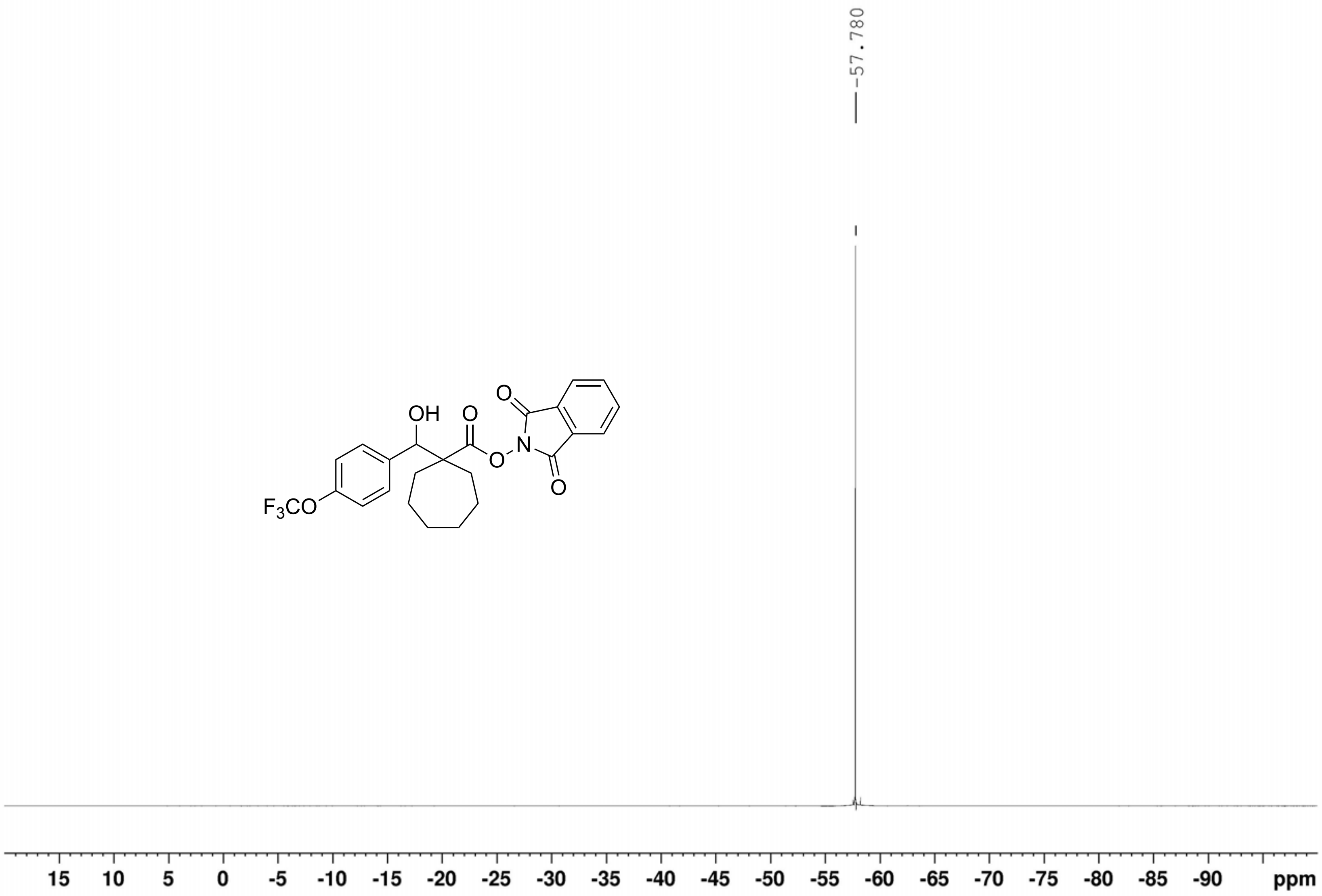

Supplementary Figure 19. ^{19}F NMR spectrum of **1e** (376 MHz, $CDCl_3$)

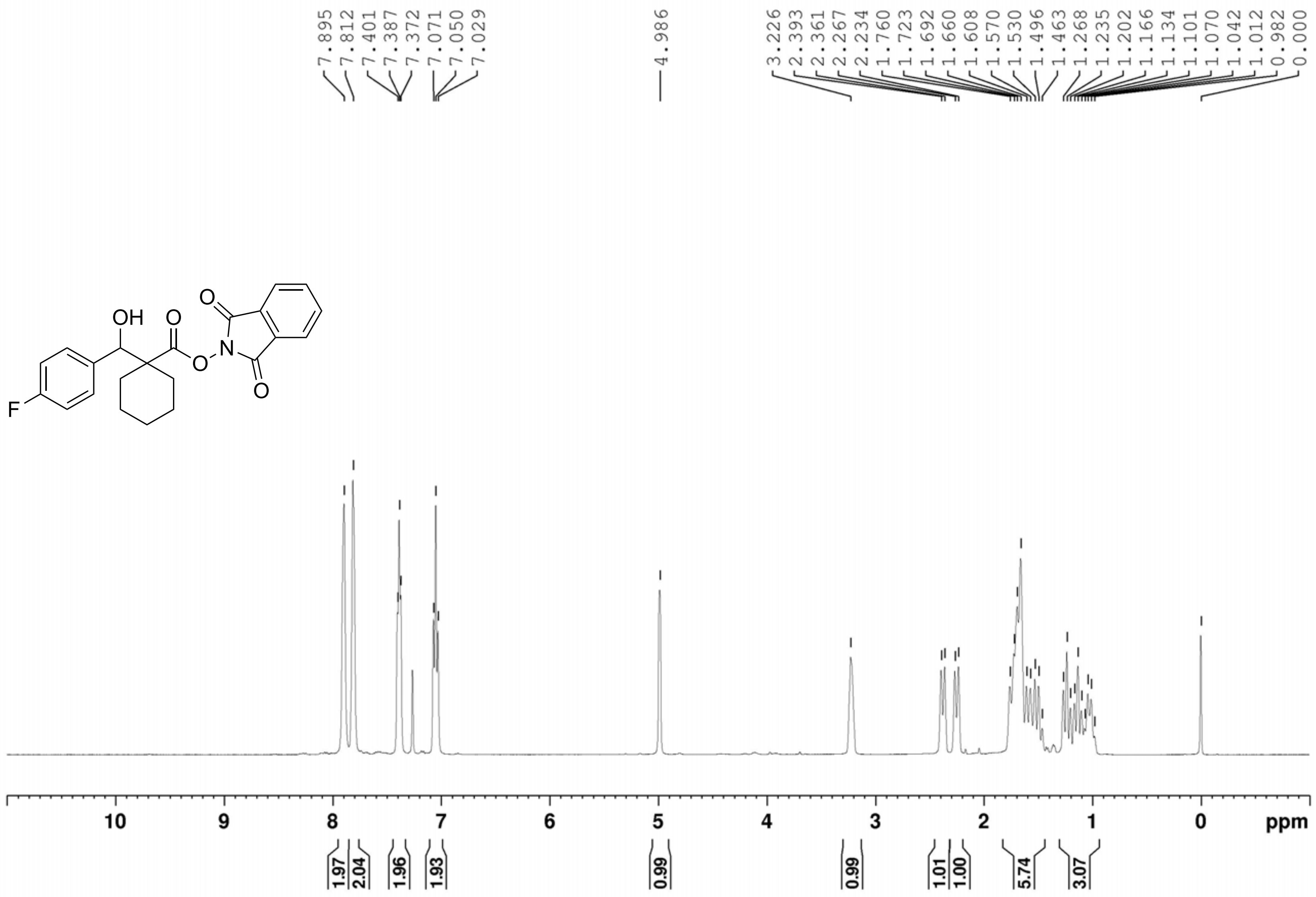

Supplementary Figure 20. ^{1}H NMR spectrum of **1f** (400 MHz, $CDCl_3$)

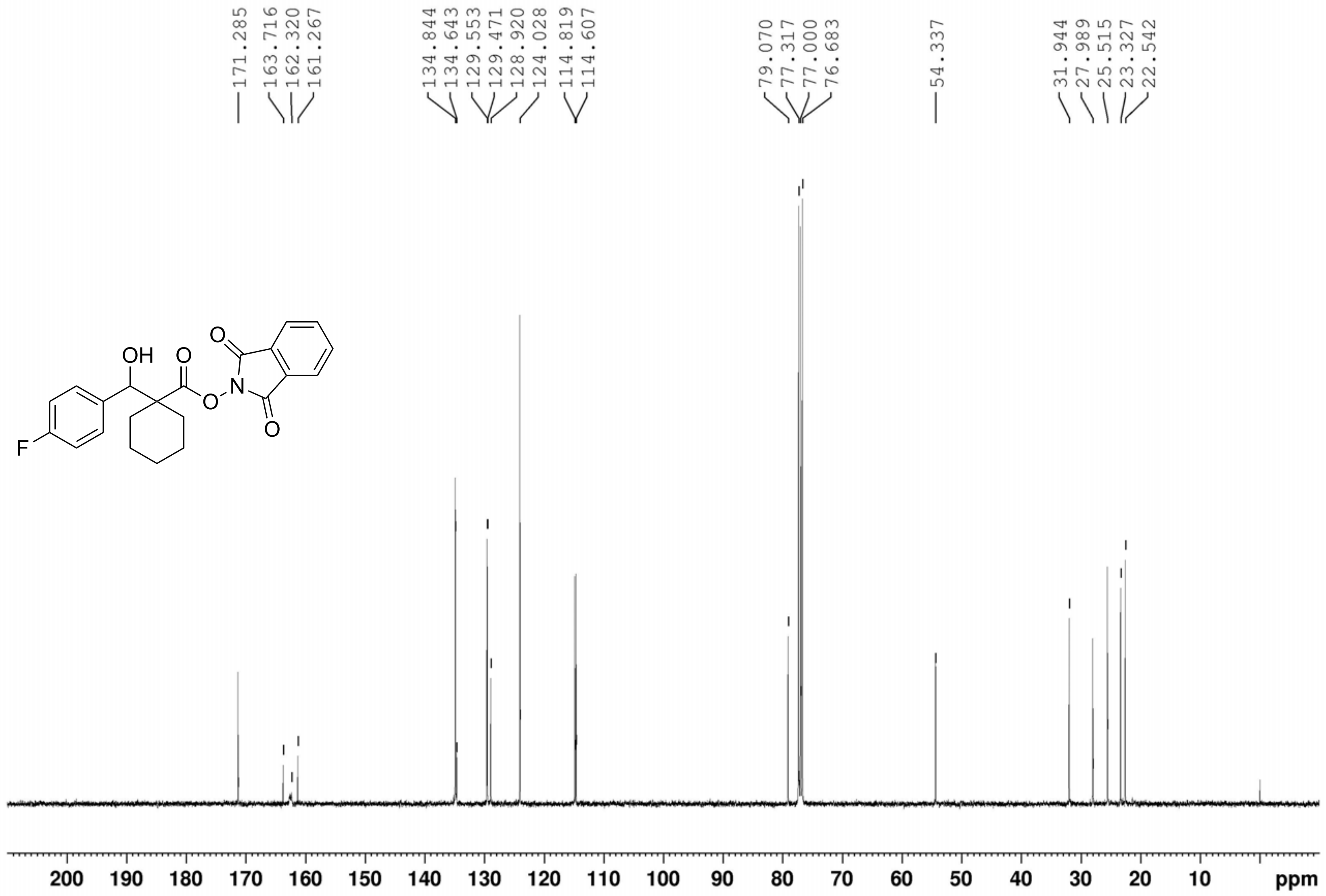

Supplementary Figure 21. ^{13}C NMR spectrum of **1f** (100.6 MHz, $CDCl_3$)

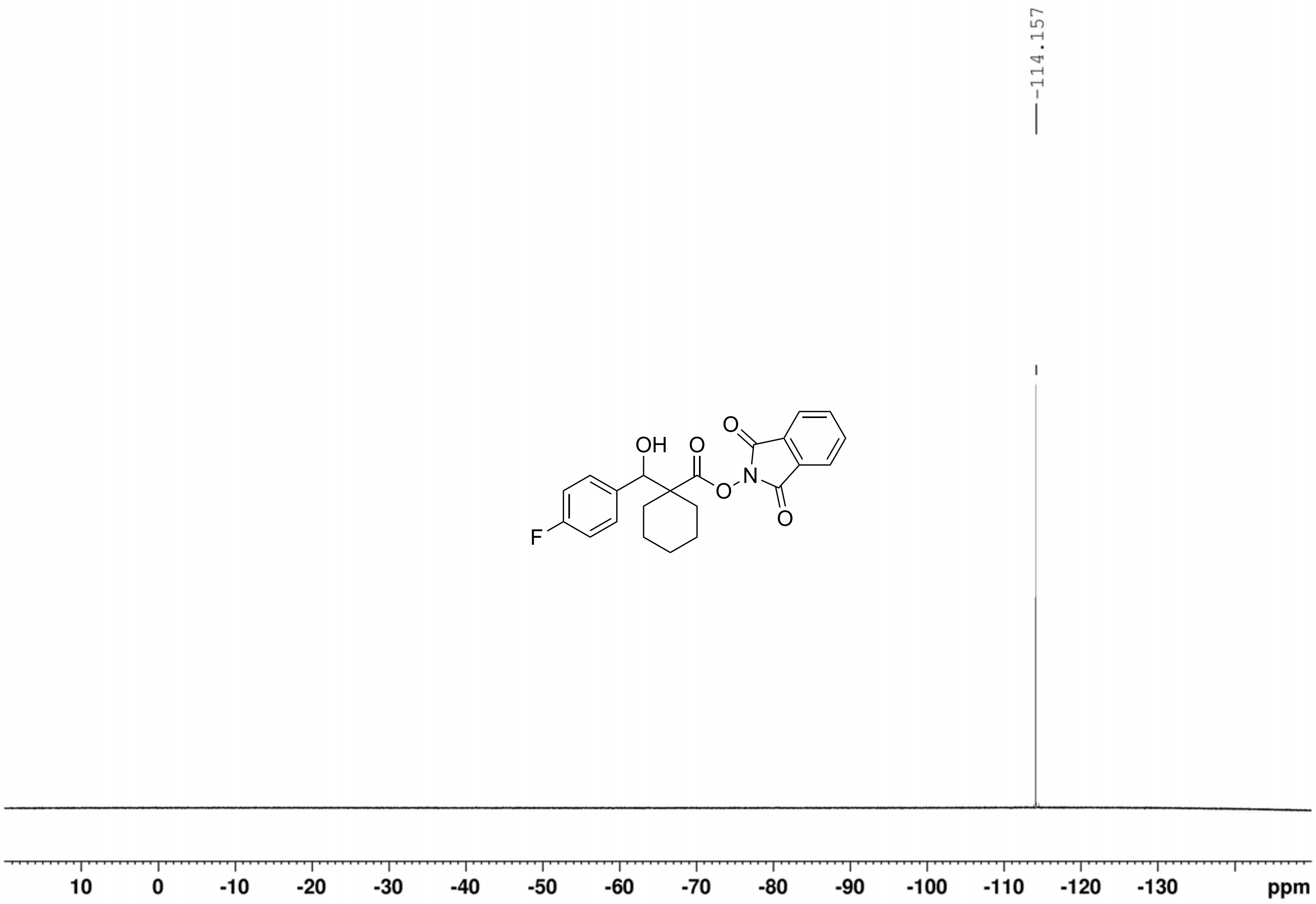

Supplementary Figure 22. ^{19}F NMR spectrum of **1f** (376 MHz, $CDCl_3$)

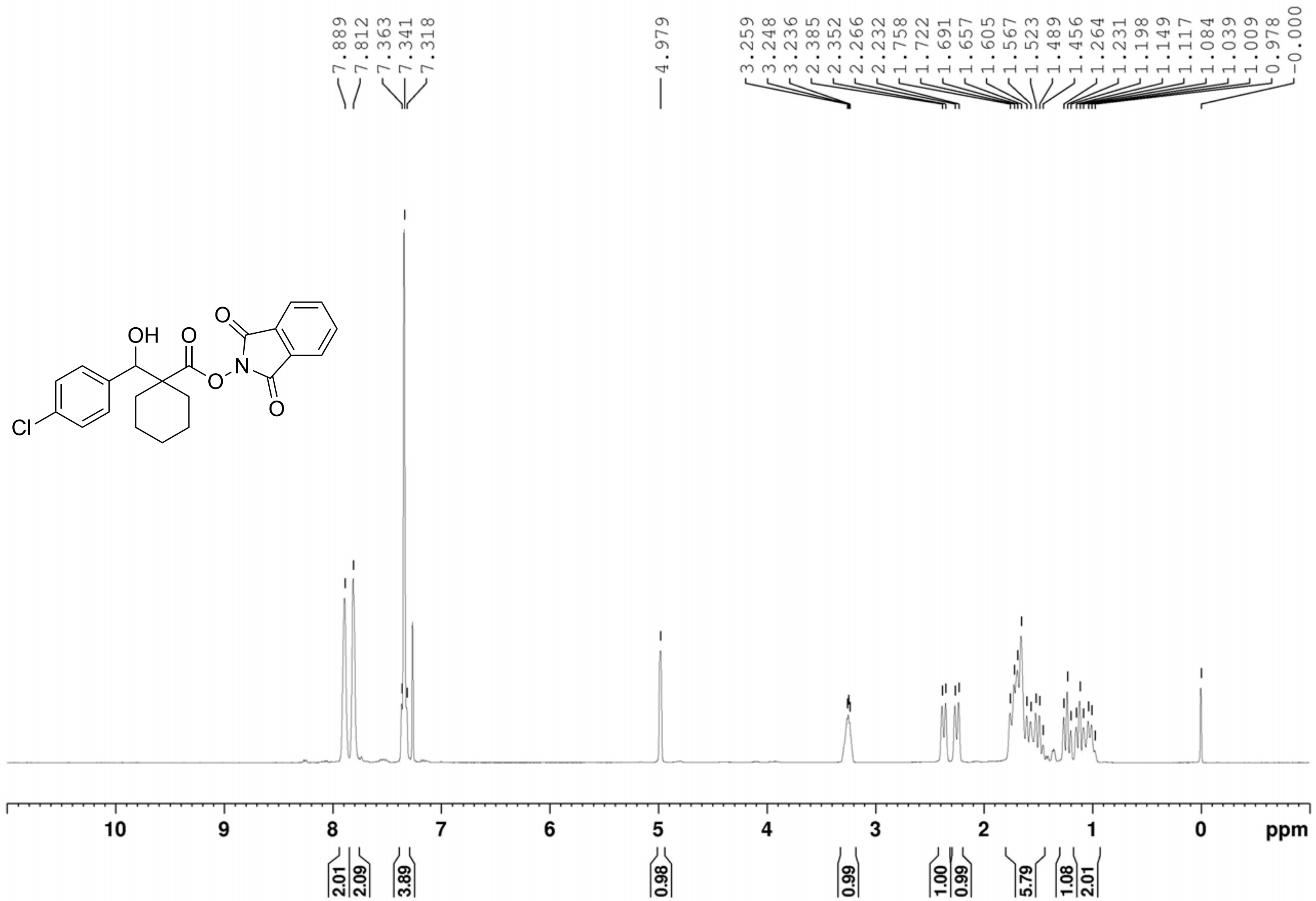

Supplementary Figure 23. ^{1}H NMR spectrum of **1g** (400 MHz, $CDCl_3$)

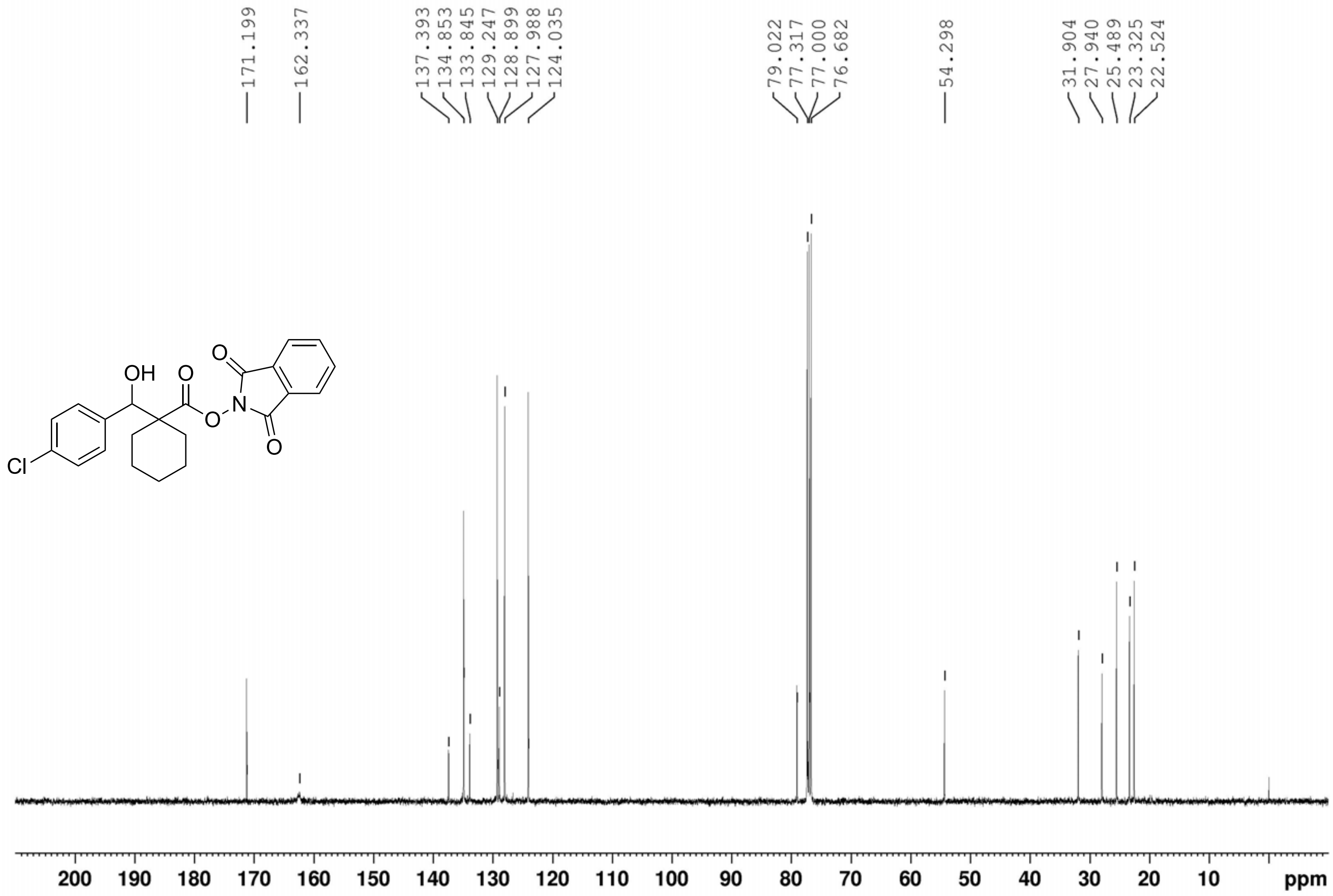

Supplementary Figure 24. ^{13}C NMR spectrum of **1g** (100.6 MHz, $CDCl_3$)

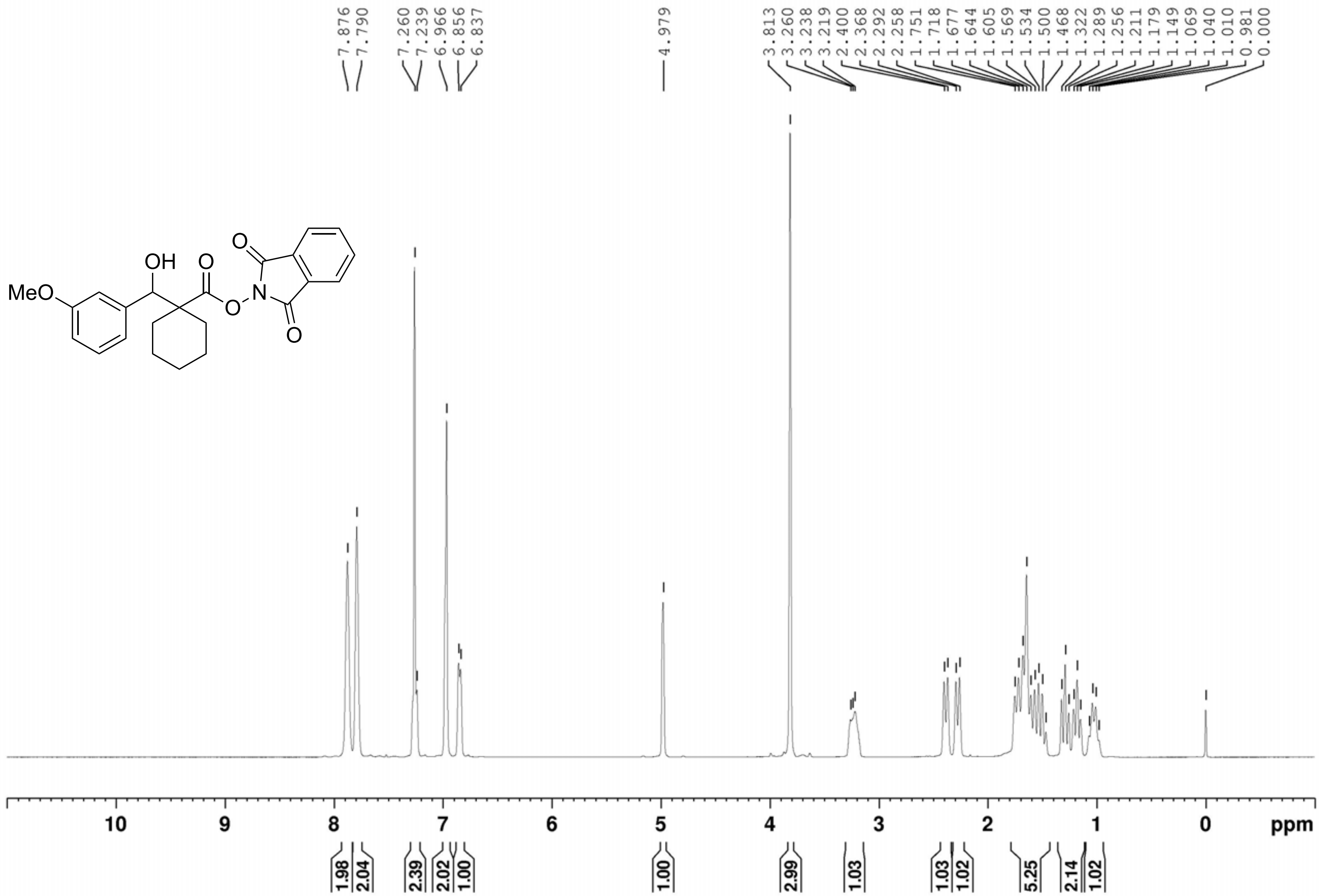

Supplementary Figure 25. ^{1}H NMR spectrum of **1h** (400 MHz, $CDCl_3$)

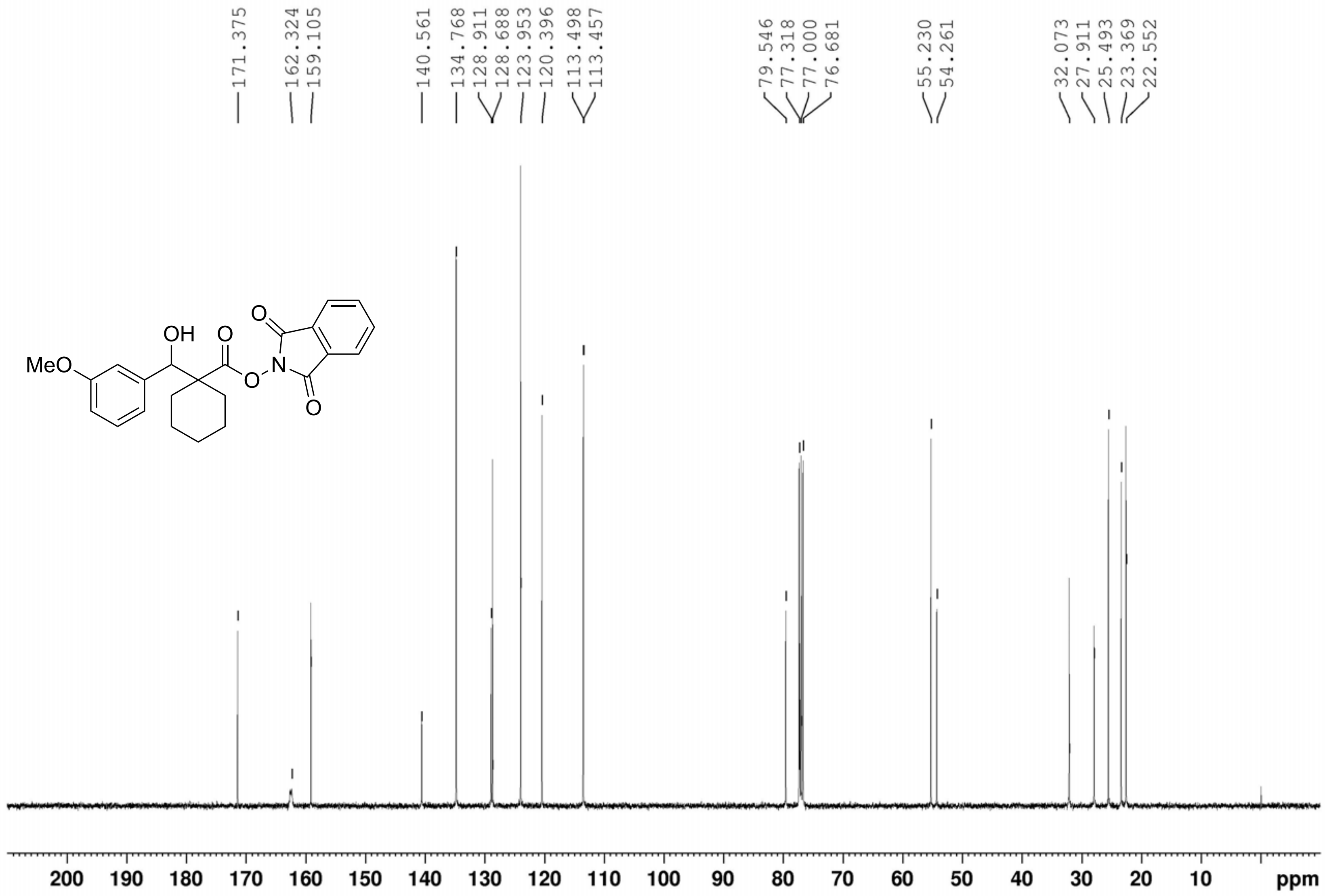

Supplementary Figure 26. ^{13}C NMR spectrum of **1h** (100.6 MHz, $CDCl_3$)

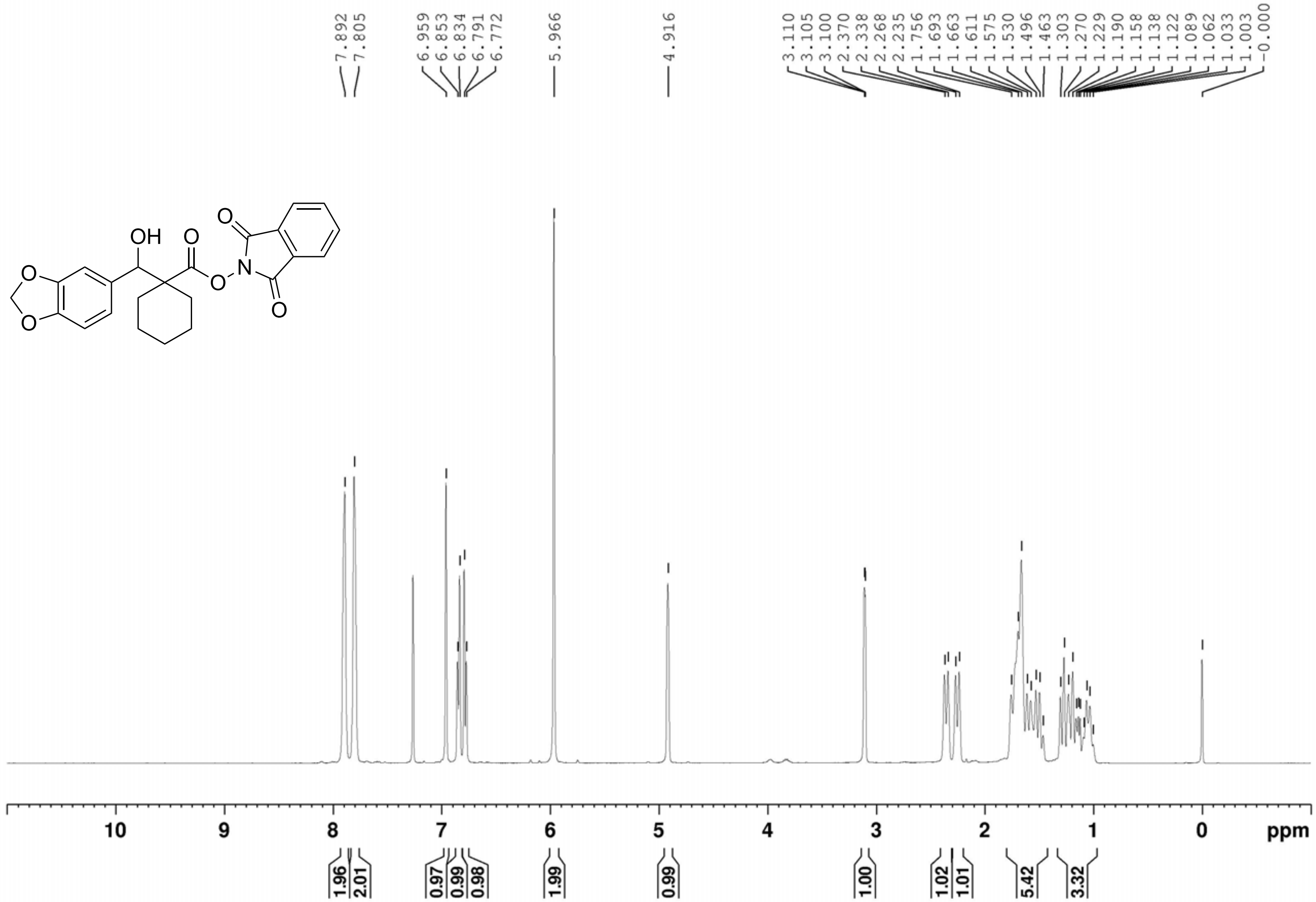

Supplementary Figure 27. ^{1}H NMR spectrum of **1i** (400 MHz, $CDCl_3$)

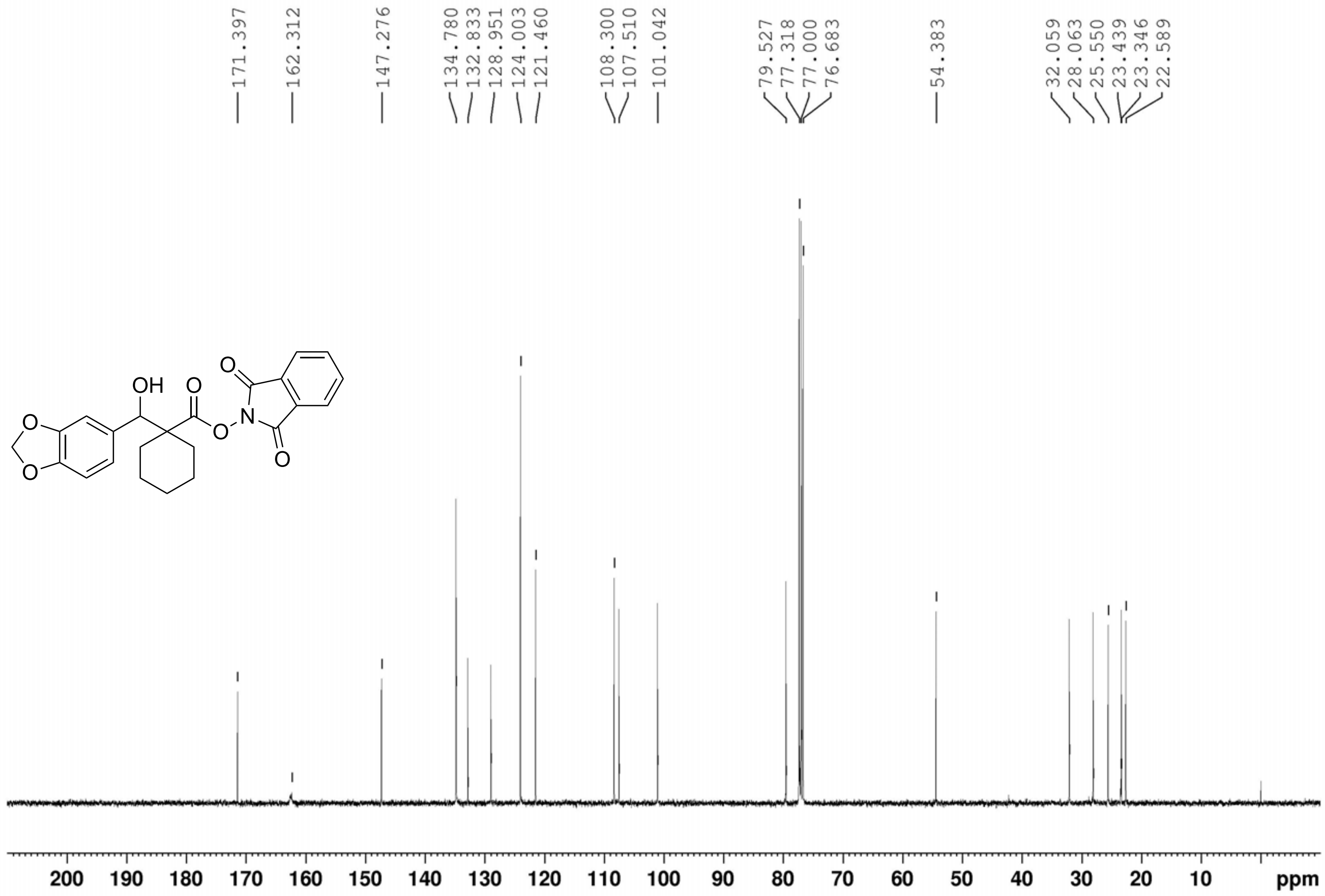

Supplementary Figure 28. ^{13}C NMR spectrum of **1i** (100.6 MHz, $CDCl_3$)

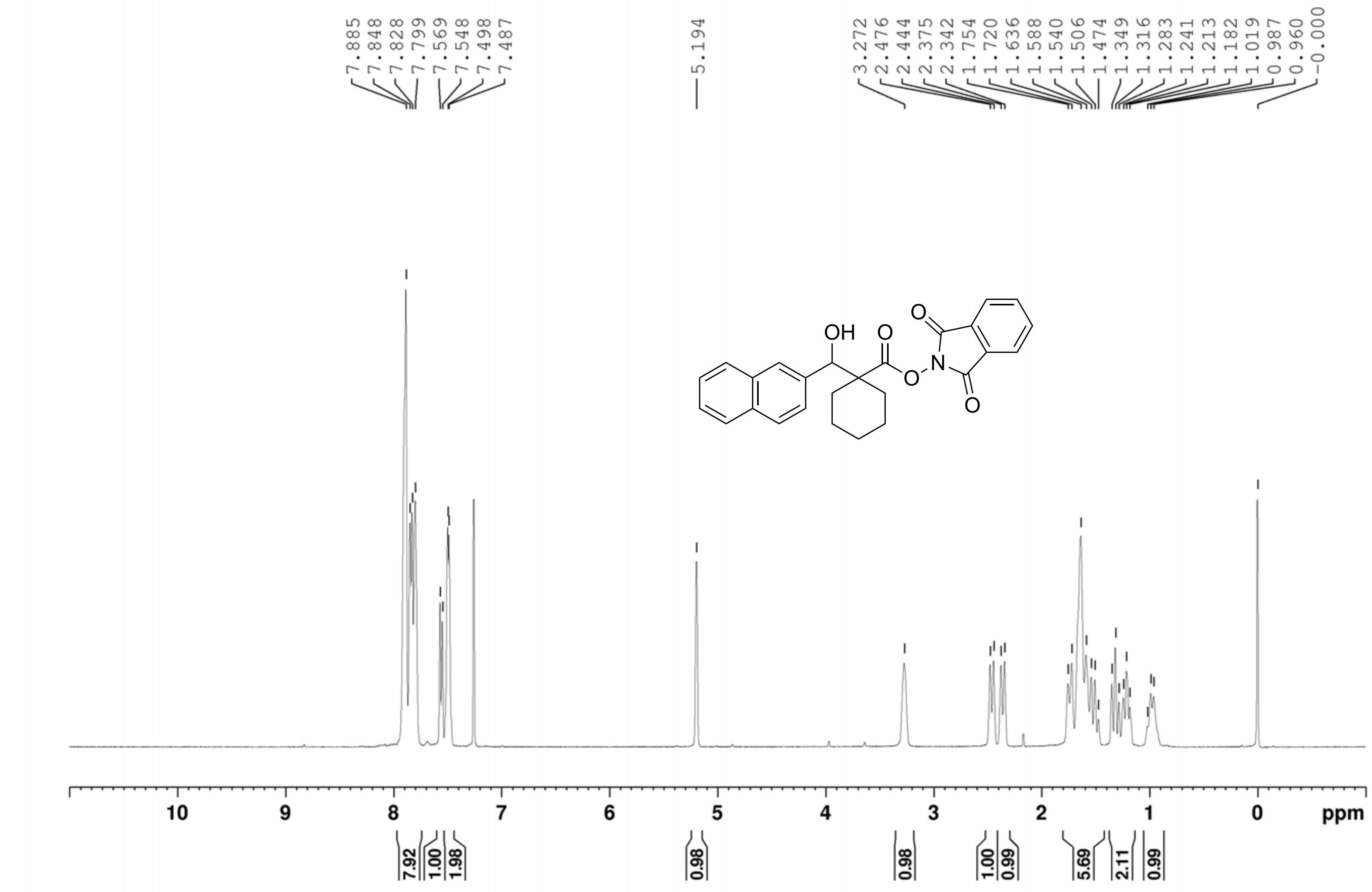

Supplementary Figure 29. ^{1}H NMR spectrum of **1j** (400 MHz, $CDCl_3$)

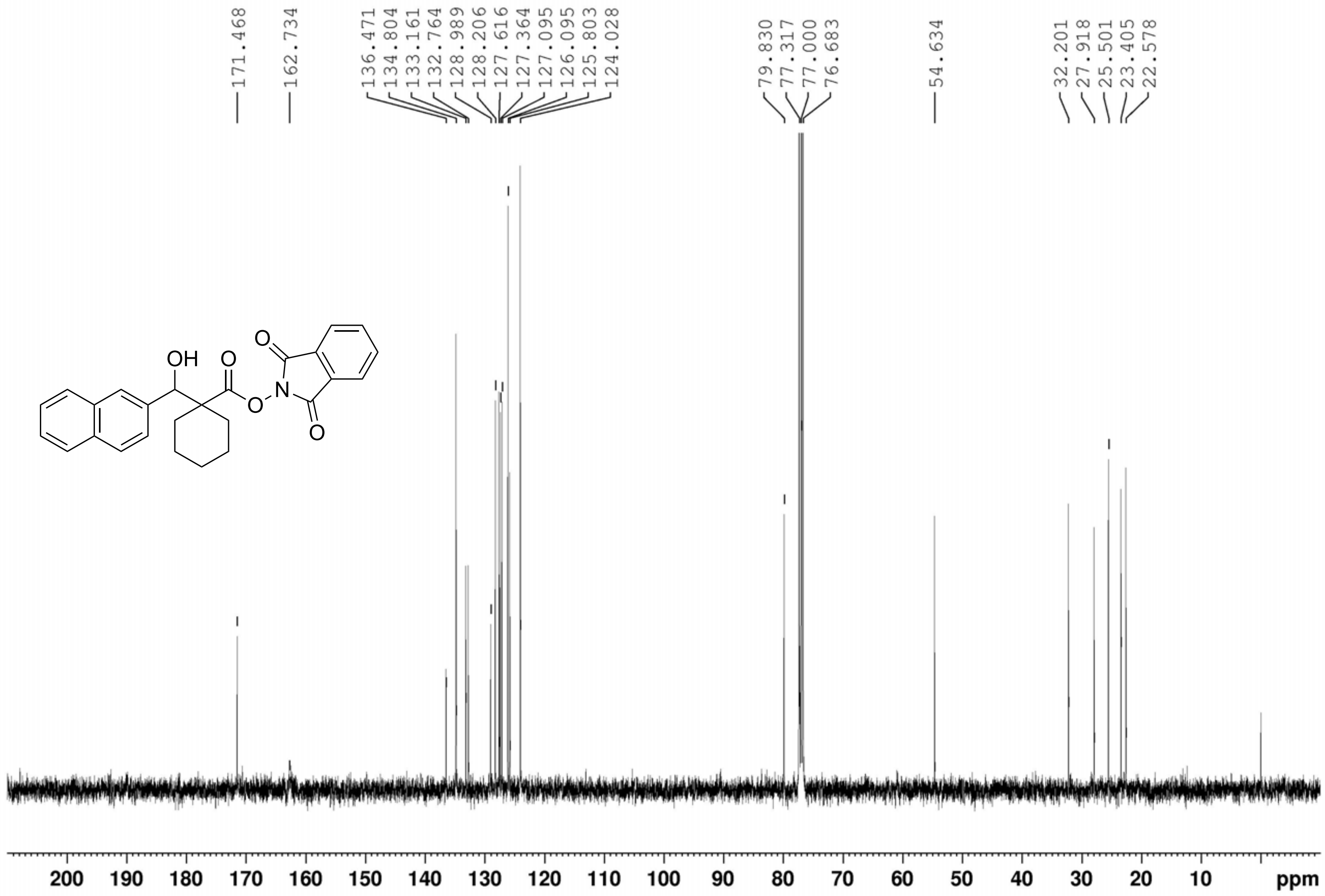

Supplementary Figure 30. ^{13}C NMR spectrum of **1j** (100.6 MHz, $CDCl_3$)

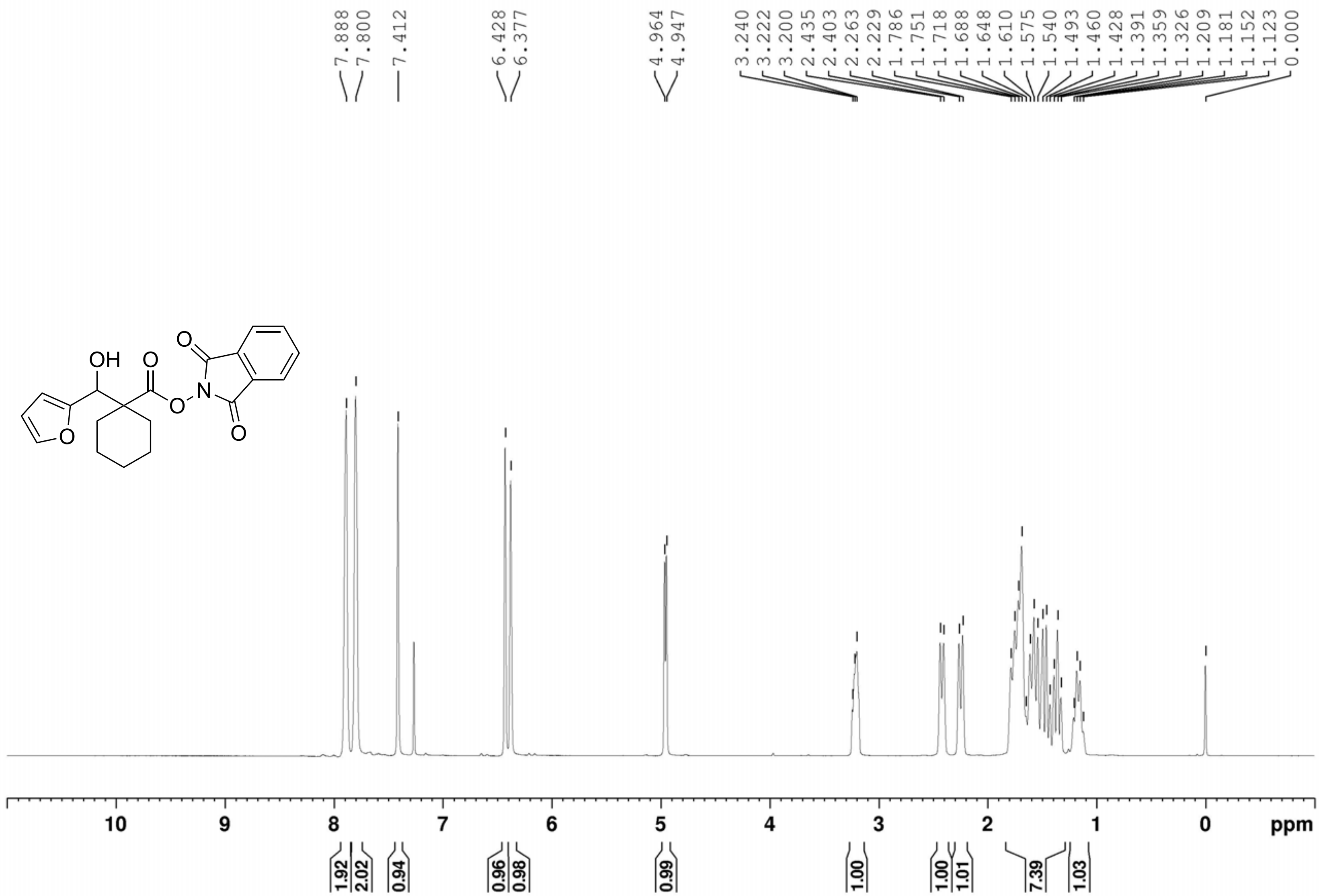

Supplementary Figure 31. ^{1}H NMR spectrum of **1k** (400 MHz, $CDCl_3$)

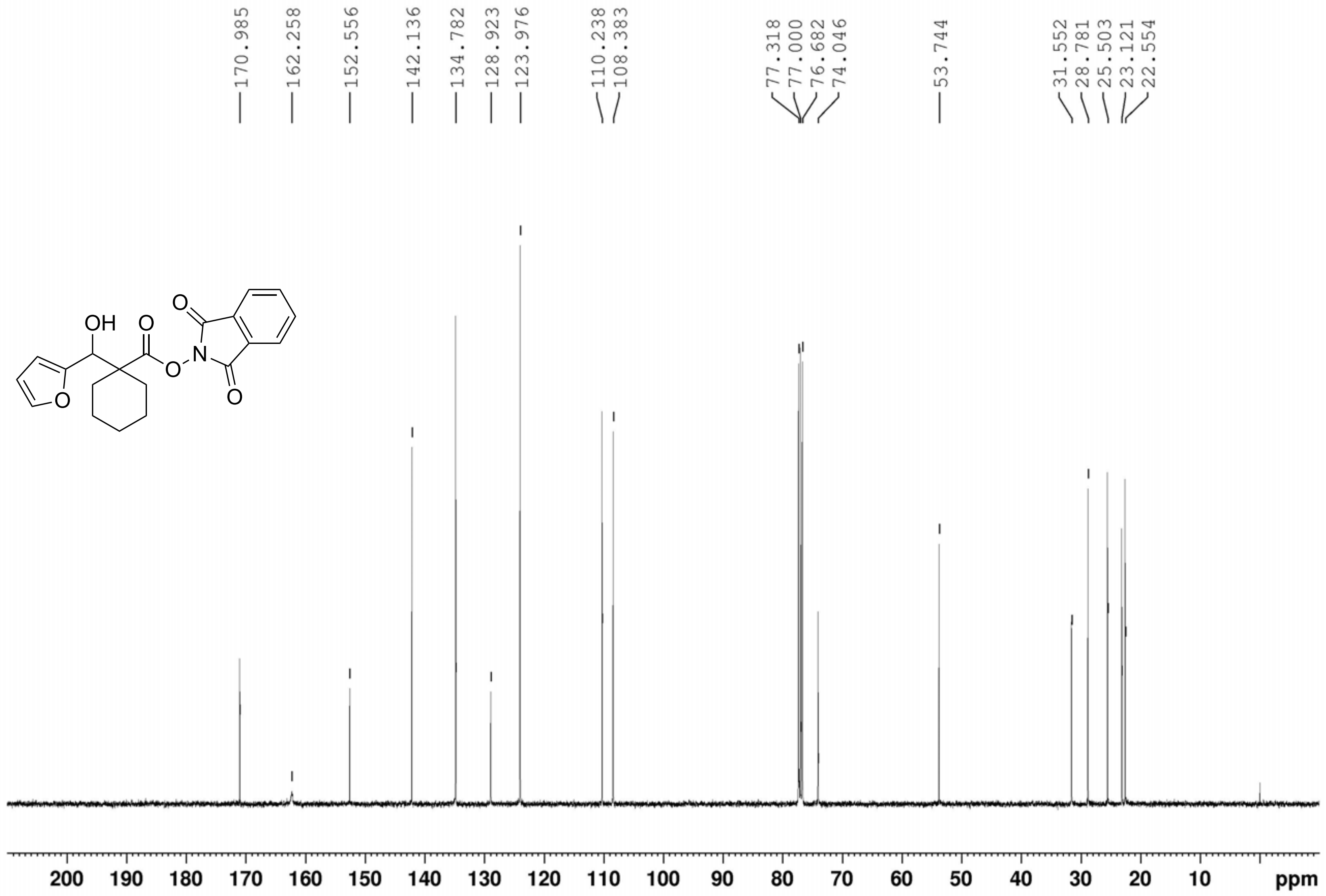

Supplementary Figure 32. ^{13}C NMR spectrum of **1k** (100.6 MHz, $CDCl_3$)

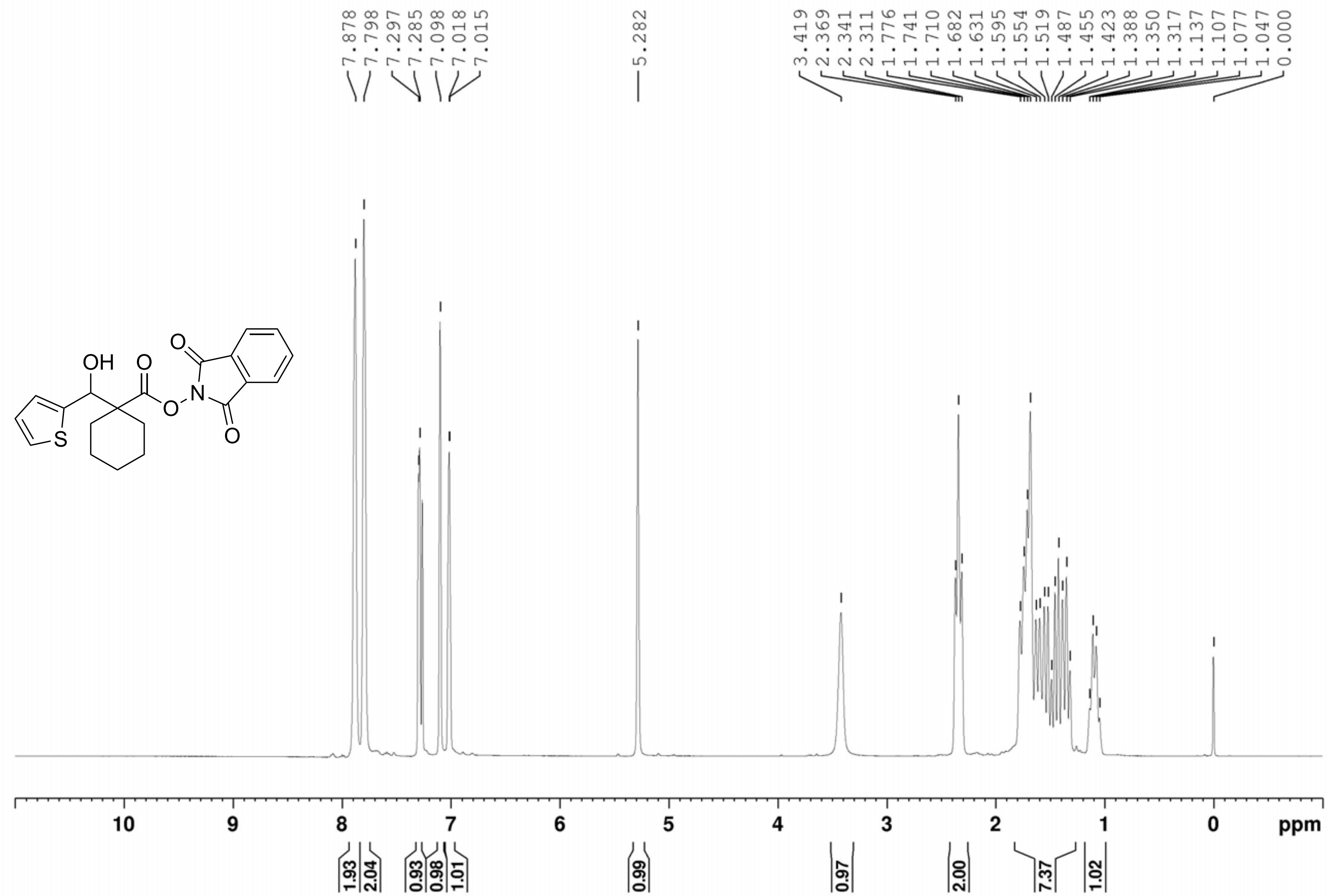

Supplementary Figure 33. ^{1}H NMR spectrum of **1l** (400 MHz, $CDCl_3$)

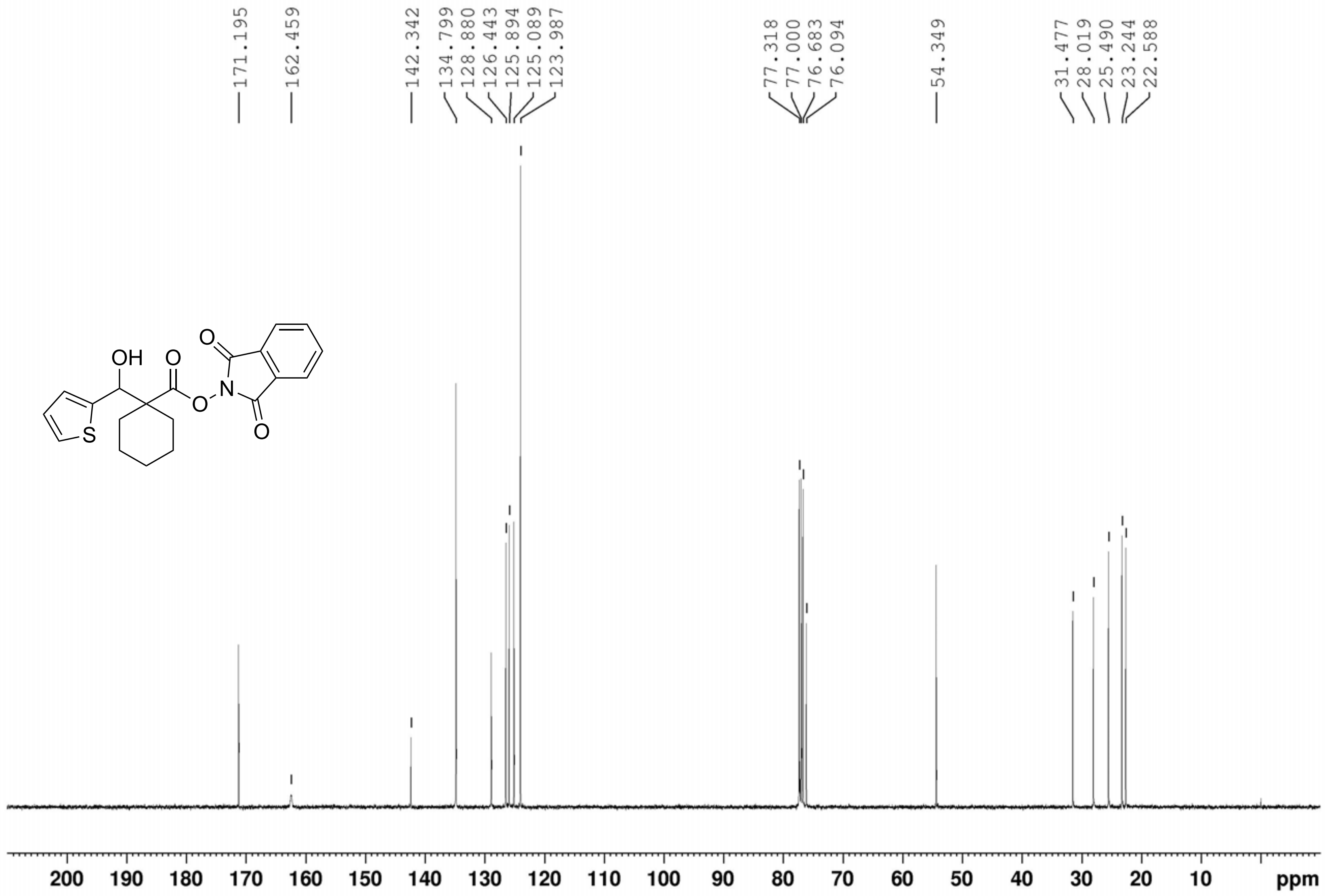

Supplementary Figure 34. ^{13}C NMR spectrum of **1l** (100.6 MHz, $CDCl_3$)

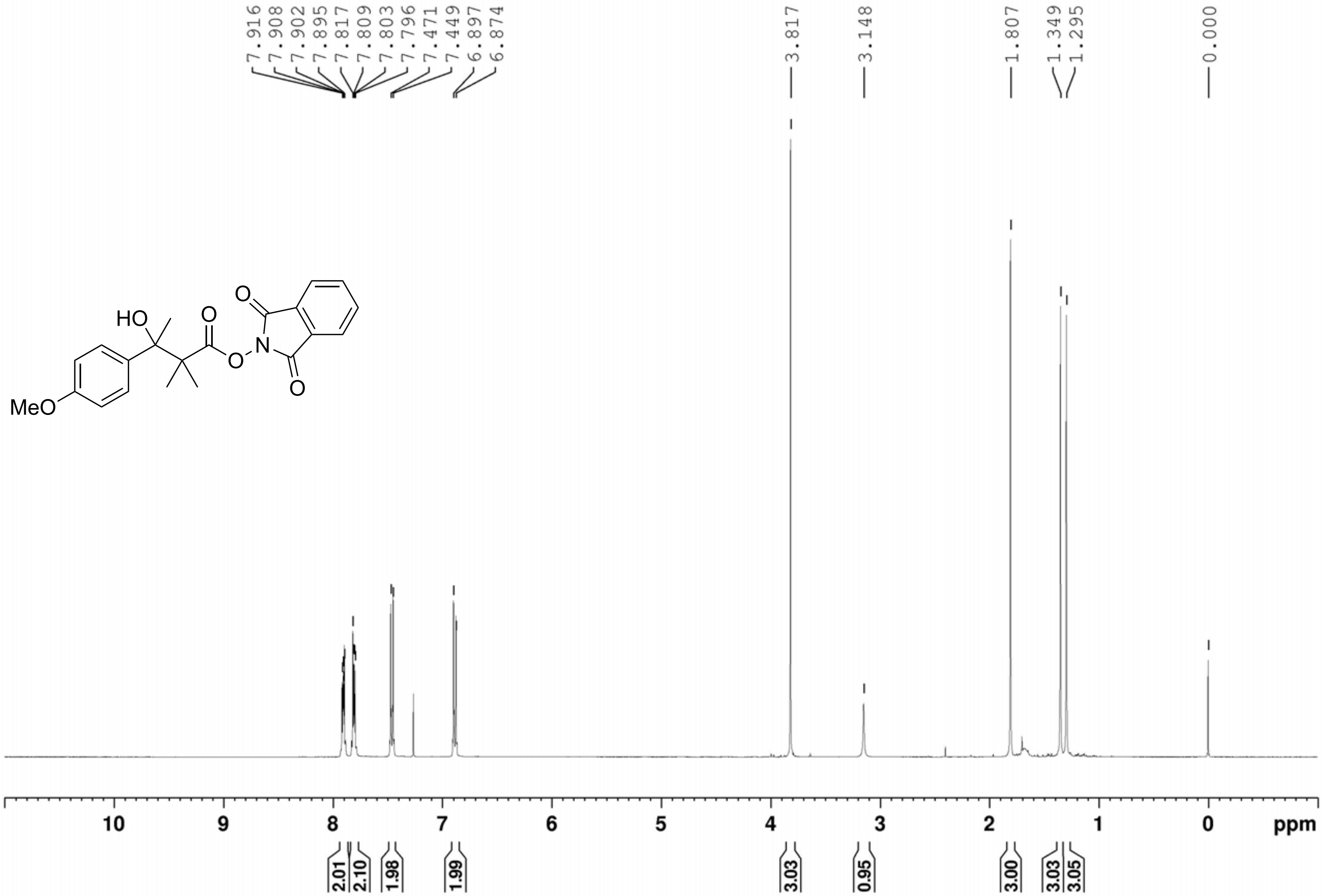

Supplementary Figure 35. ^{1}H NMR spectrum of **1m** (400 MHz, $CDCl_3$)

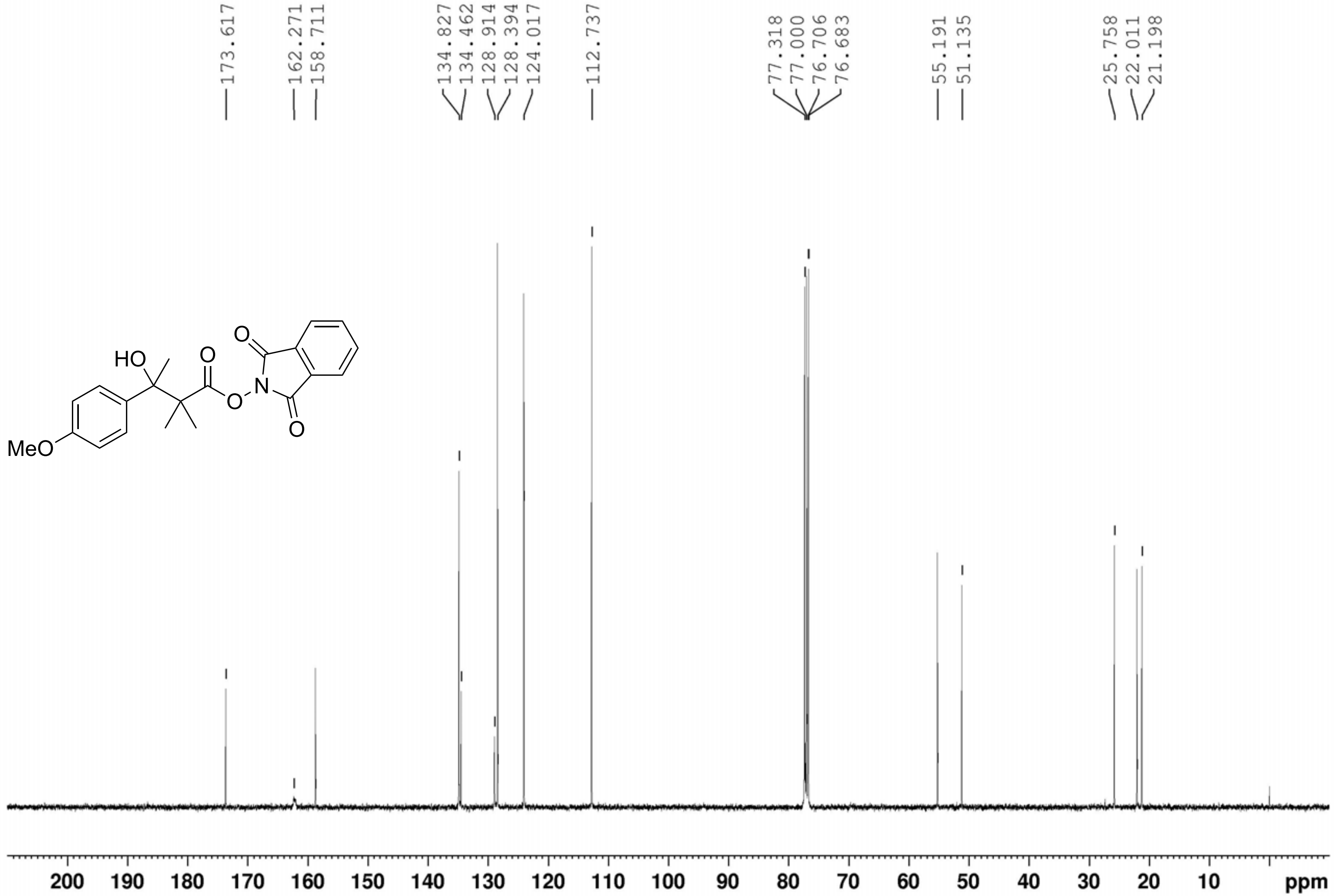

Supplementary Figure 36. ^{13}C NMR spectrum of **1m** (100.6 MHz, $CDCl_3$)

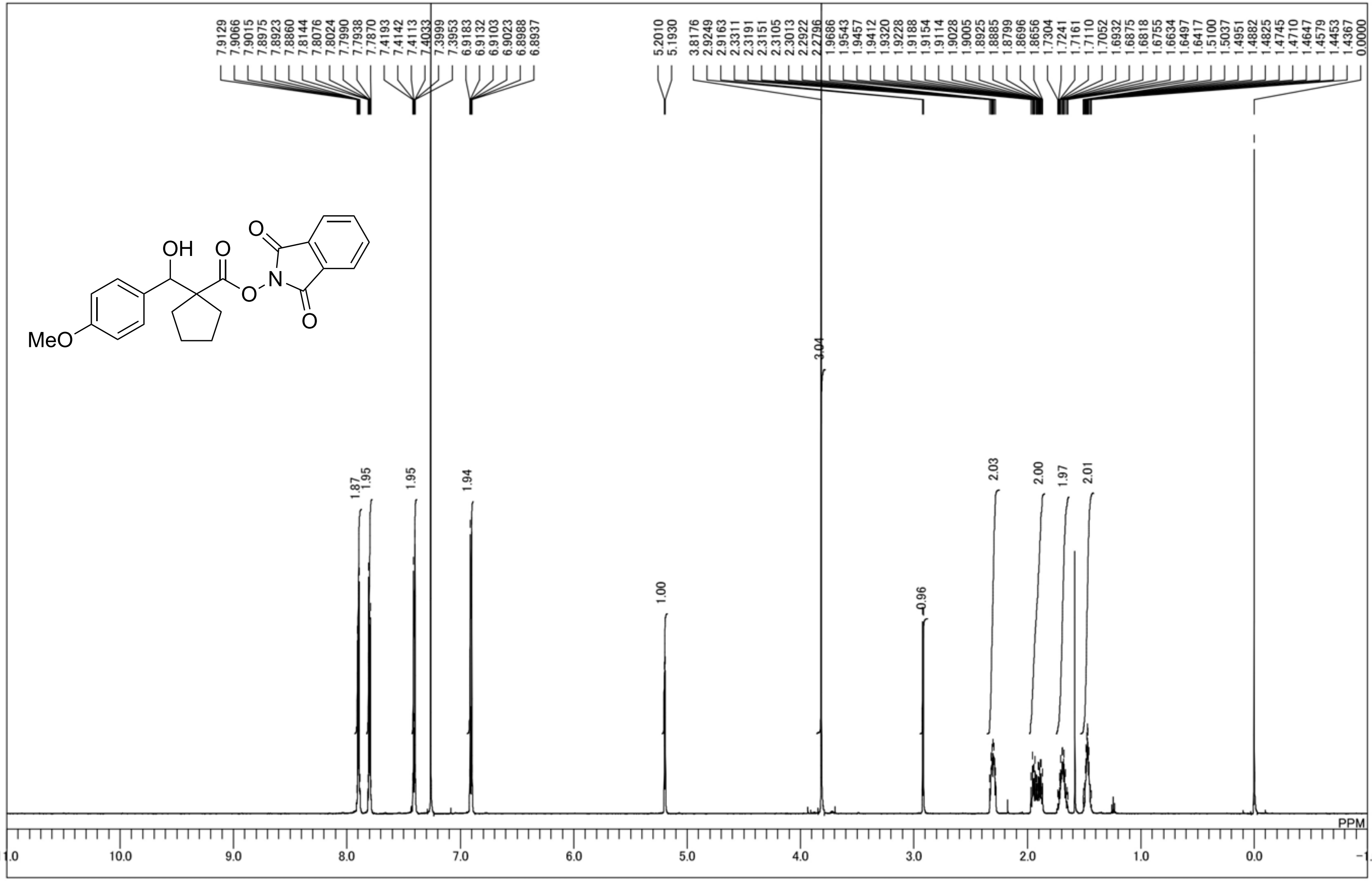

Supplementary Figure 37. ^{1}H NMR spectrum of **1n** (600 MHz, $CDCl_3$)

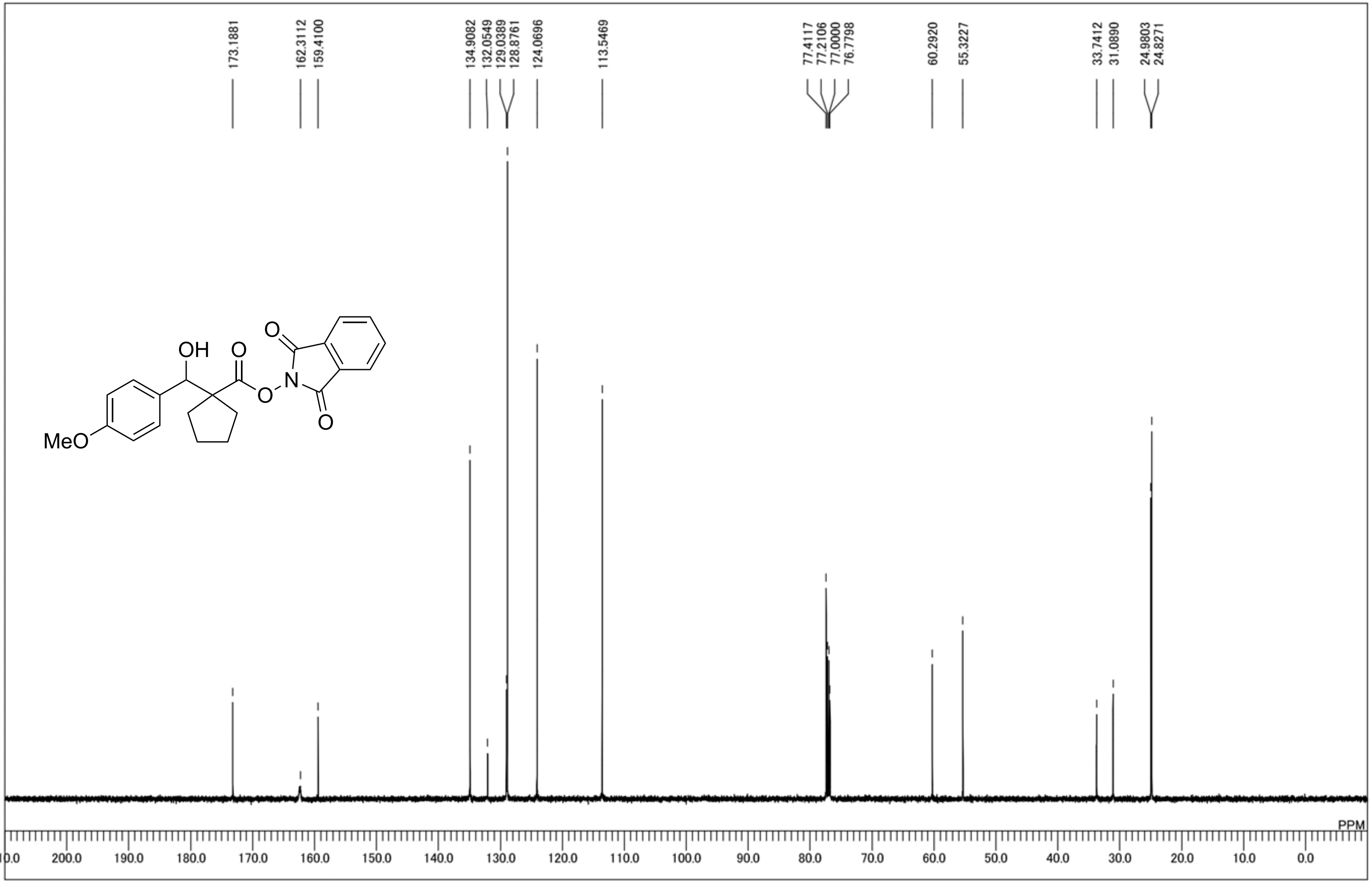

Supplementary Figure 38. ^{13}C NMR spectrum of **1n** (100.6 MHz, $CDCl_3$)

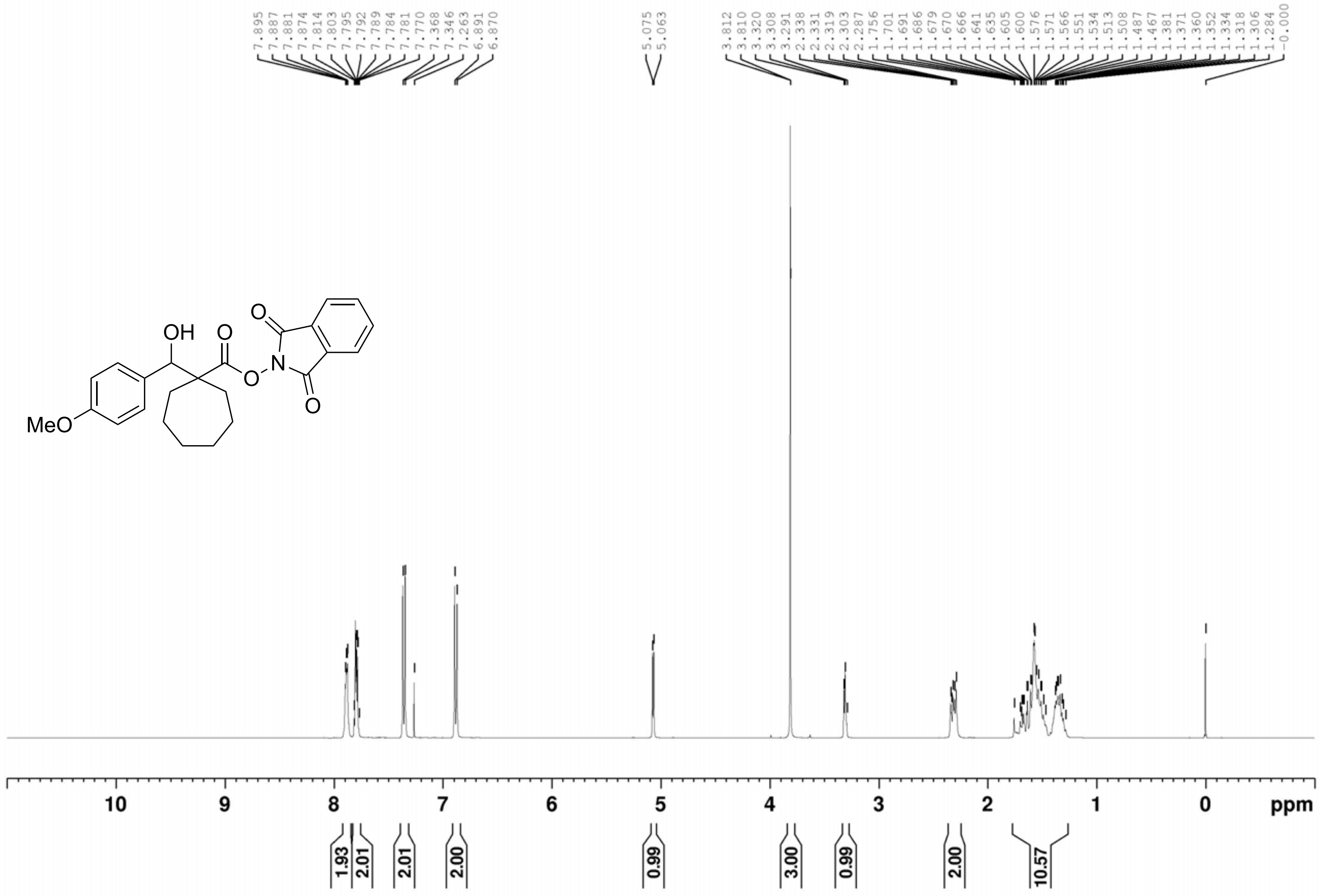

Supplementary Figure 39. ^{1}H NMR spectrum of **1o** (400 MHz, $CDCl_3$)

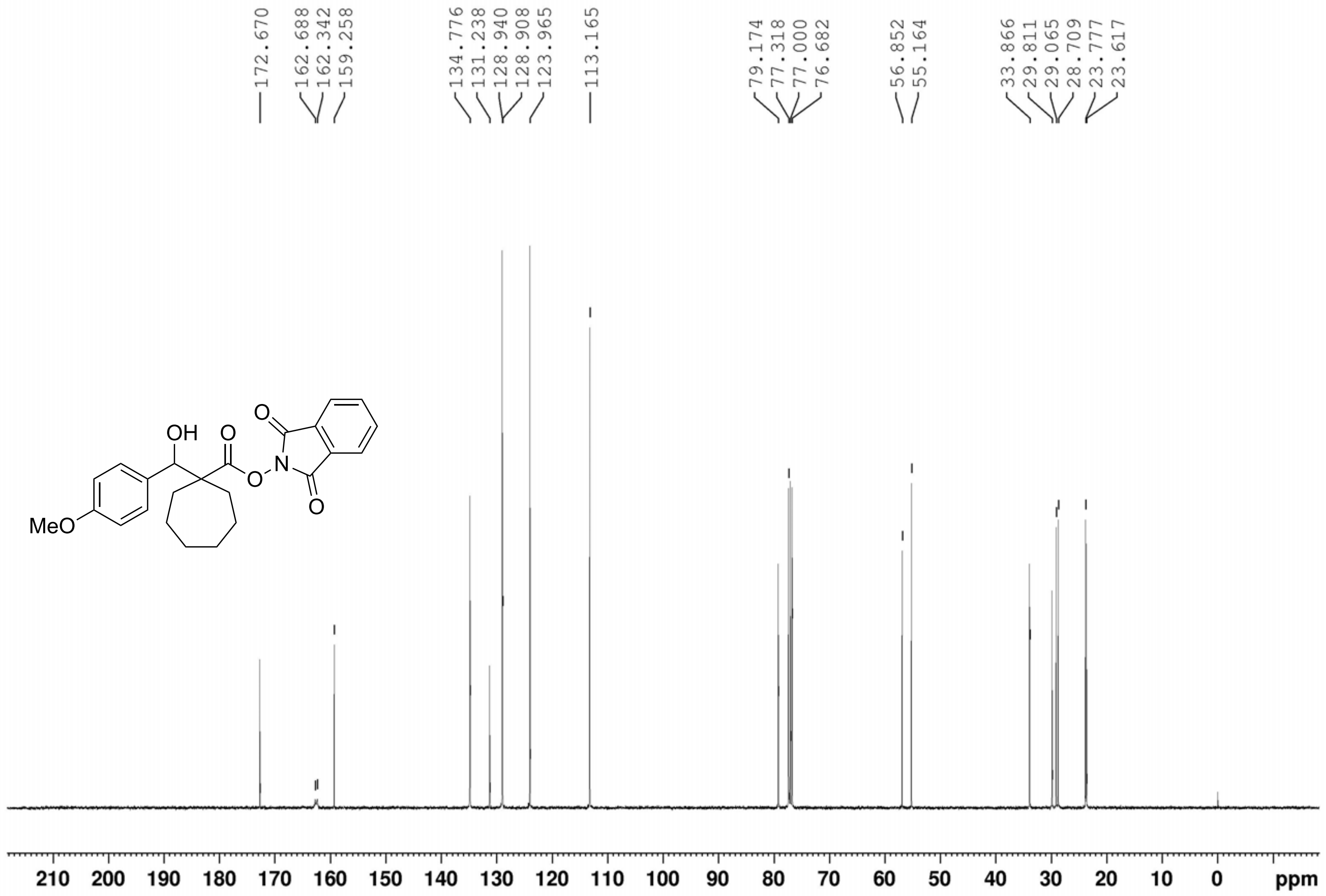

Supplementary Figure 40. ^{13}C NMR spectrum of **1o** (100.6 MHz, $CDCl_3$)

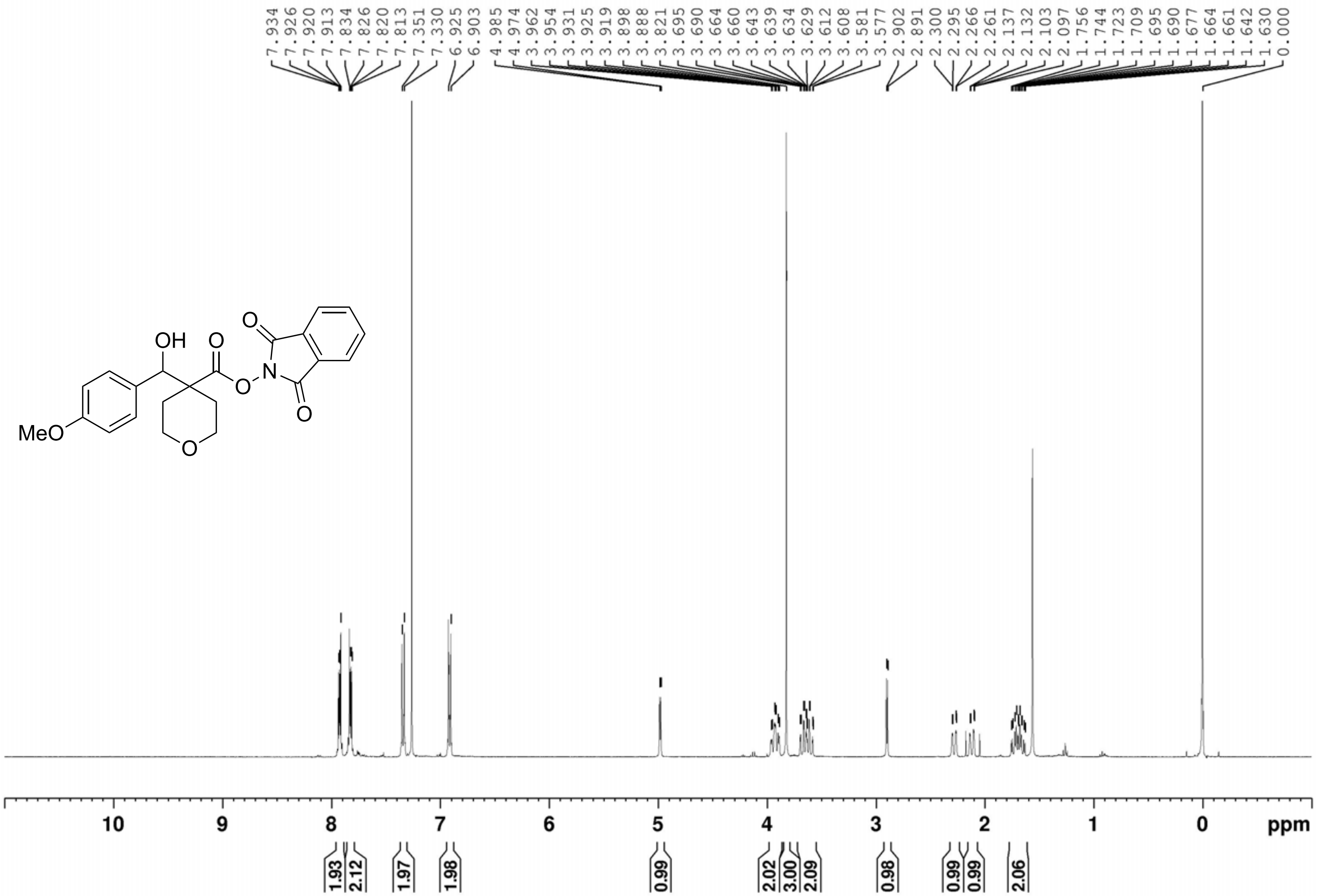

Supplementary Figure 41. ^{1}H NMR spectrum of **1p** (400 MHz, $CDCl_3$)

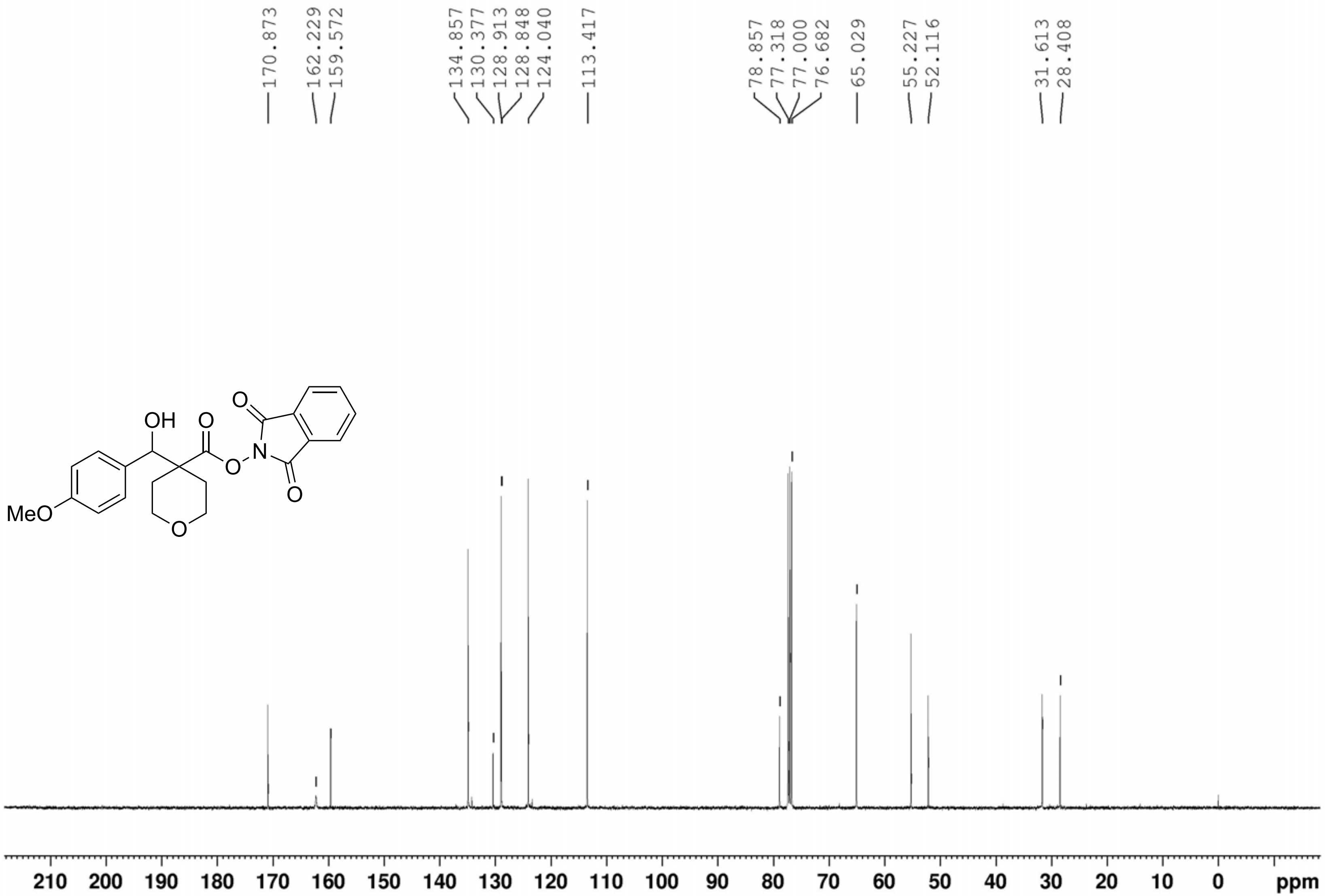

Supplementary Figure 42. ^{13}C NMR spectrum of **1p** (100.6 MHz, $CDCl_3$)

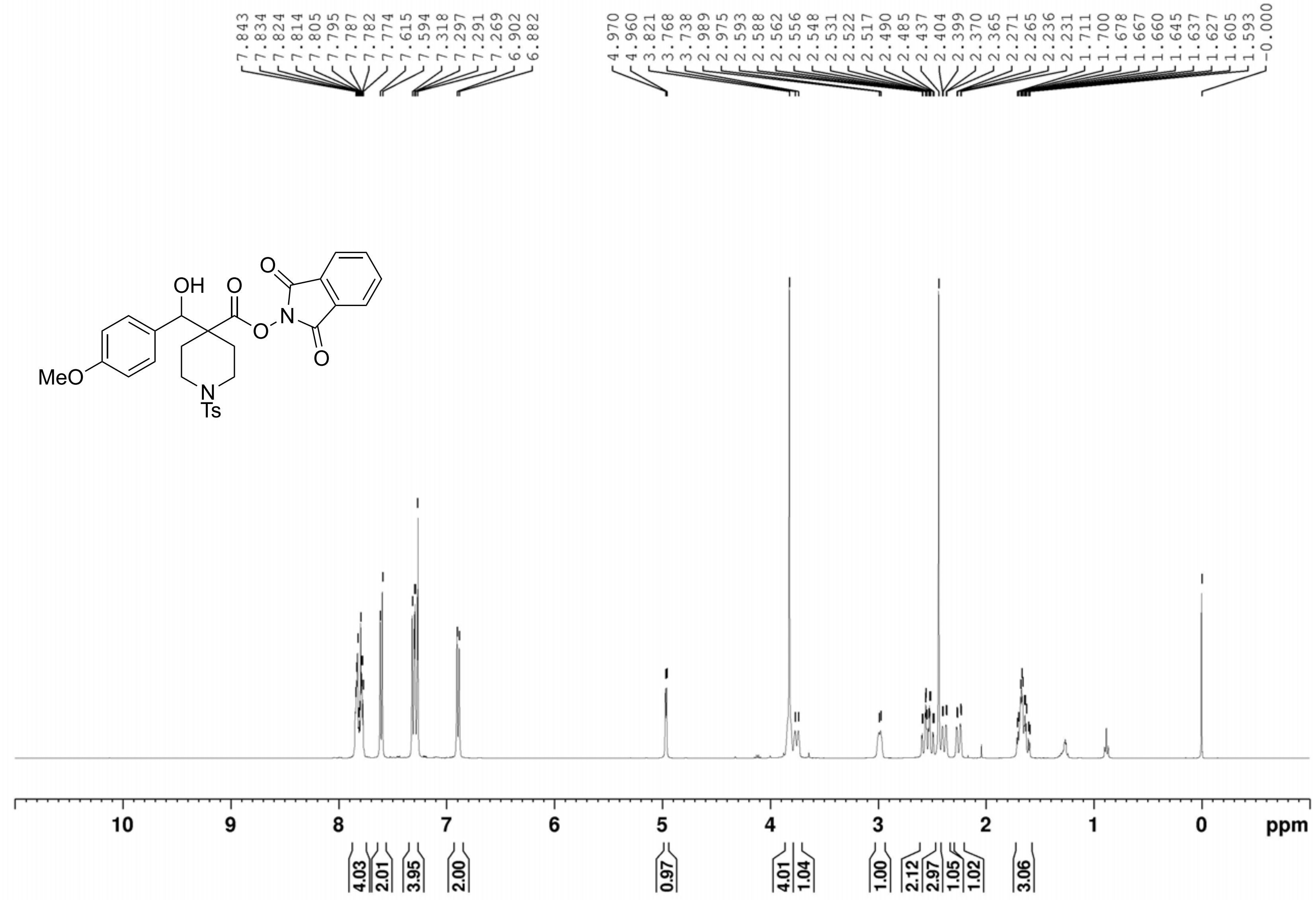

Supplementary Figure 43. ^{1}H NMR spectrum of **1q** (400 MHz, $CDCl_3$)

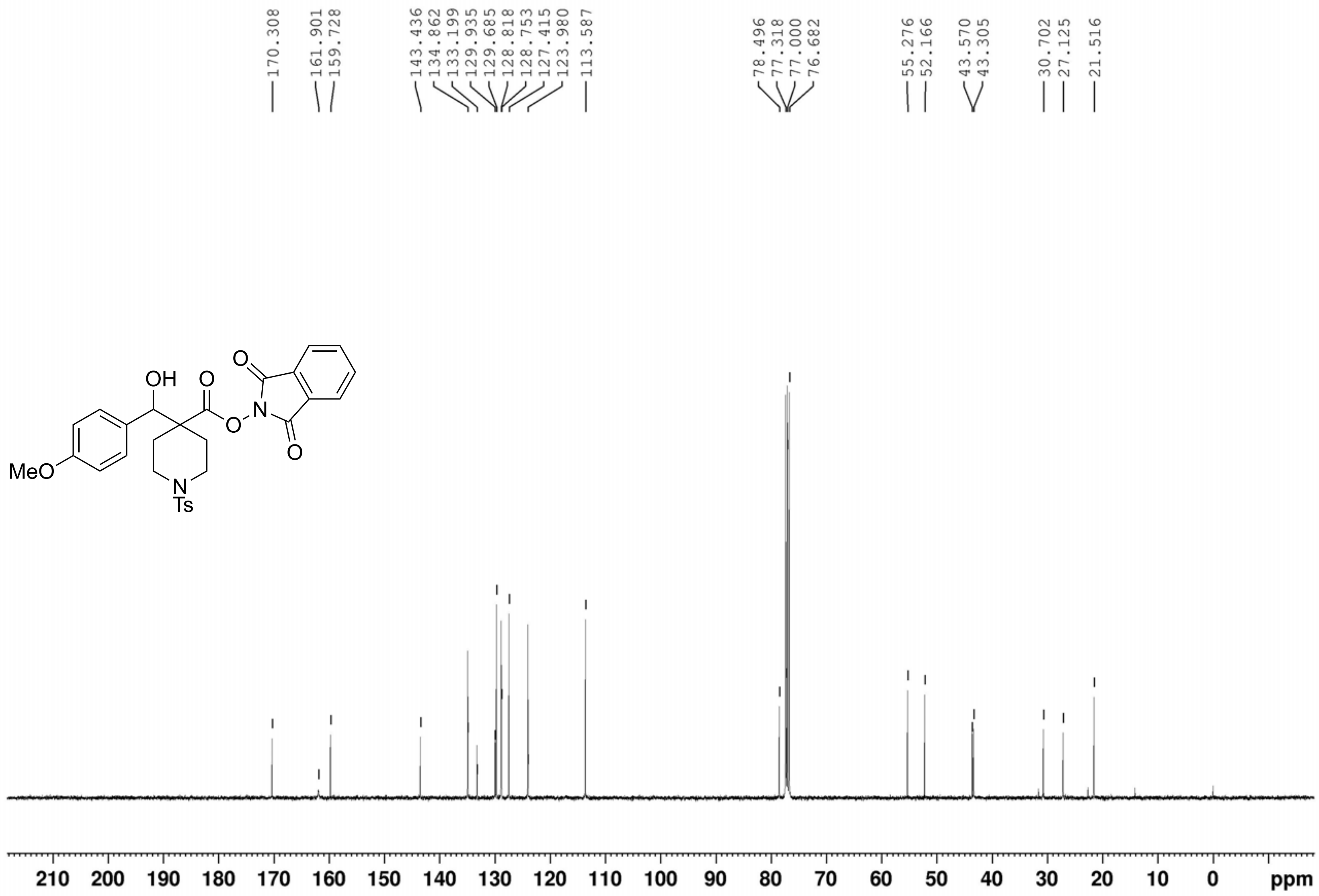

Supplementary Figure 44. ^{13}C NMR spectrum of **1q** (100.6 MHz, $CDCl_3$)

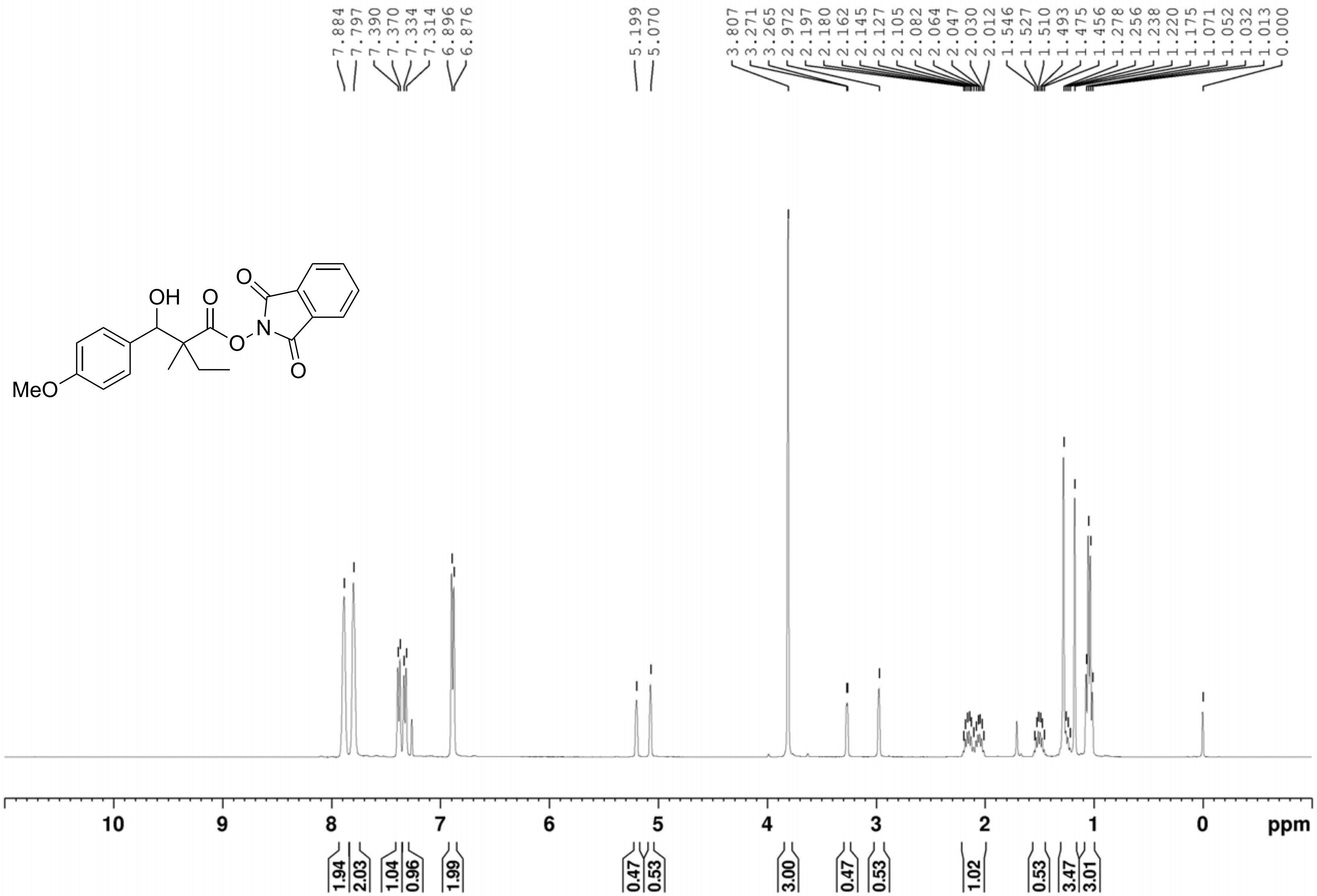

Supplementary Figure 45. ^{1}H NMR spectrum of **1r** (400 MHz, $CDCl_3$)

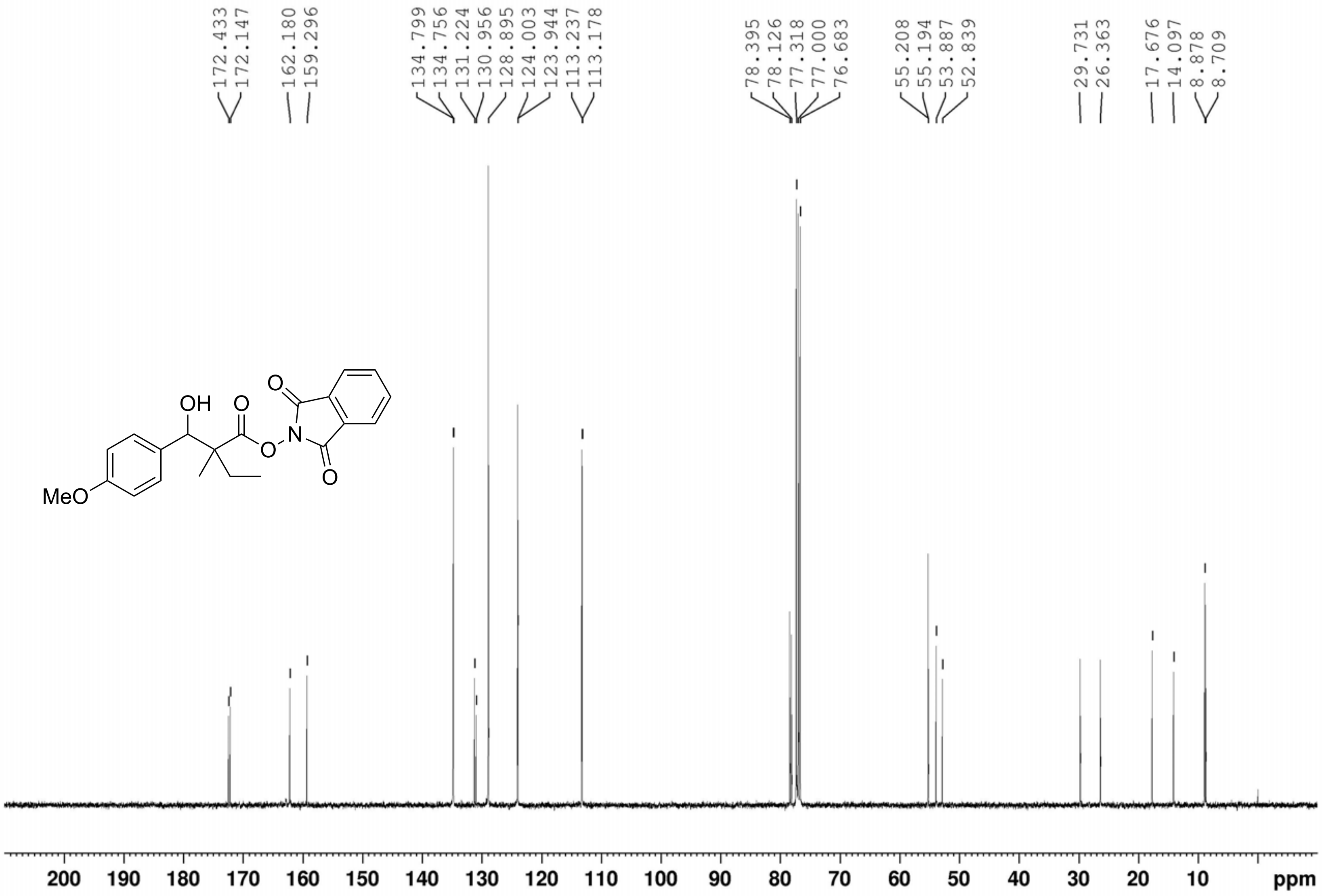

Supplementary Figure 46. ^{13}C NMR spectrum of **1r** (100.6 MHz, $CDCl_3$)

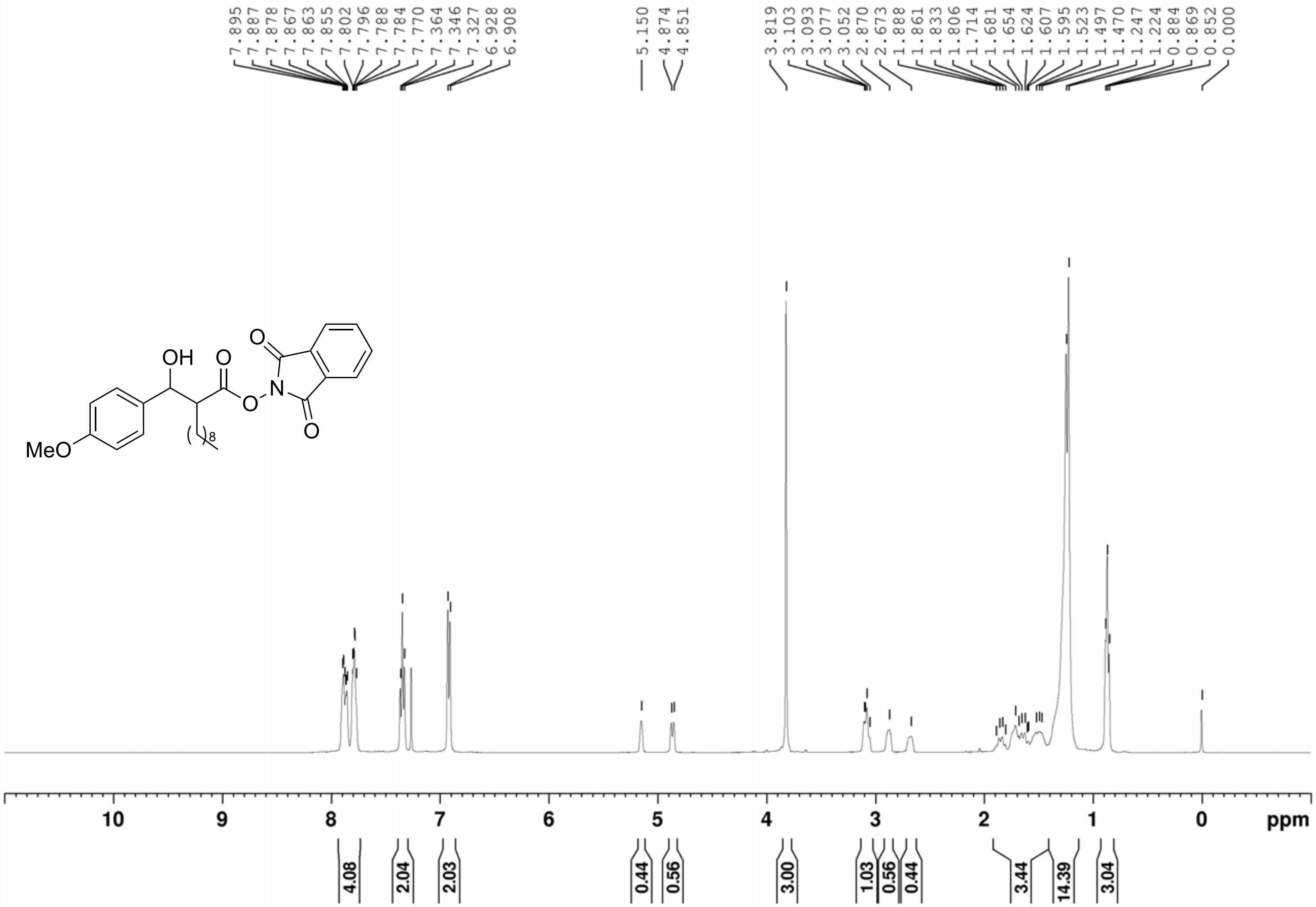

Supplementary Figure 47. ^{1}H NMR spectrum of **1s** (400 MHz, $CDCl_3$)

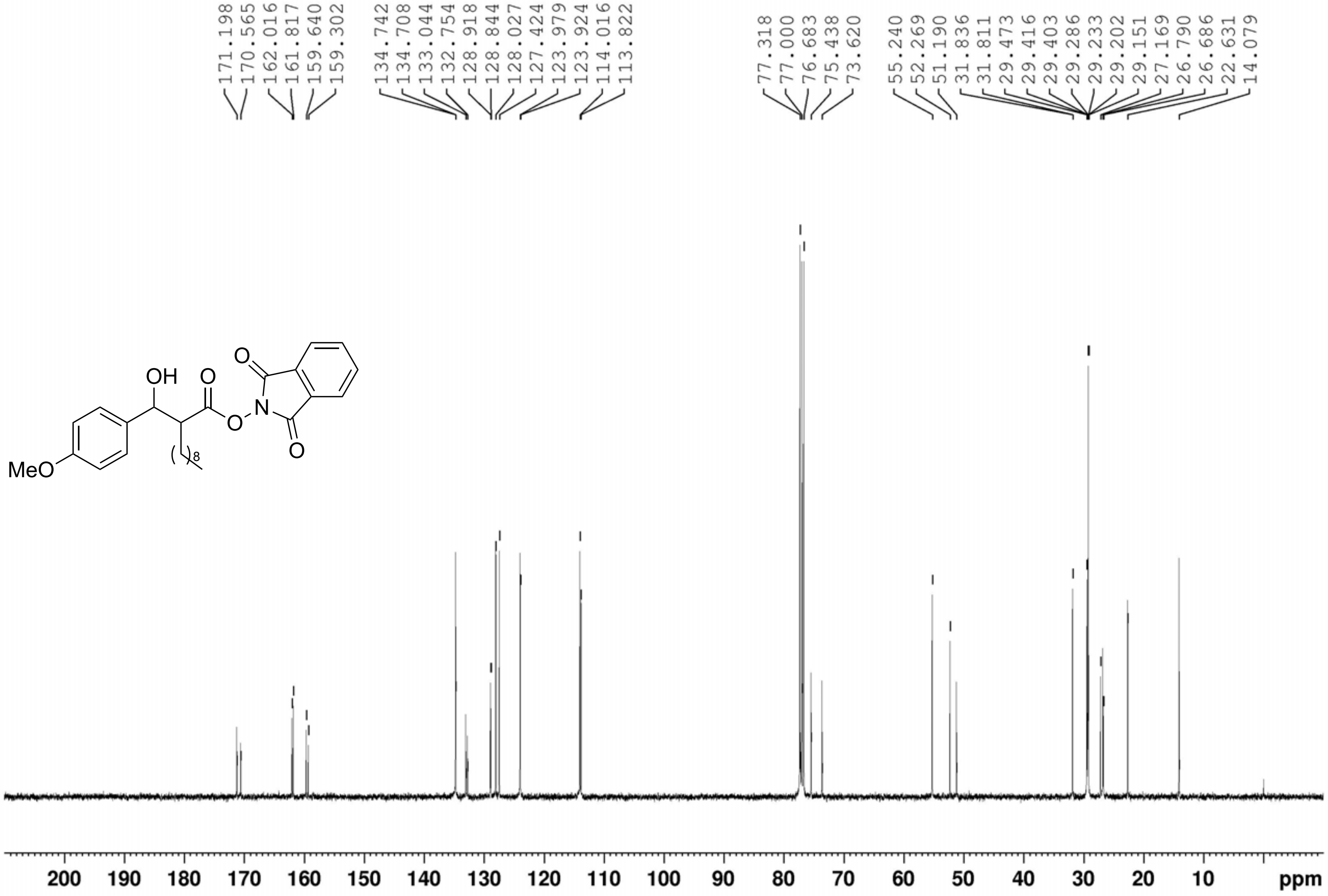

Supplementary Figure 48. ^{13}C NMR spectrum of **1s** (100.6 MHz, $CDCl_3$)

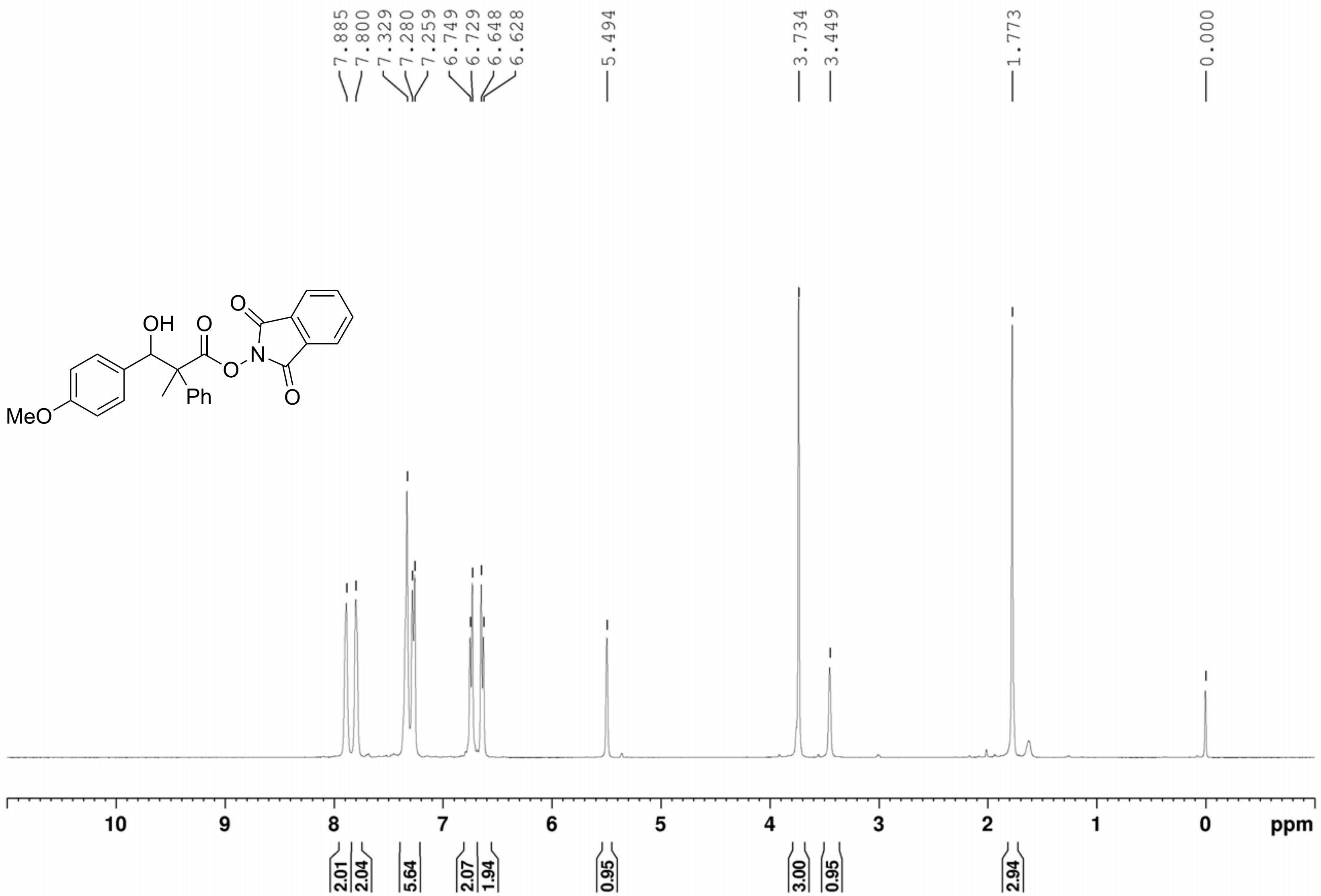

Supplementary Figure 49. ^{1}H NMR spectrum of **1t** (400 MHz, $CDCl_3$)

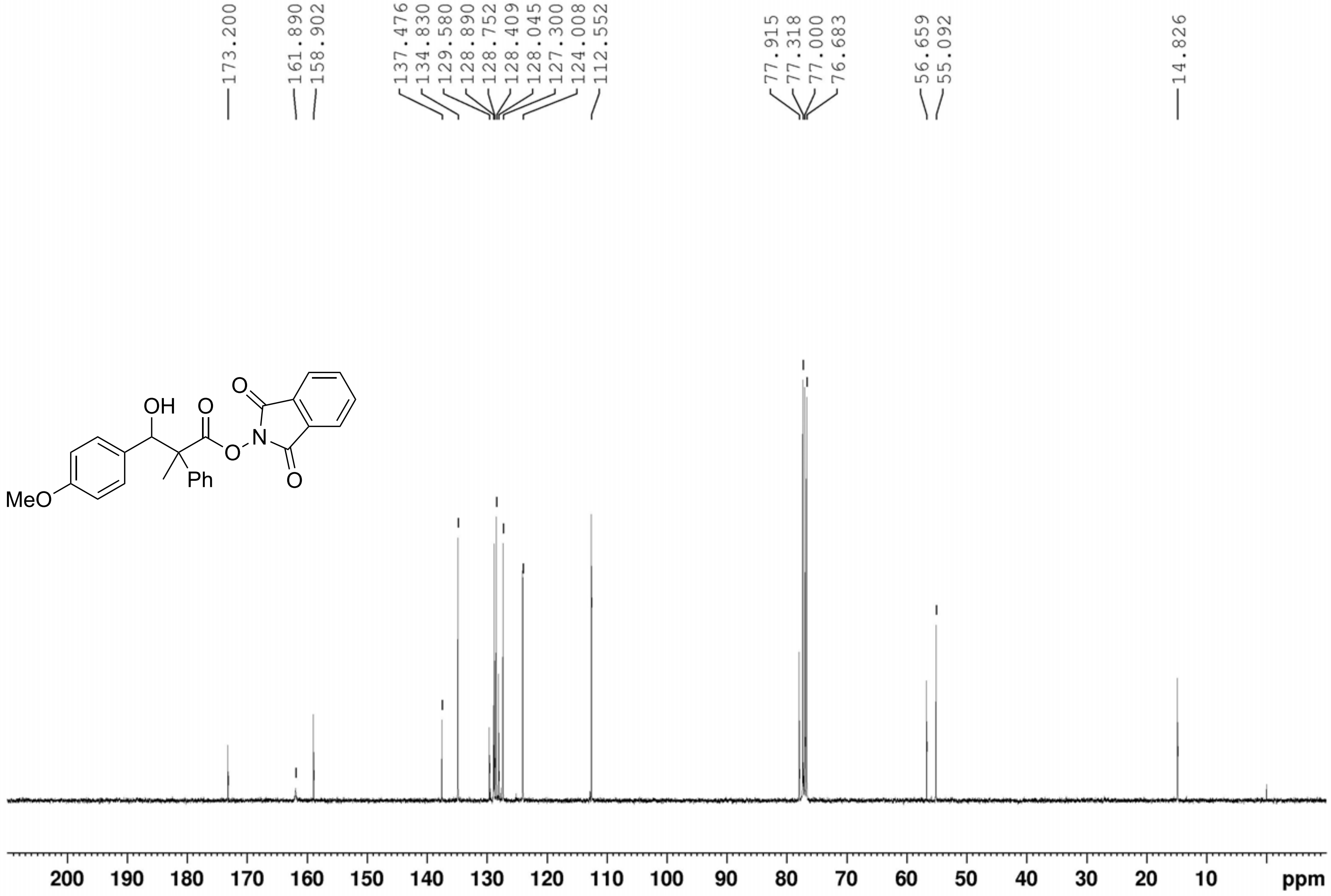

Supplementary Figure 50. ^{13}C NMR spectrum of **1t** (100.6 MHz, $CDCl_3$)

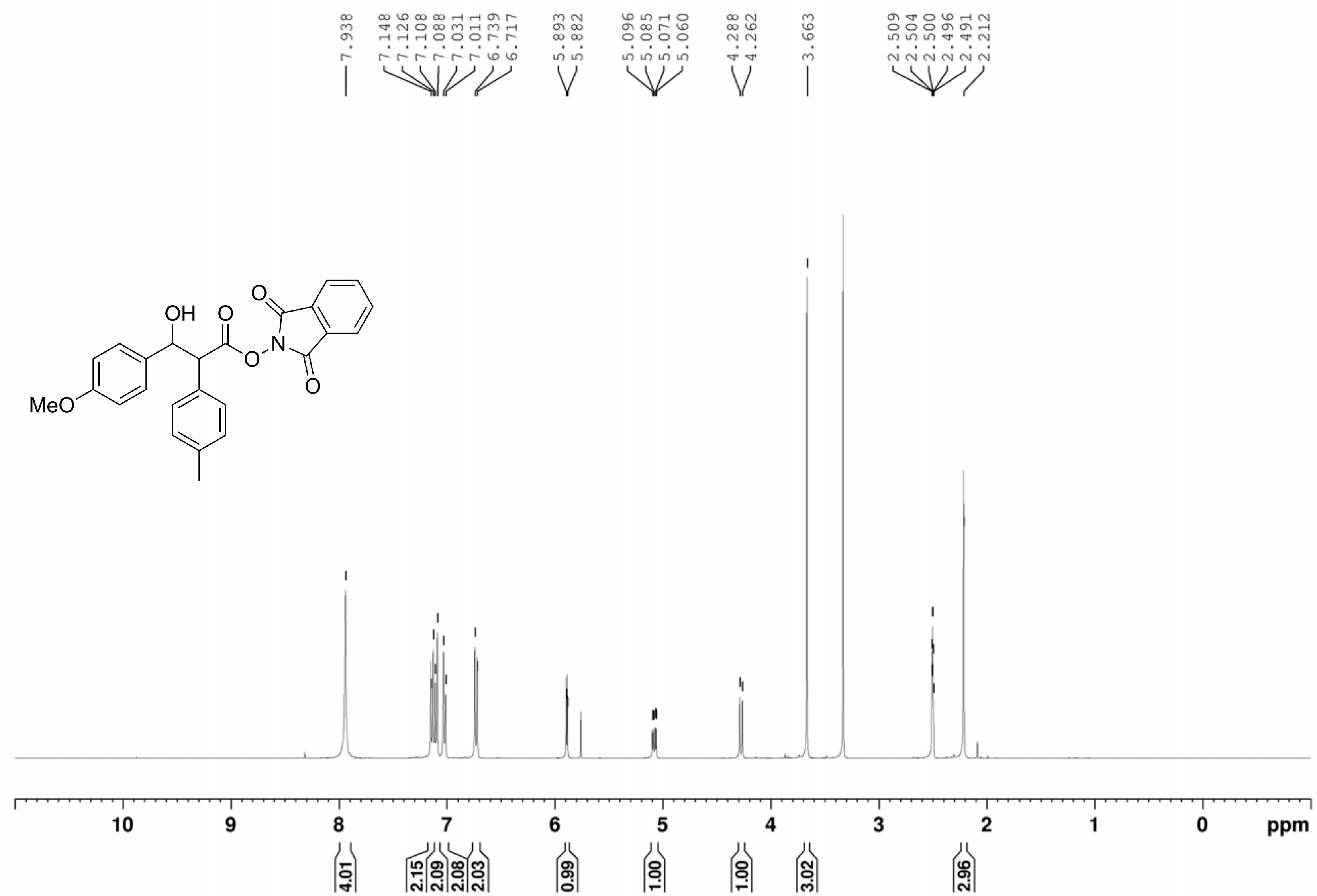

Supplementary Figure 51. ^{1}H NMR spectrum of **1u** (400 MHz, DMSO)

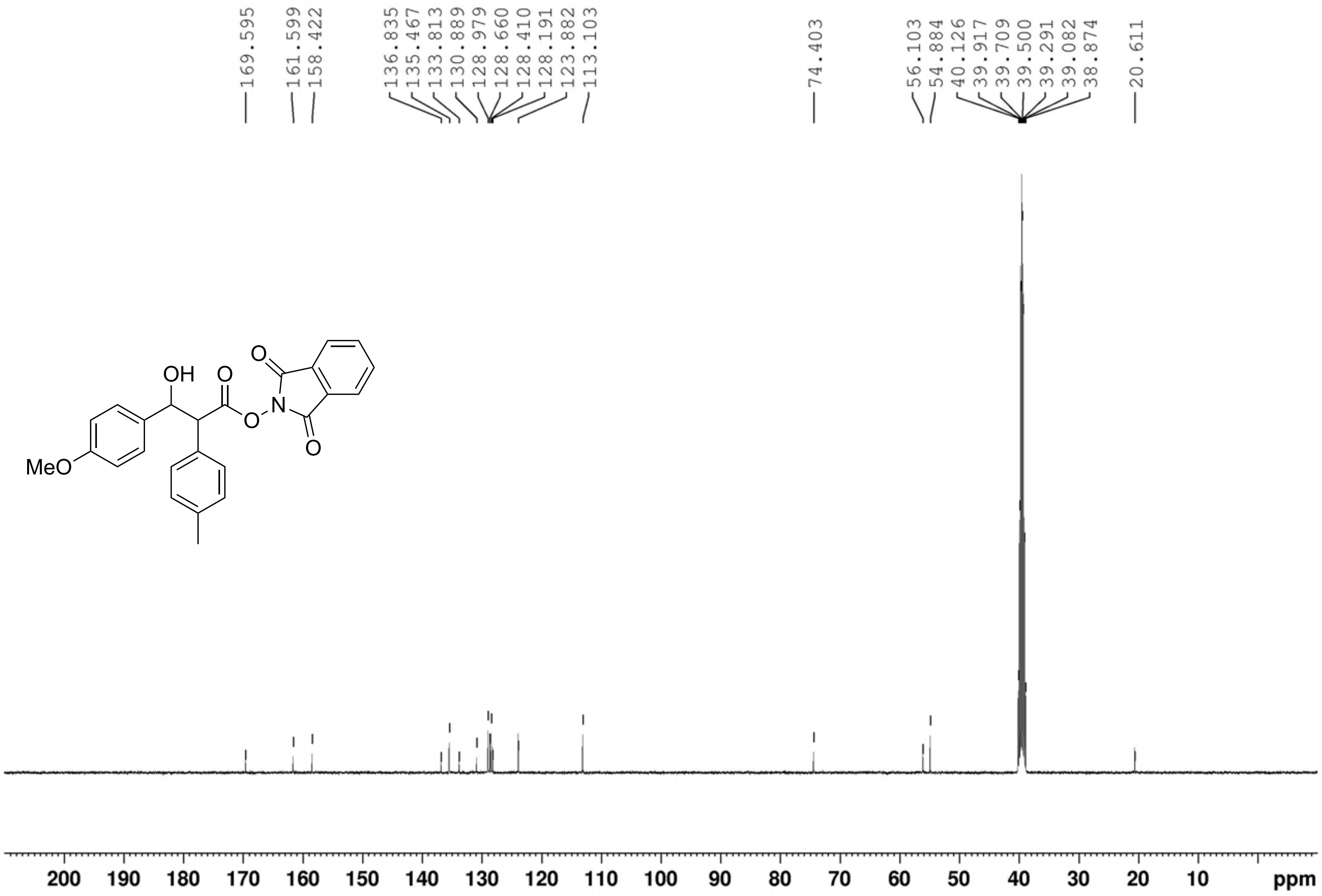

Supplementary Figure 52. ^{13}C NMR spectrum of **1u** (100.6 MHz, DMSO)

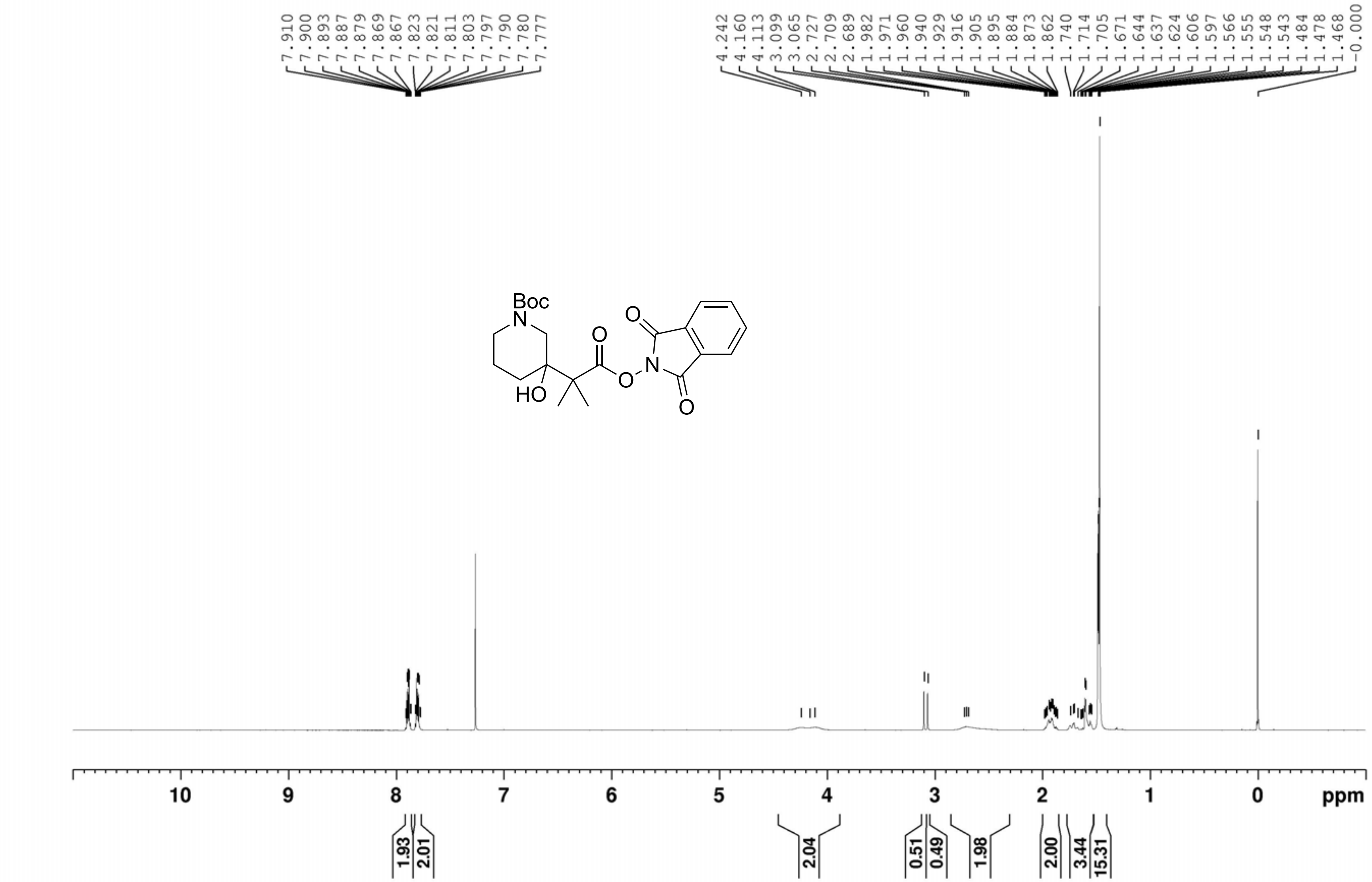

Supplementary Figure 53. ^{1}H NMR spectrum of **1v** (400 MHz, $CDCl_3$)

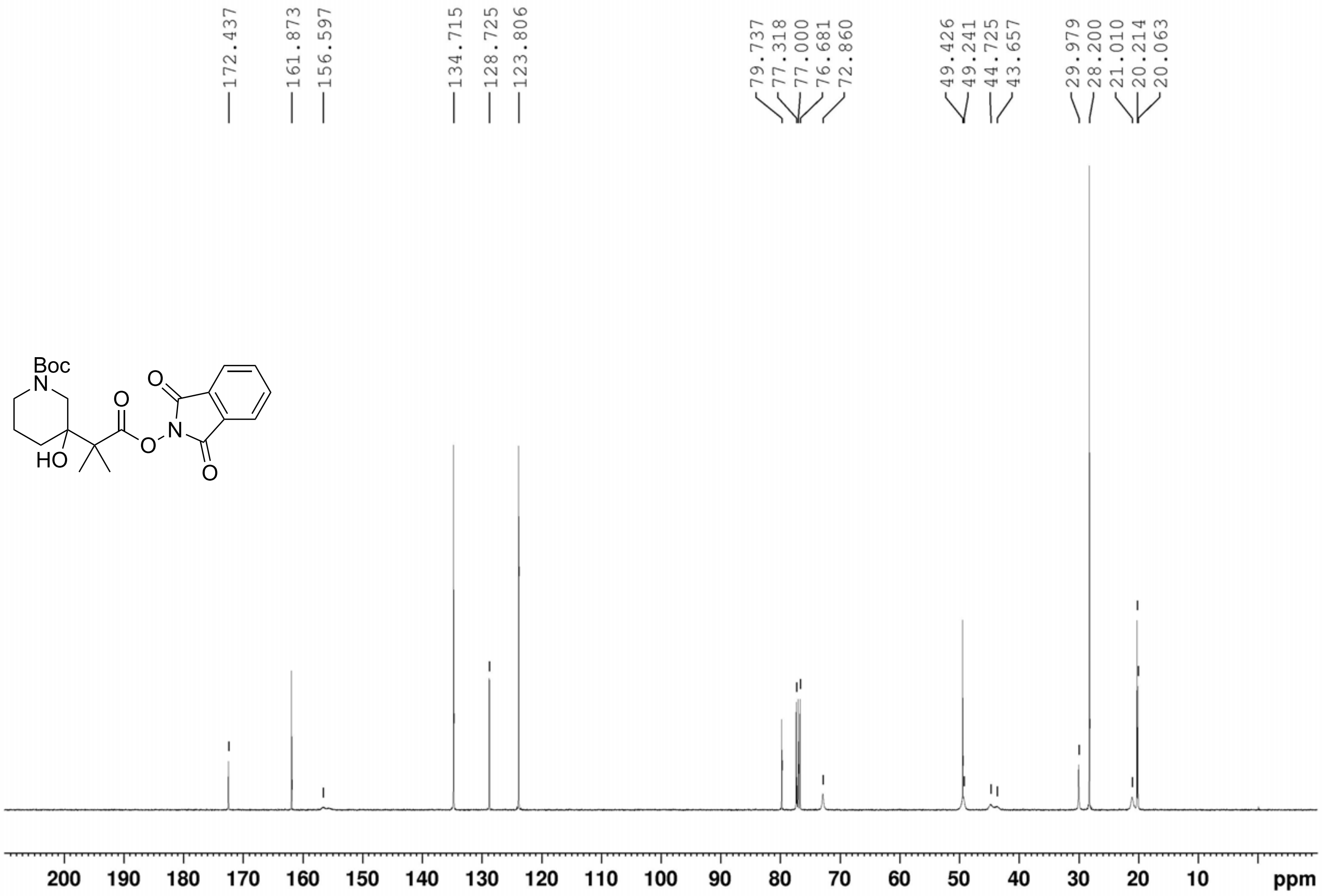

Supplementary Figure 54. ^{13}C NMR spectrum of **1v** (100.6 MHz, $CDCl_3$)

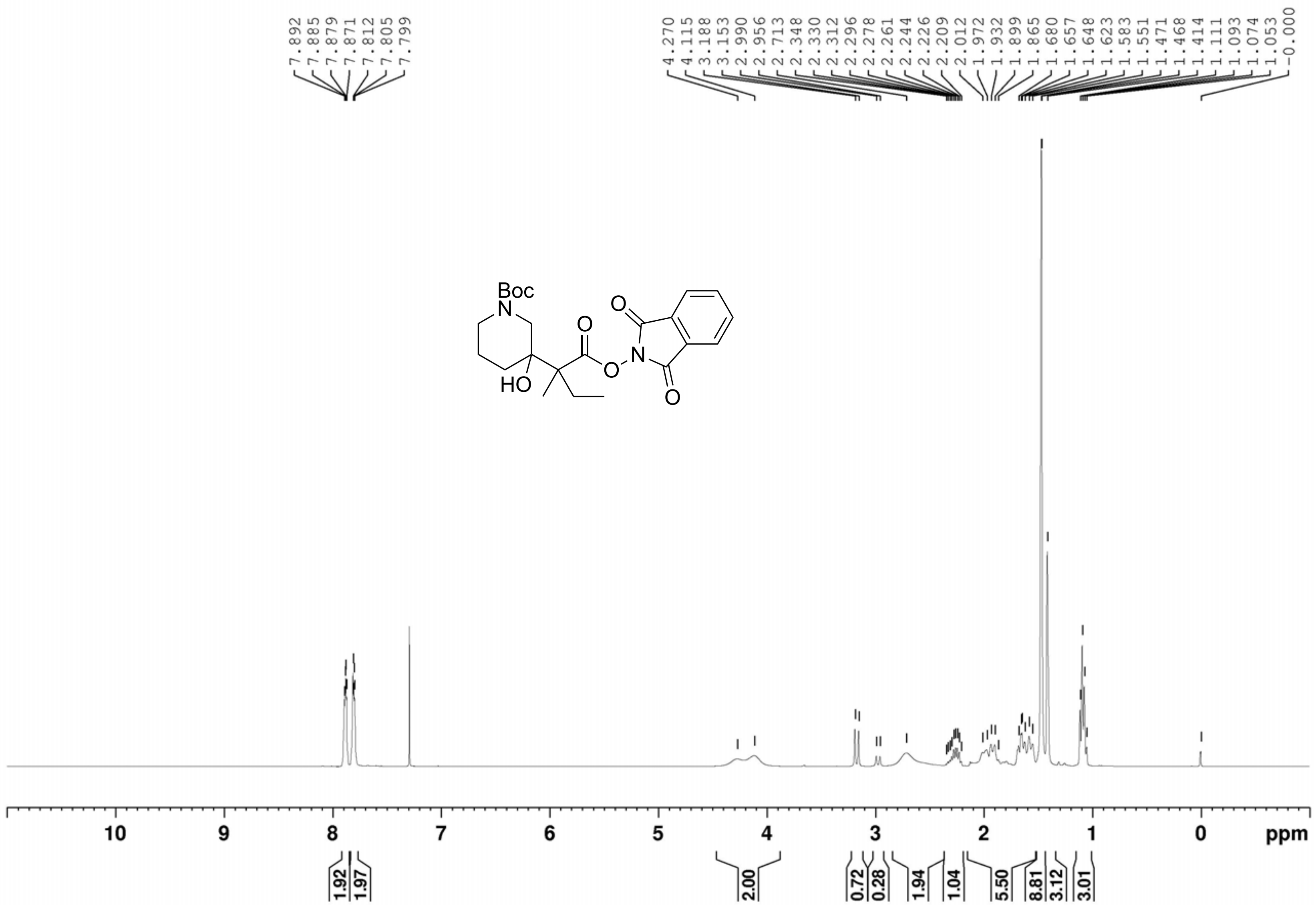

Supplementary Figure 55. ^{1}H NMR spectrum of **1w** (400 MHz, $CDCl_3$)

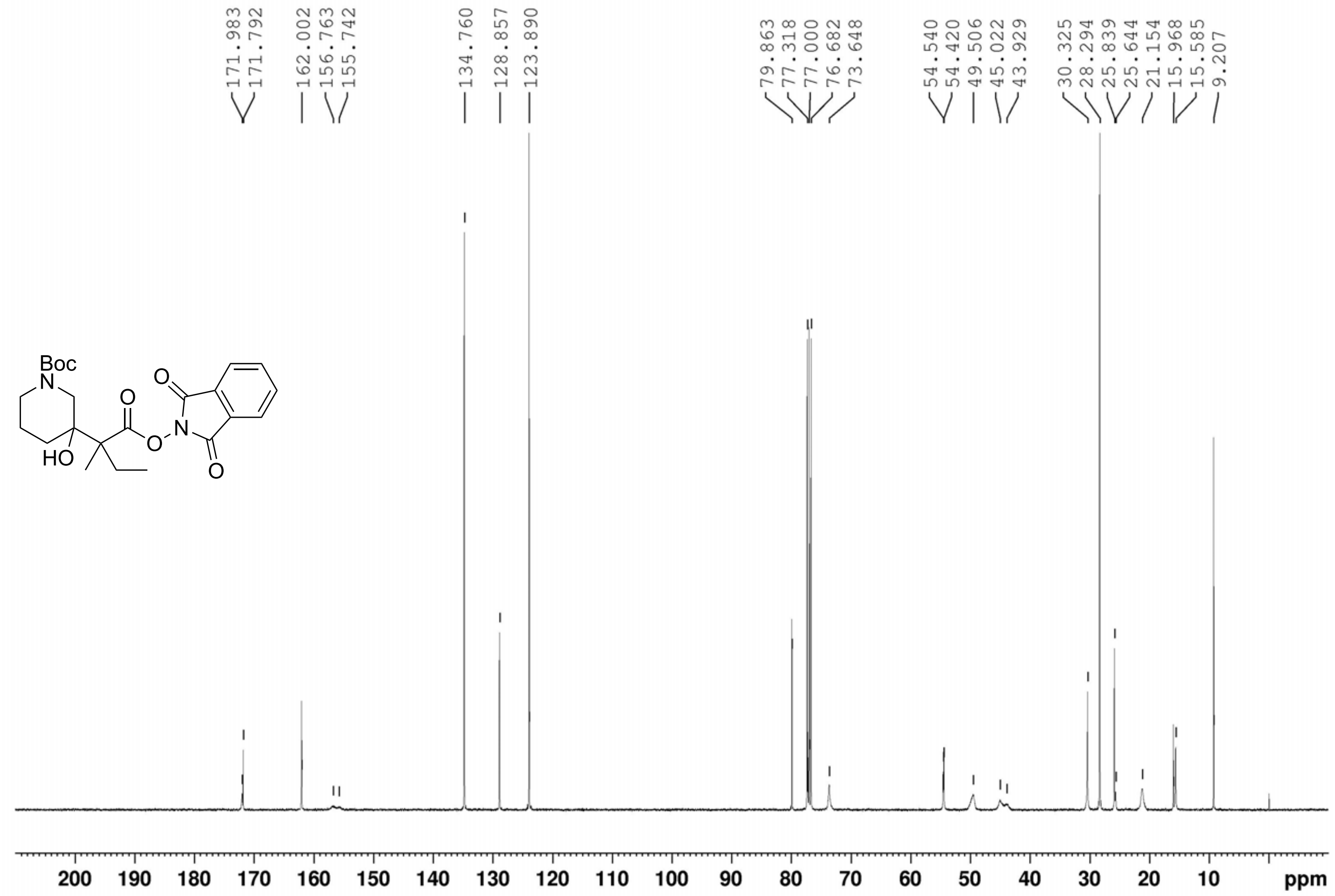

Supplementary Figure 56. ^{13}C NMR spectrum of **1w** (100.6 MHz, $CDCl_3$)

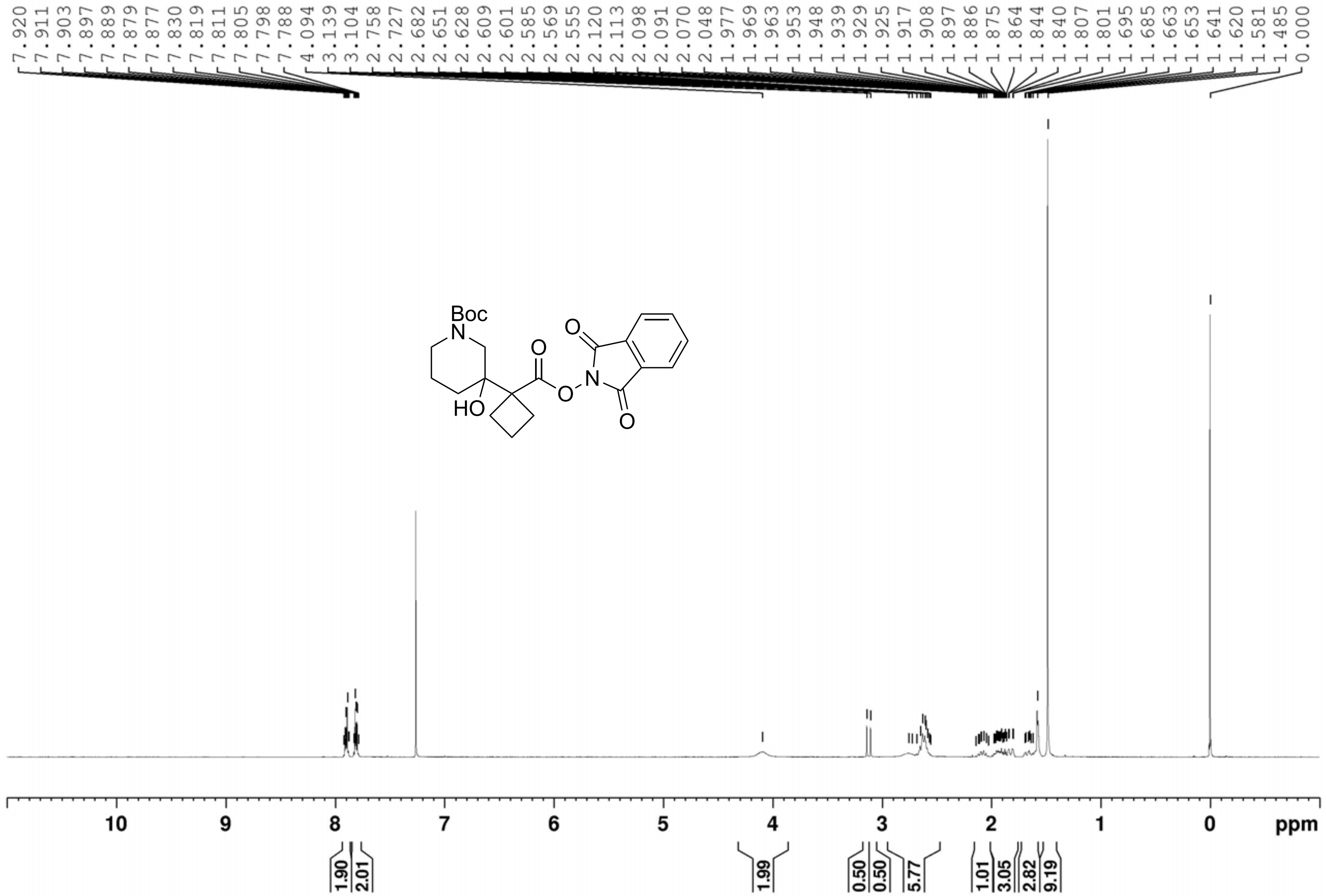

Supplementary Figure 57. ^{1}H NMR spectrum of **1x** (400 MHz, $CDCl_3$)

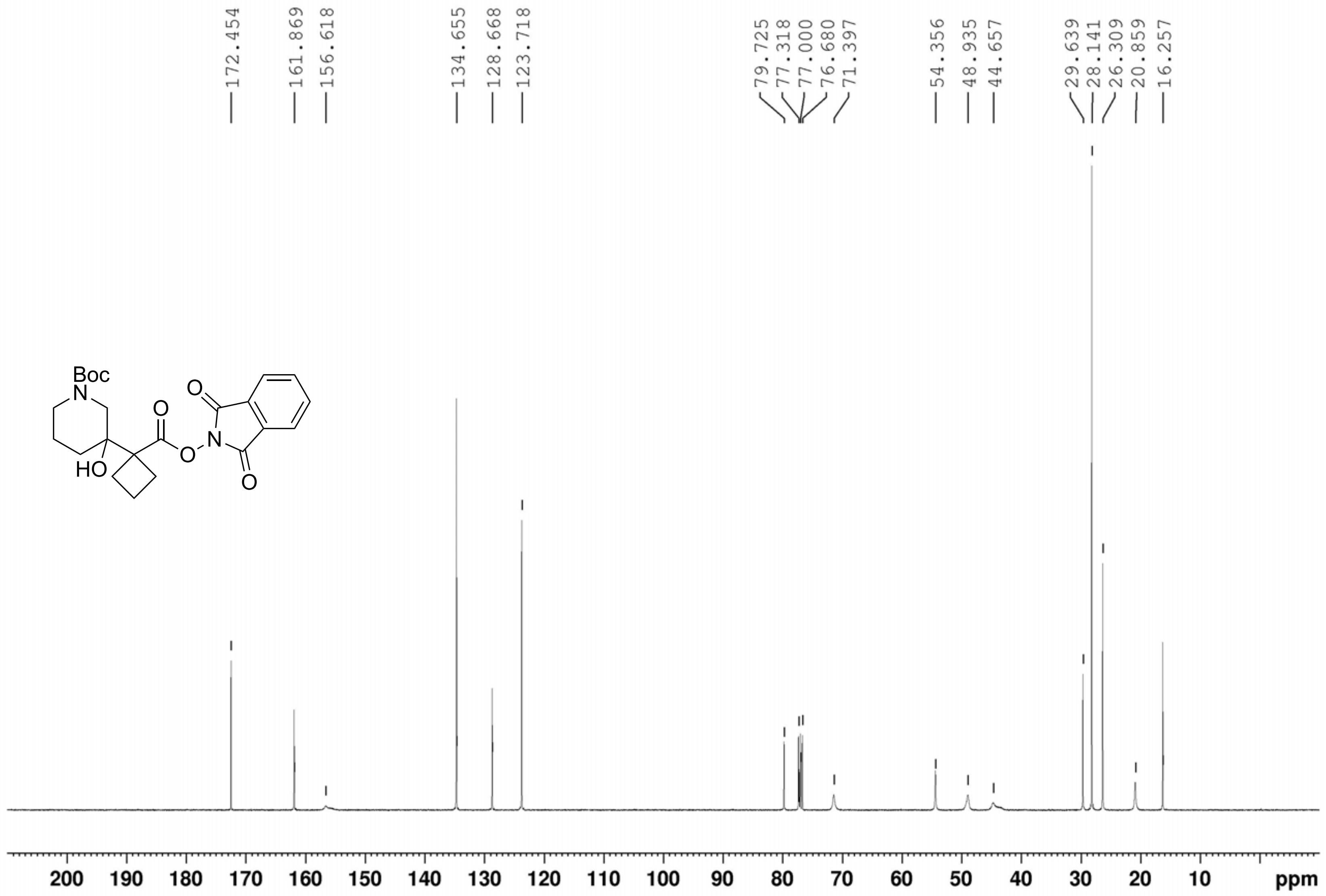

Supplementary Figure 58. ^{13}C NMR spectrum of **1x** (100.6 MHz, $CDCl_3$)

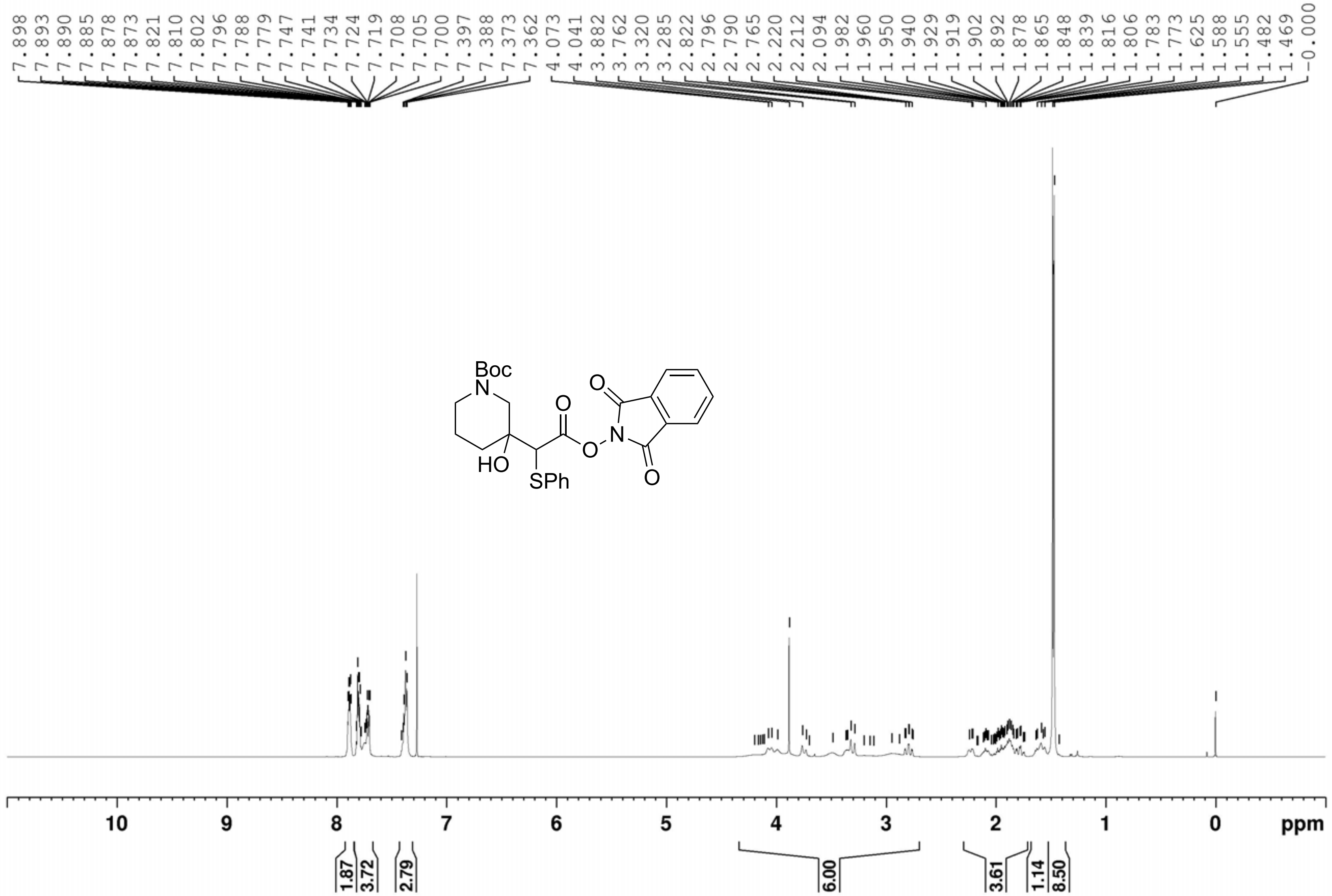

Supplementary Figure 59. ^{1}H NMR spectrum of **1y** (400 MHz, $CDCl_3$)

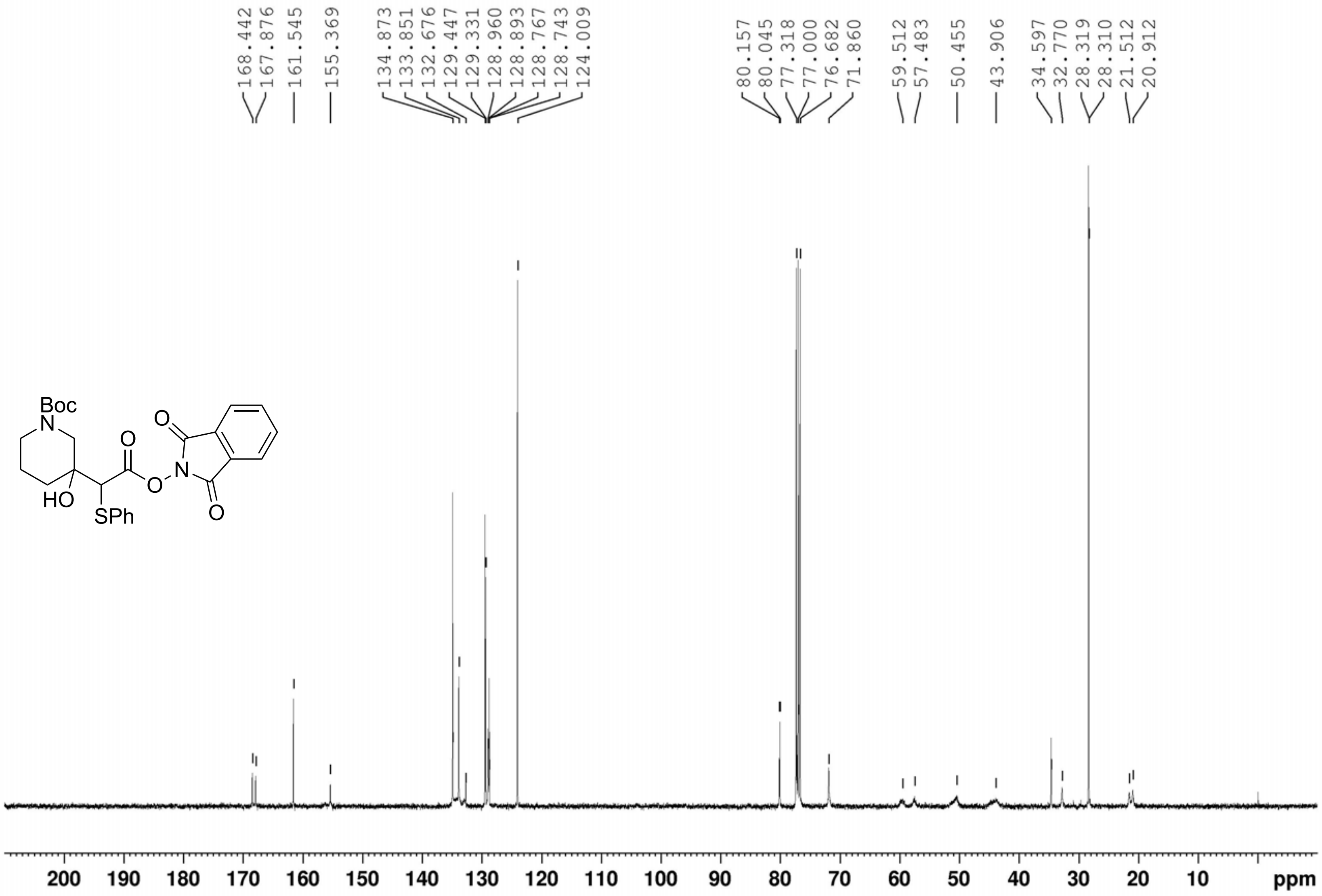

Supplementary Figure 60. ^{13}C NMR spectrum of **1y** (100.6 MHz, $CDCl_3$)

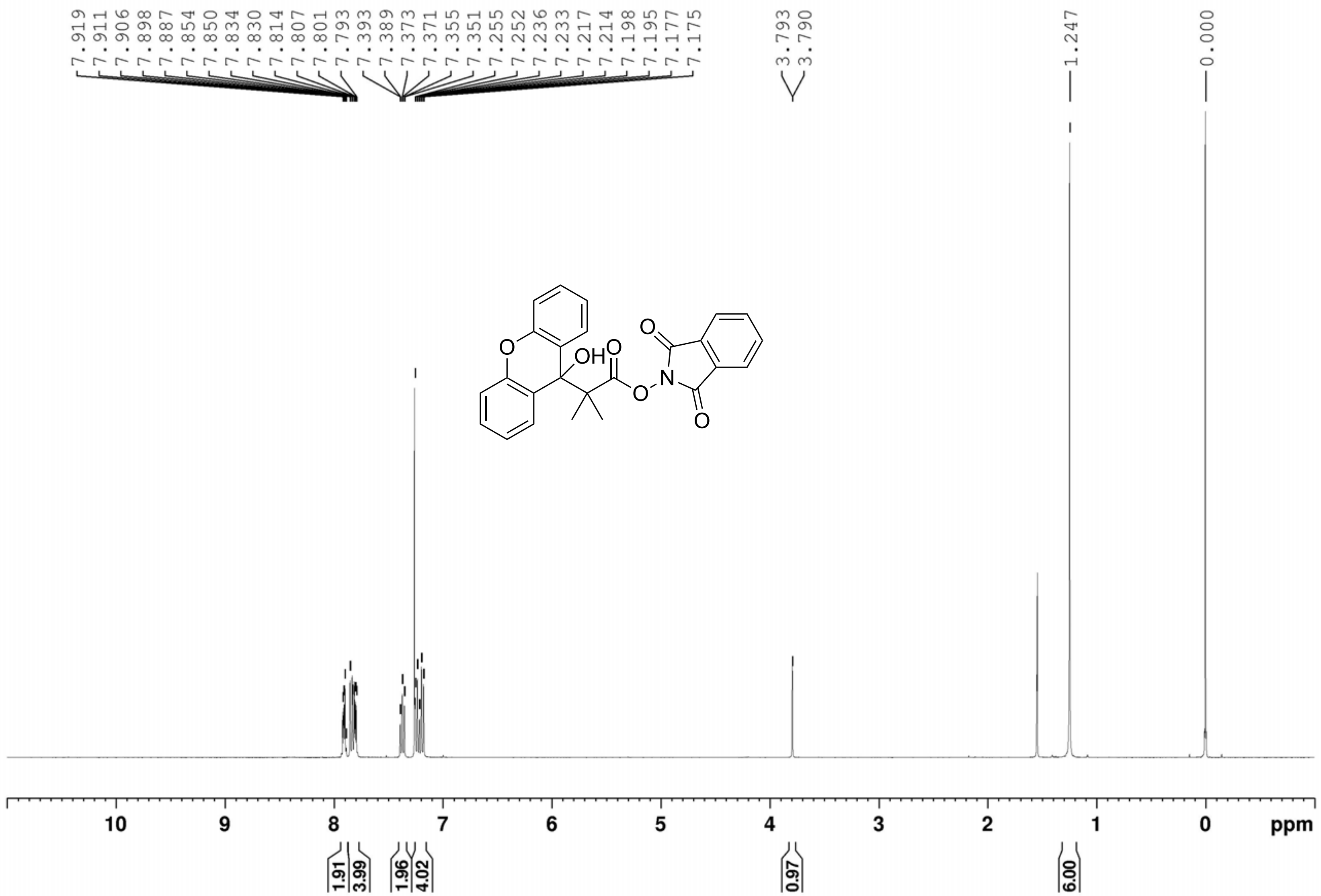

Supplementary Figure 61. ^{1}H NMR spectrum of **1z** (400 MHz, $CDCl_3$)

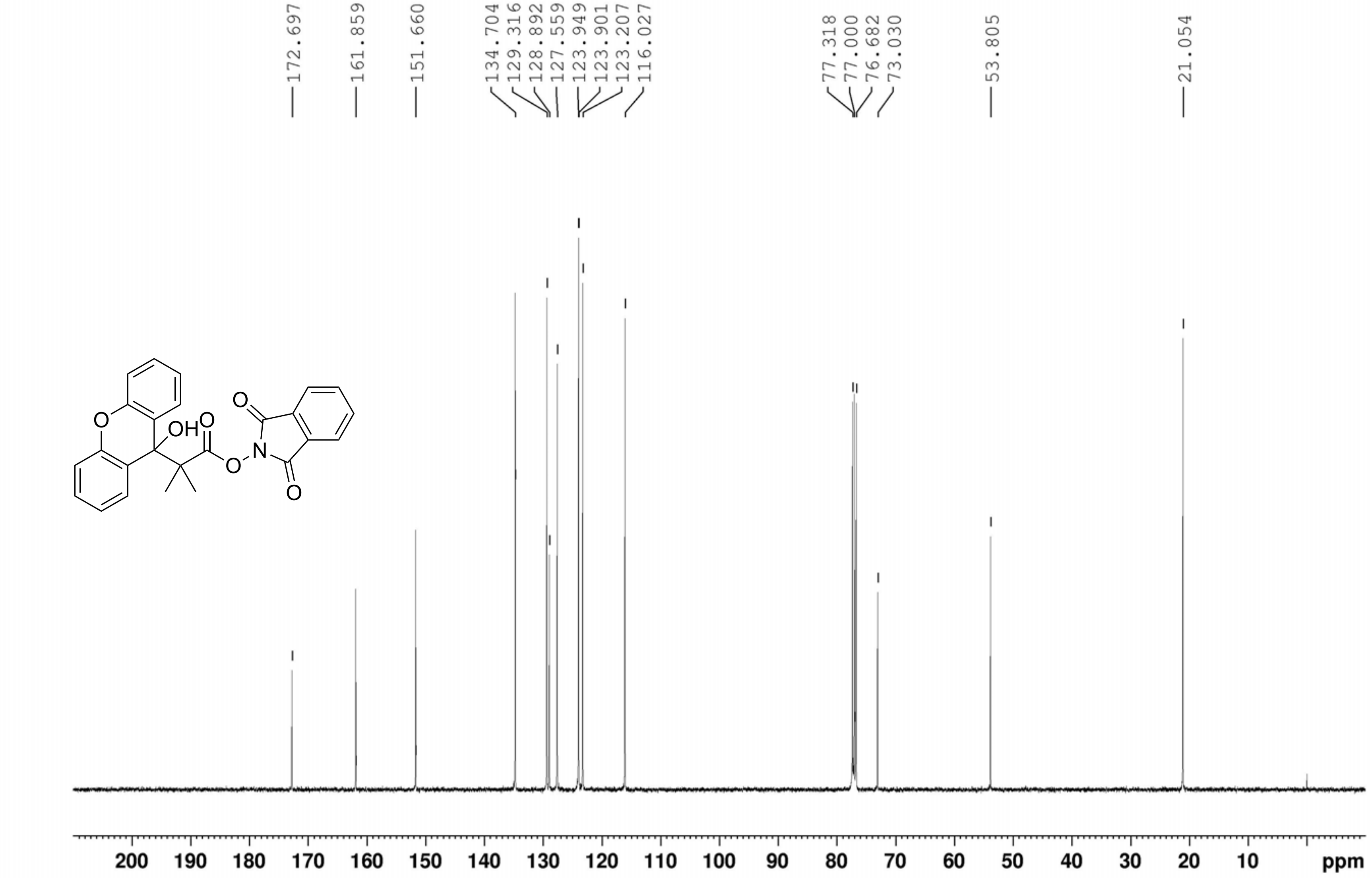

Supplementary Figure 62. ^{13}C NMR spectrum of **1z** (100.6 MHz, $CDCl_3$)

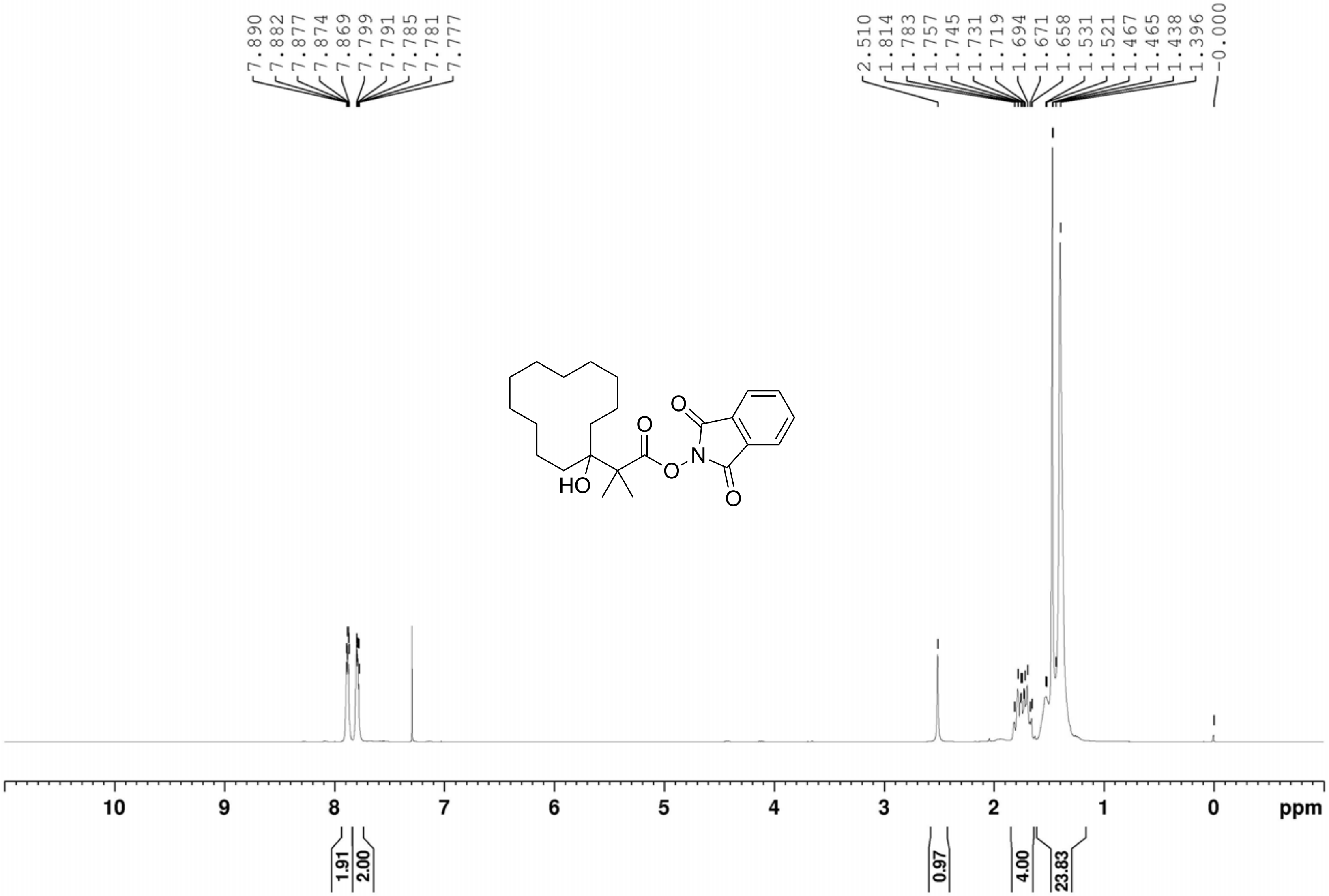

Supplementary Figure 63. ^{1}H NMR spectrum of **1A** (400 MHz, $CDCl_3$)

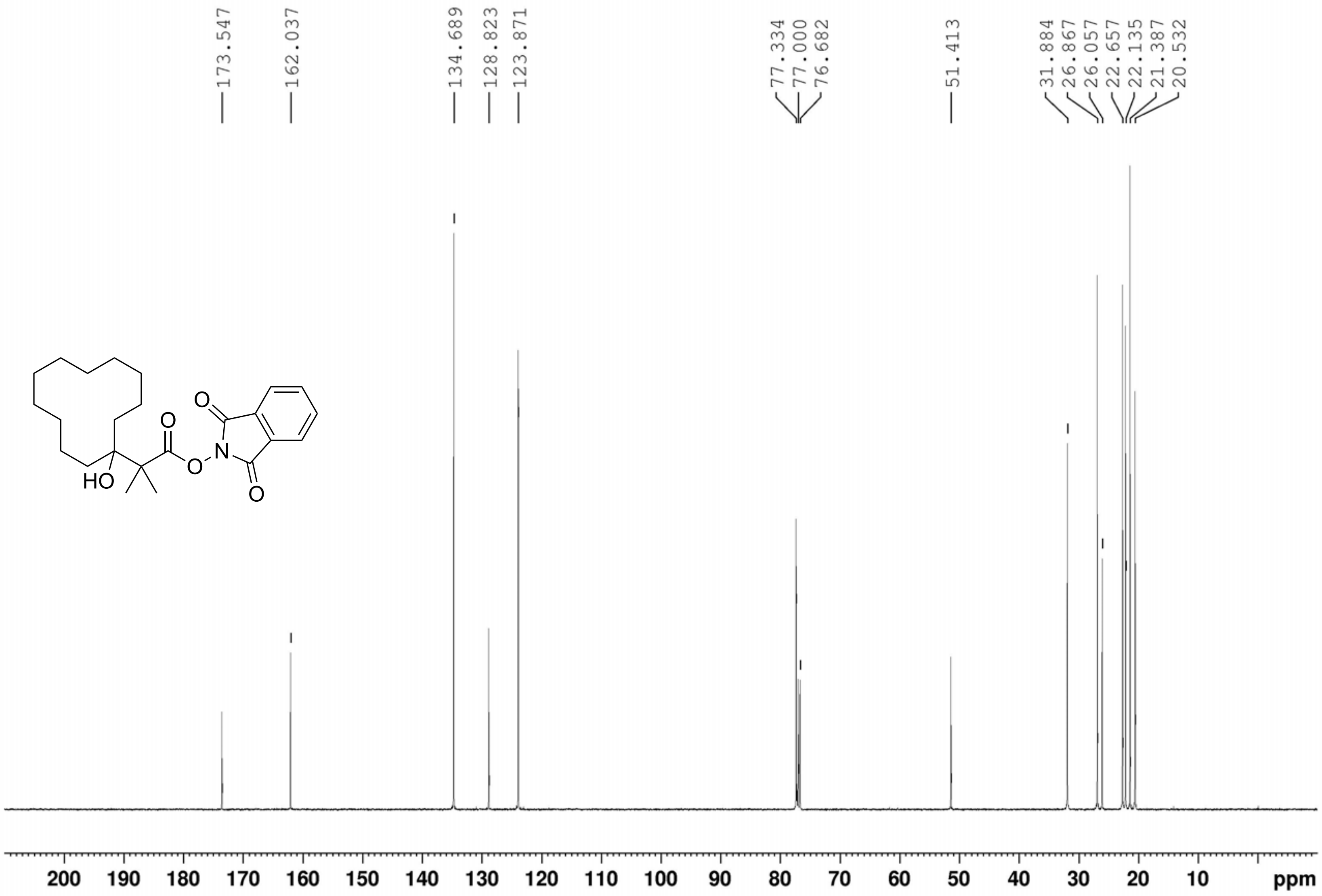

Supplementary Figure 64. ^{13}C NMR spectrum of **1A** (100.6 MHz, $CDCl_3$)

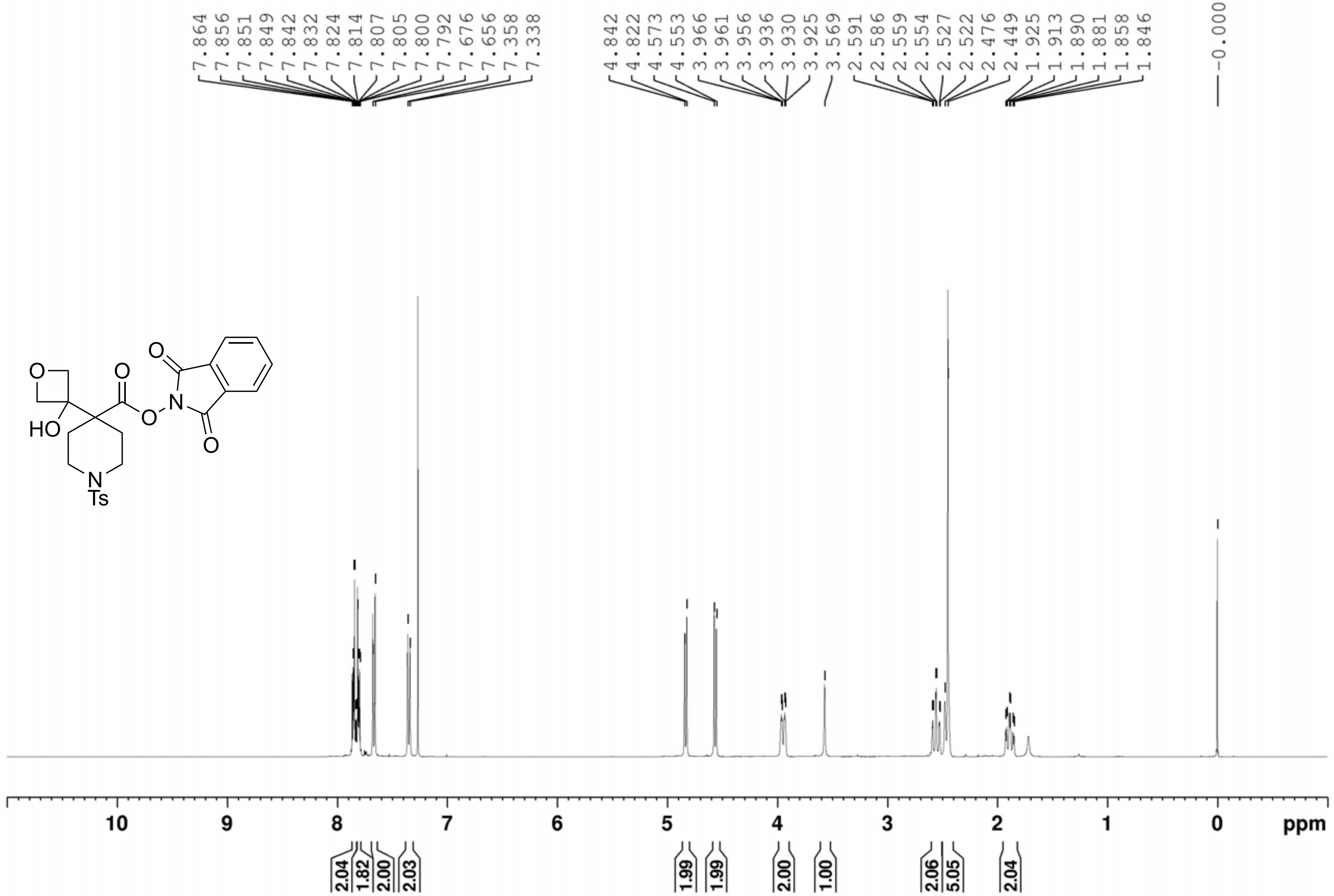

Supplementary Figure 65. ^{1}H NMR spectrum of **1B** (400 MHz, $CDCl_3$)

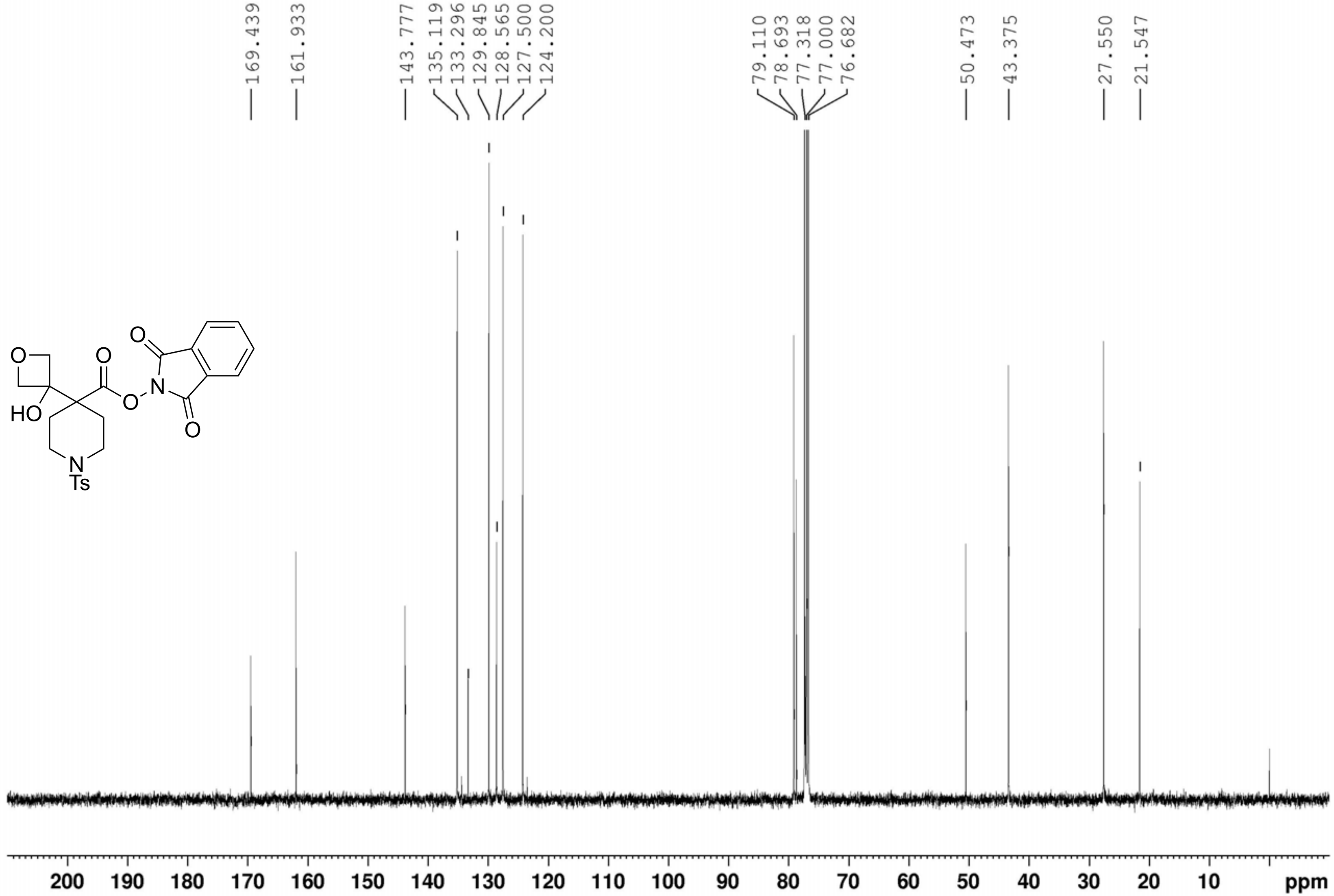

Supplementary Figure 66. ^{13}C NMR spectrum of **1B** (100.6 MHz, $CDCl_3$)

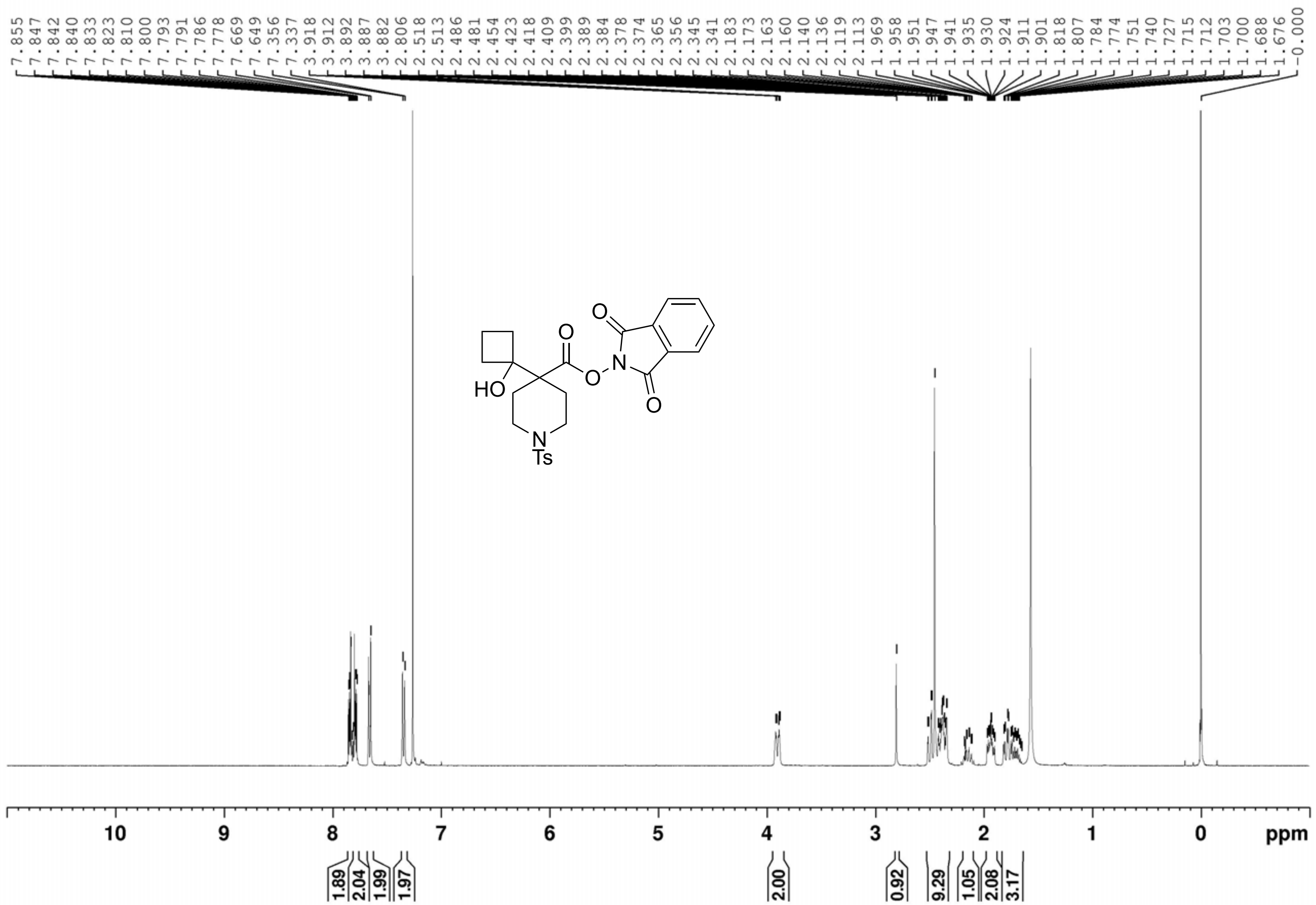

Supplementary Figure 67. ^{1}H NMR spectrum of **1C** (400 MHz, $CDCl_3$)

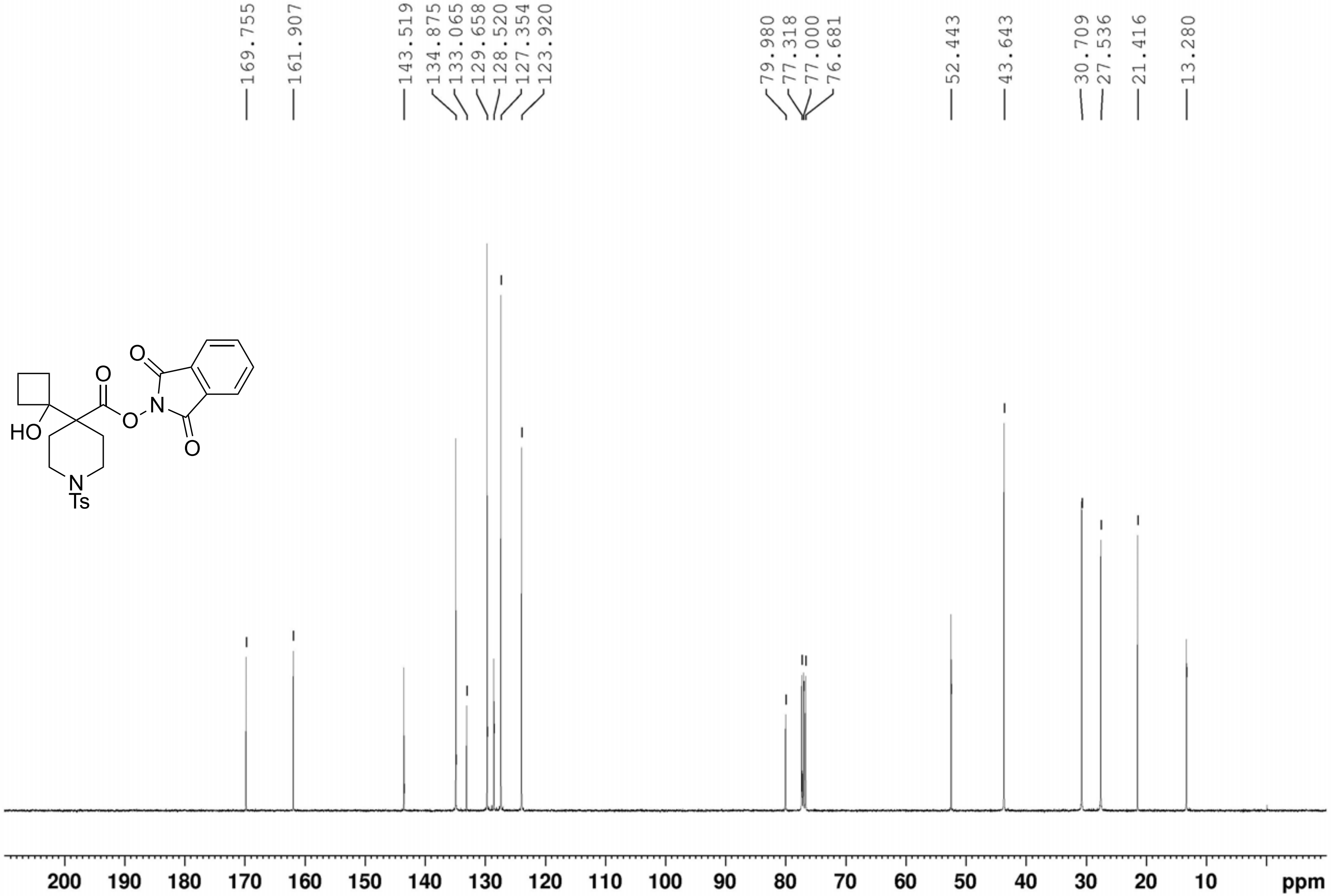

Supplementary Figure 68. ^{13}C NMR spectrum of **1C** (100.6 MHz, $CDCl_3$)

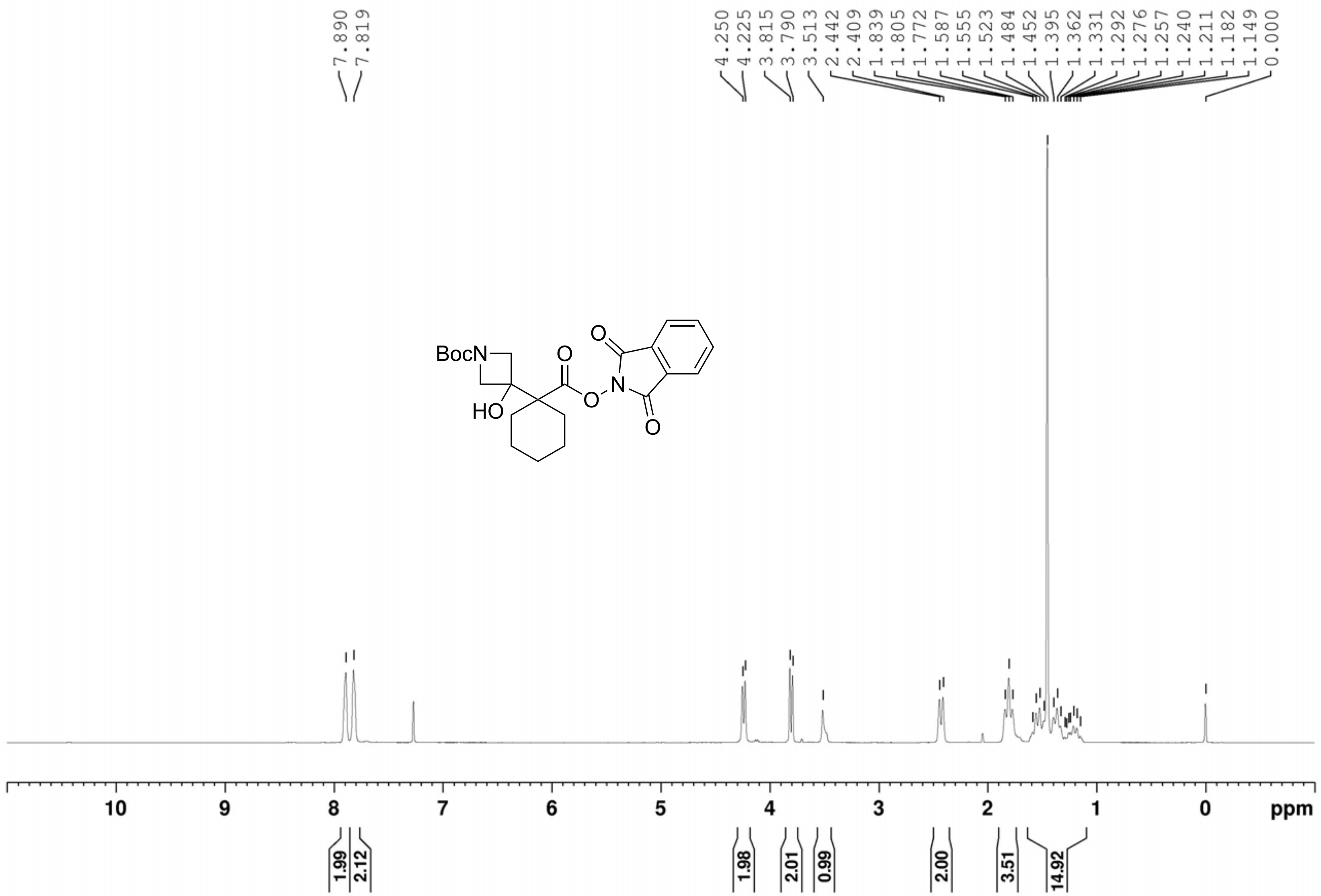

Supplementary Figure 69. ^{1}H NMR spectrum of **1D** (400 MHz, $CDCl_3$)

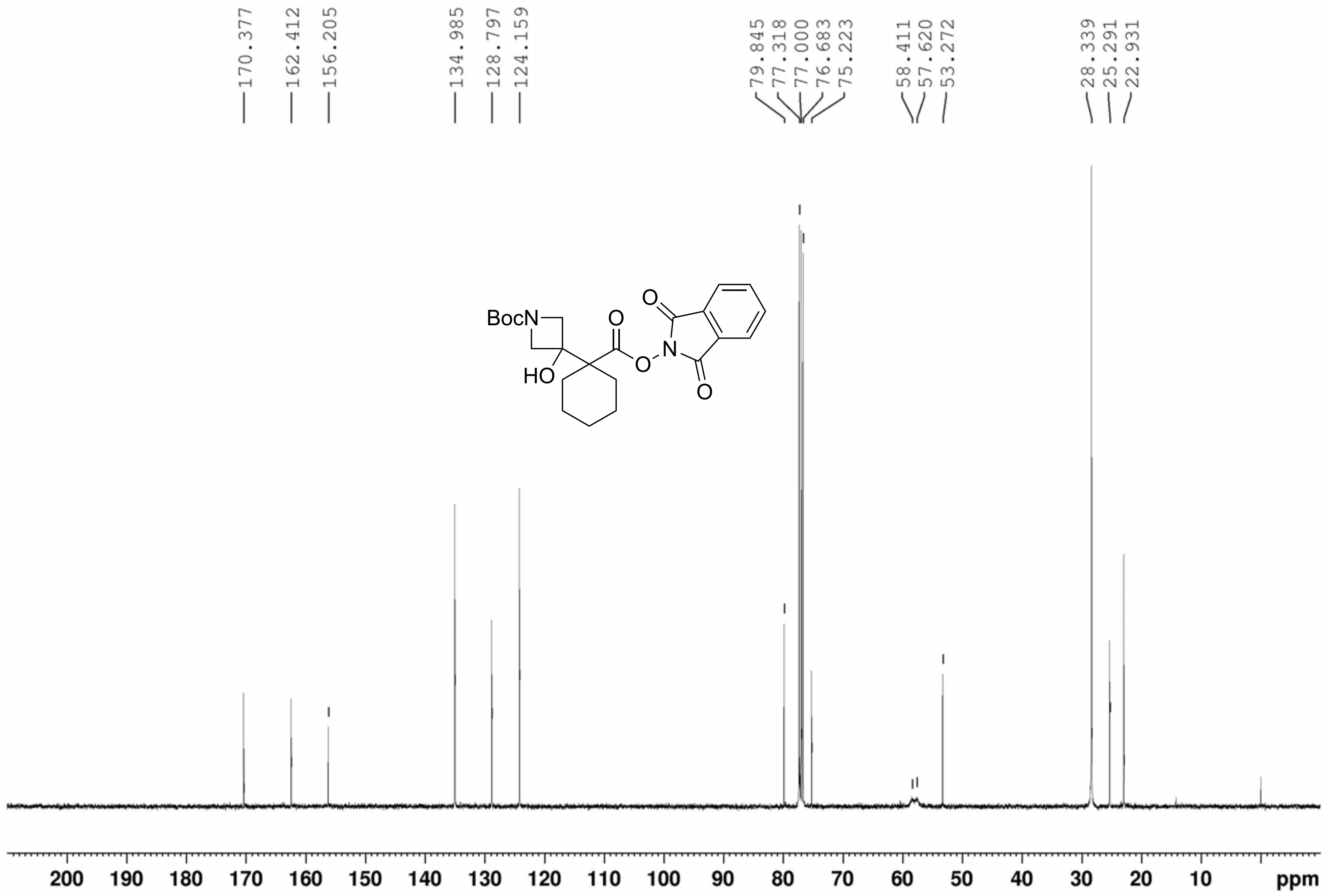

Supplementary Figure 70. ^{13}C NMR spectrum of **1D** (100.6 MHz, $CDCl_3$)

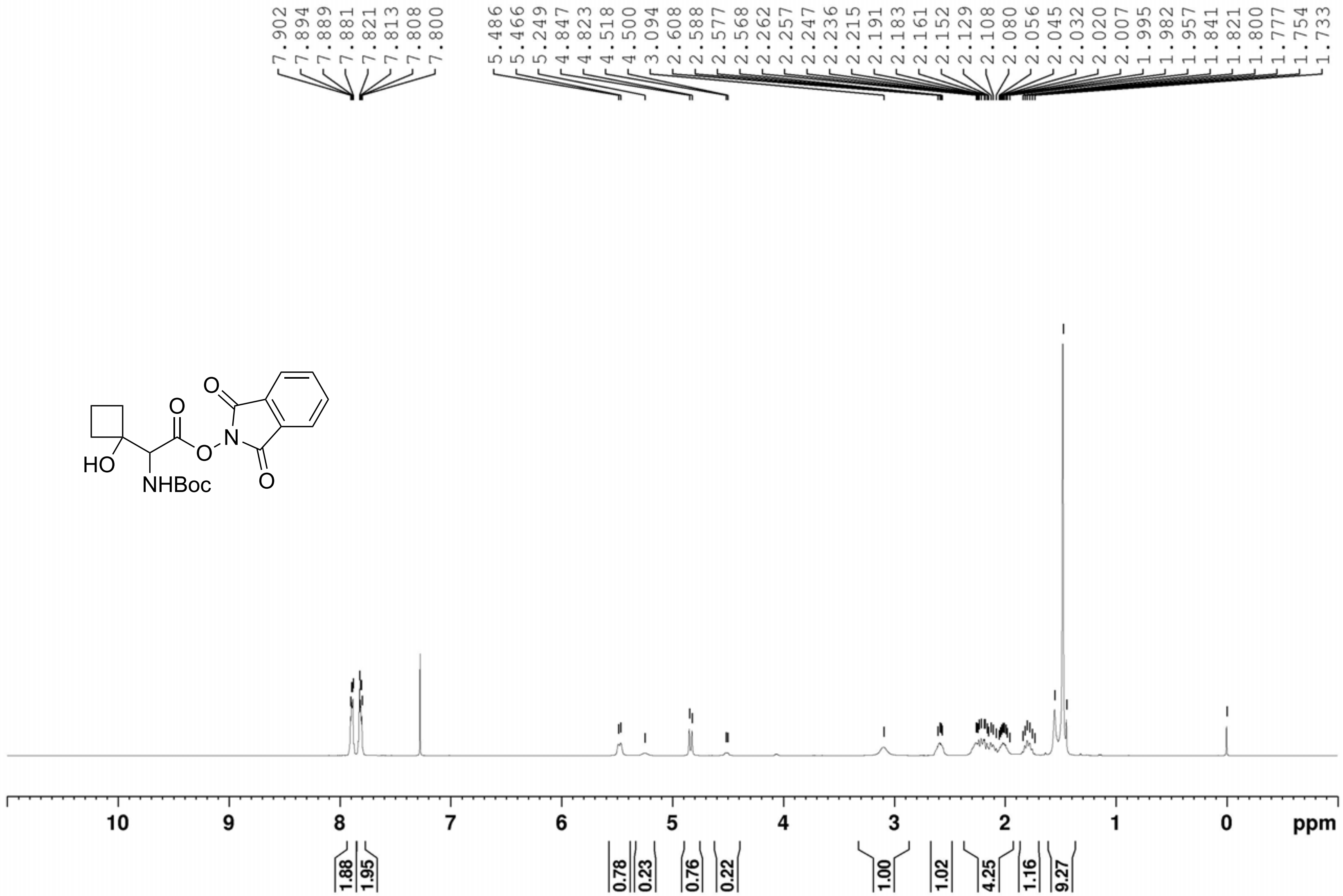

Supplementary Figure 71. ^{1}H NMR spectrum of **1E** (400 MHz, $CDCl_3$)

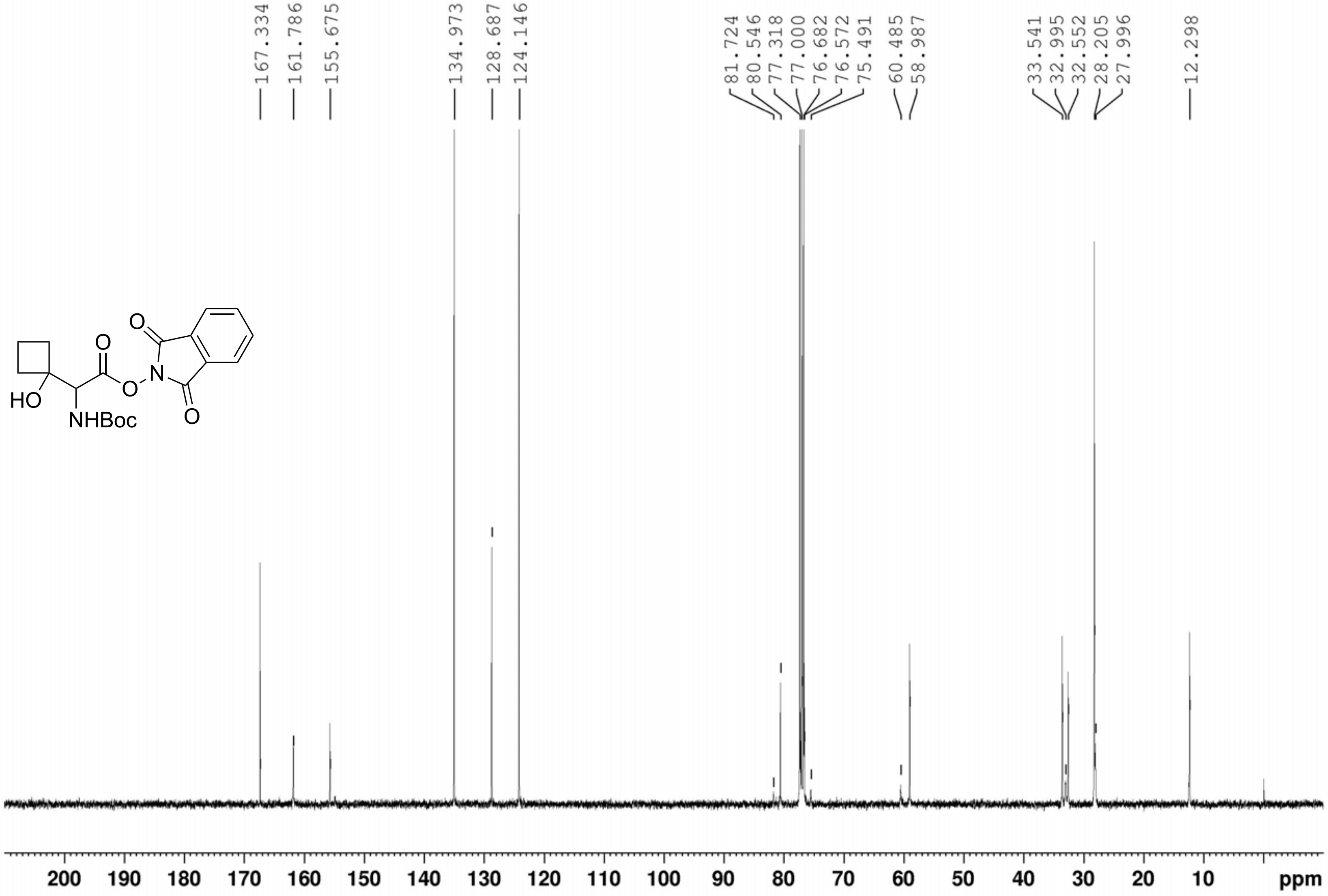

Supplementary Figure 72. ^{13}C NMR spectrum of **1E** (100.6 MHz, $CDCl_3$)

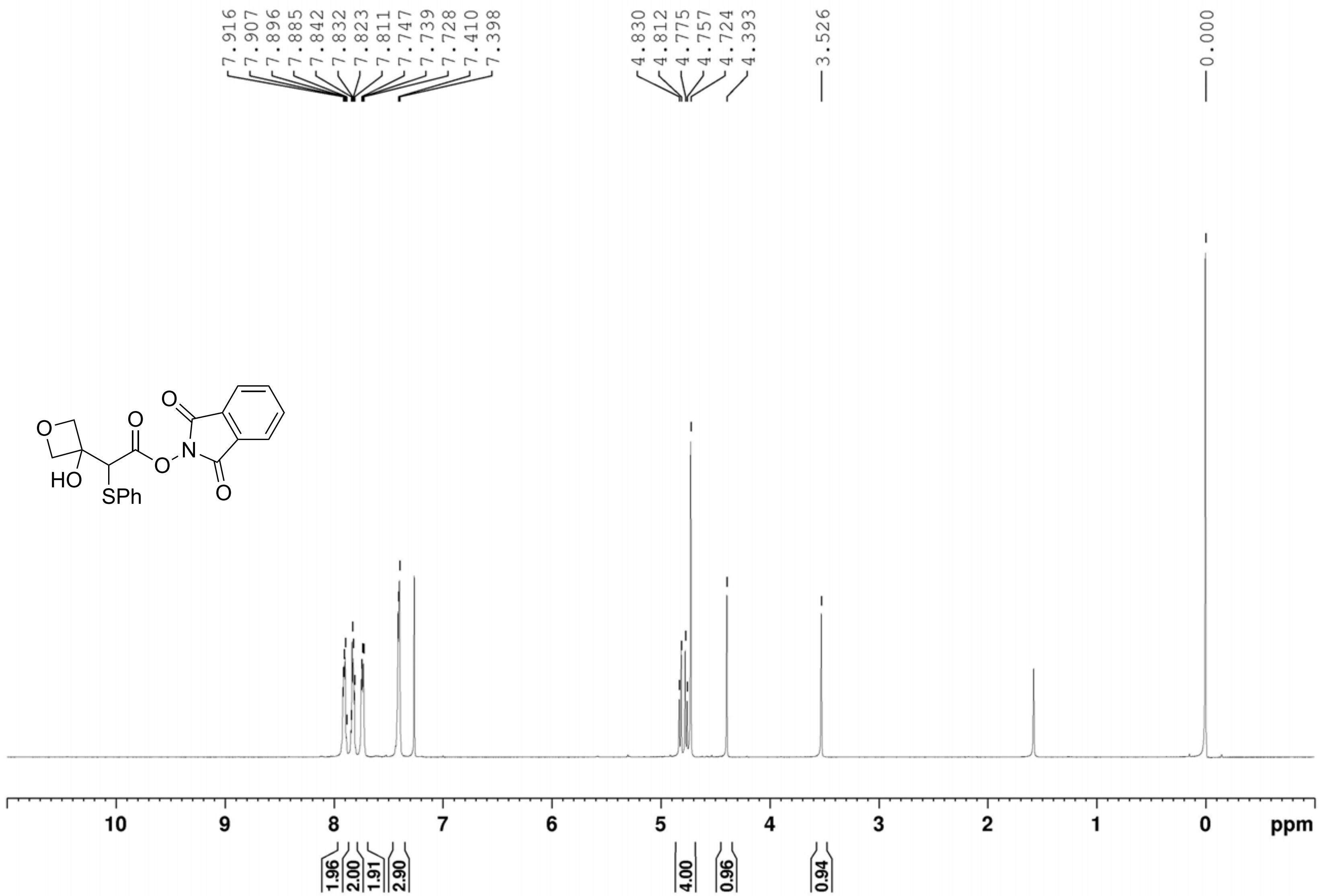

Supplementary Figure 73. ^{1}H NMR spectrum of **1F** (400 MHz, $CDCl_3$)

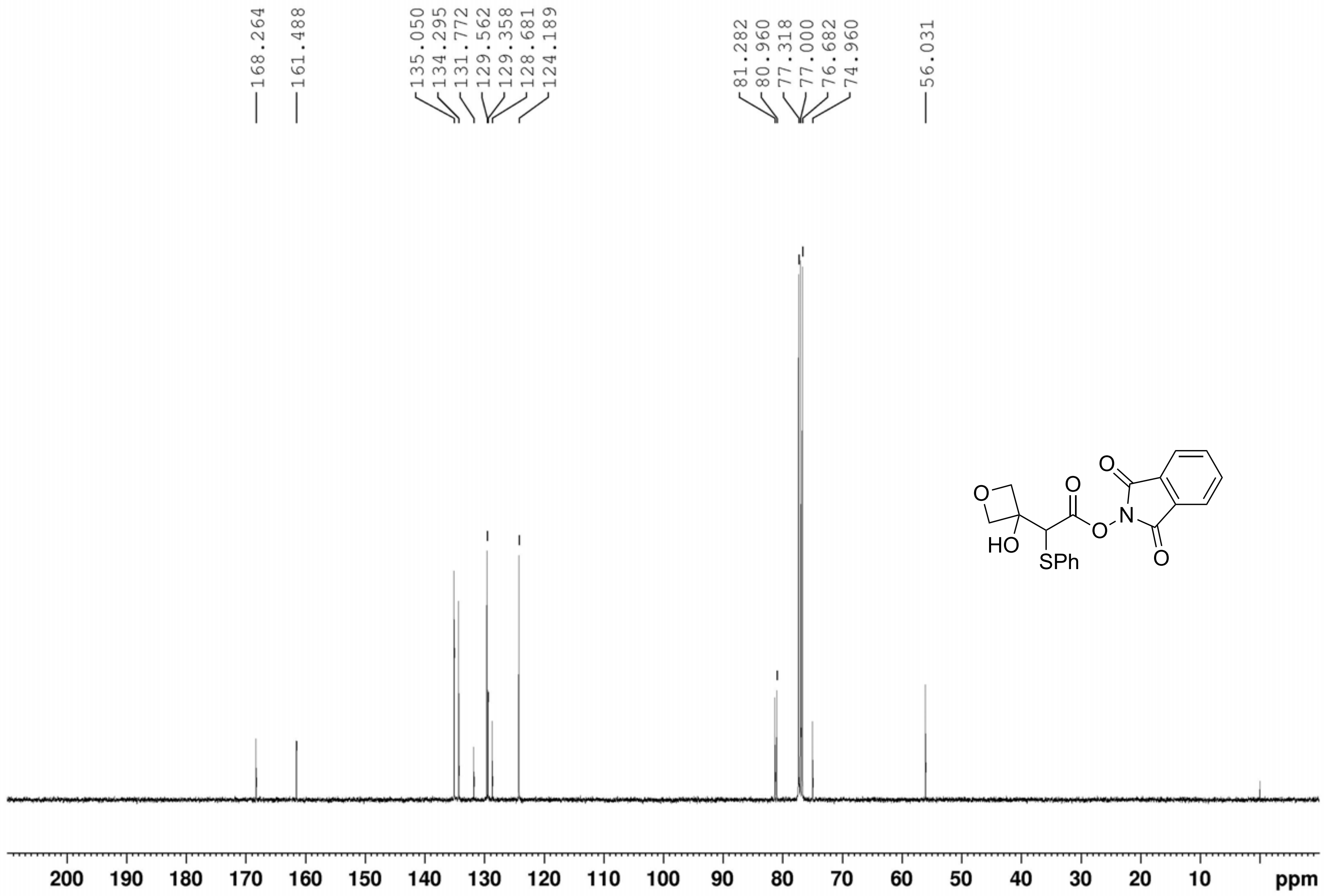

Supplementary Figure 74. ^{13}C NMR spectrum of **1F** (100.6 MHz, $CDCl_3$)

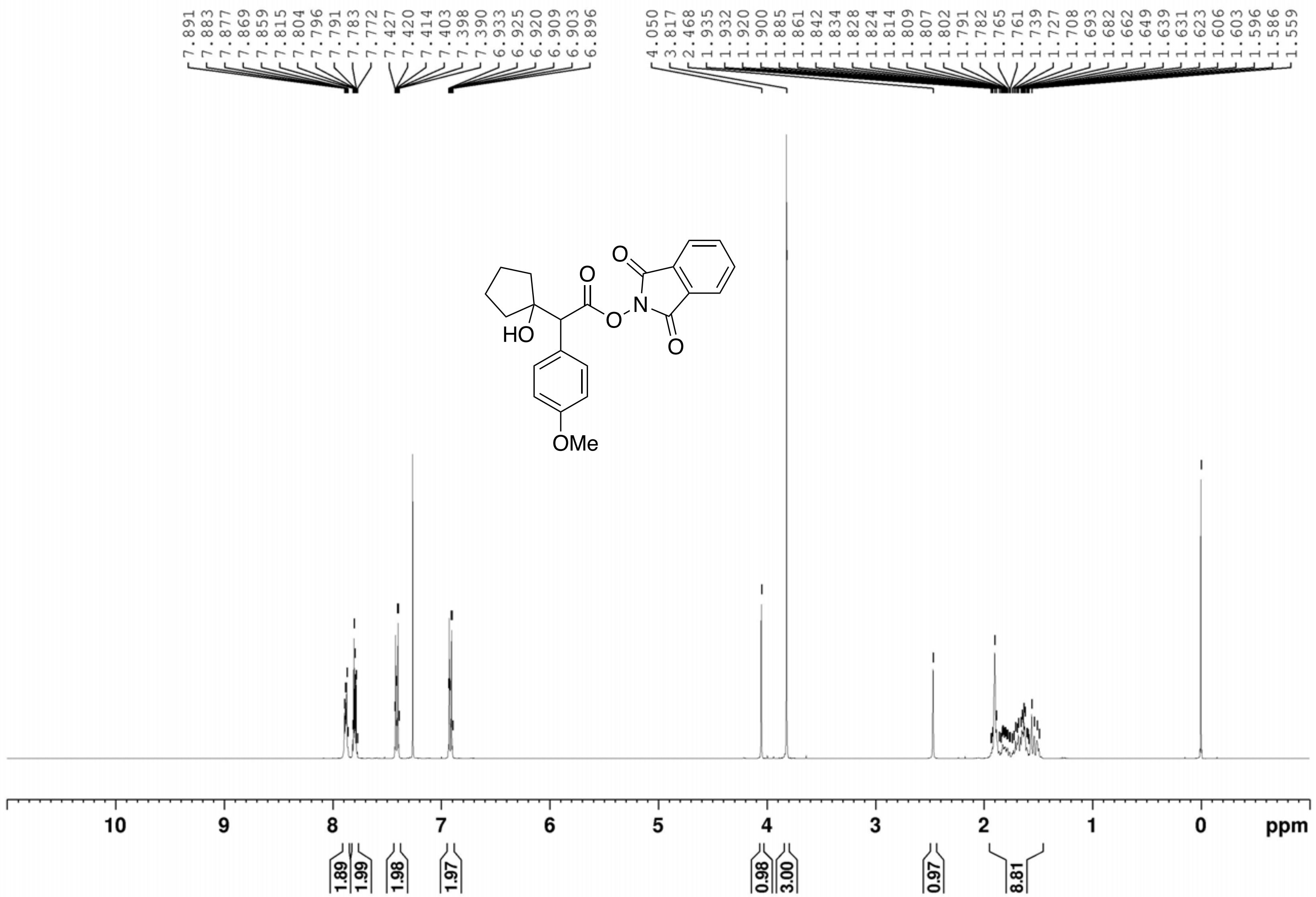

Supplementary Figure 75. ^{1}H NMR spectrum of **1G** (400 MHz, $CDCl_3$)

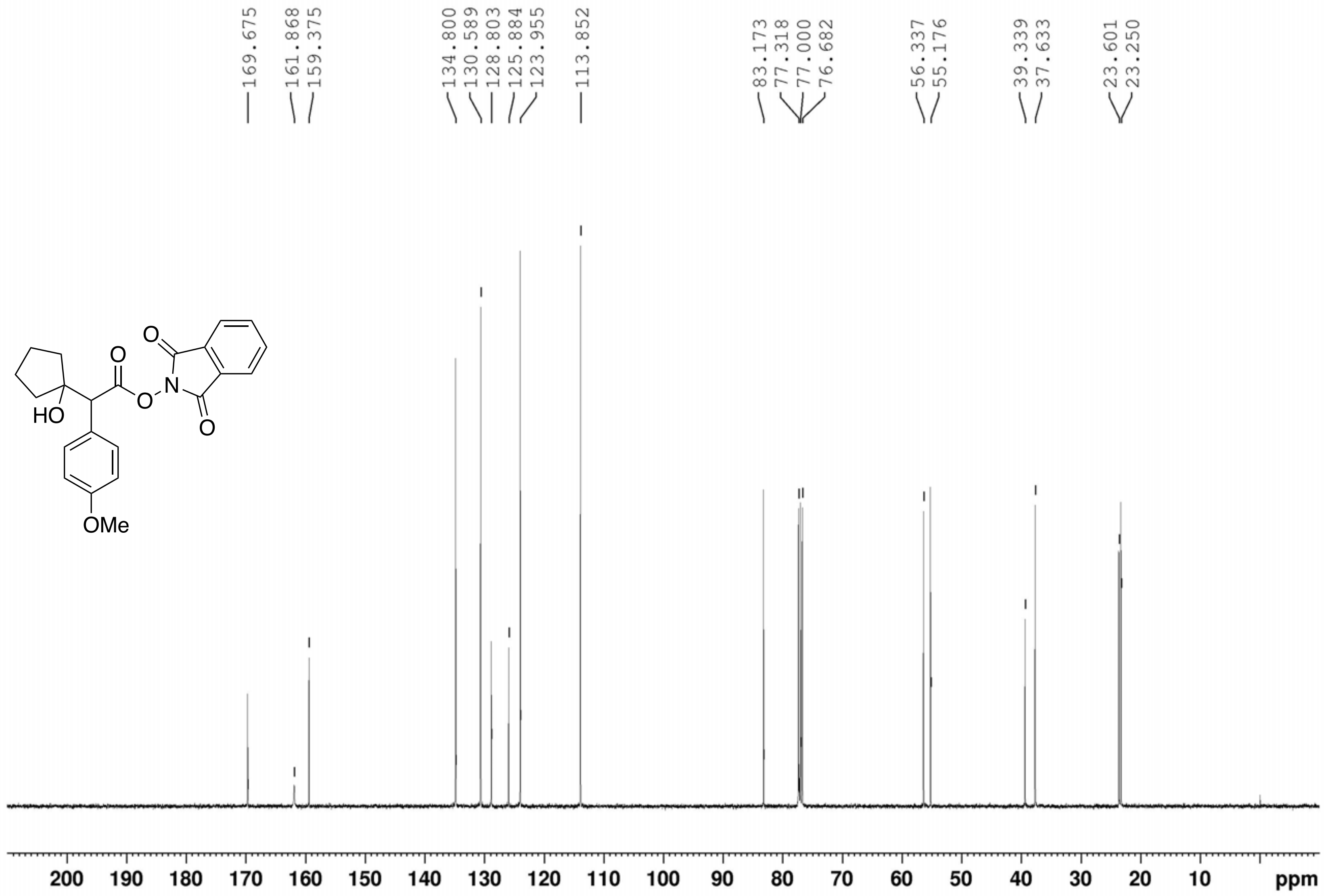

Supplementary Figure 76. ^{13}C NMR spectrum of **1G** (100.6 MHz, $CDCl_3$)

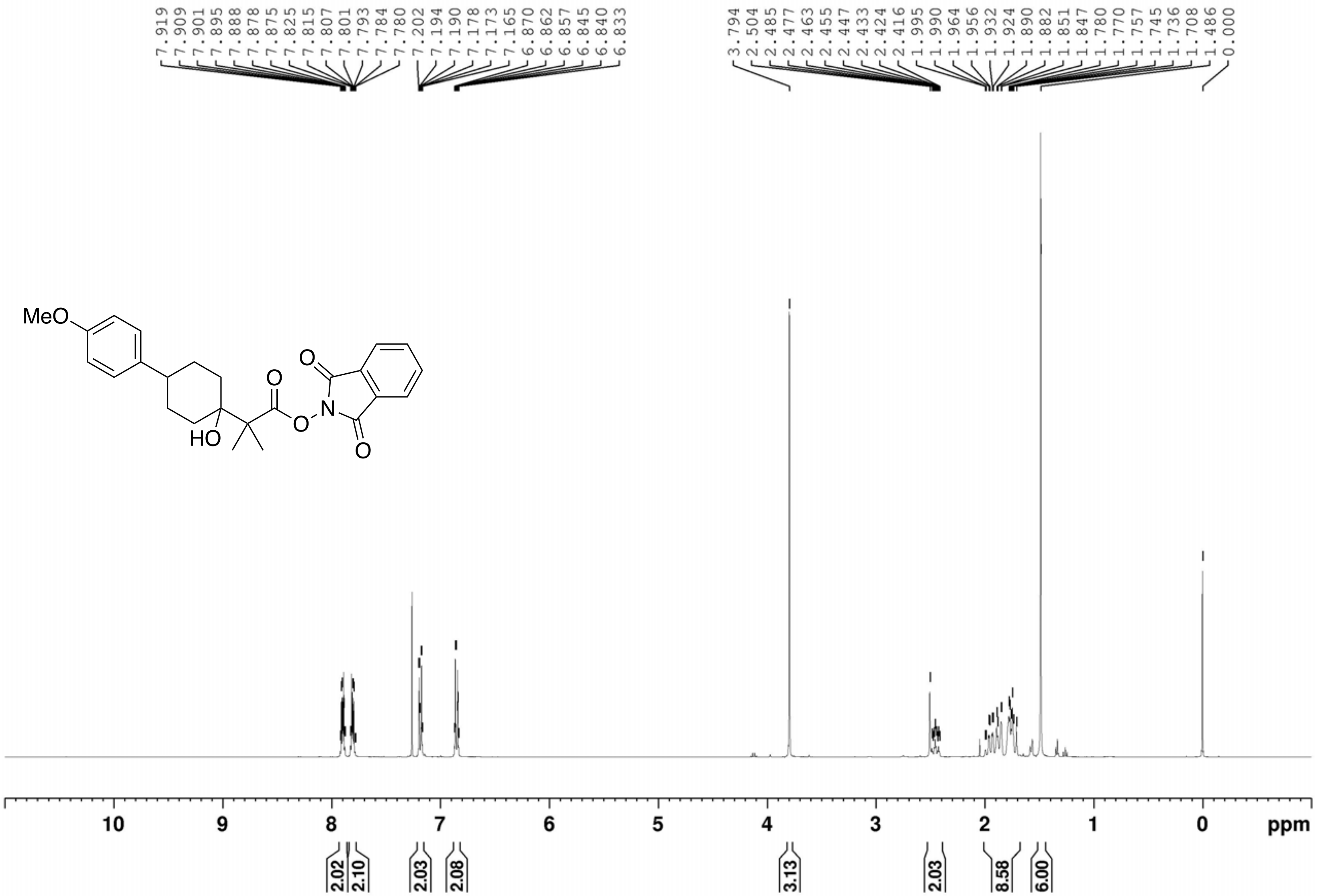

Supplementary Figure 77. ^{1}H NMR spectrum of **1H** (400 MHz, $CDCl_3$)

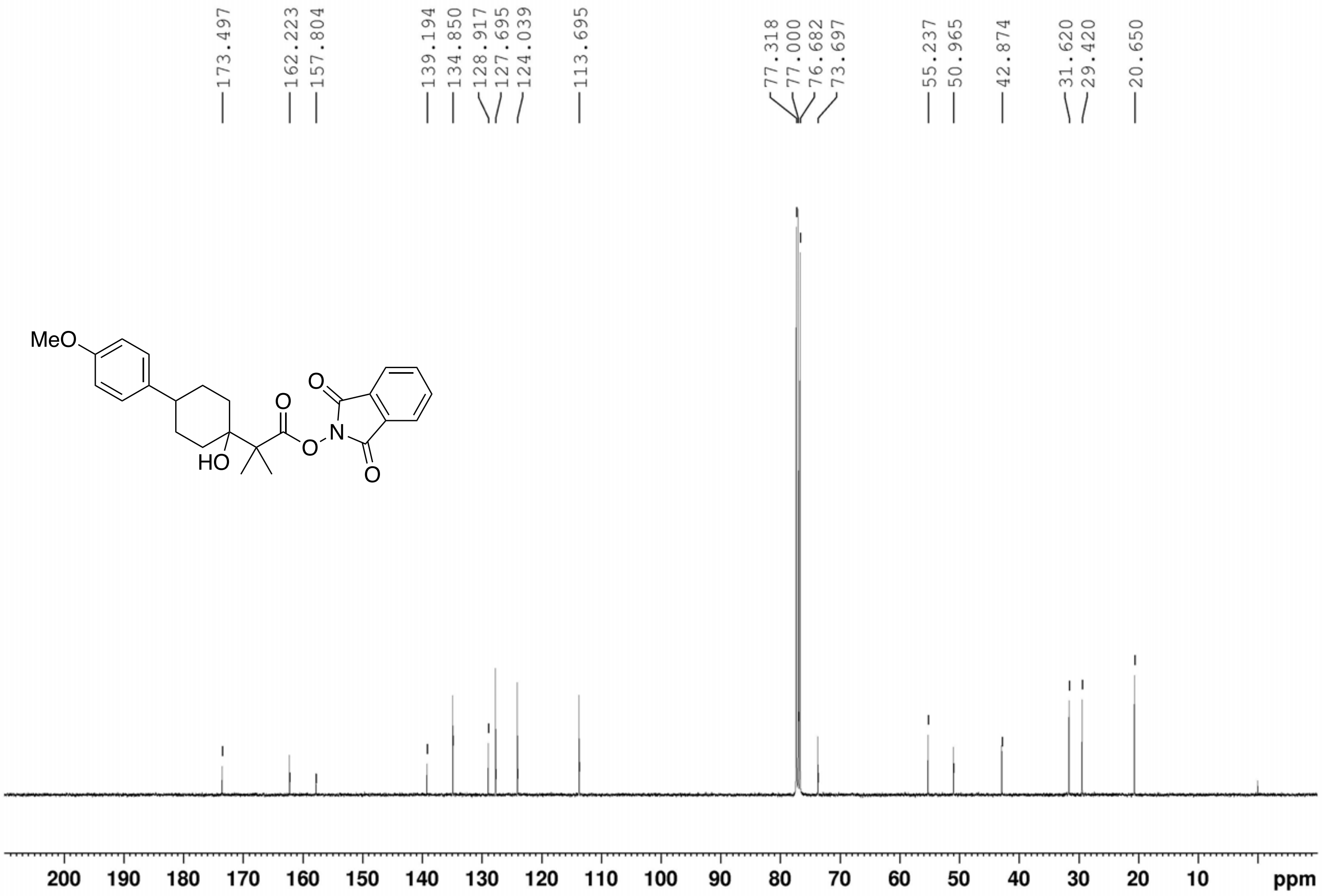

Supplementary Figure 78. ^{13}C NMR spectrum of **1H** (100.6 MHz, $CDCl_3$)

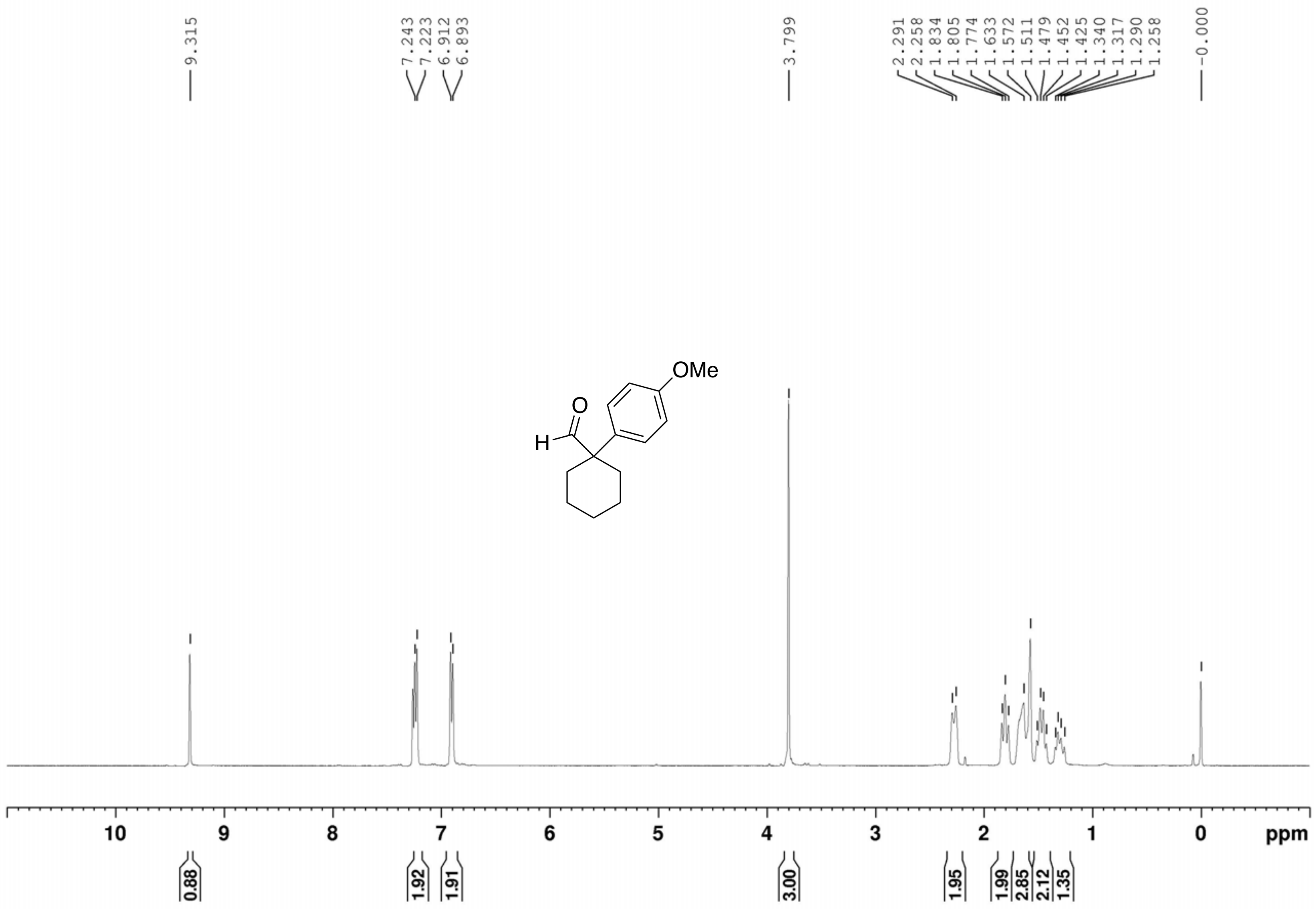

Supplementary Figure 79. ^{1}H NMR spectrum of **2a** (400 MHz, $CDCl_3$)

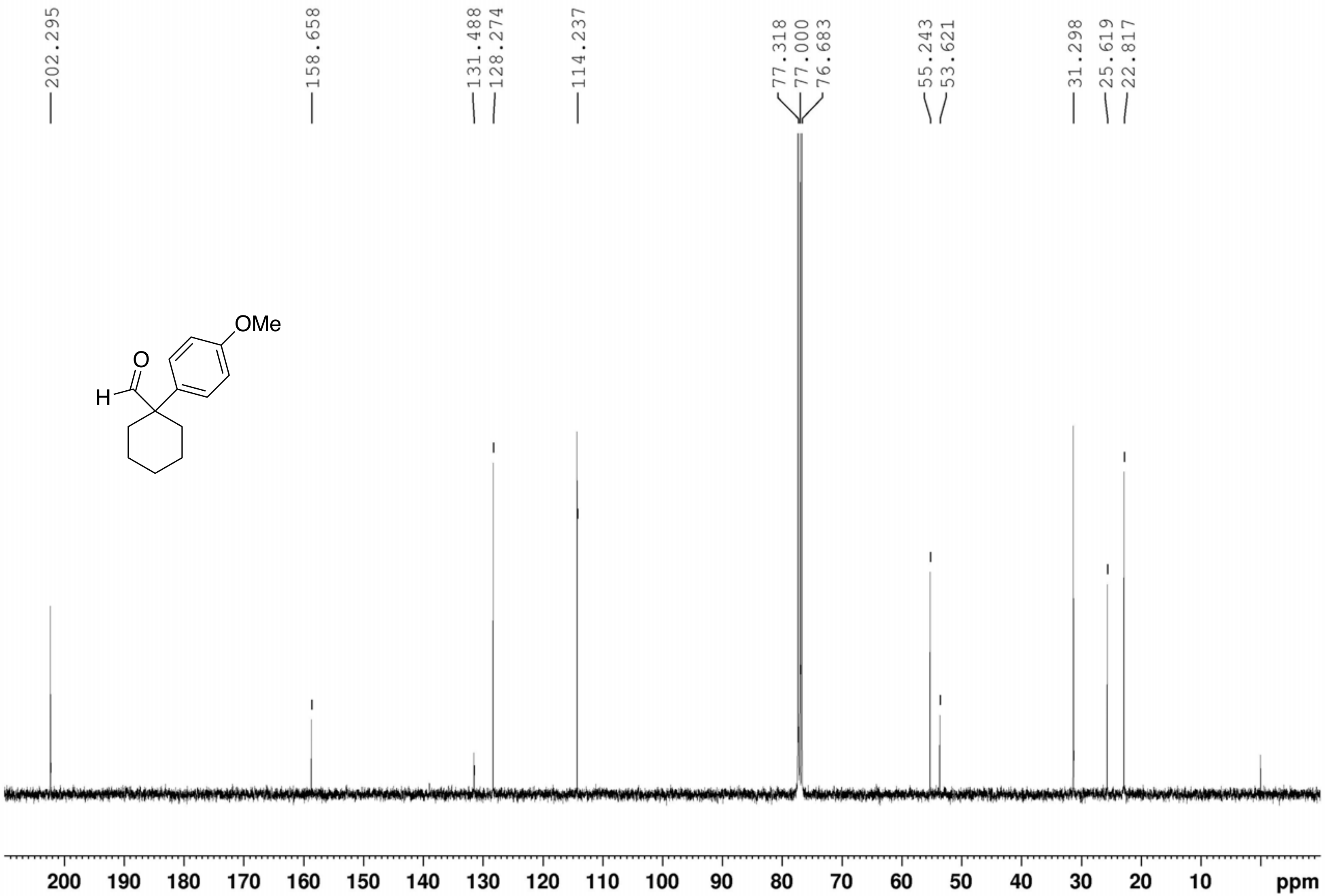

Supplementary Figure 80. ^{13}C NMR spectrum of **2a** (100.6 MHz, $CDCl_3$)

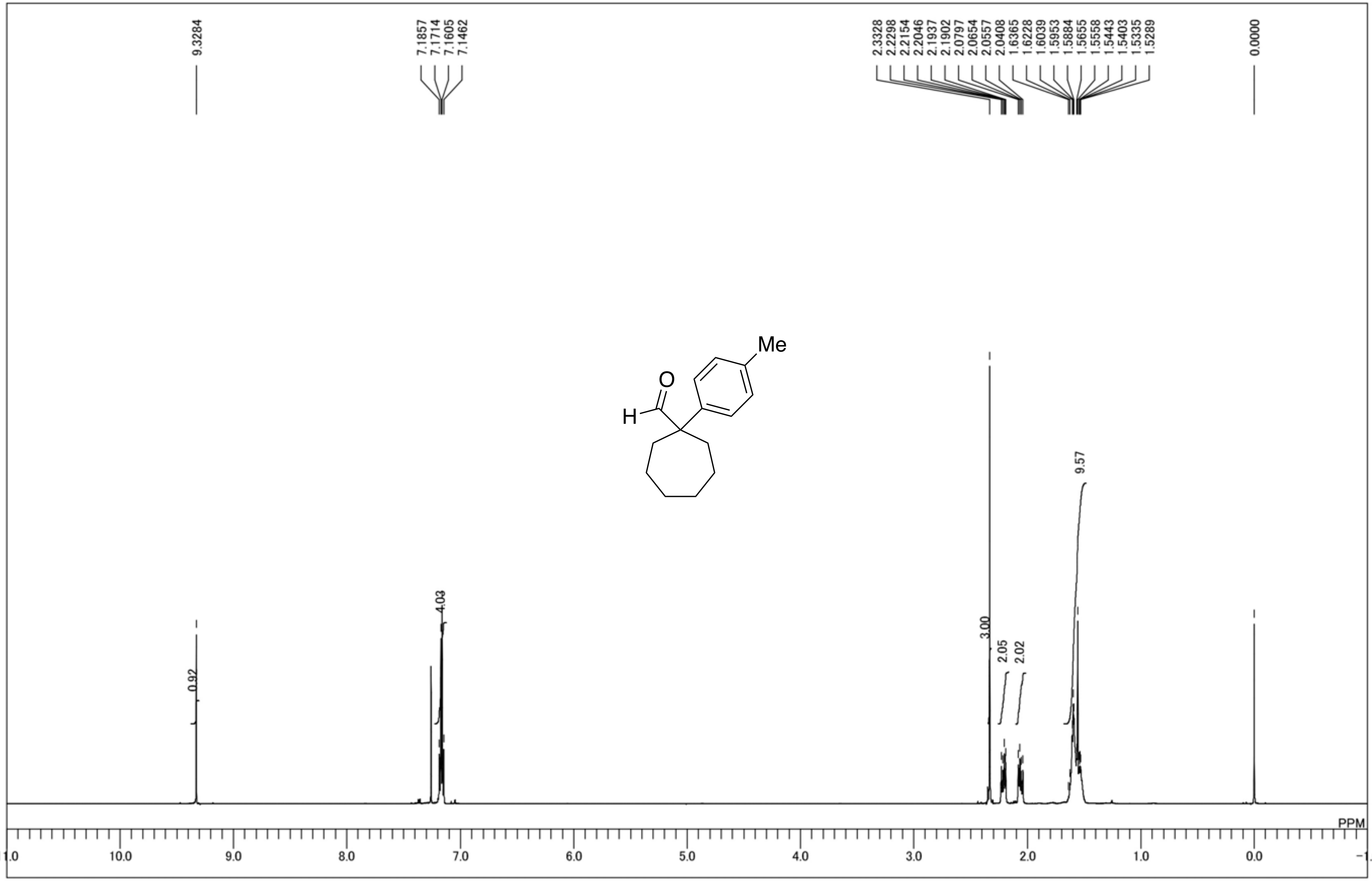

Supplementary Figure 81. ^{1}H NMR spectrum of **2b** (600 MHz, $CDCl_3$)

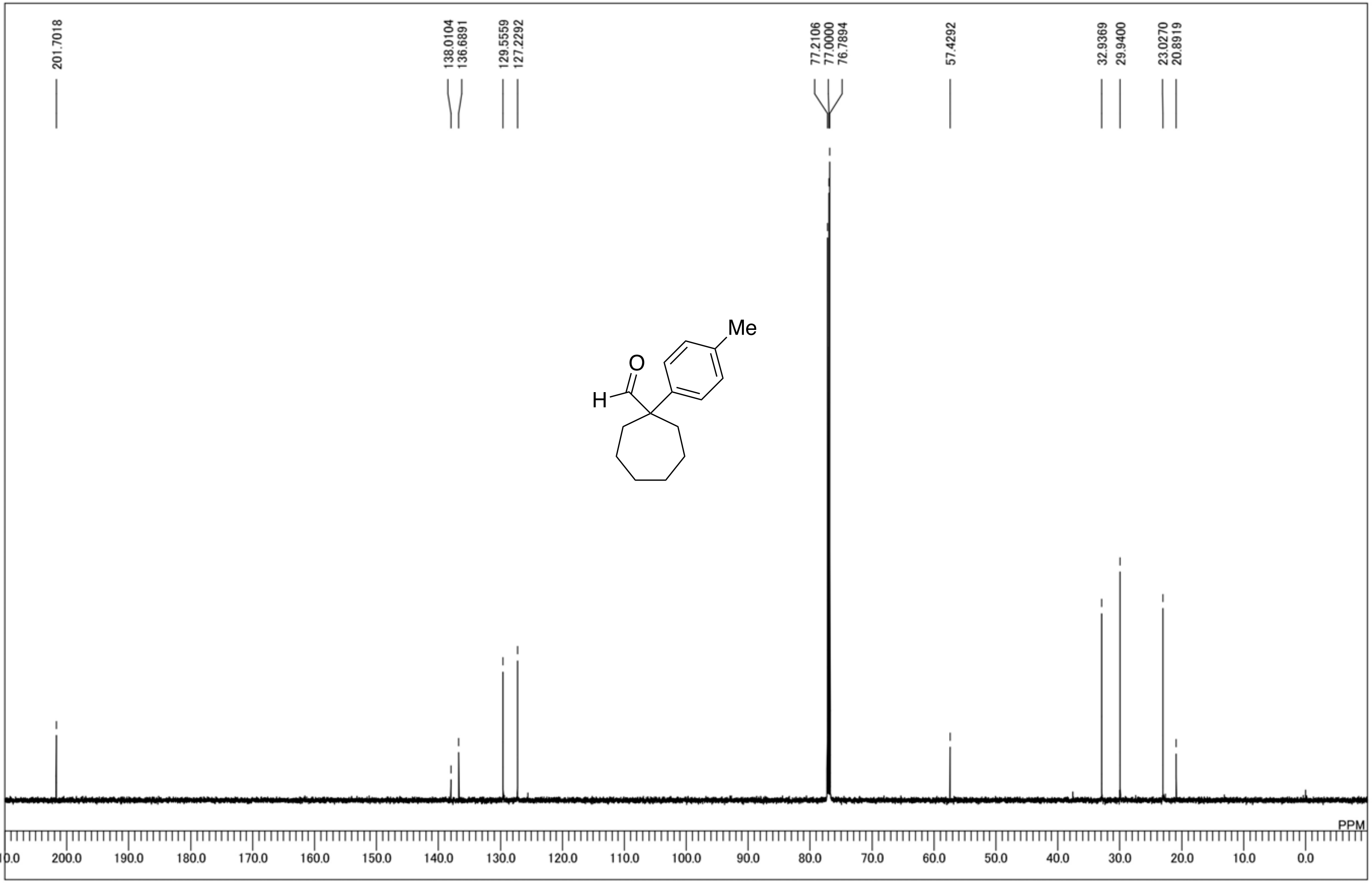

Supplementary Figure 82. ^{13}C NMR spectrum of **2b** (150.9 MHz, $CDCl_3$)

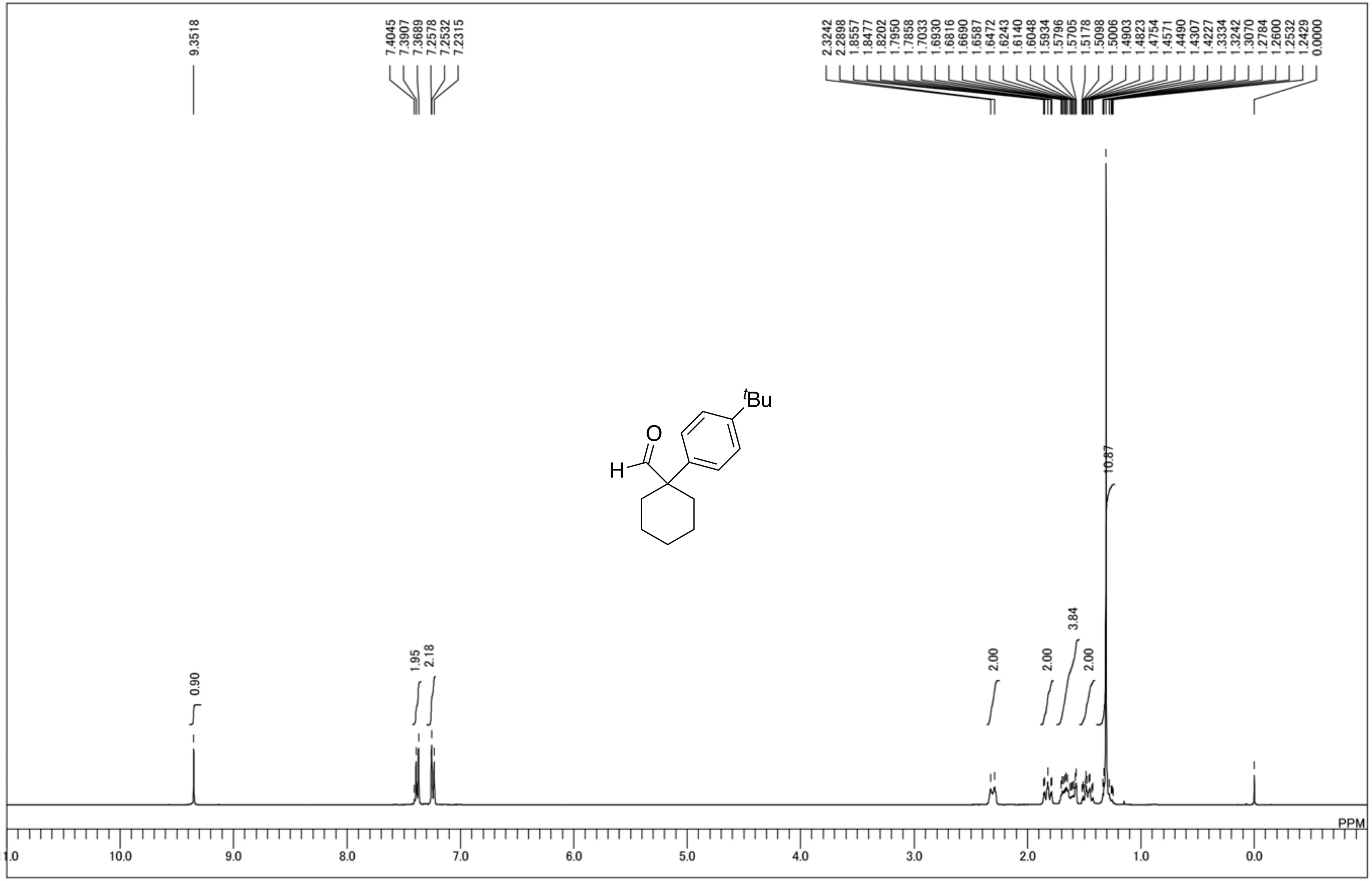

Supplementary Figure 83. ^{1}H NMR spectrum of **2c** (400 MHz, $CDCl_3$)

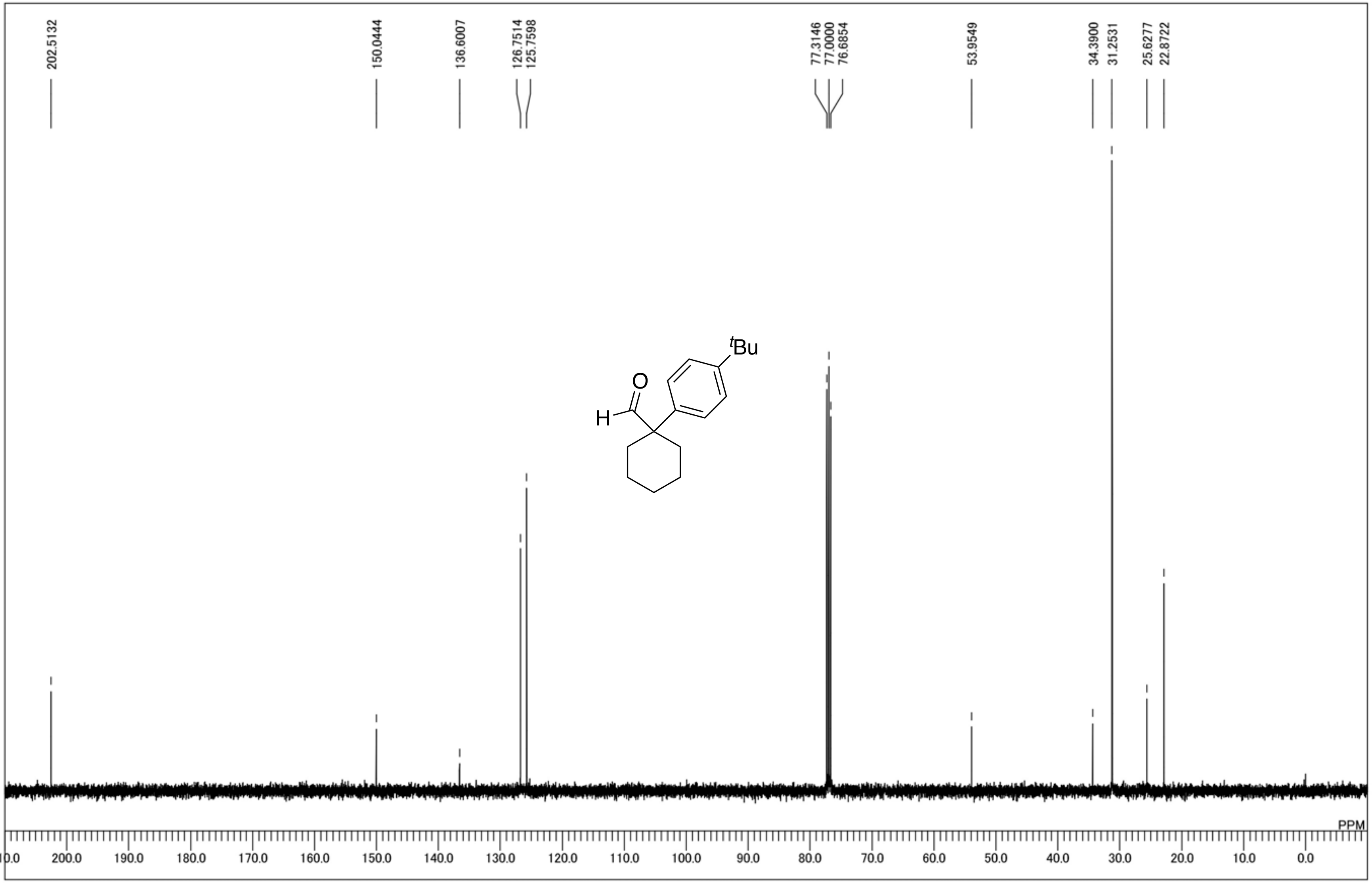

Supplementary Figure 84. ^{13}C NMR spectrum of **2c** (100.6 MHz, $CDCl_3$)

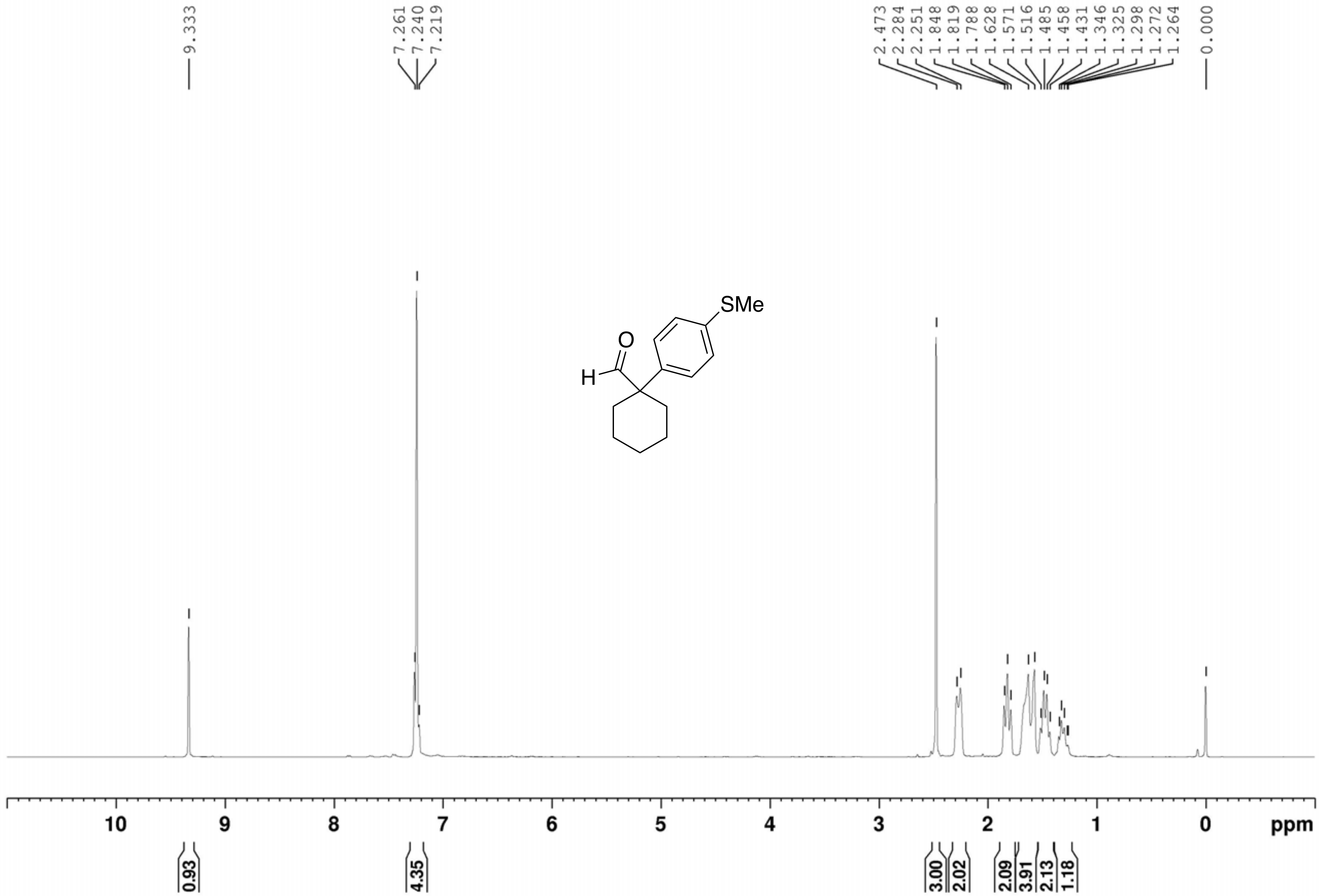

Supplementary Figure 85. ^{1}H NMR spectrum of **2d** (400 MHz, $CDCl_3$)

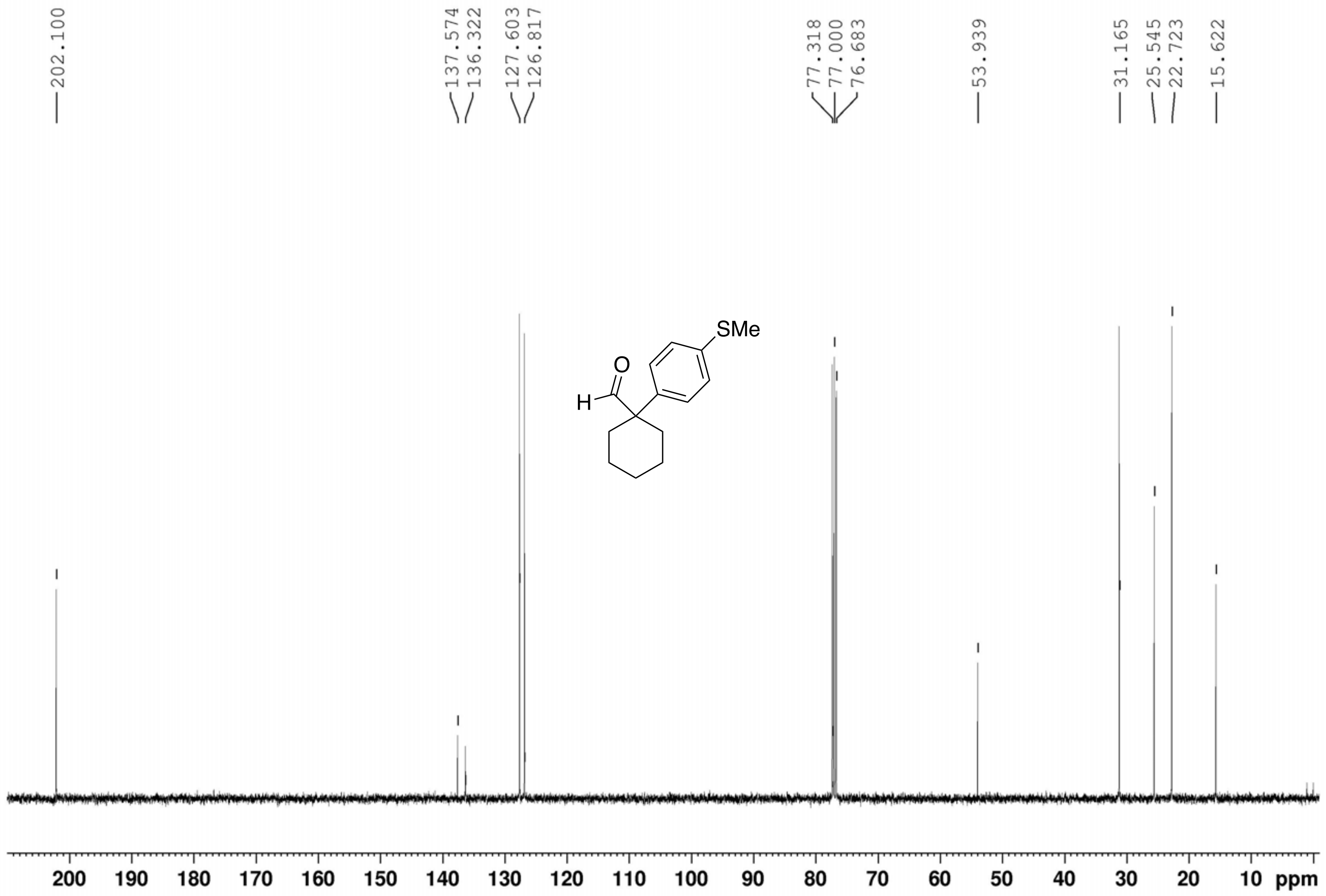

Supplementary Figure 86. ^{13}C NMR spectrum of **2d** (100.6 MHz, $CDCl_3$)

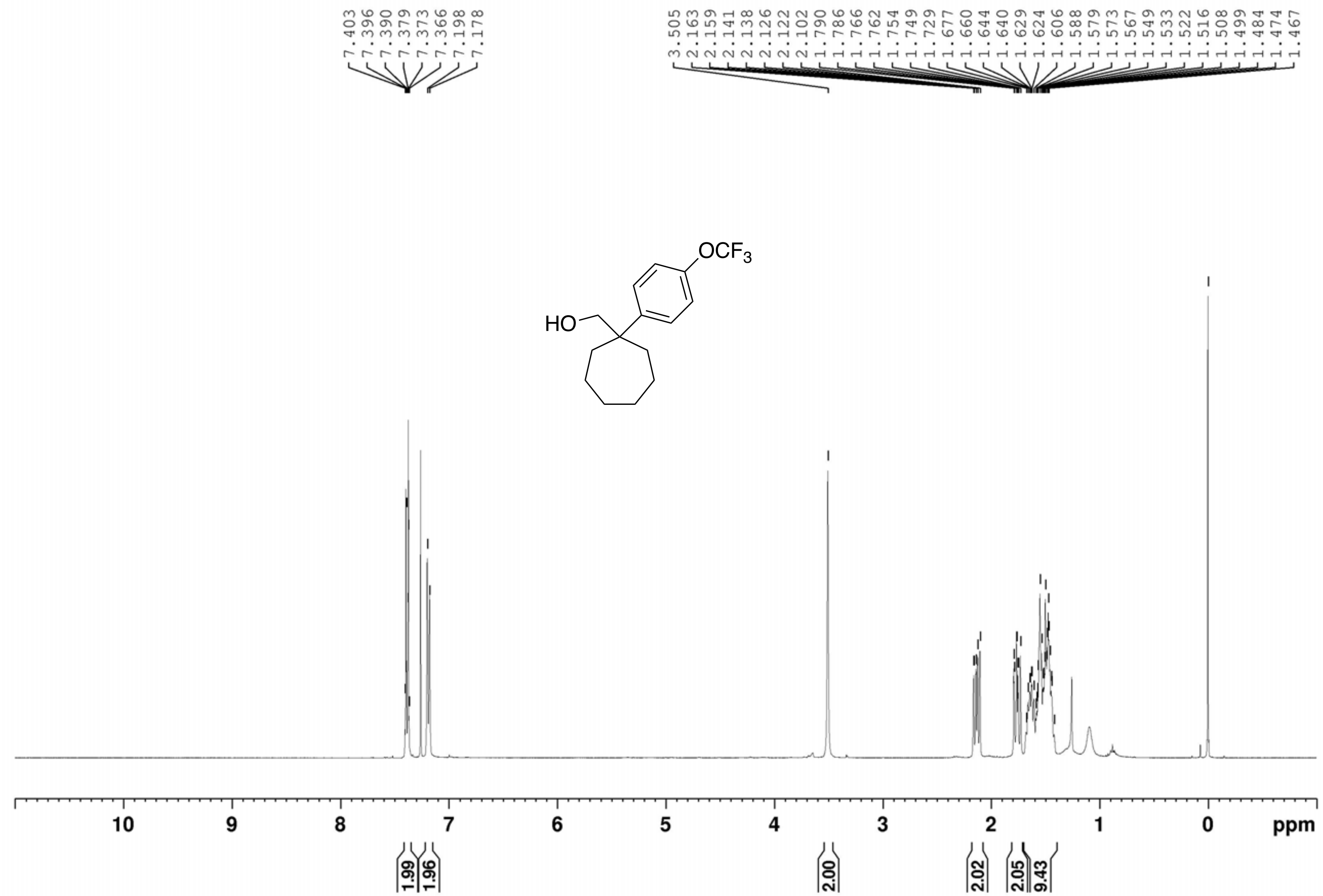

Supplementary Figure 87. ^{1}H NMR spectrum of **2e** (400 MHz, $CDCl_3$)

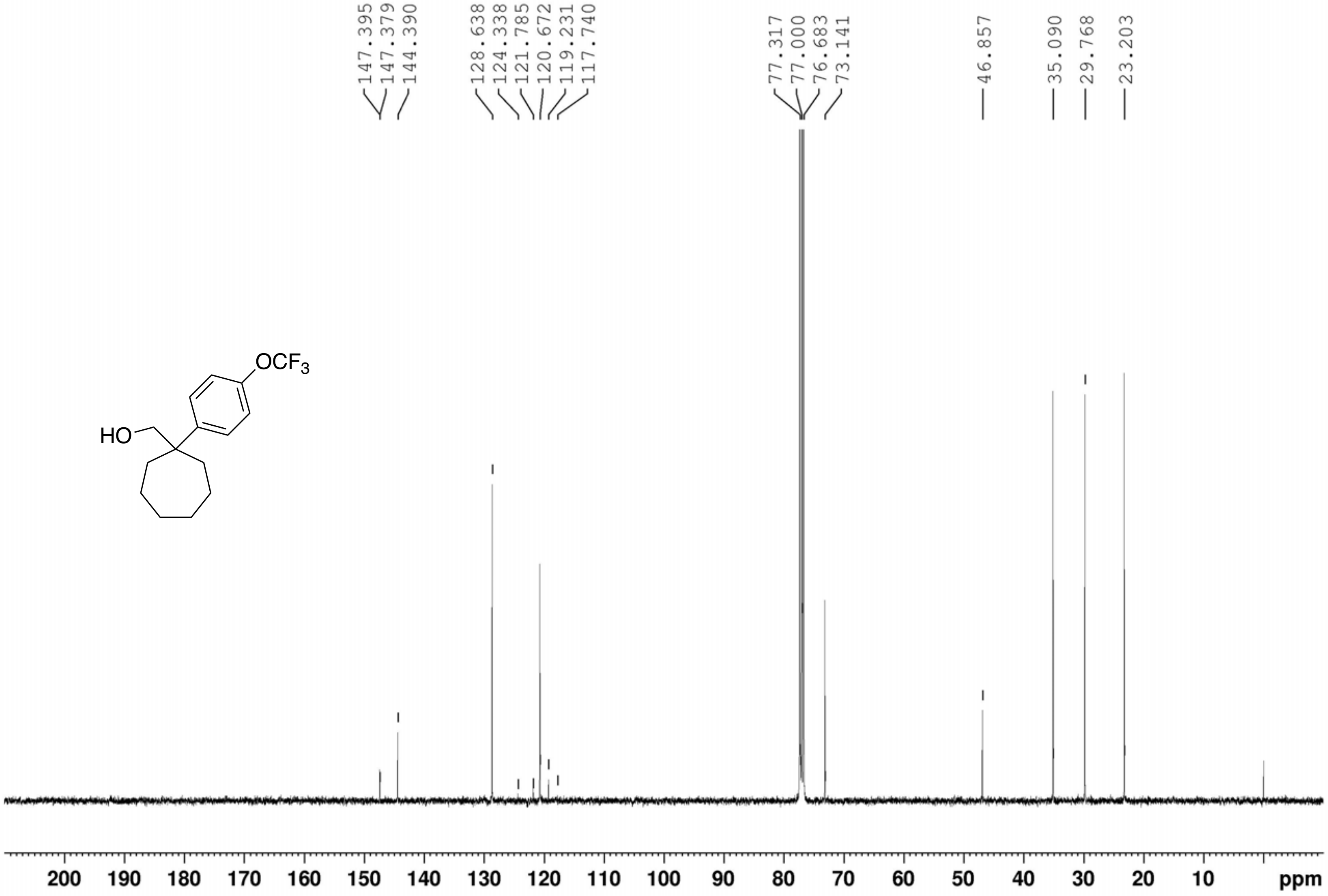

Supplementary Figure 88. ^{13}C NMR spectrum of **2e** (100.6 MHz, $CDCl_3$)

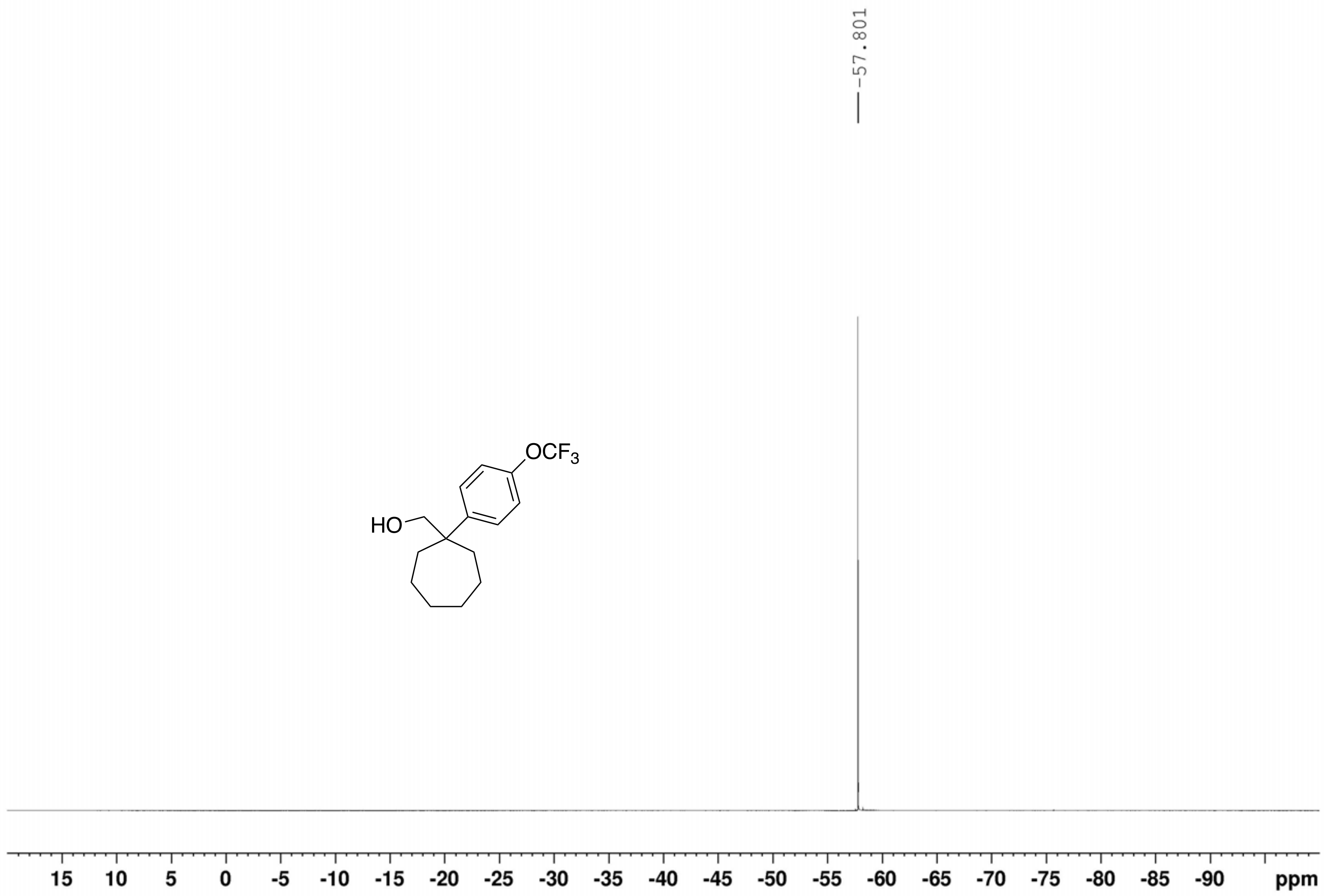

Supplementary Figure 89. ^{19}F NMR spectrum of **2e** (376 MHz, $CDCl_3$)

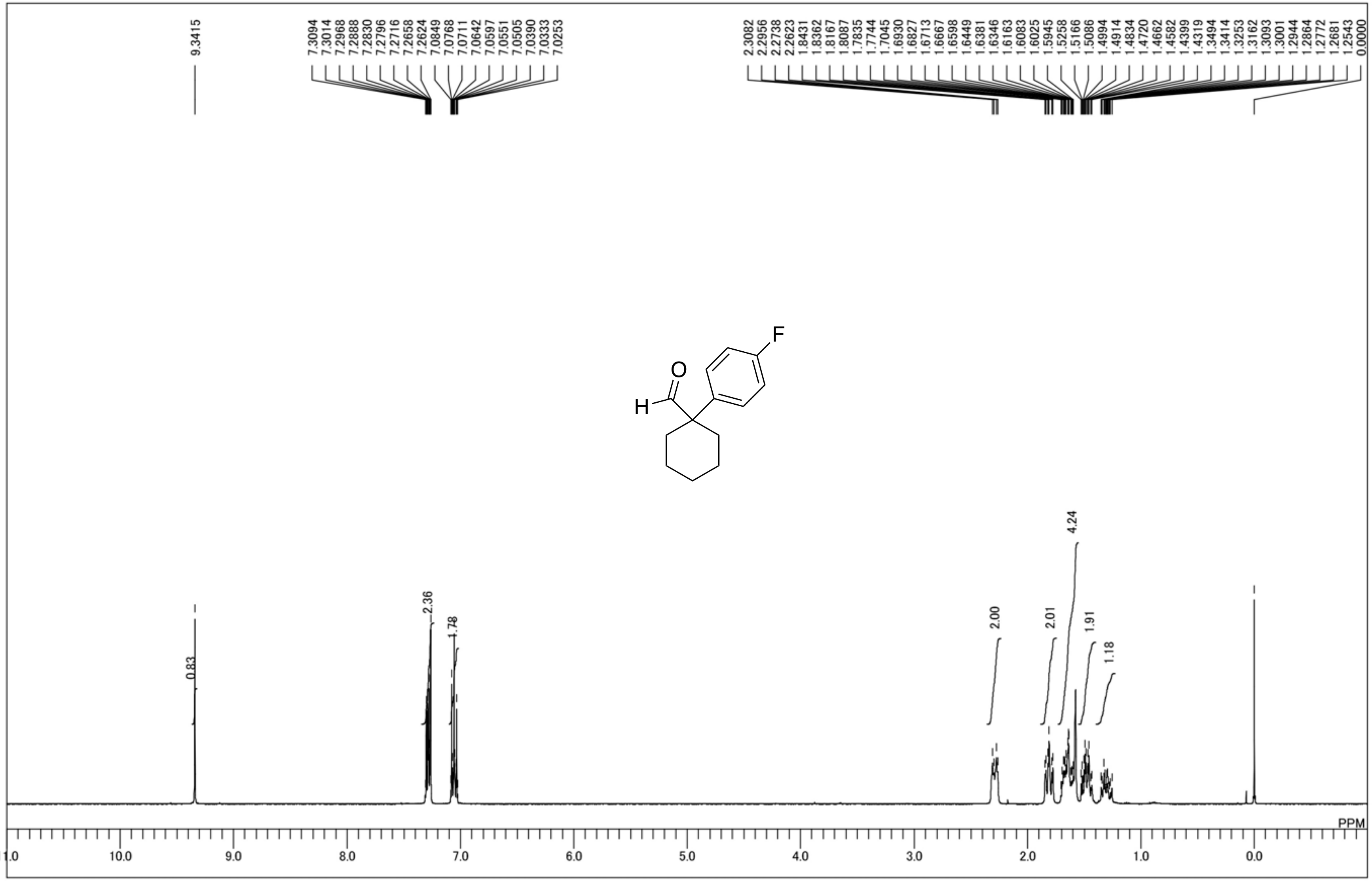

Supplementary Figure 90. ^{1}H NMR spectrum of **2f** (400 MHz, $CDCl_3$)

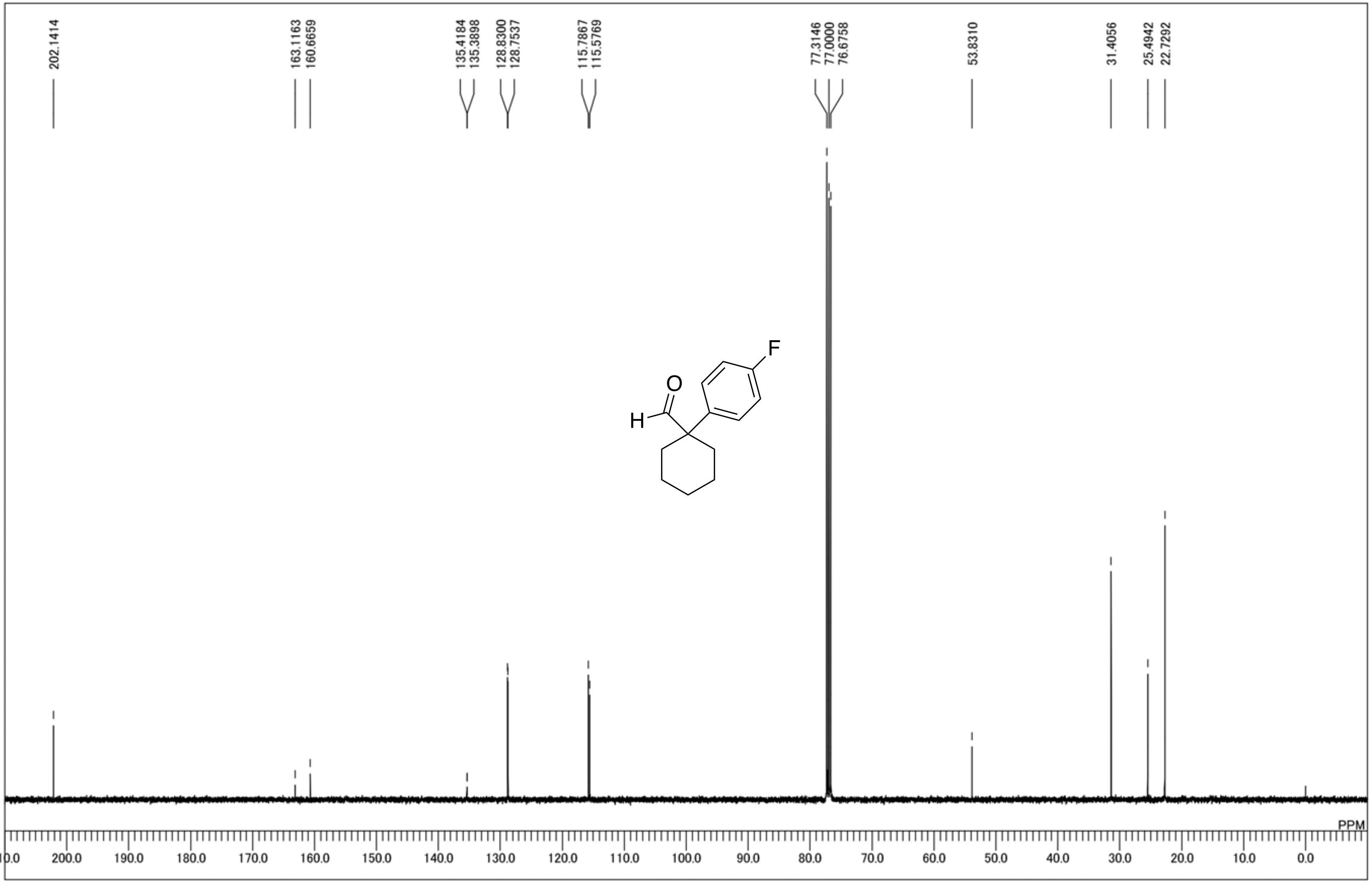

Supplementary Figure 91. ^{13}C NMR spectrum of **2f** (100.6 MHz, $CDCl_3$)

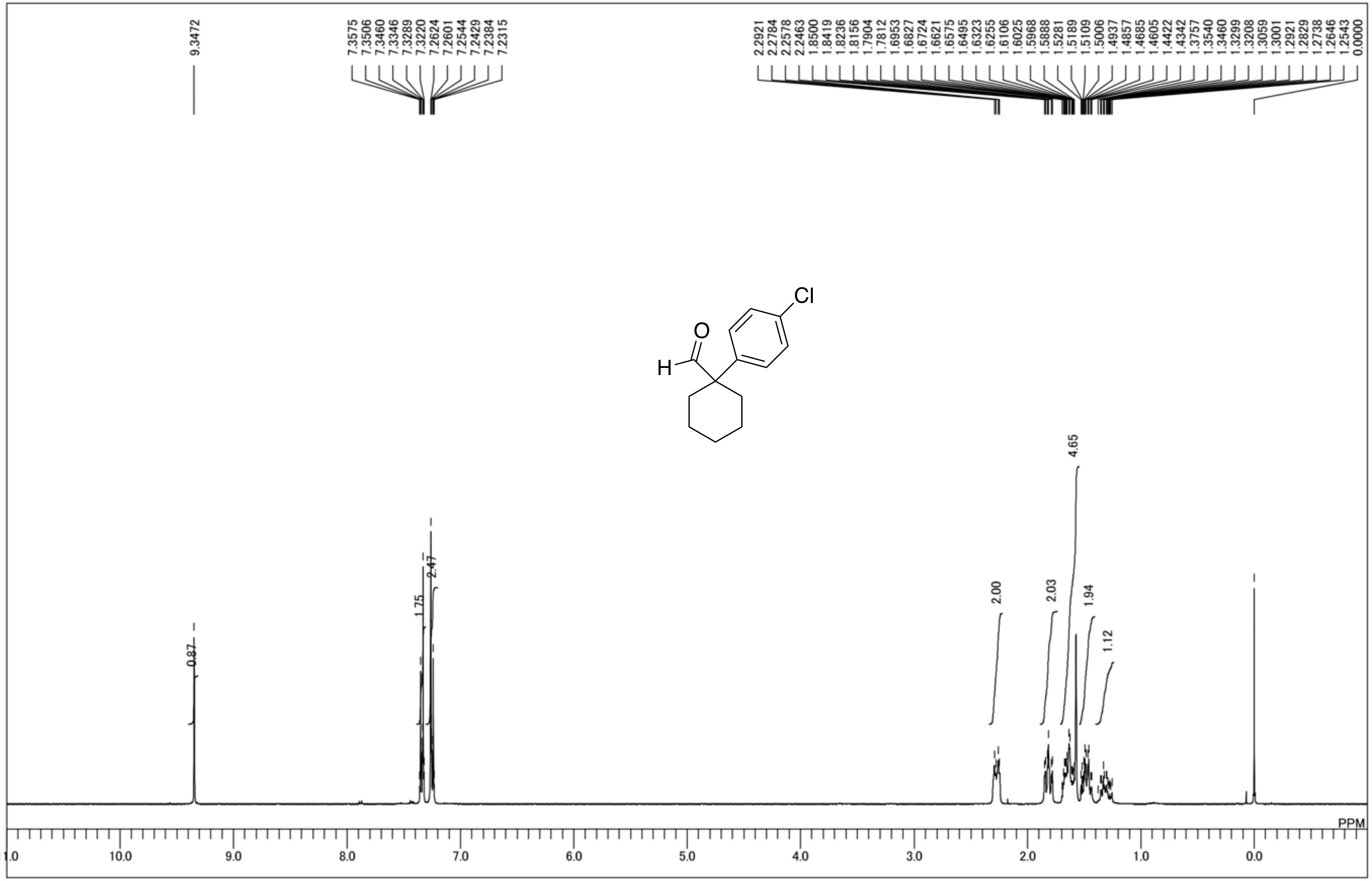

Supplementary Figure 92. ^{1}H NMR spectrum of **2g** (400 MHz, $CDCl_3$)

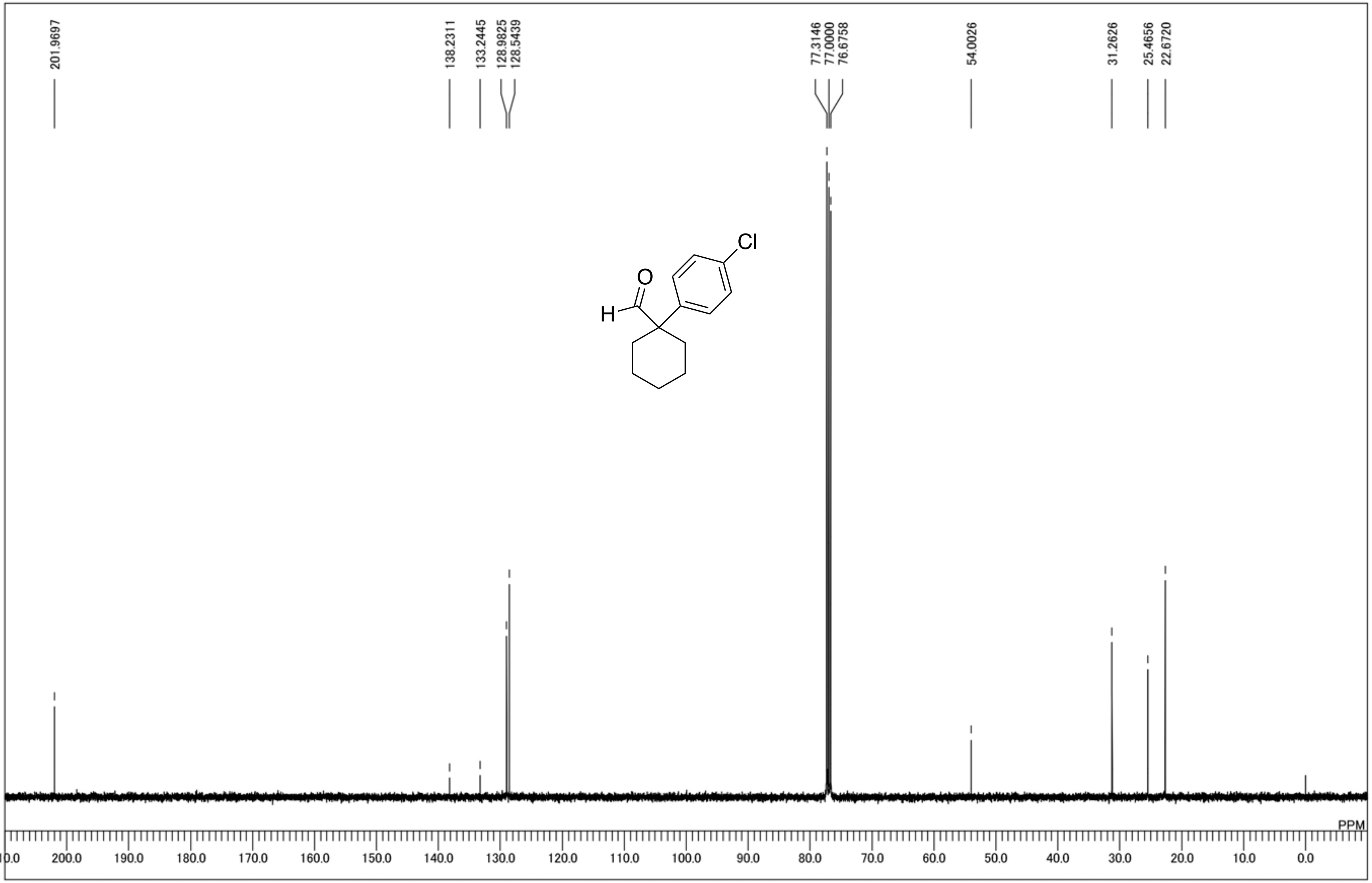

Supplementary Figure 93. ^{13}C NMR spectrum of **2g** (100.6 MHz, $CDCl_3$)

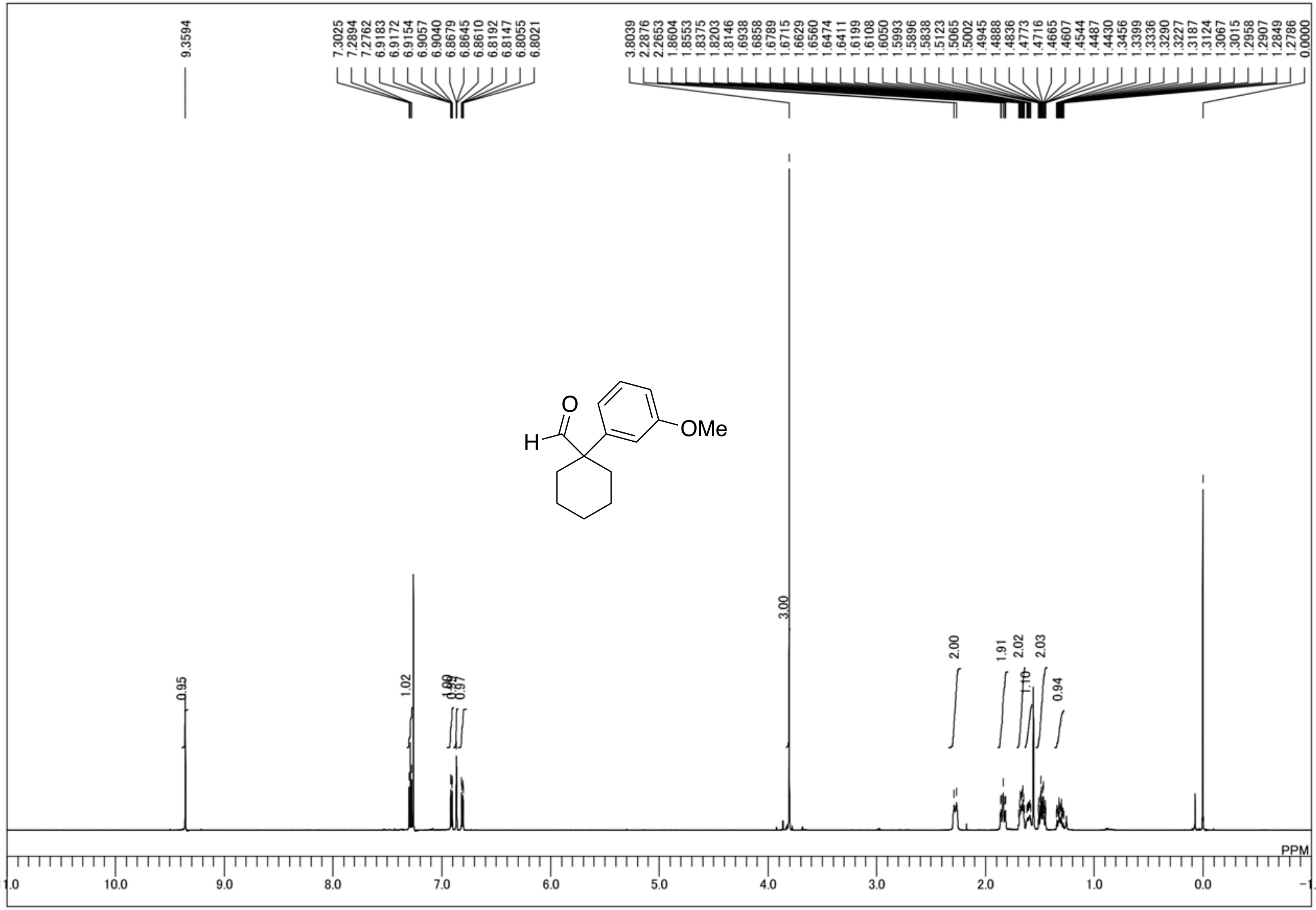

Supplementary Figure 94. ^{1}H NMR spectrum of **2h** (600 MHz, $CDCl_3$)

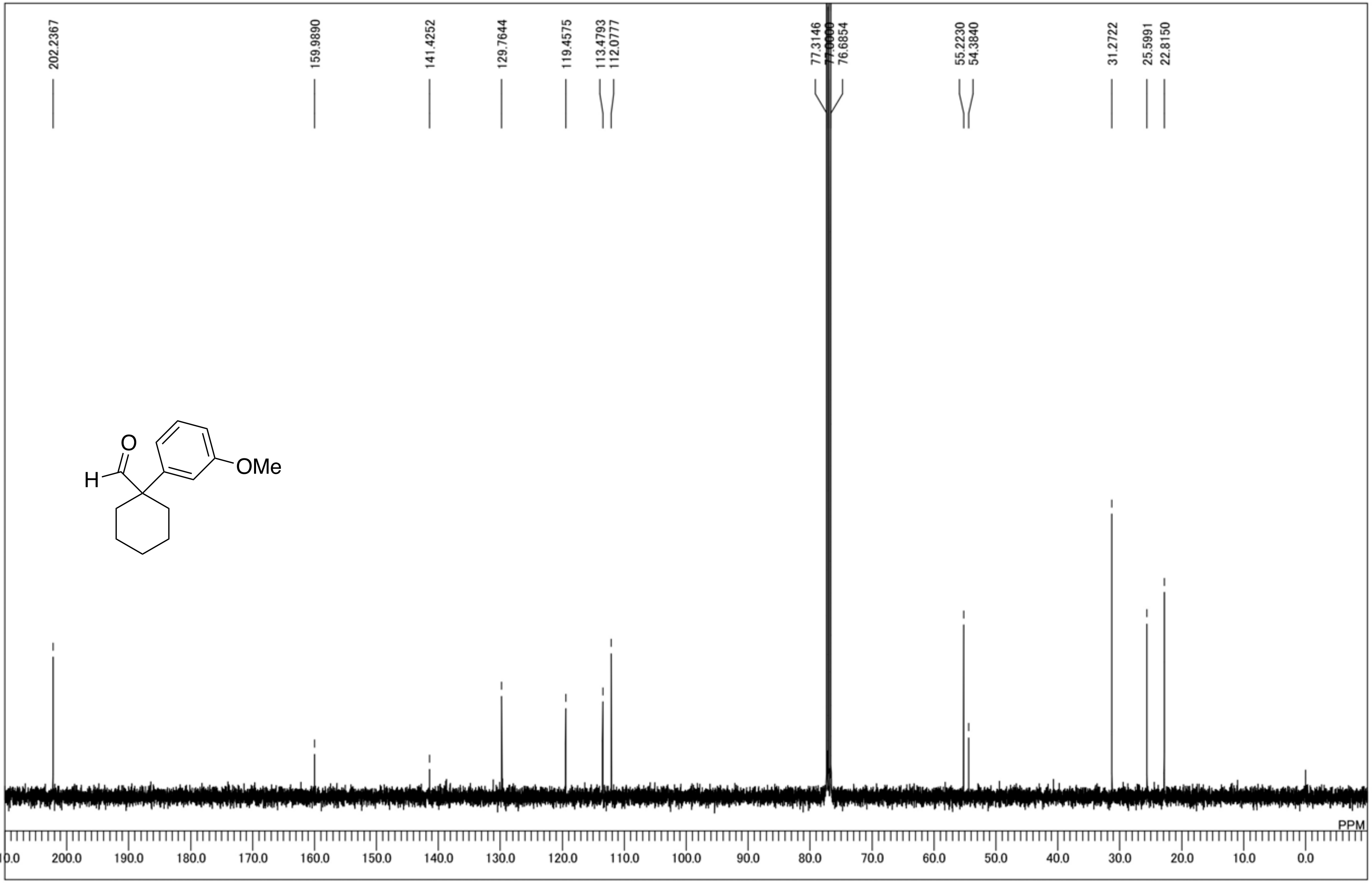

Supplementary Figure 95. ^{13}C NMR spectrum of **2h** (100.6 MHz, $CDCl_3$)

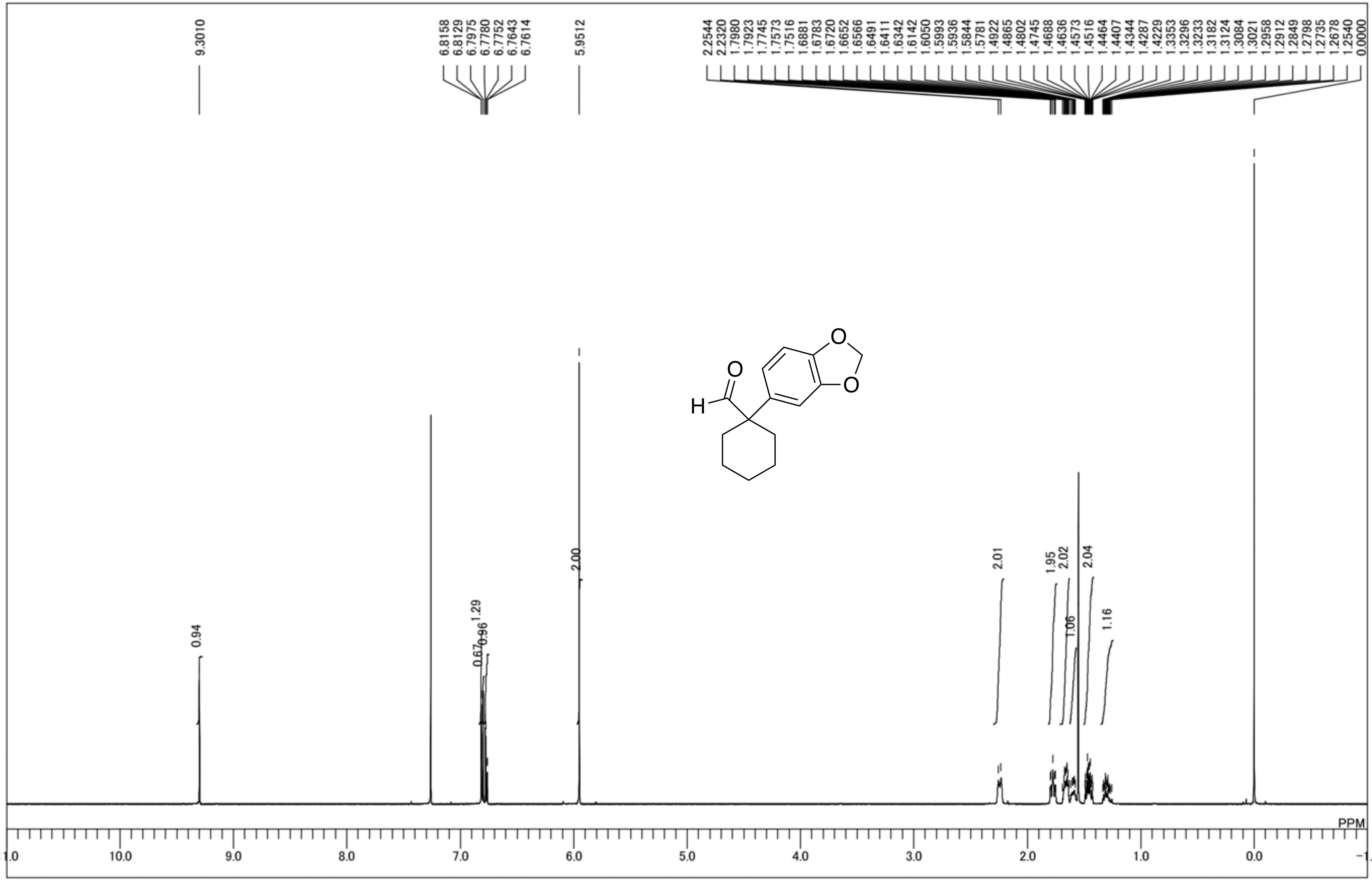

Supplementary Figure 96. ^{1}H NMR spectrum of **2i** (600 MHz, $CDCl_3$)

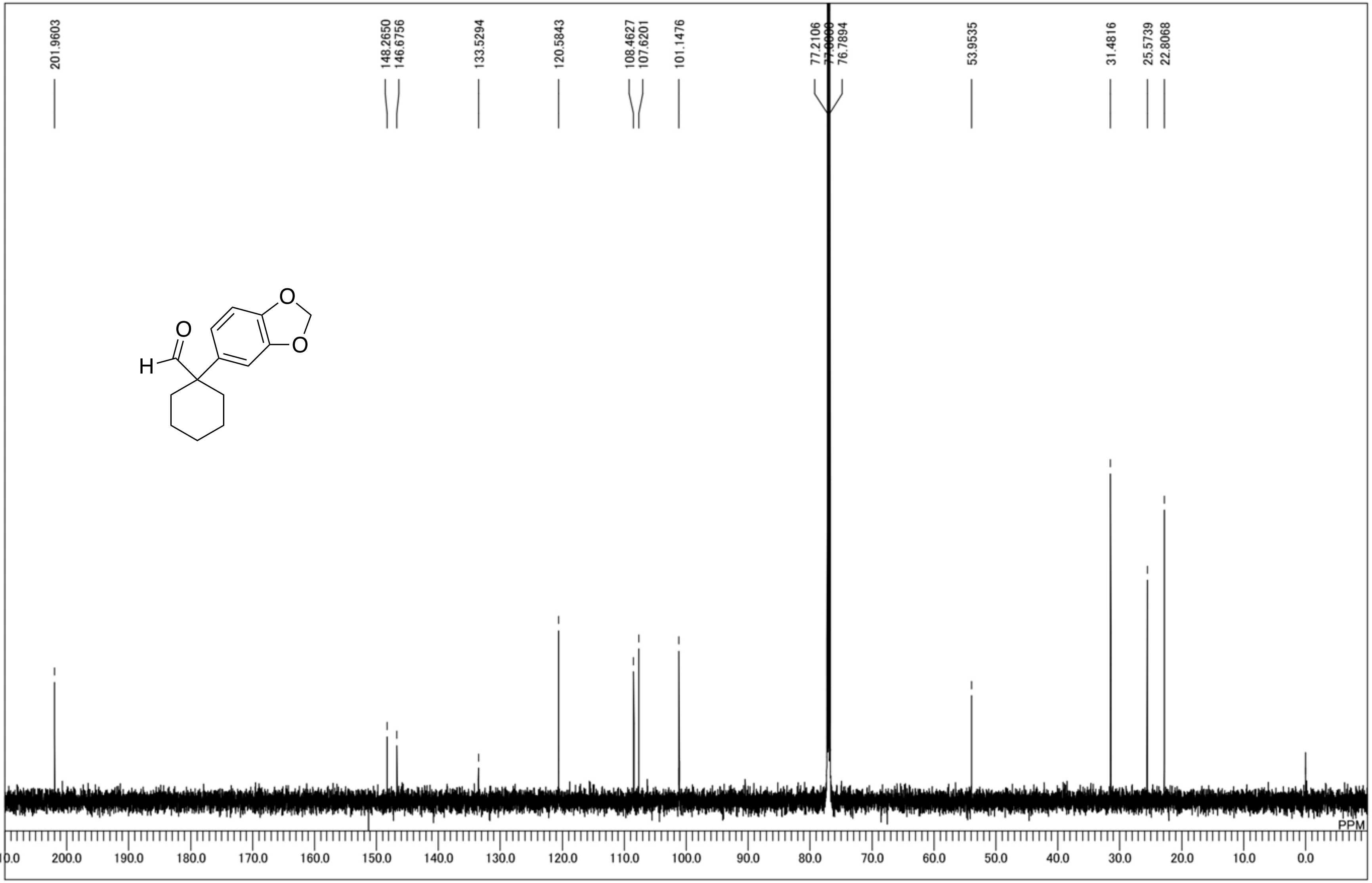

Supplementary Figure 97. ^{13}C NMR spectrum of **2i** (150.9 MHz, $CDCl_3$)

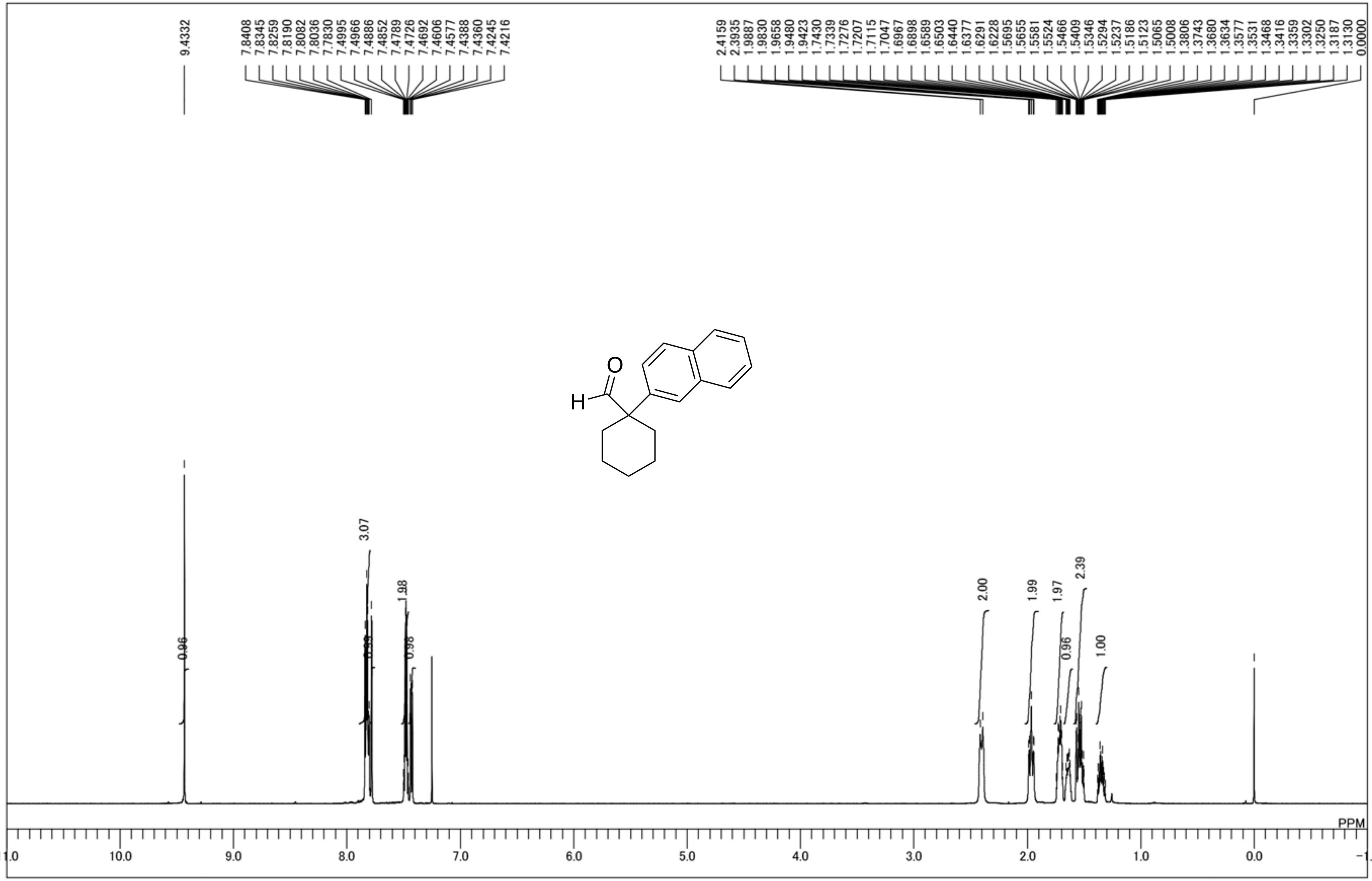

Supplementary Figure 98. ^{1}H NMR spectrum of **2j** (600 MHz, $CDCl_3$)

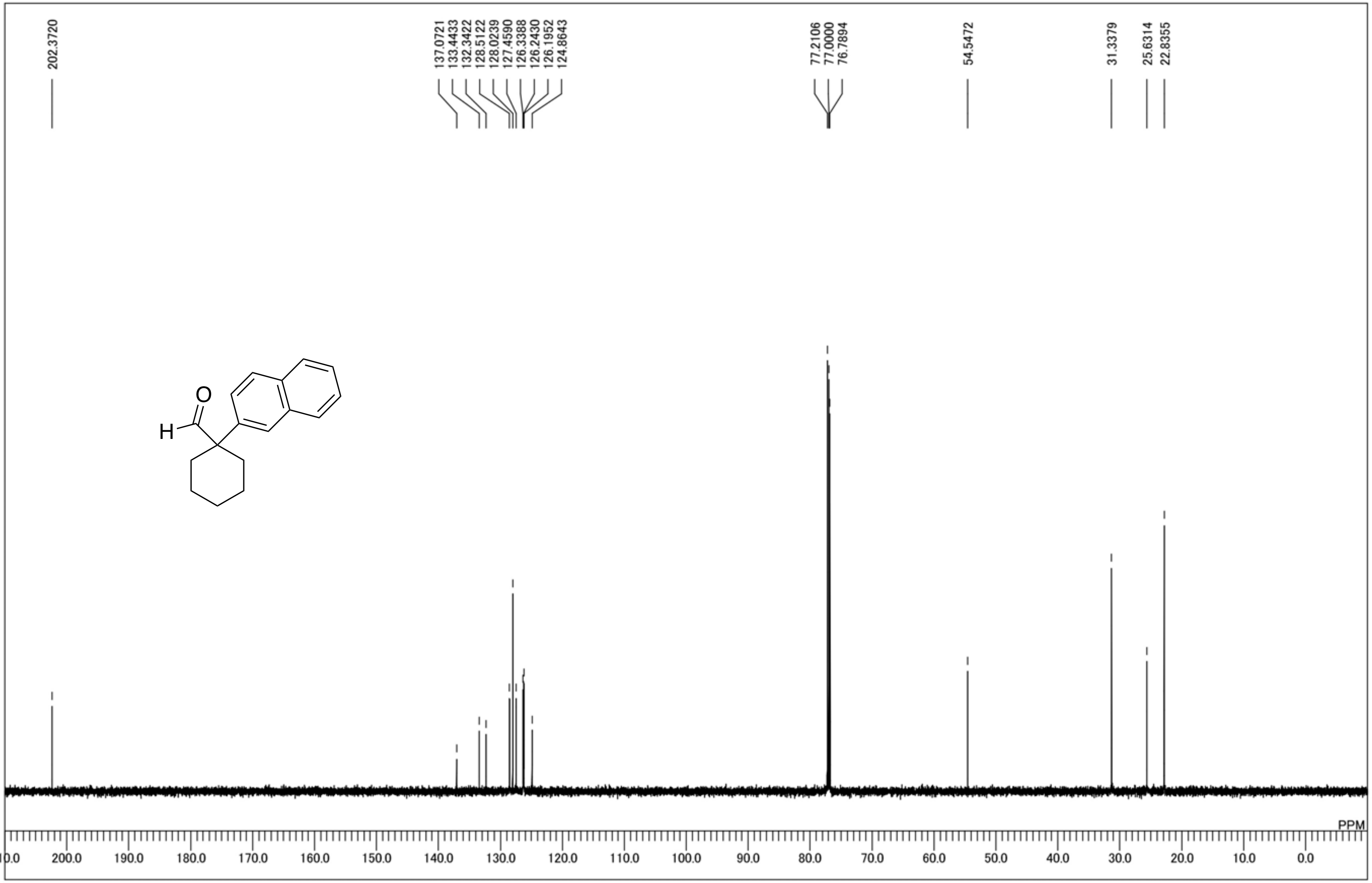

Supplementary Figure 99. ^{13}C NMR spectrum of **2j** (150.9 MHz, $CDCl_3$)

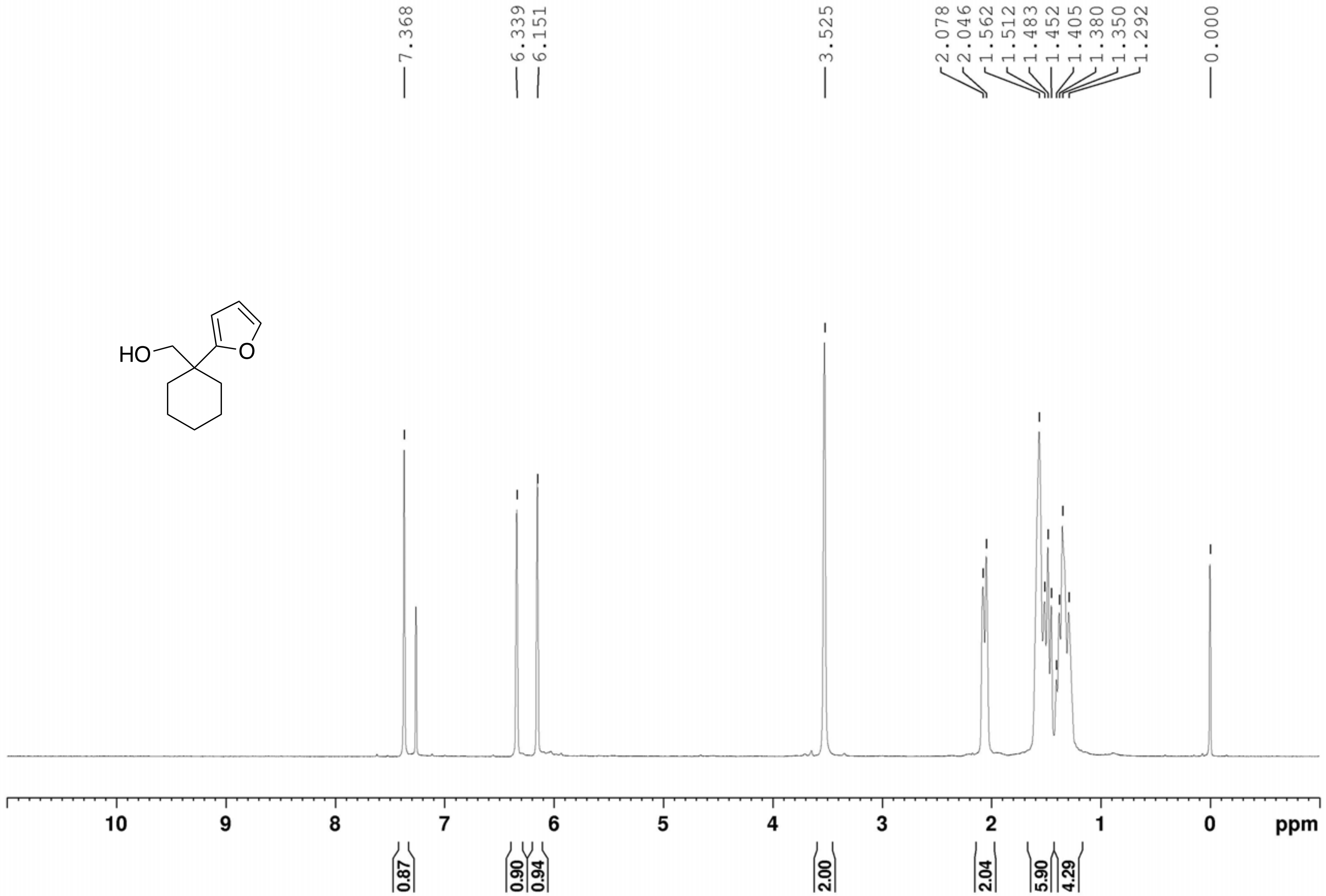

Supplementary Figure 100. ^{1}H NMR spectrum of **2k** (400 MHz, $CDCl_3$)

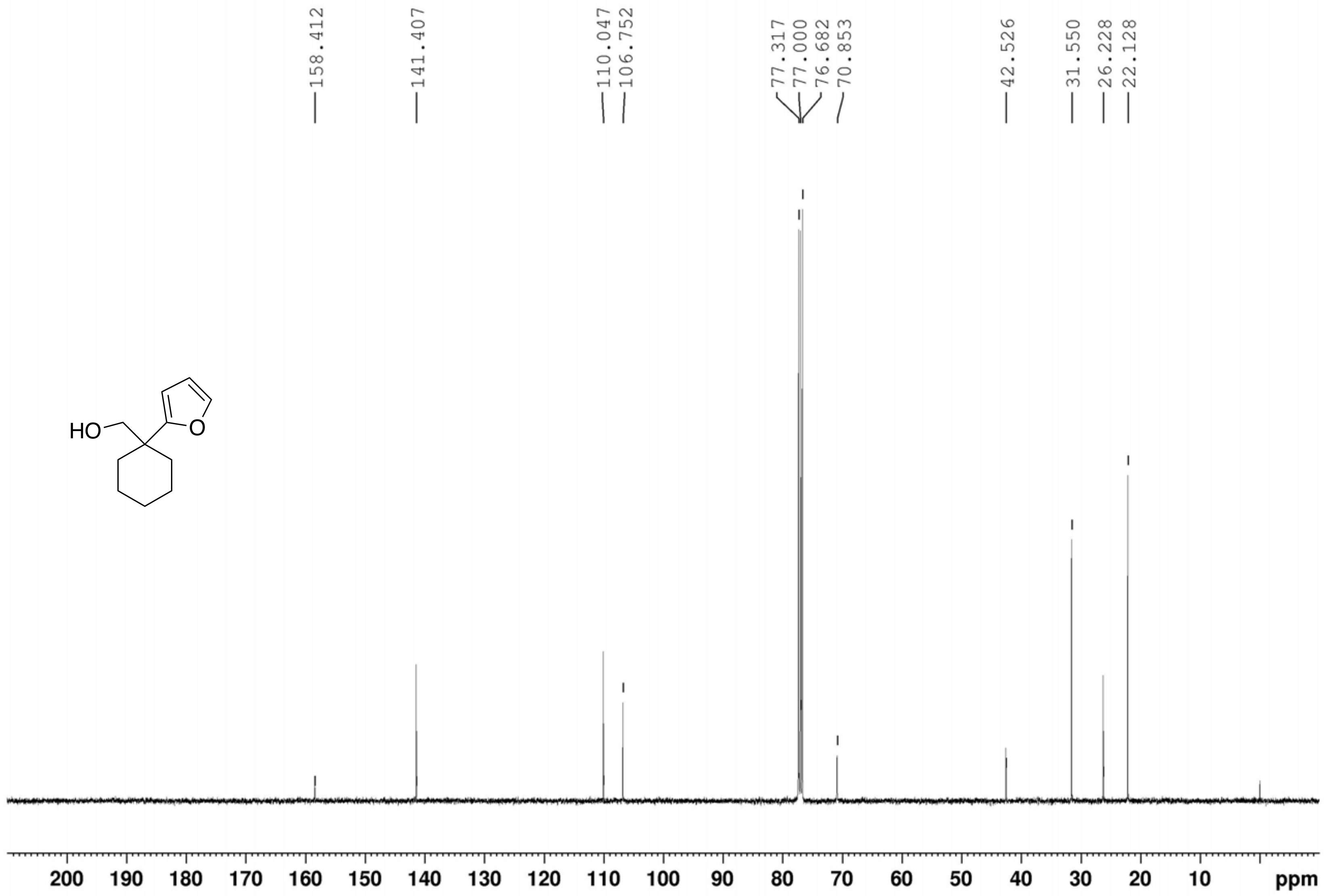

Supplementary Figure 101. ^{13}C NMR spectrum of **2k** (100.6 MHz, $CDCl_3$)

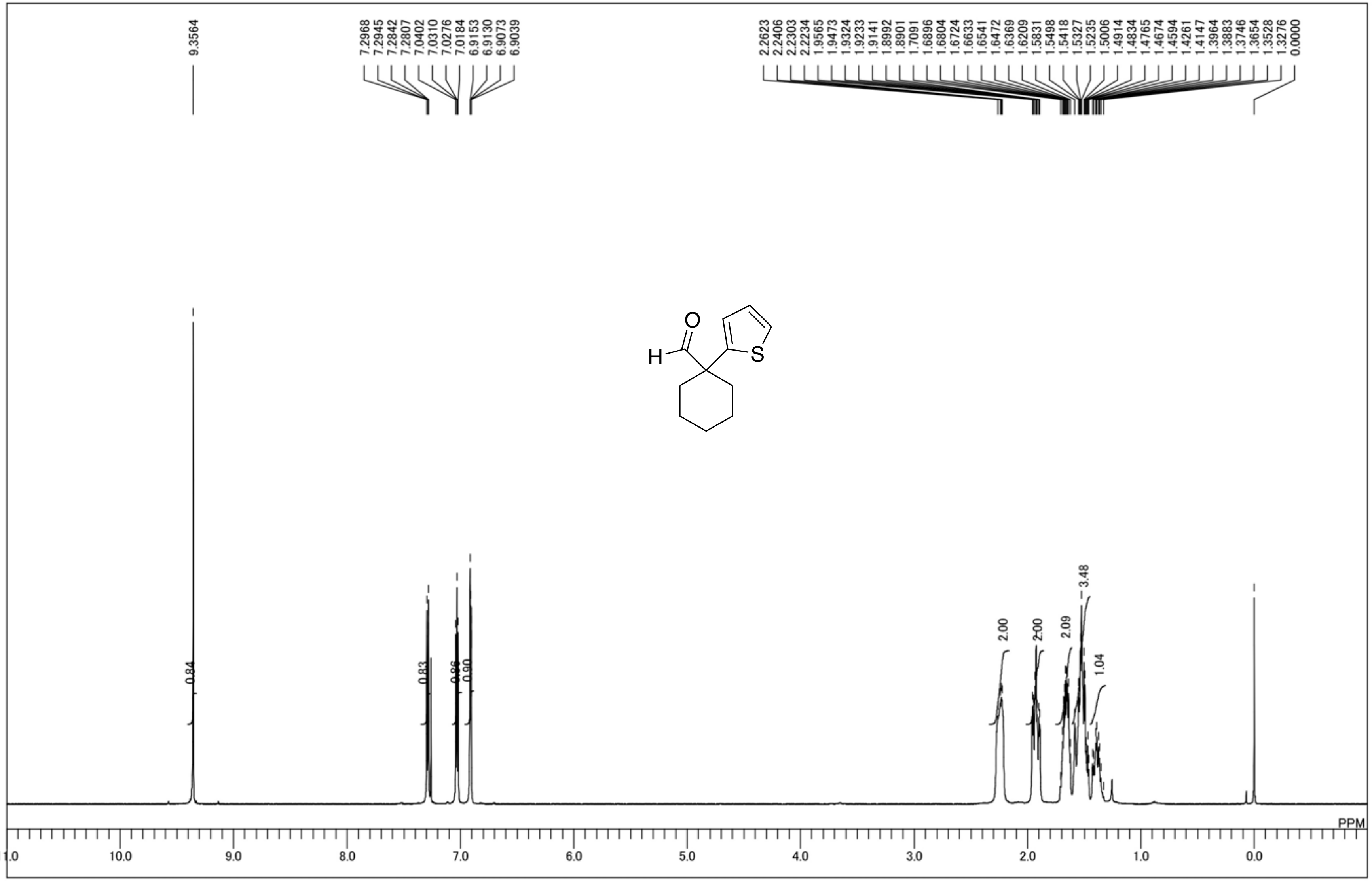

Supplementary Figure 102. ^{1}H NMR spectrum of **2l** (400 MHz, $CDCl_3$)

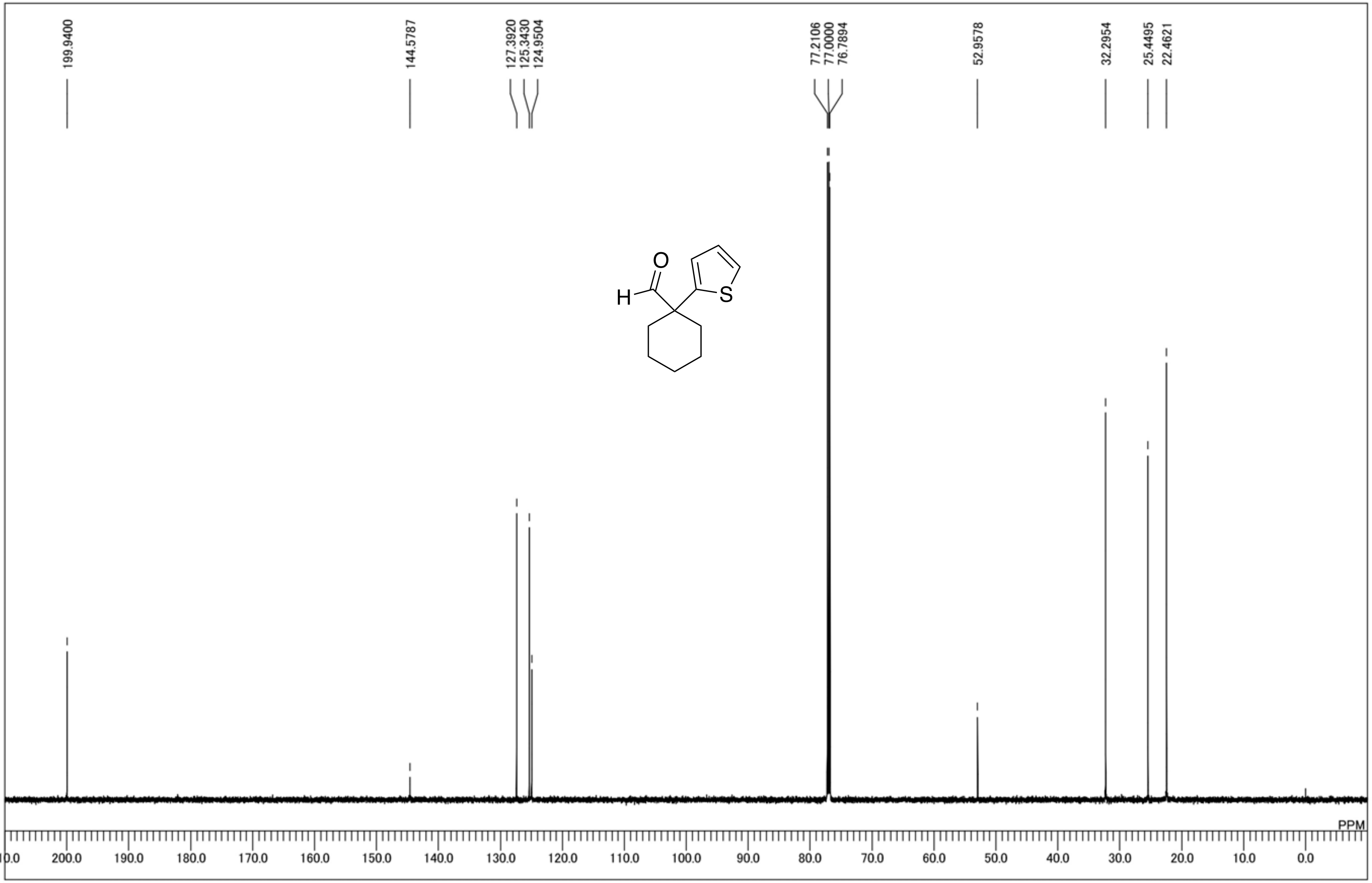

Supplementary Figure 103. ^{13}C NMR spectrum of **2l** (150.9 MHz, $CDCl_3$)

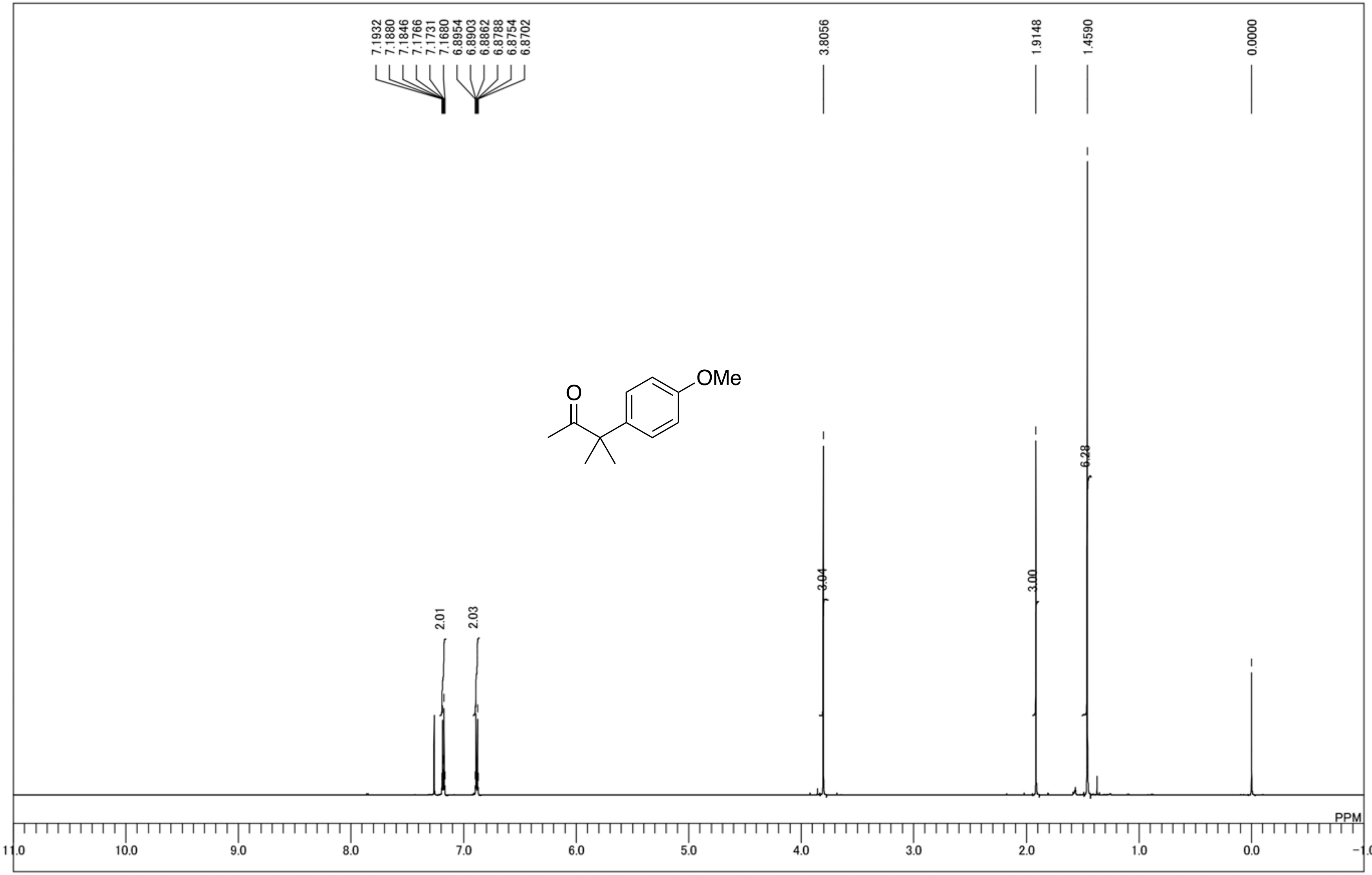

Supplementary Figure 104. ^{1}H NMR spectrum of **2m** (600 MHz, $CDCl_3$)

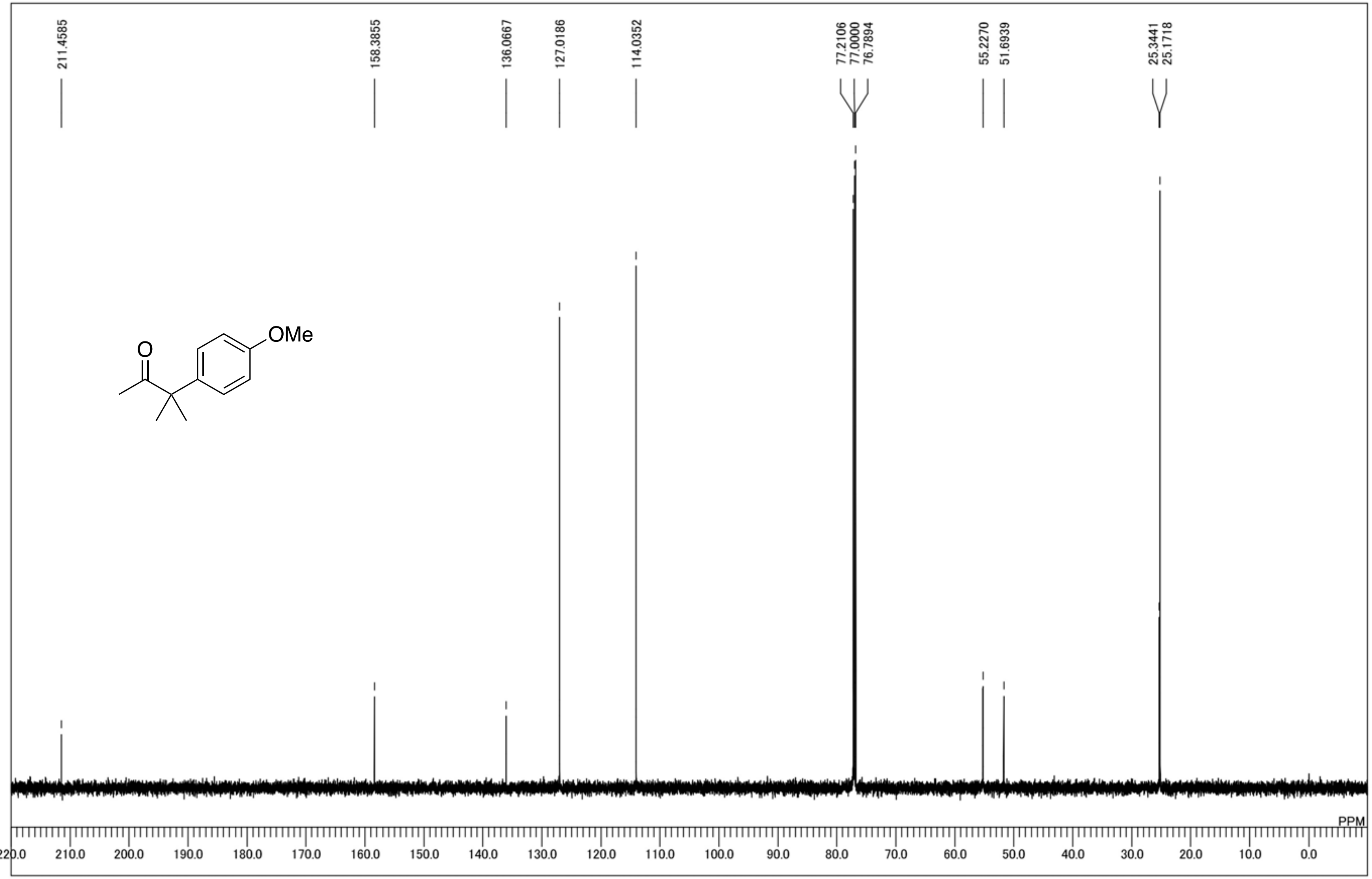

Supplementary Figure 105. ^{13}C NMR spectrum of **2m** (150.9 MHz, $CDCl_3$)

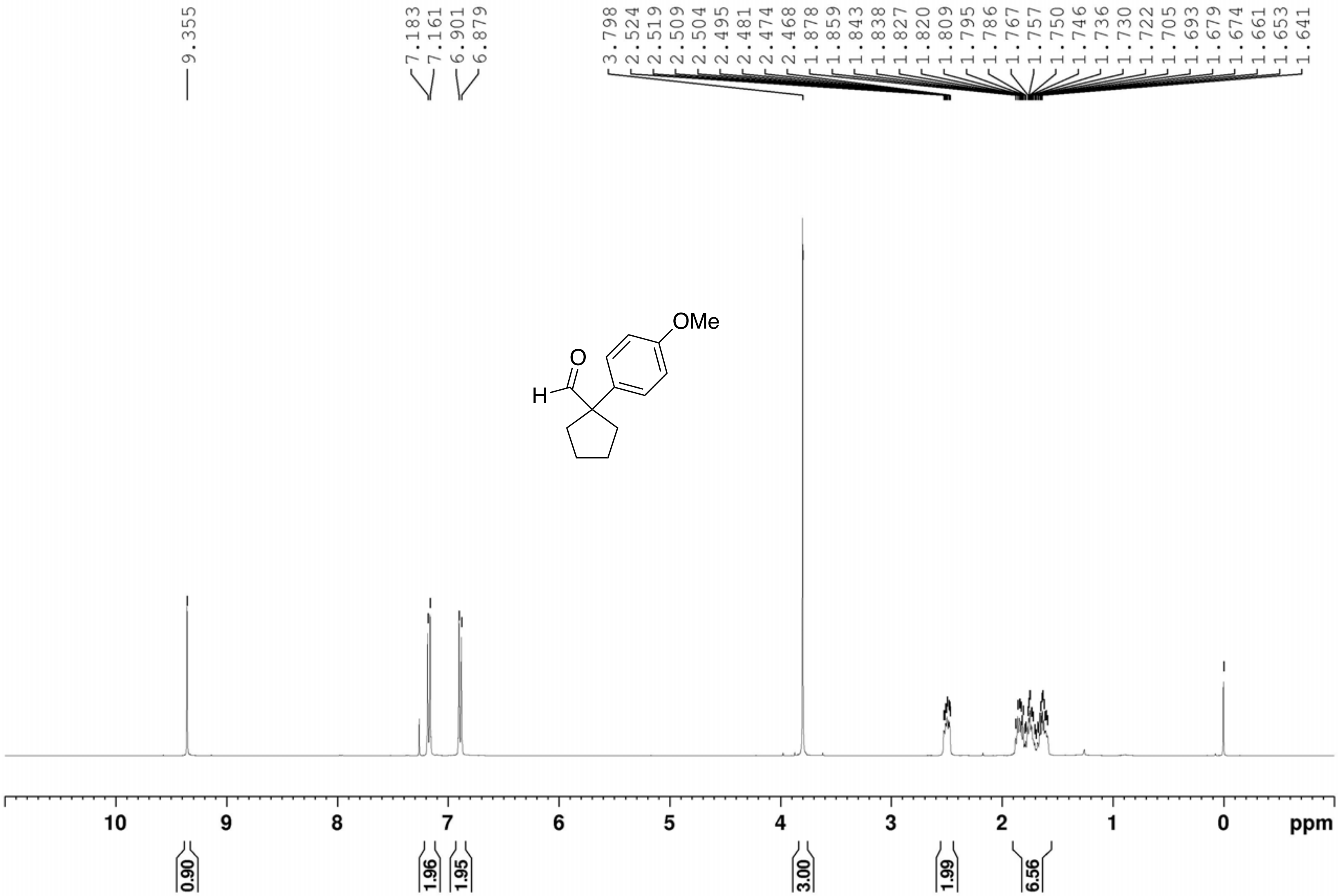

Supplementary Figure 106. ^{1}H NMR spectrum of **2n** (400 MHz, $CDCl_3$)

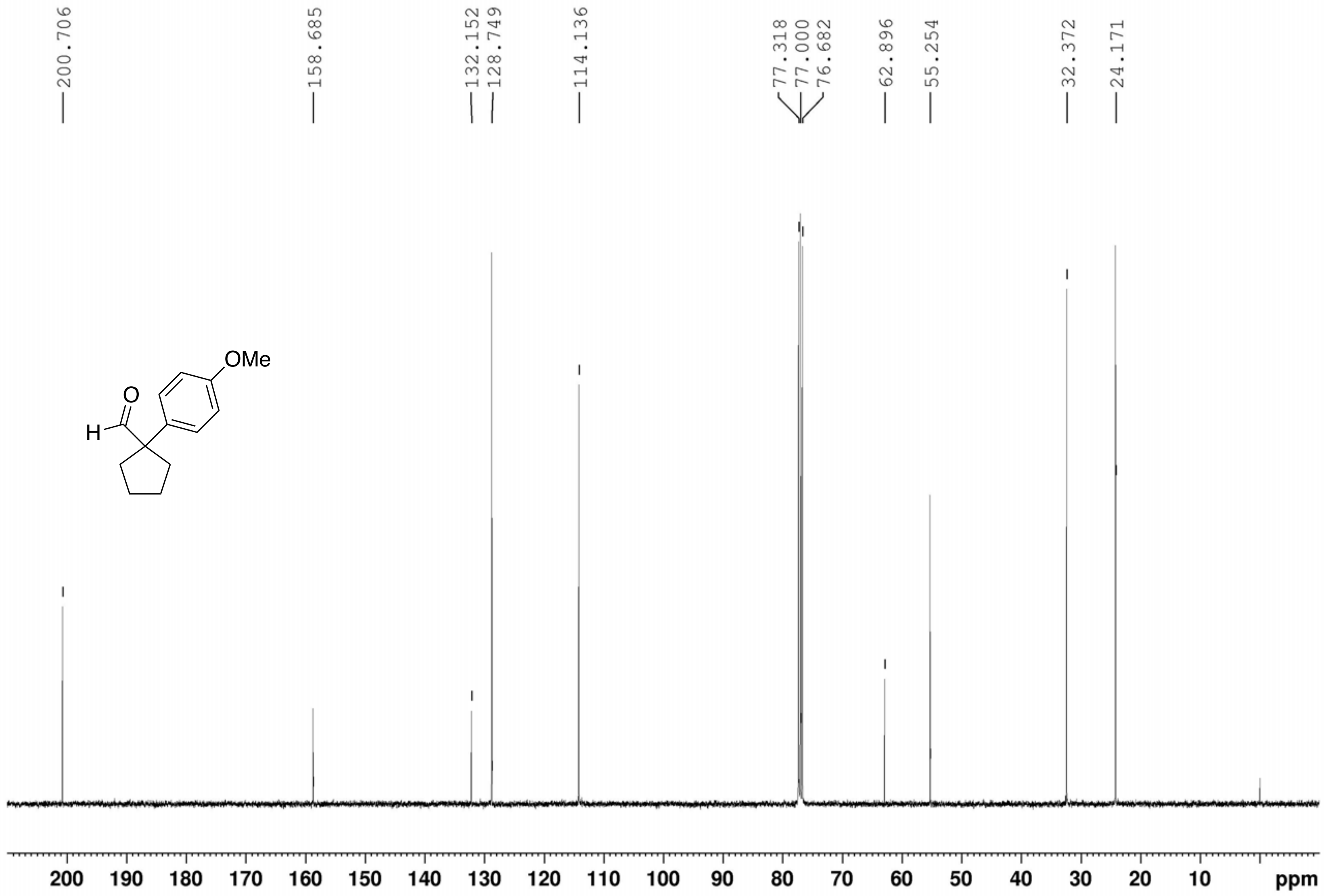

Supplementary Figure 107. ^{13}C NMR spectrum of **2n** (100.6 MHz, $CDCl_3$)

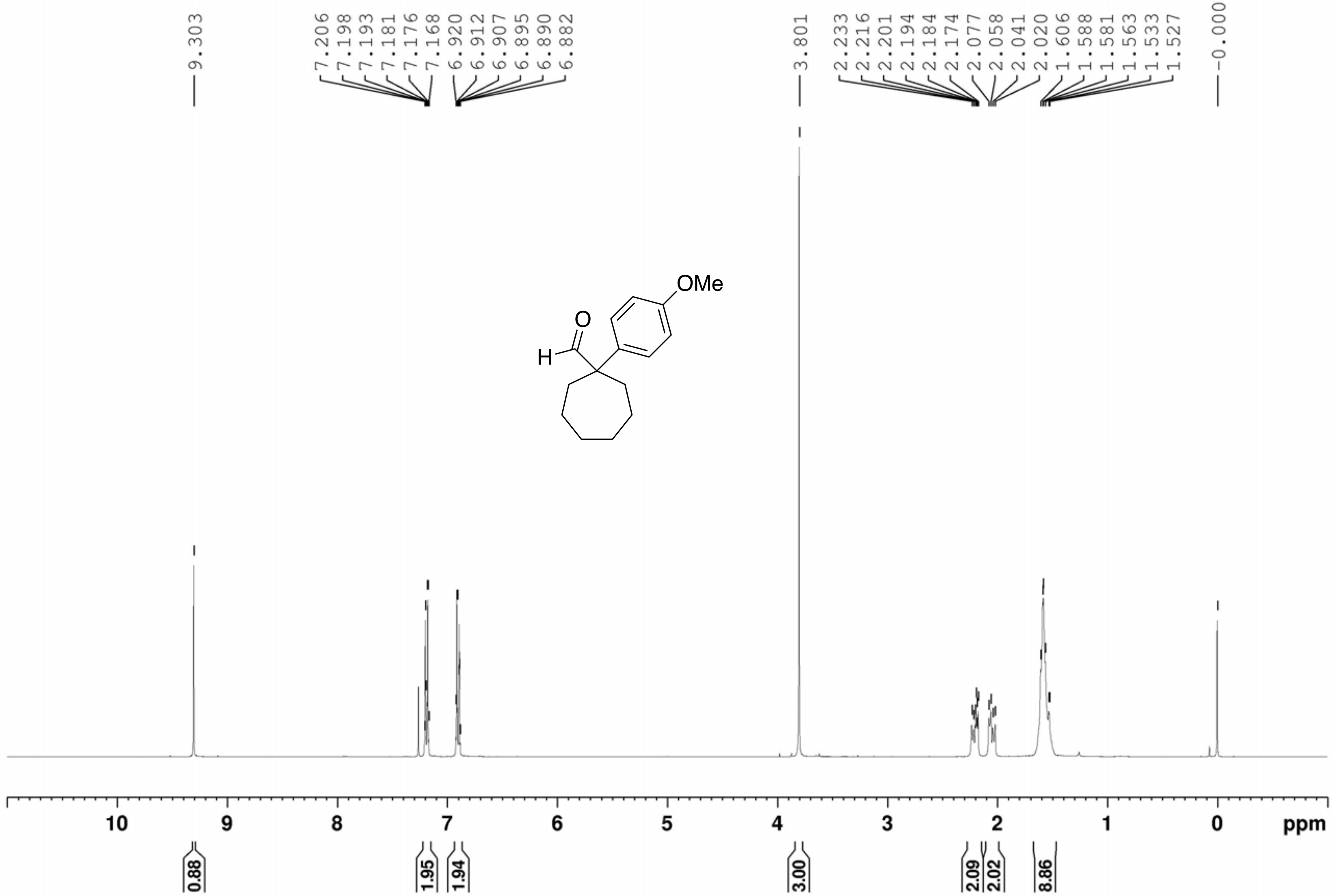

Supplementary Figure 108. ^{1}H NMR spectrum of **2o** (400 MHz, $CDCl_3$)

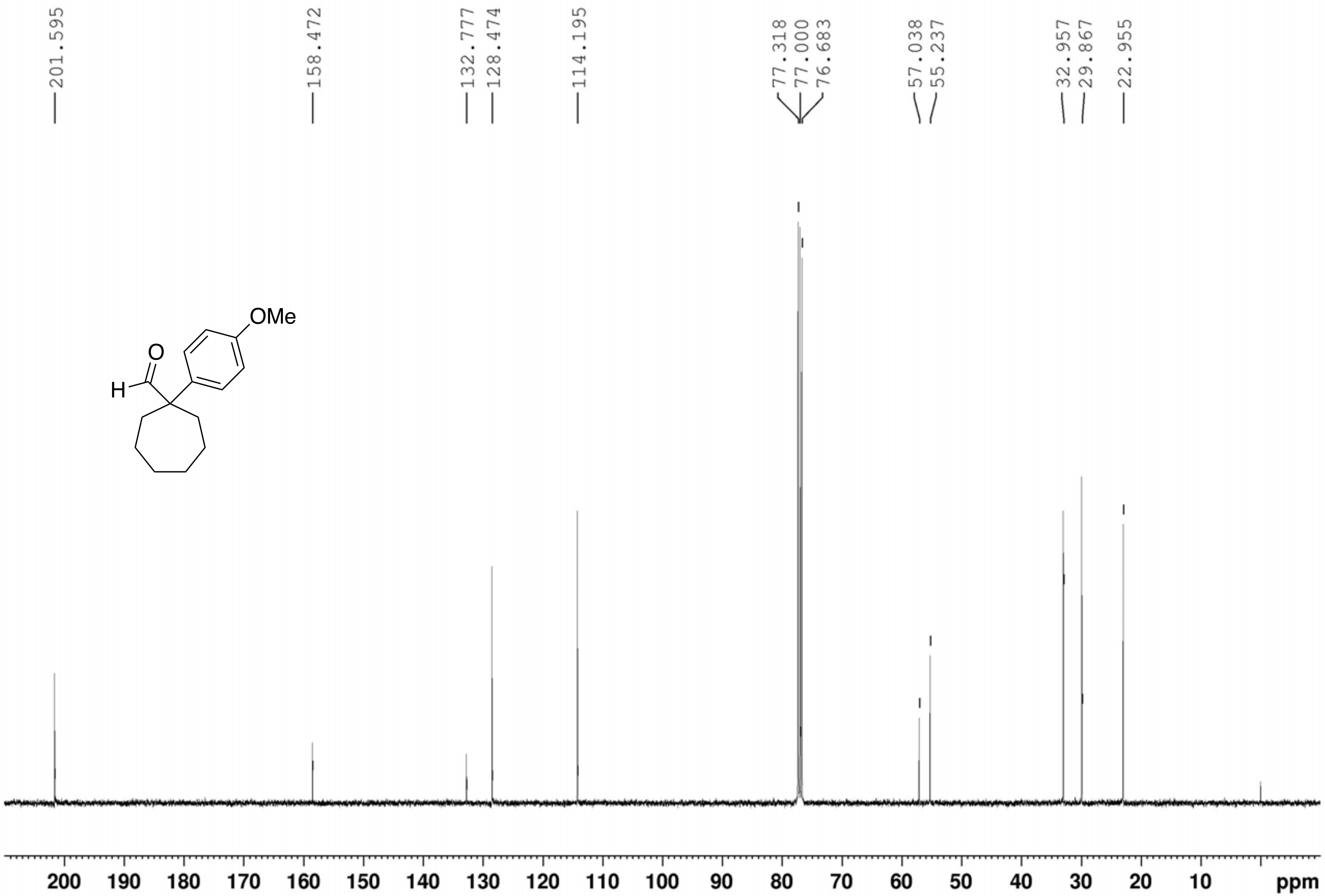

Supplementary Figure 109. ^{13}C NMR spectrum of **2o** (100.6 MHz, $CDCl_3$)

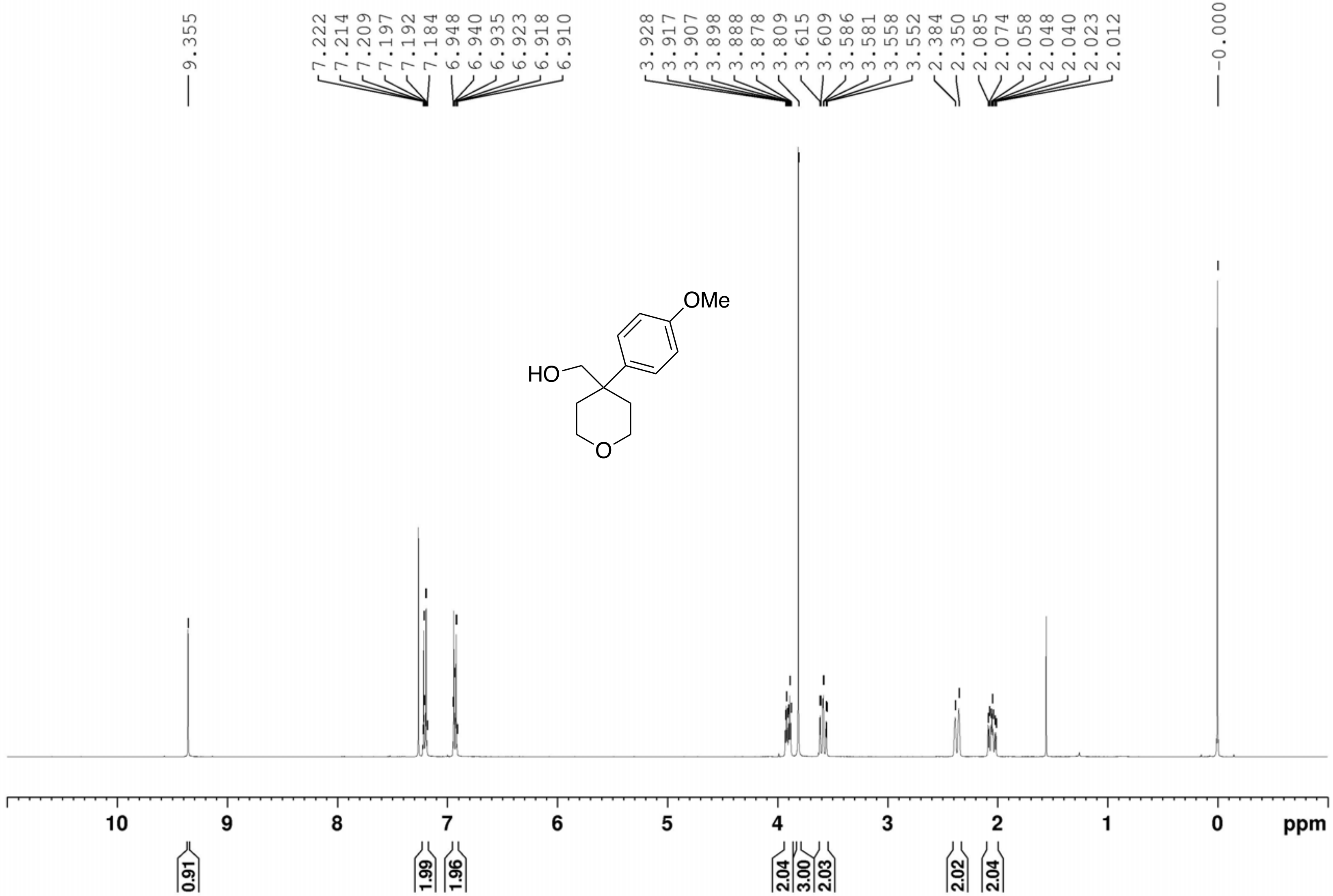

Supplementary Figure 110. ^{1}H NMR spectrum of **2p** (400 MHz, $CDCl_3$)

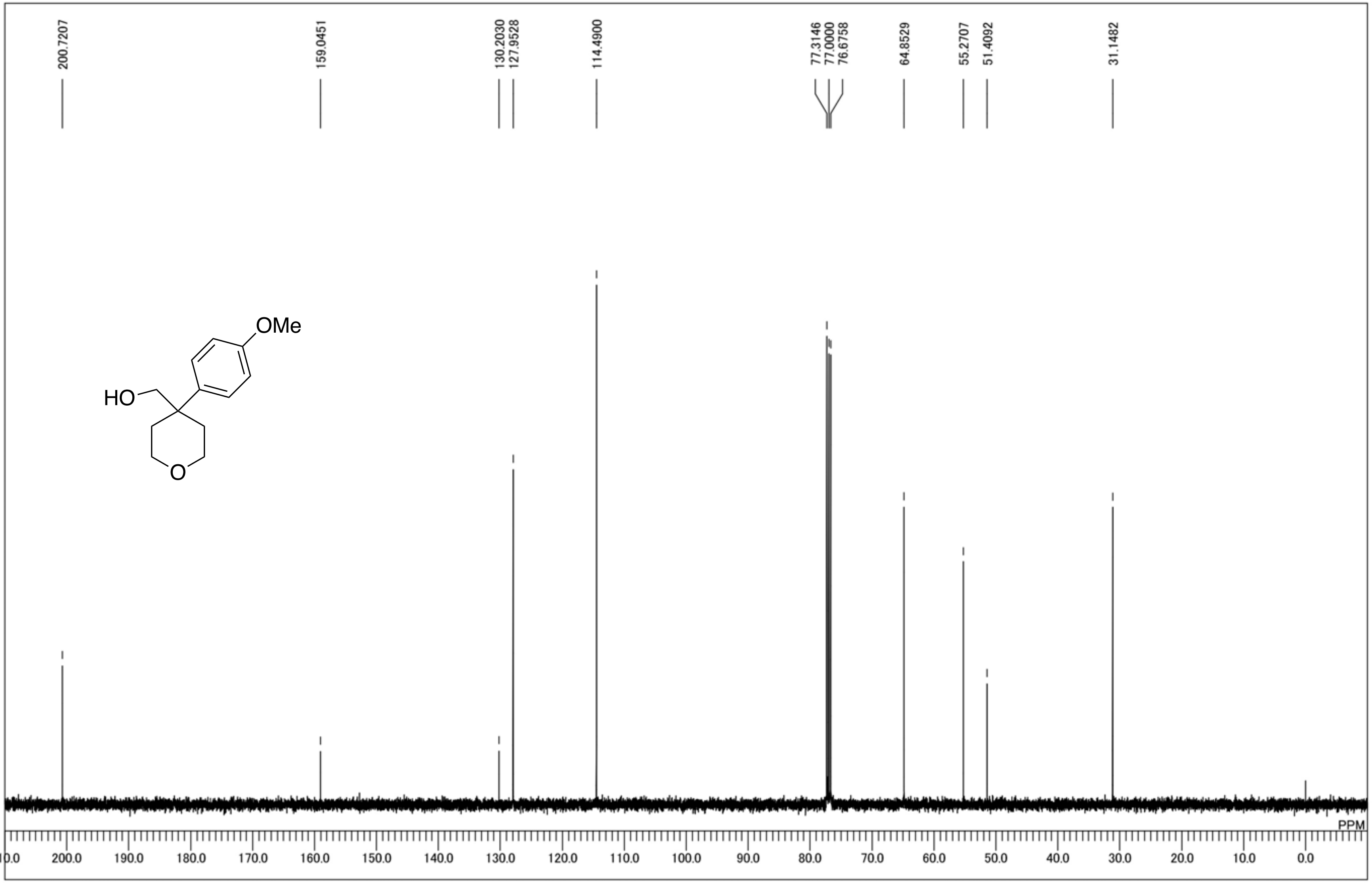

Supplementary Figure 111. ^{13}C NMR spectrum of **2p** (100.6 MHz, $CDCl_3$)

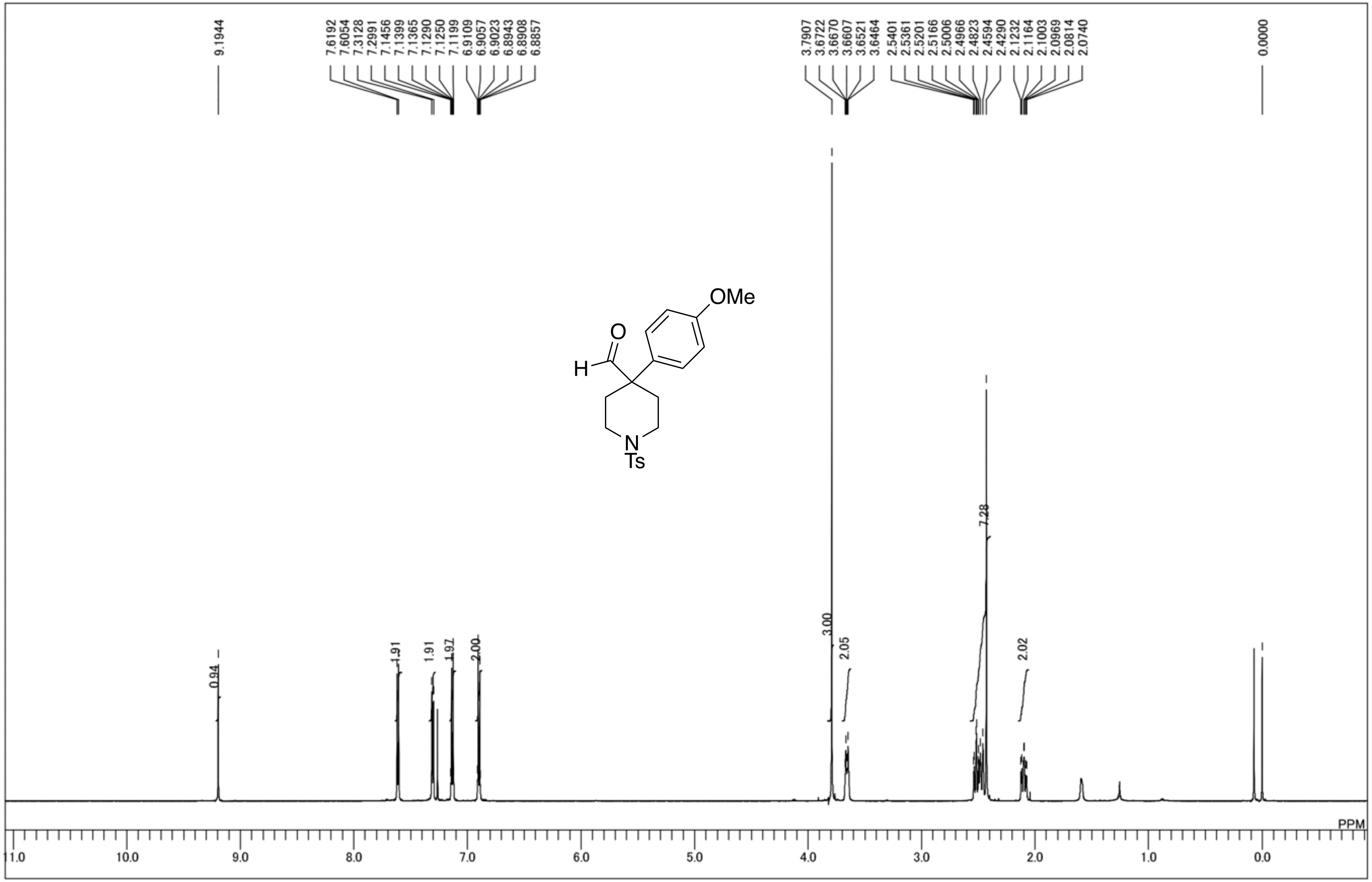

Supplementary Figure 112. ^{1}H NMR spectrum of **2q** (600 MHz, $CDCl_3$)

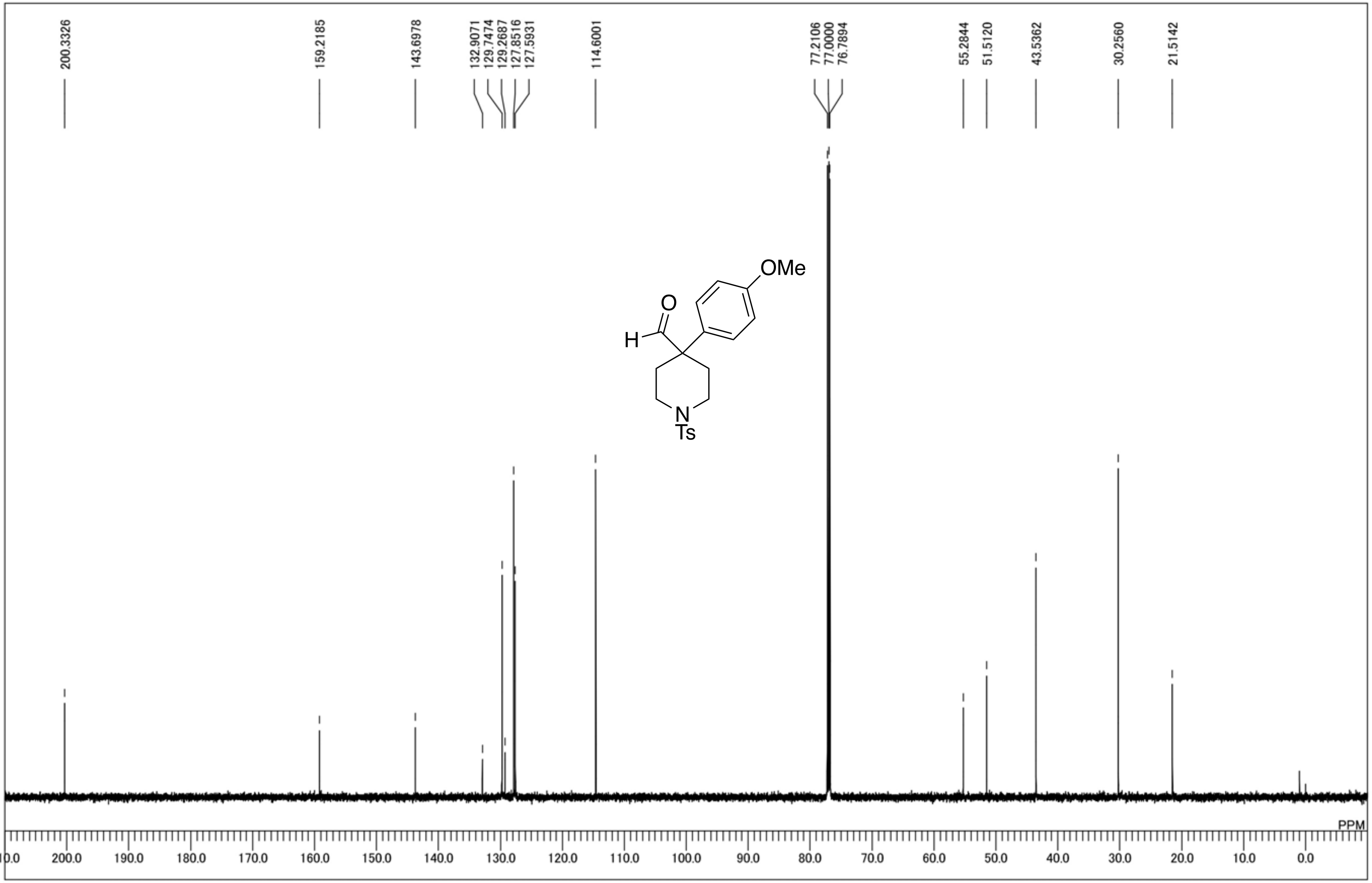

Supplementary Figure 113. ^{13}C NMR spectrum of **2q** (150.9 MHz, $CDCl_3$)

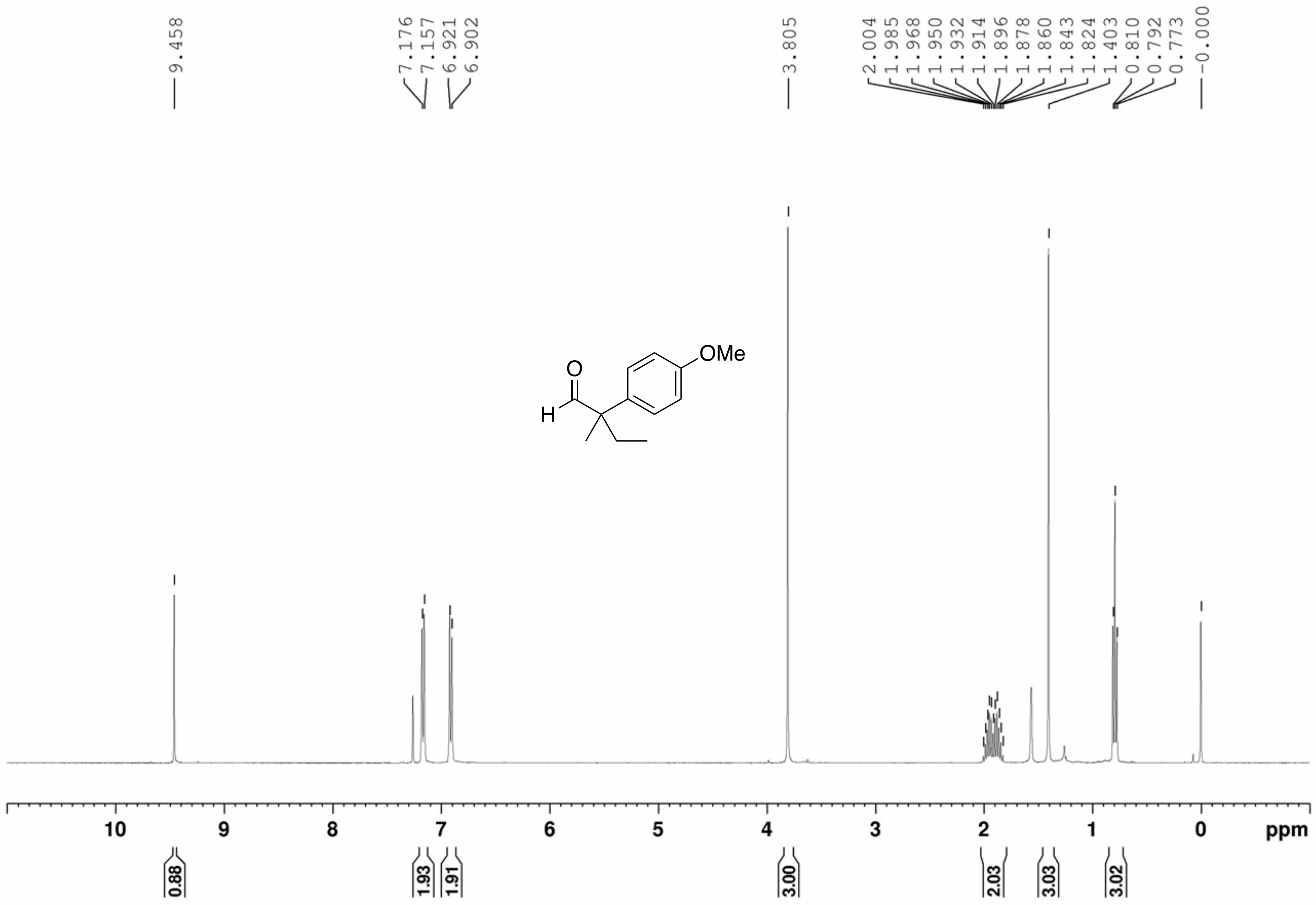

Supplementary Figure 114. ^{1}H NMR spectrum of **2r** (400 MHz, $CDCl_3$)

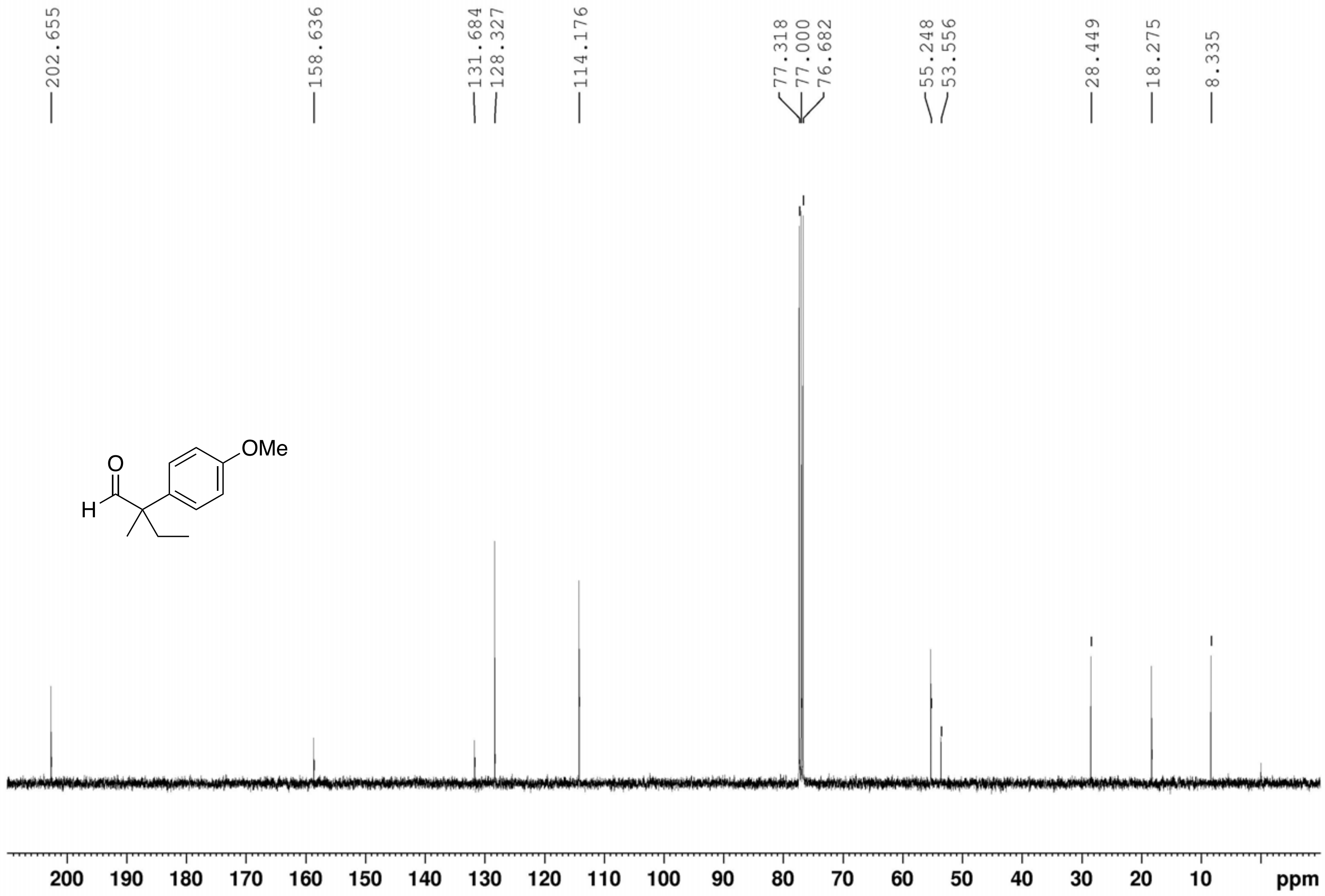

Supplementary Figure 115. ^{13}C NMR spectrum of **2r** (100.6 MHz, $CDCl_3$)

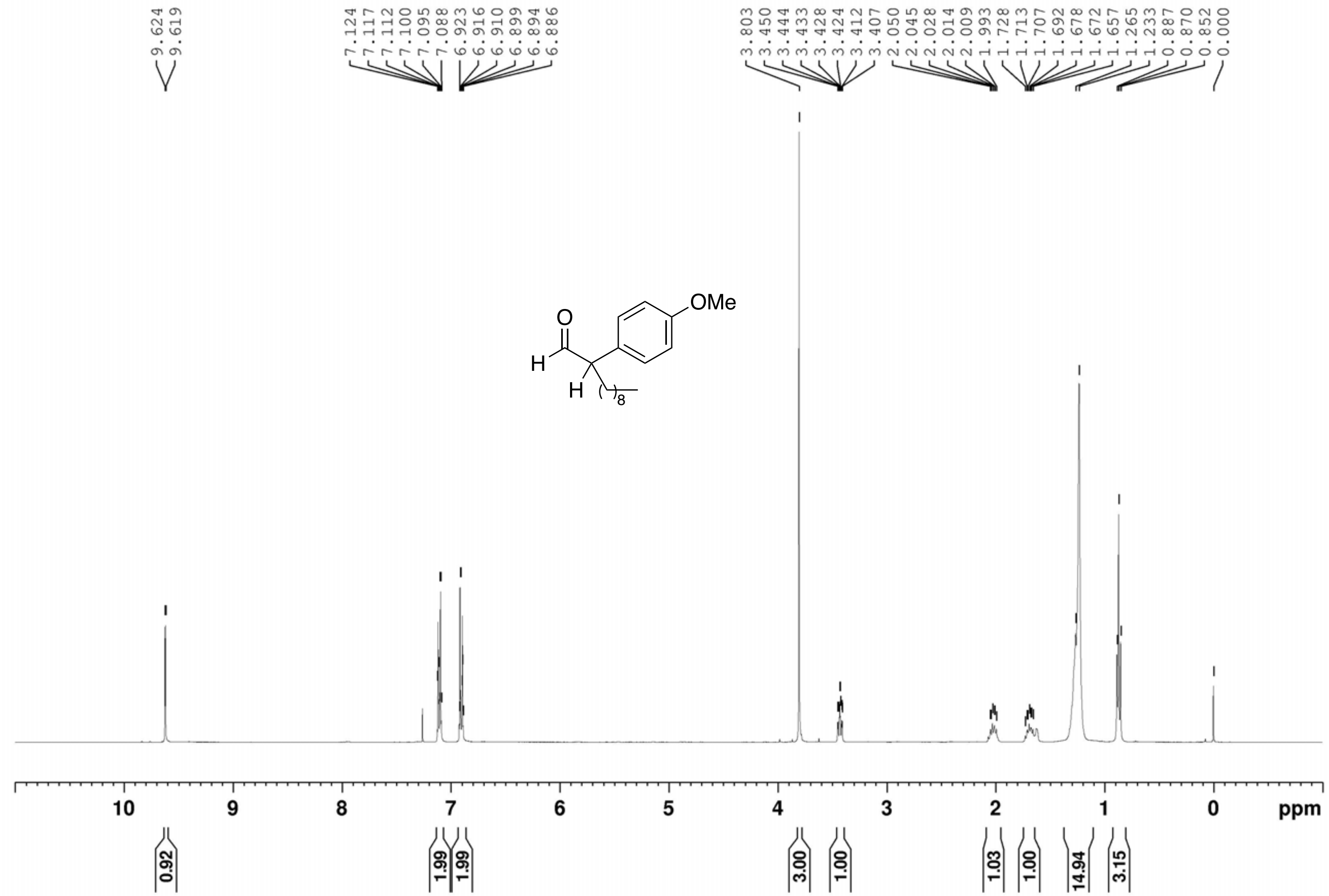

Supplementary Figure 116. ^{1}H NMR spectrum of **2s** (400 MHz, $CDCl_3$)

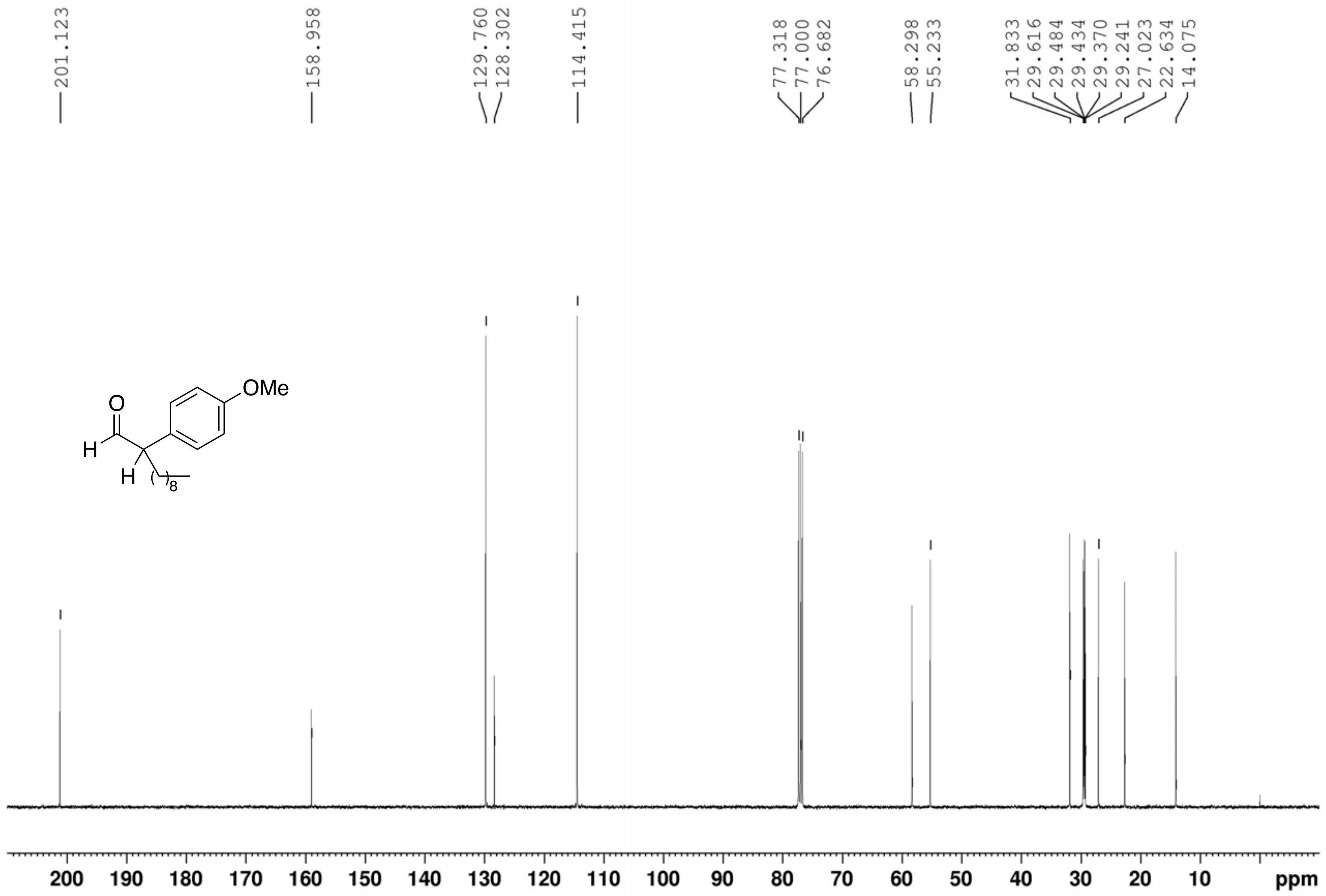

Supplementary Figure 117. ${}^{13}C$ NMR spectrum of **2s** (100.6 MHz, $CDCl_3$)

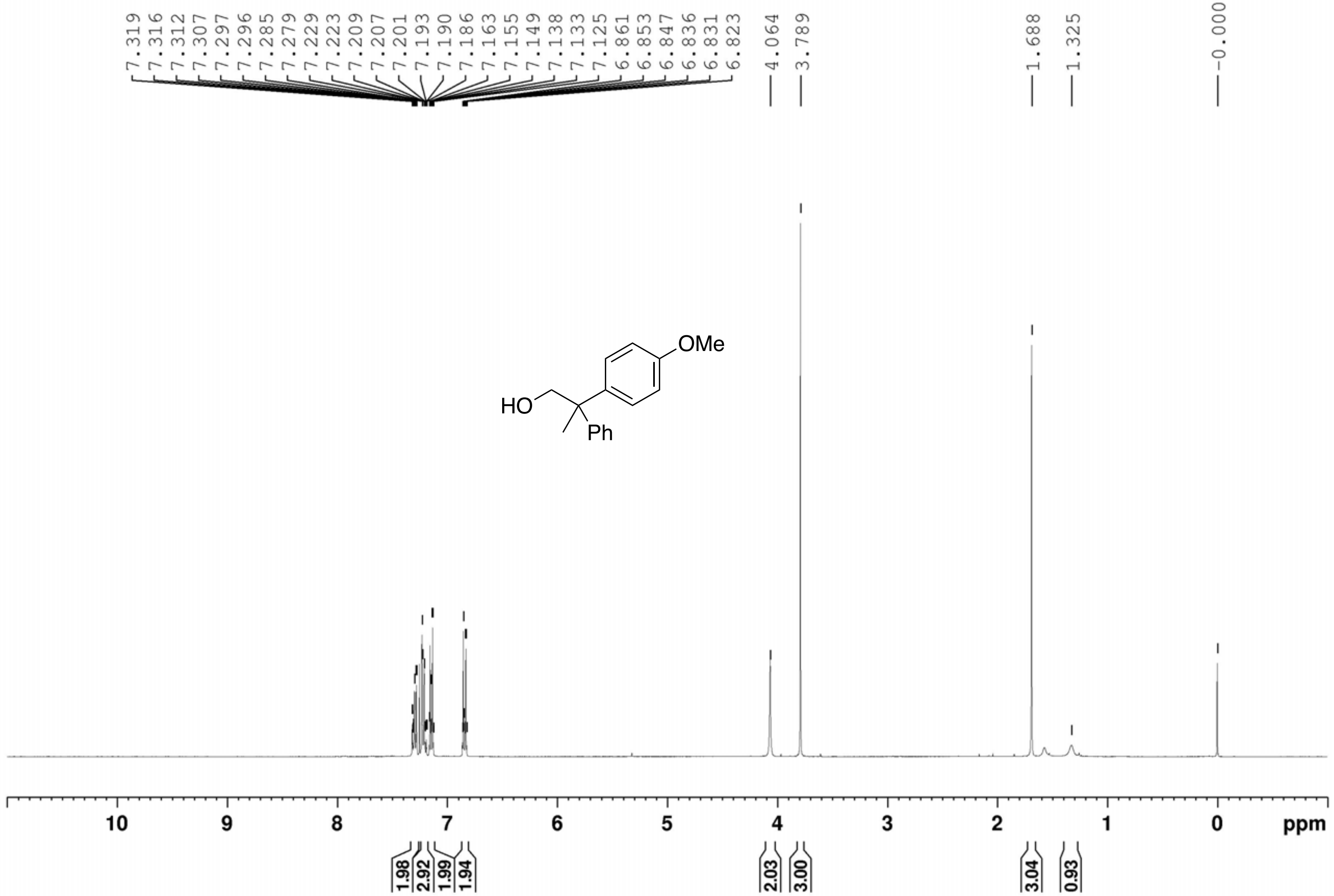

Supplementary Figure 118. ^{1}H NMR spectrum of **2t** (400 MHz, $CDCl_3$)

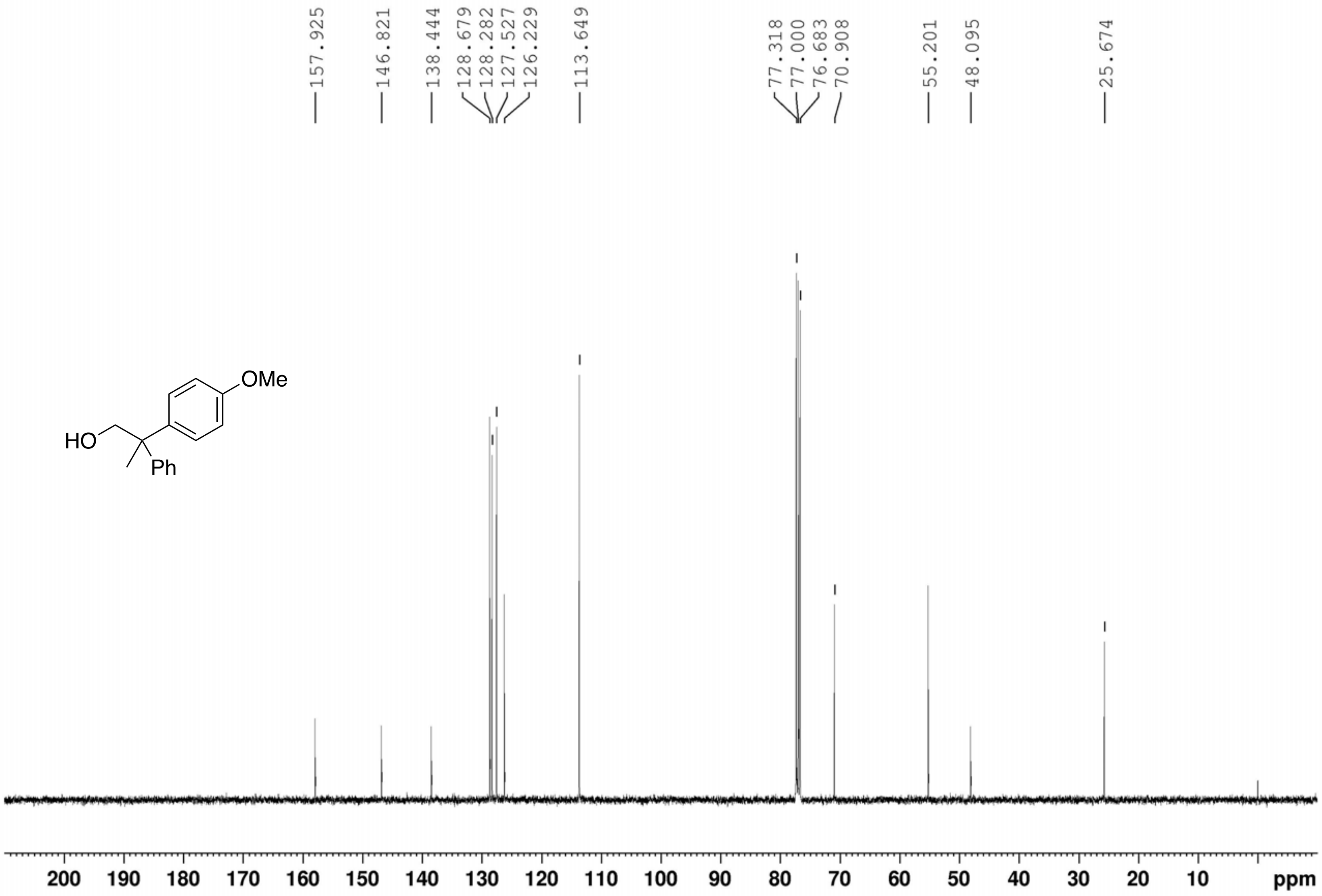

Supplementary Figure 119. ^{13}C NMR spectrum of **2t** (100.6 MHz, $CDCl_3$)

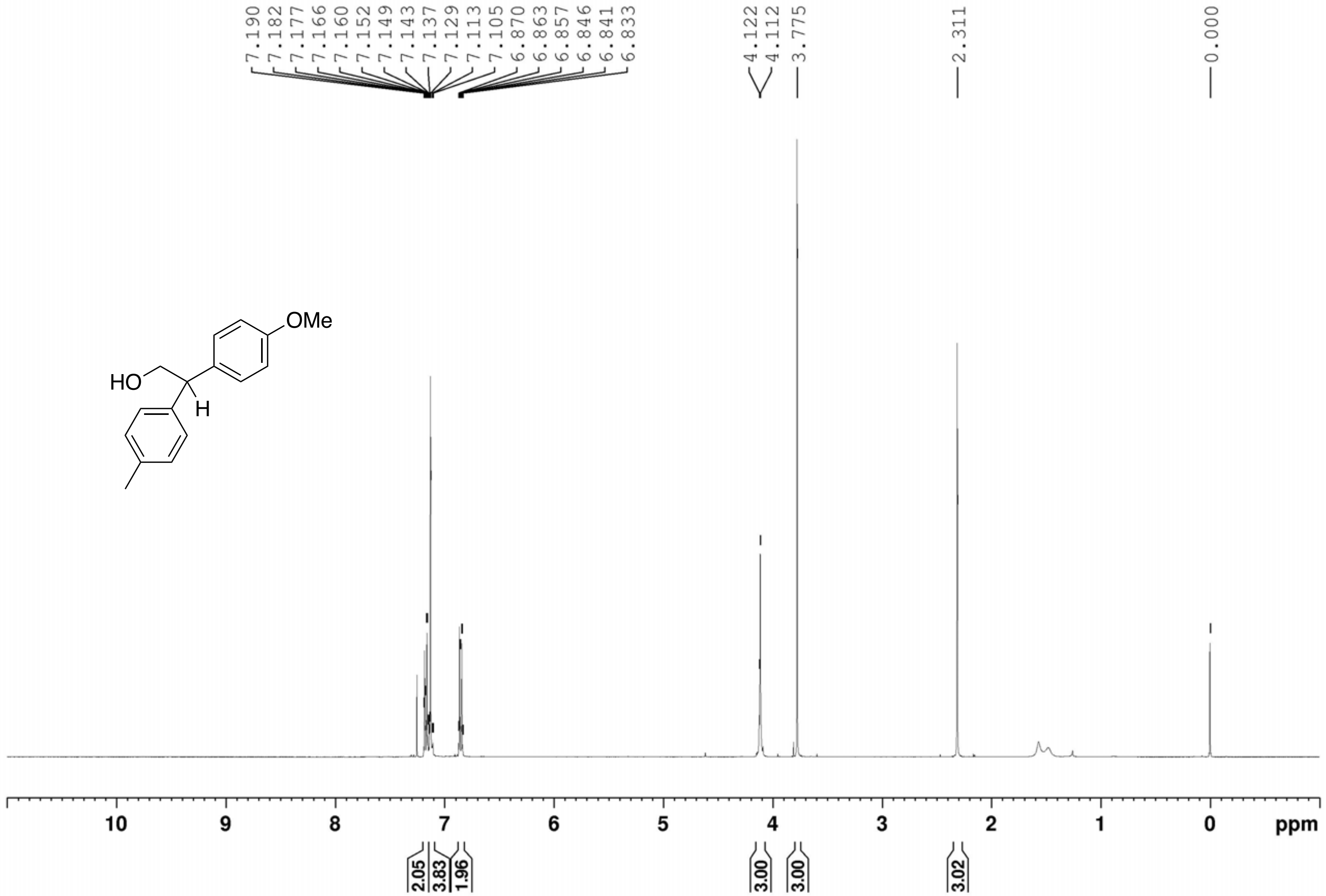

Supplementary Figure 120. ^{1}H NMR spectrum of **2u** (400 MHz, $CDCl_3$)

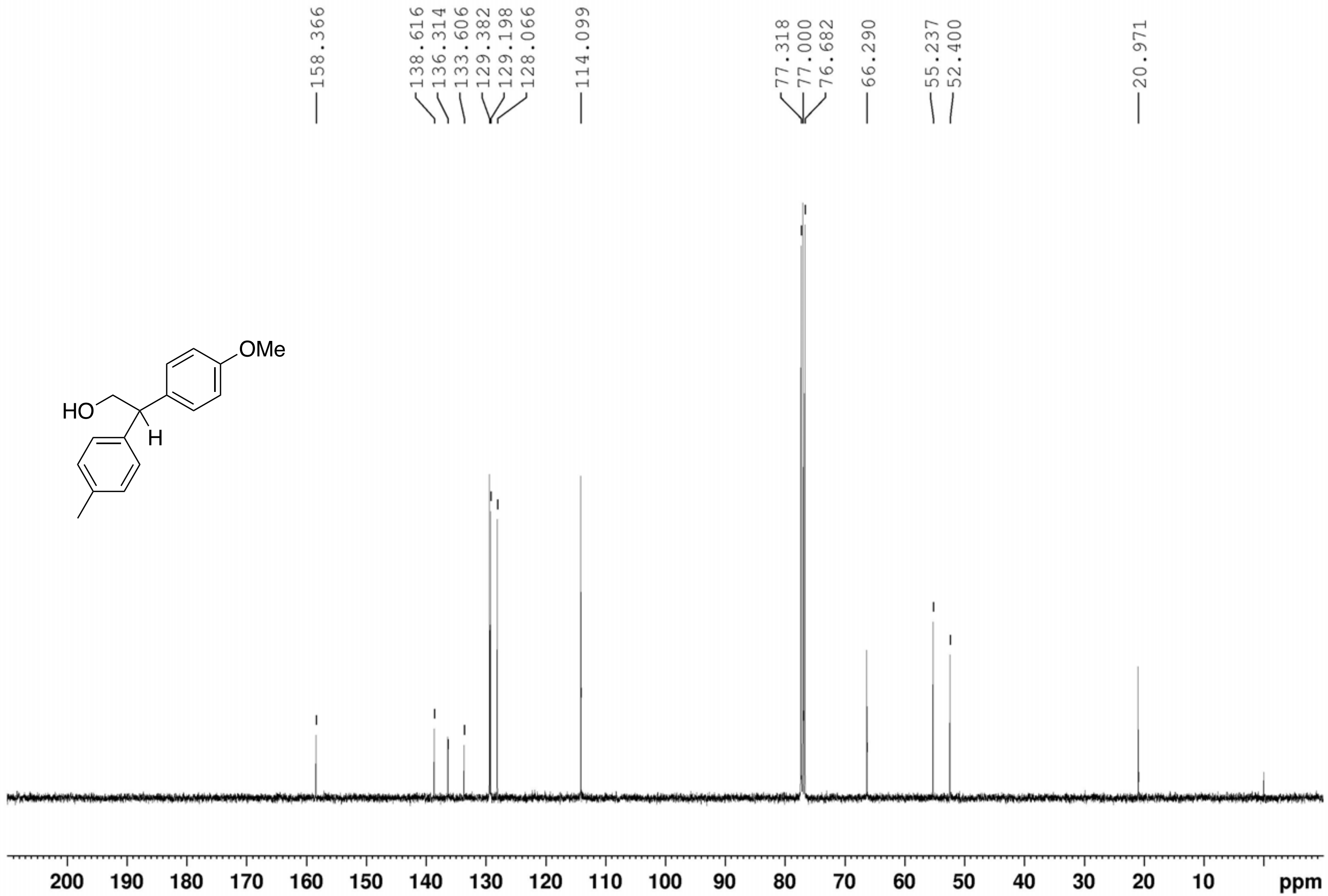

Supplementary Figure 121. ^{13}C NMR spectrum of **2u** (100.6 MHz, $CDCl_3$)

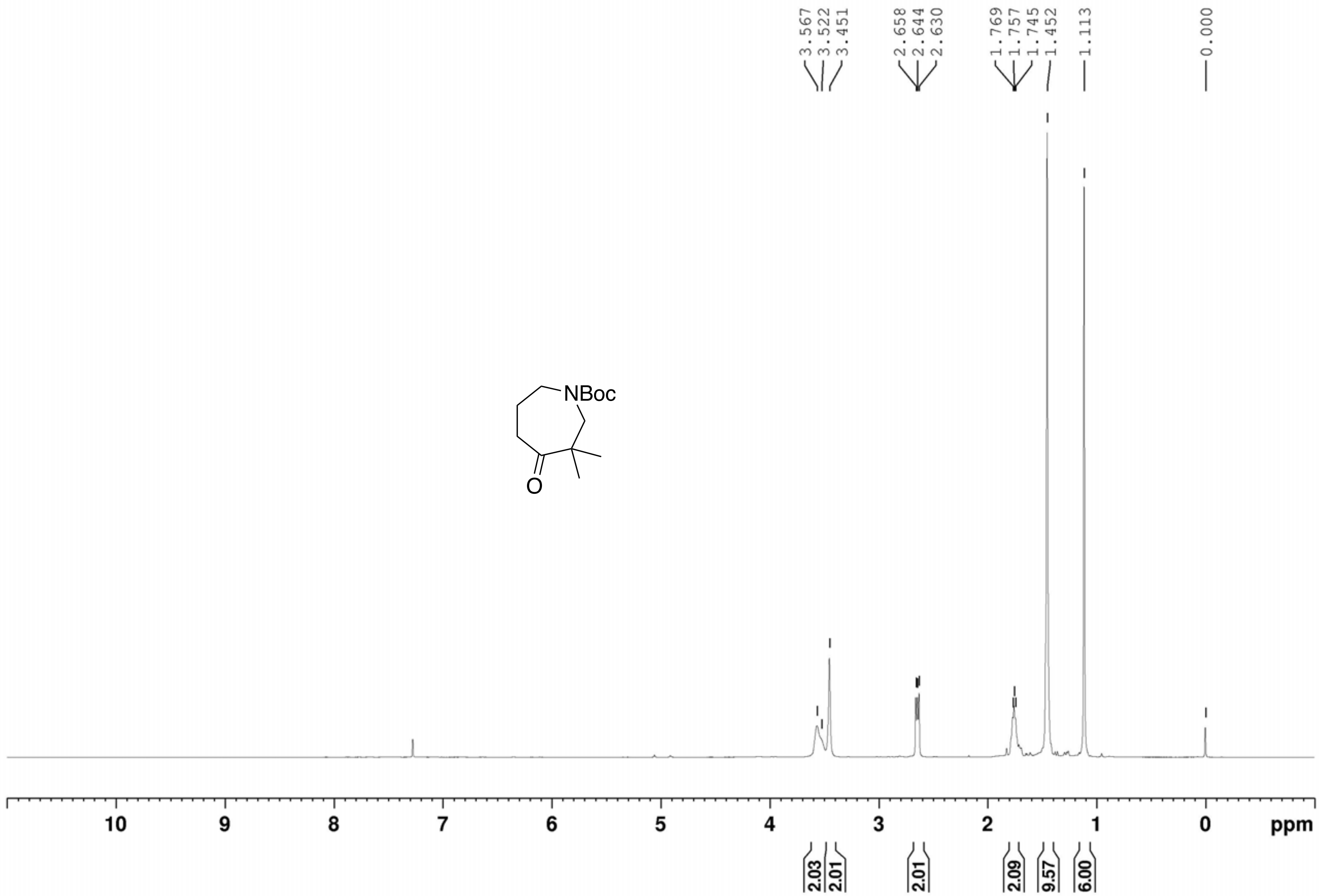

Supplementary Figure 122. ^{1}H NMR spectrum of **2v** (400 MHz, $CDCl_3$)

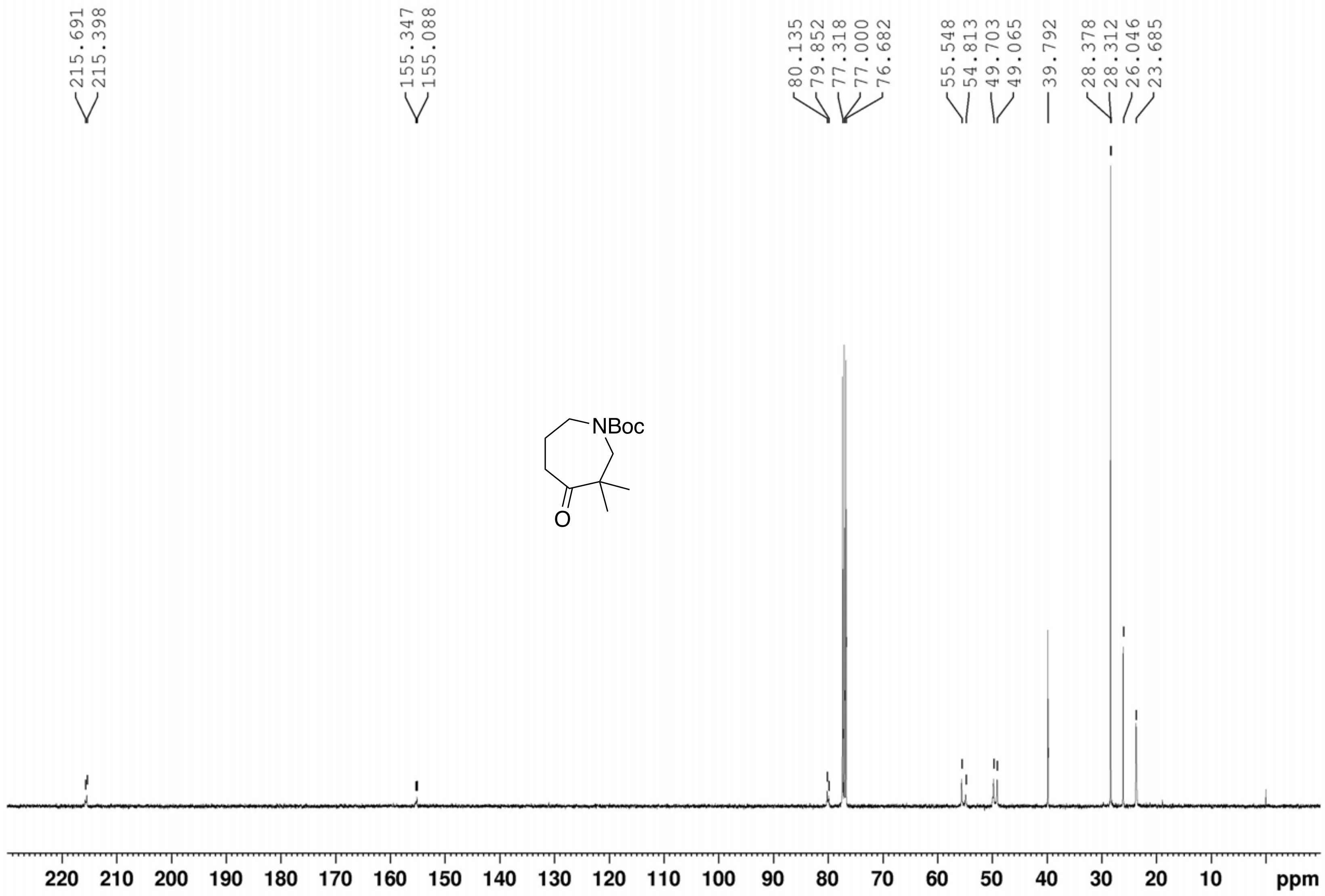

Supplementary Figure 123. ^{13}C NMR spectrum of **2v** (100.6 MHz, $CDCl_3$)

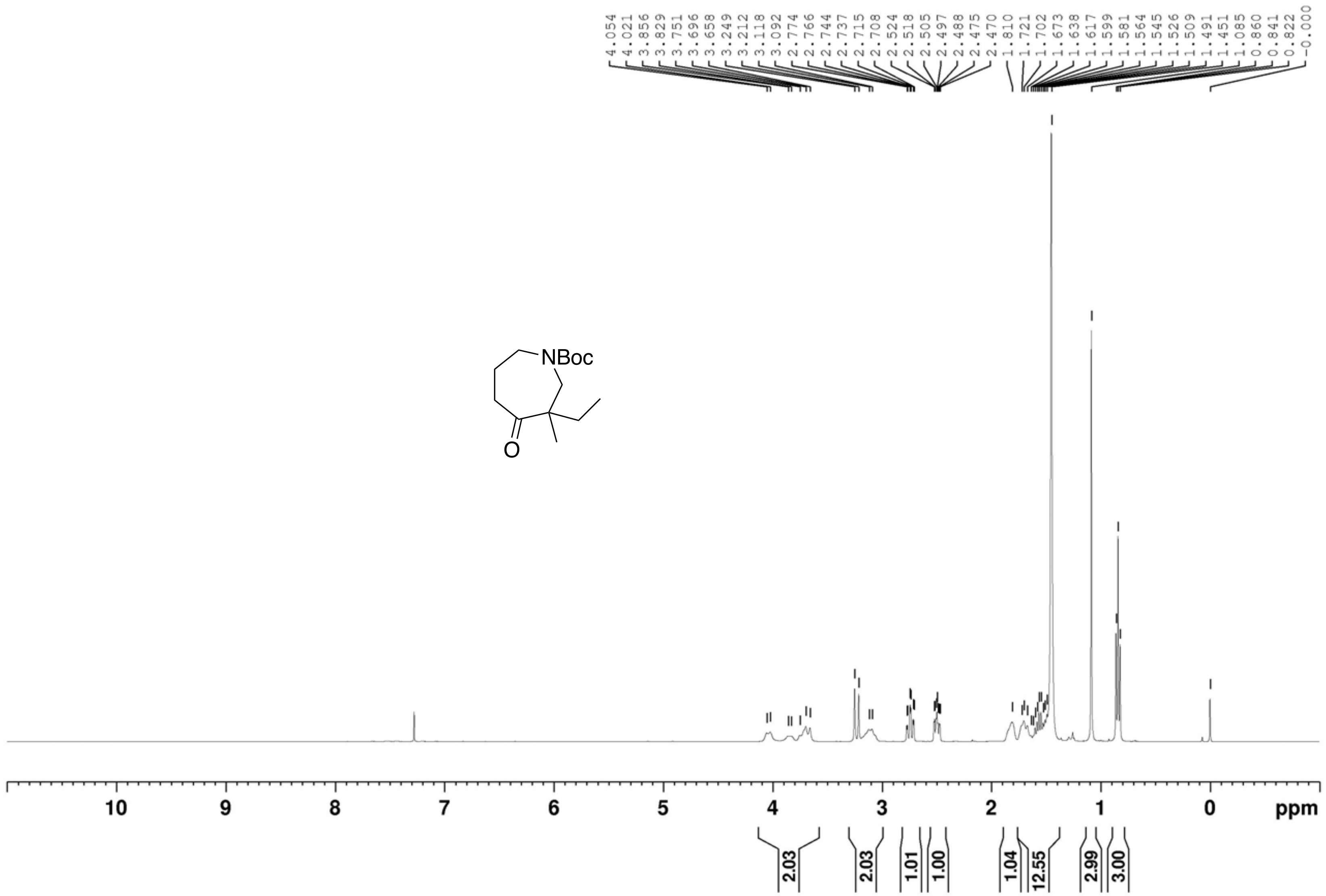

Supplementary Figure 124. ^{1}H NMR spectrum of **2w** (400 MHz, $CDCl_3$)

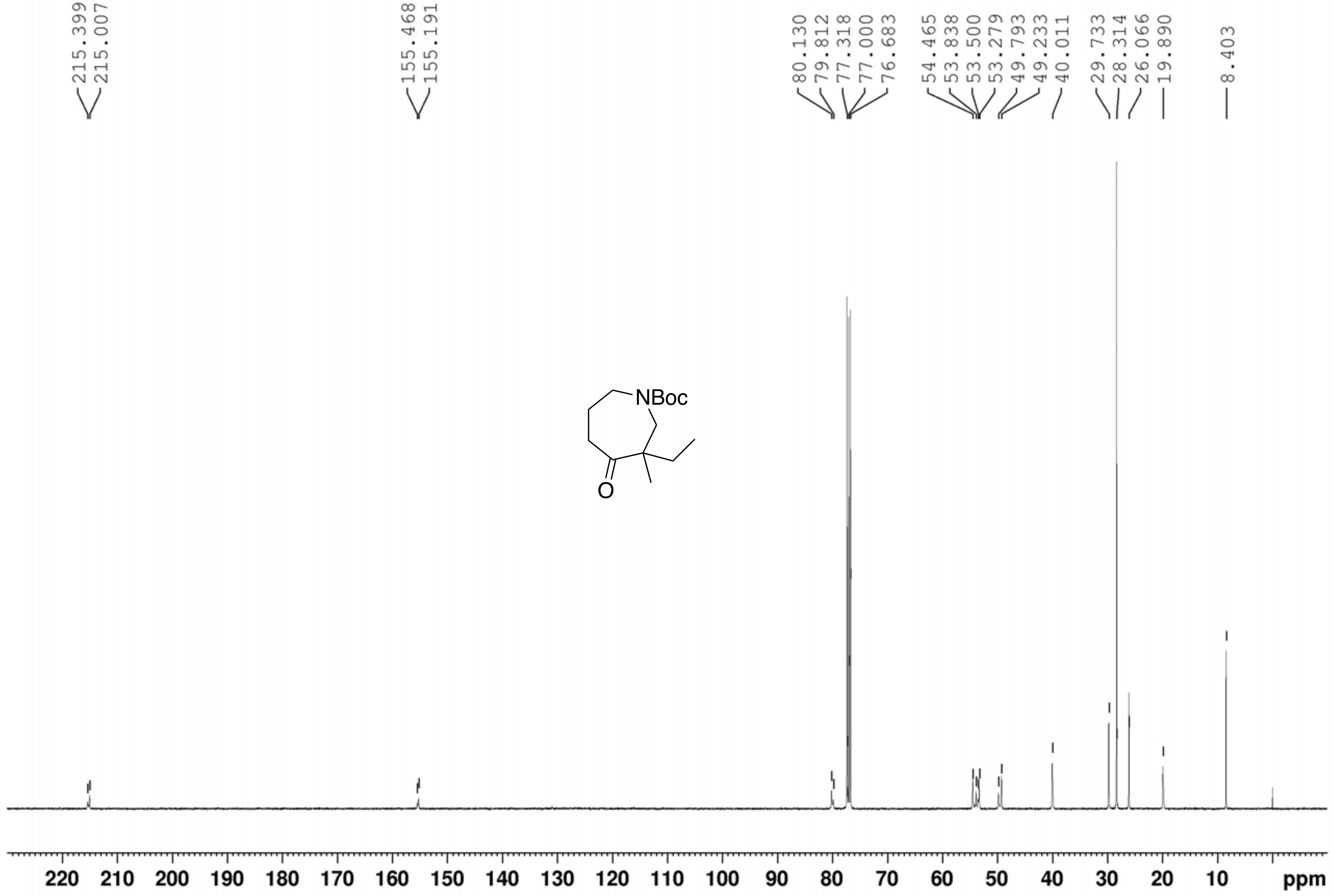

Supplementary Figure 125. ^{13}C NMR spectrum of **2w** (100.6 MHz, $CDCl_3$)

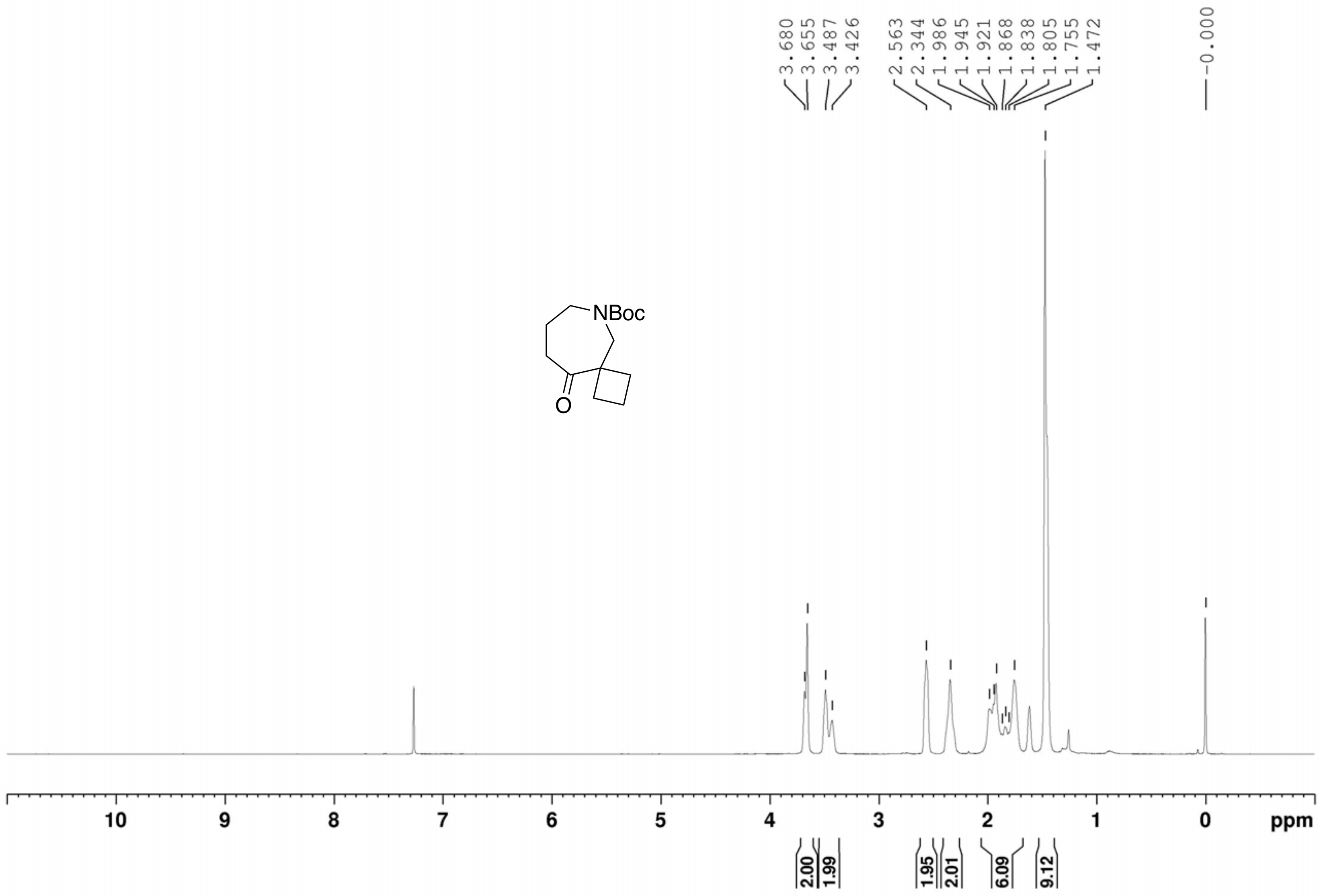

Supplementary Figure 126. ^{1}H NMR spectrum of **2x** (400 MHz, $CDCl_3$)

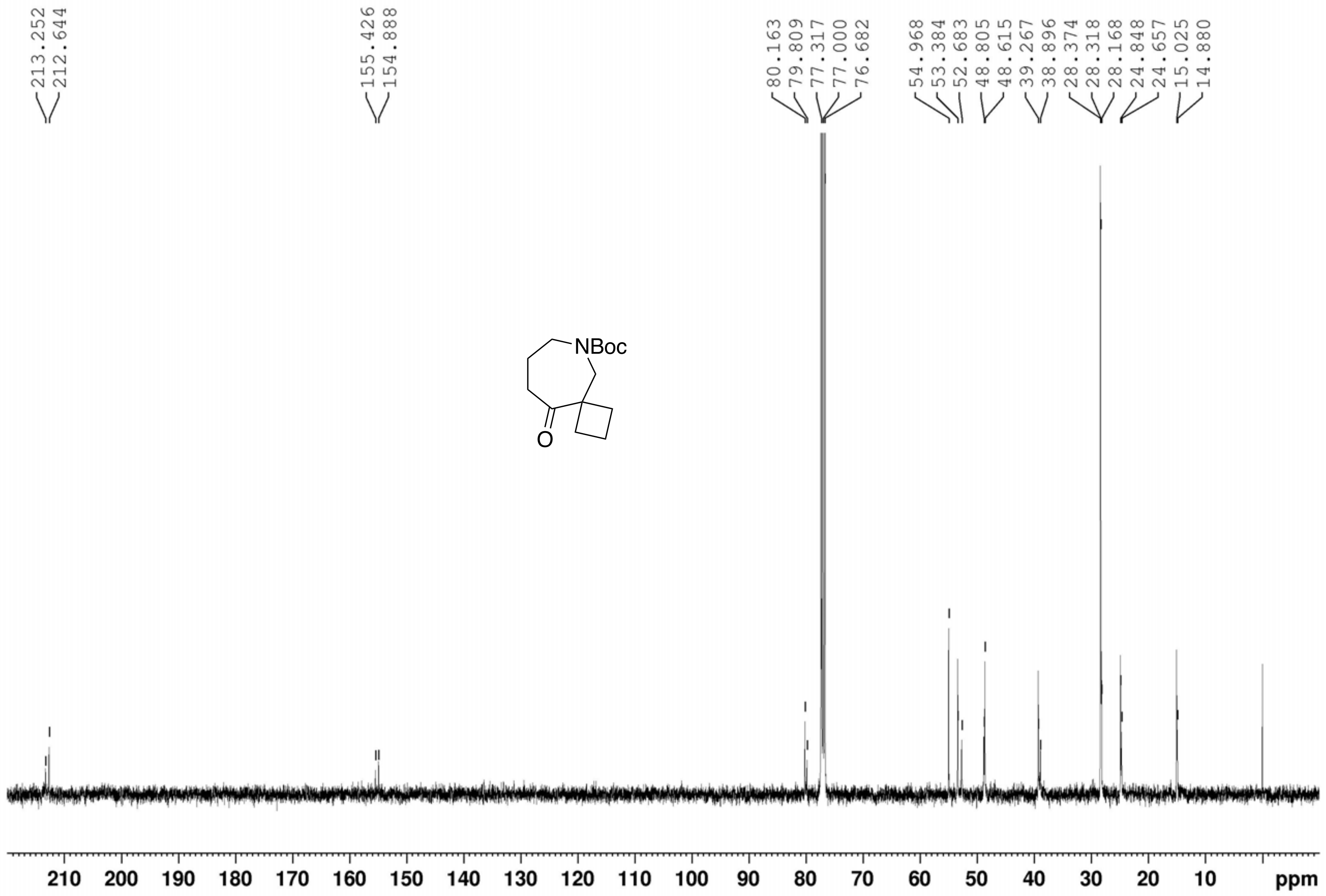

Supplementary Figure 127. ^{13}C NMR spectrum of **2x** (100.6 MHz, $CDCl_3$)

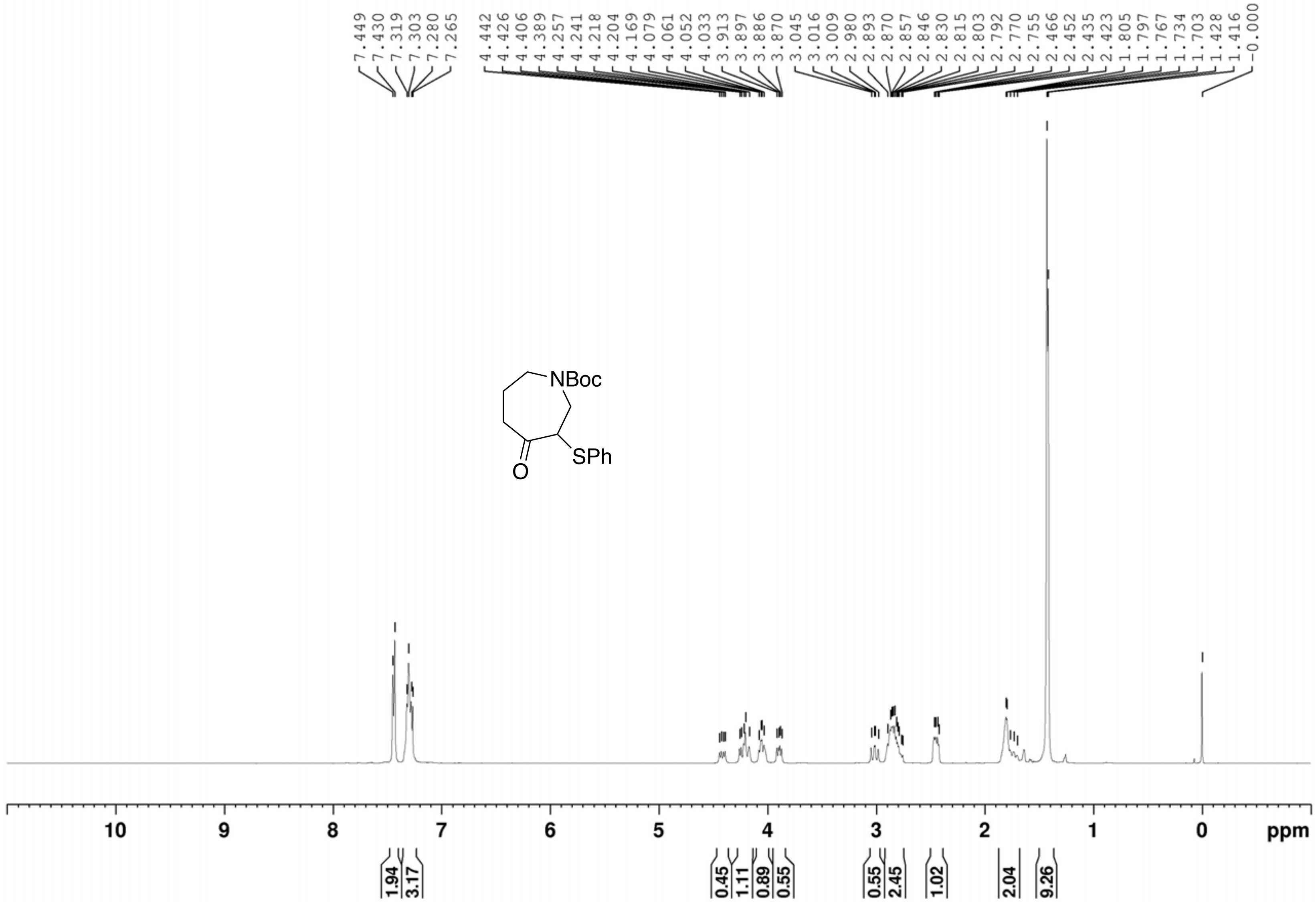

Supplementary Figure 128. ^{1}H NMR spectrum of **2y** (400 MHz, $CDCl_3$)

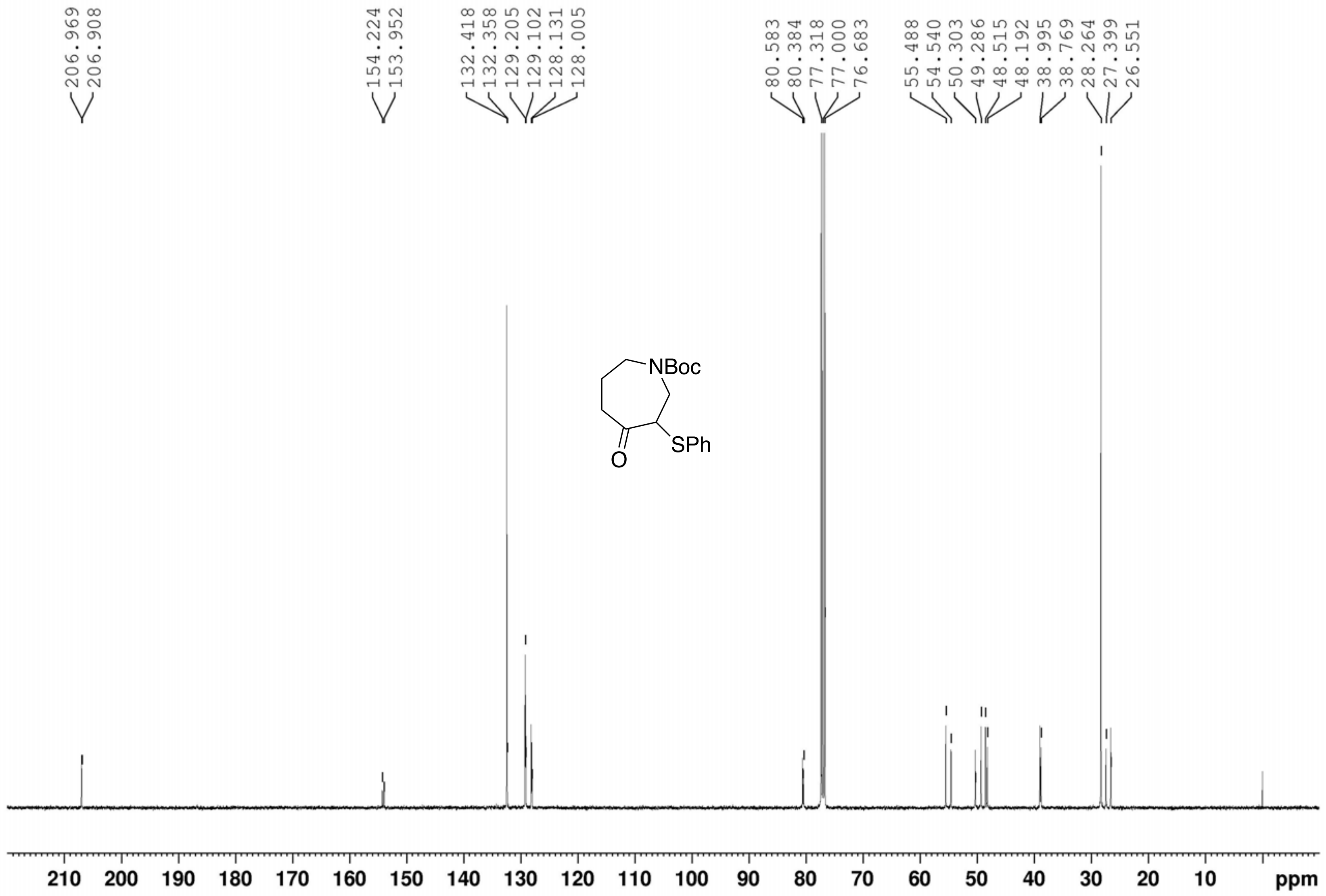

Supplementary Figure 129. ^{13}C NMR spectrum of **2y** (100.6 MHz, $CDCl_3$)

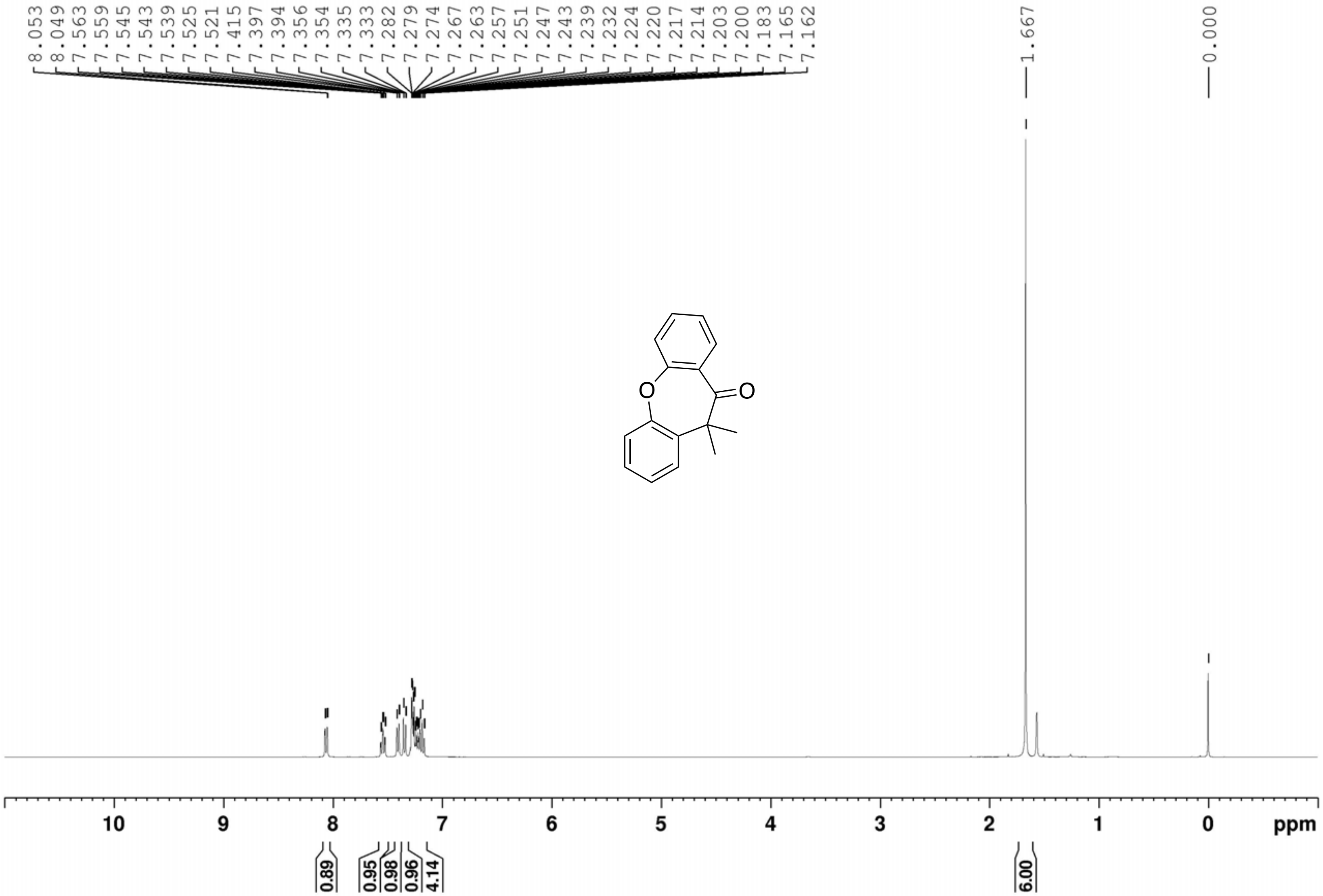

Supplementary Figure 130. ^{1}H NMR spectrum of **2z** (400 MHz, $CDCl_3$)

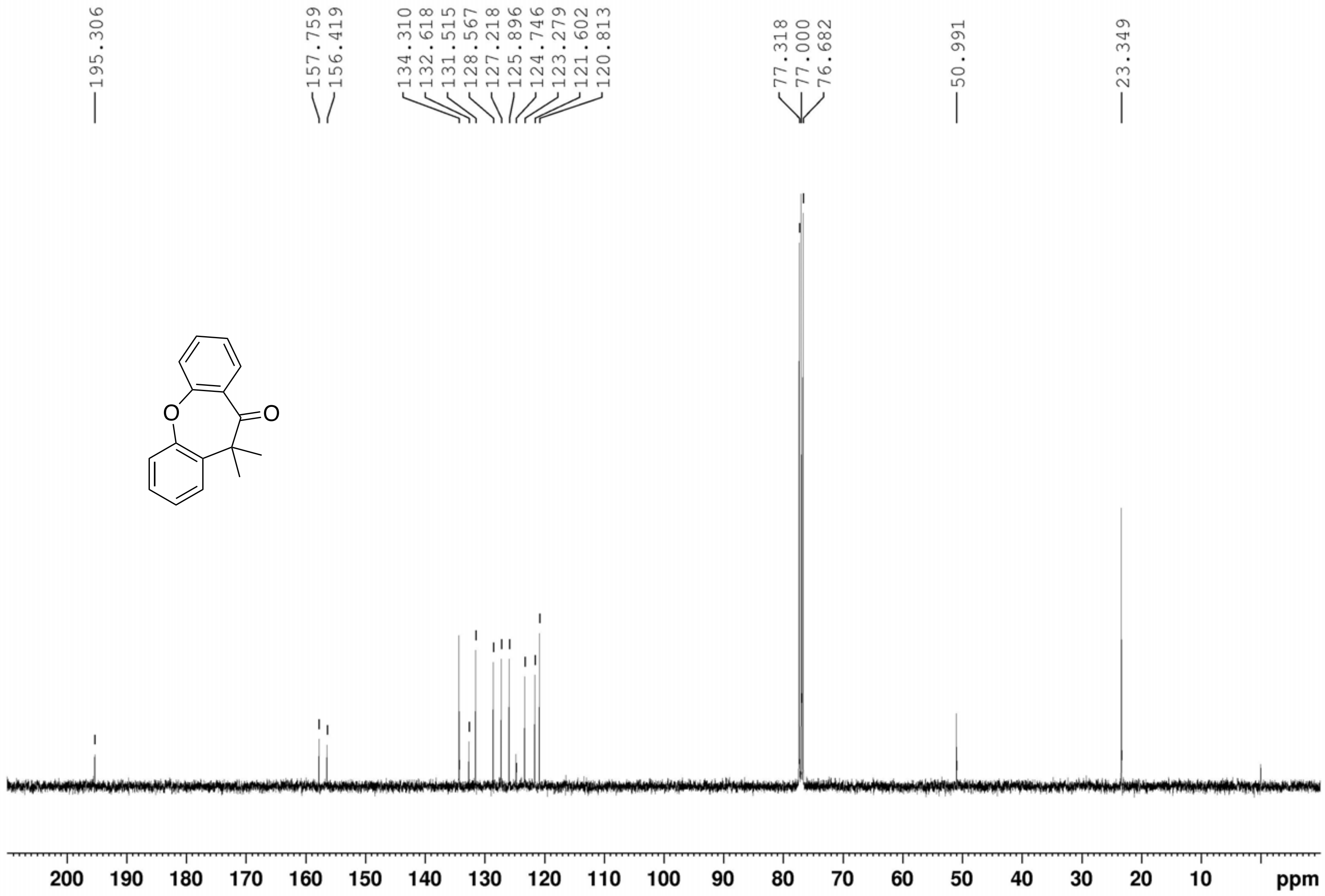

Supplementary Figure 131. ^{13}C NMR spectrum of **2z** (100.6 MHz, $CDCl_3$)

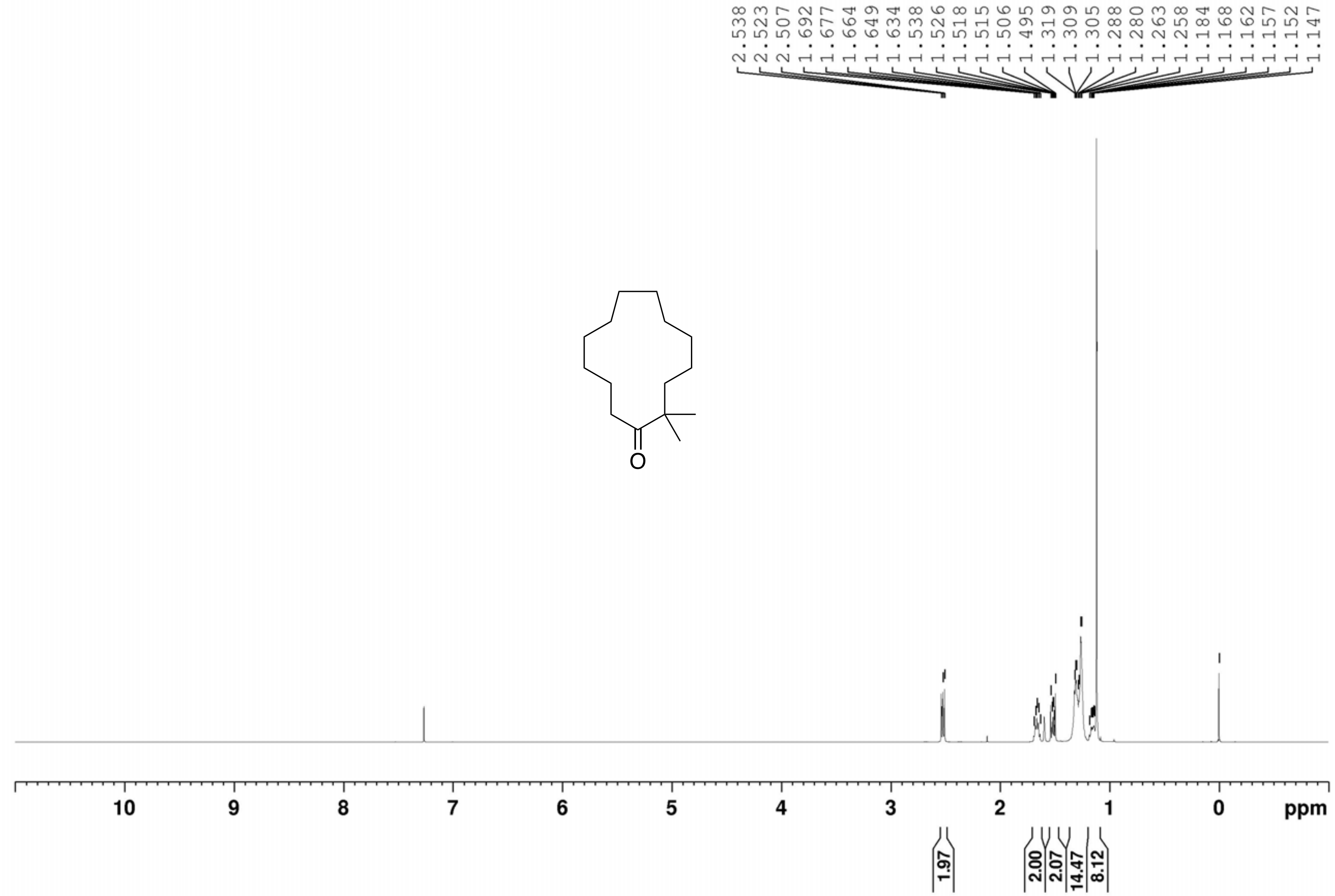

Supplementary Figure 132. ^{1}H NMR spectrum of **2A** (400 MHz, $CDCl_3$)

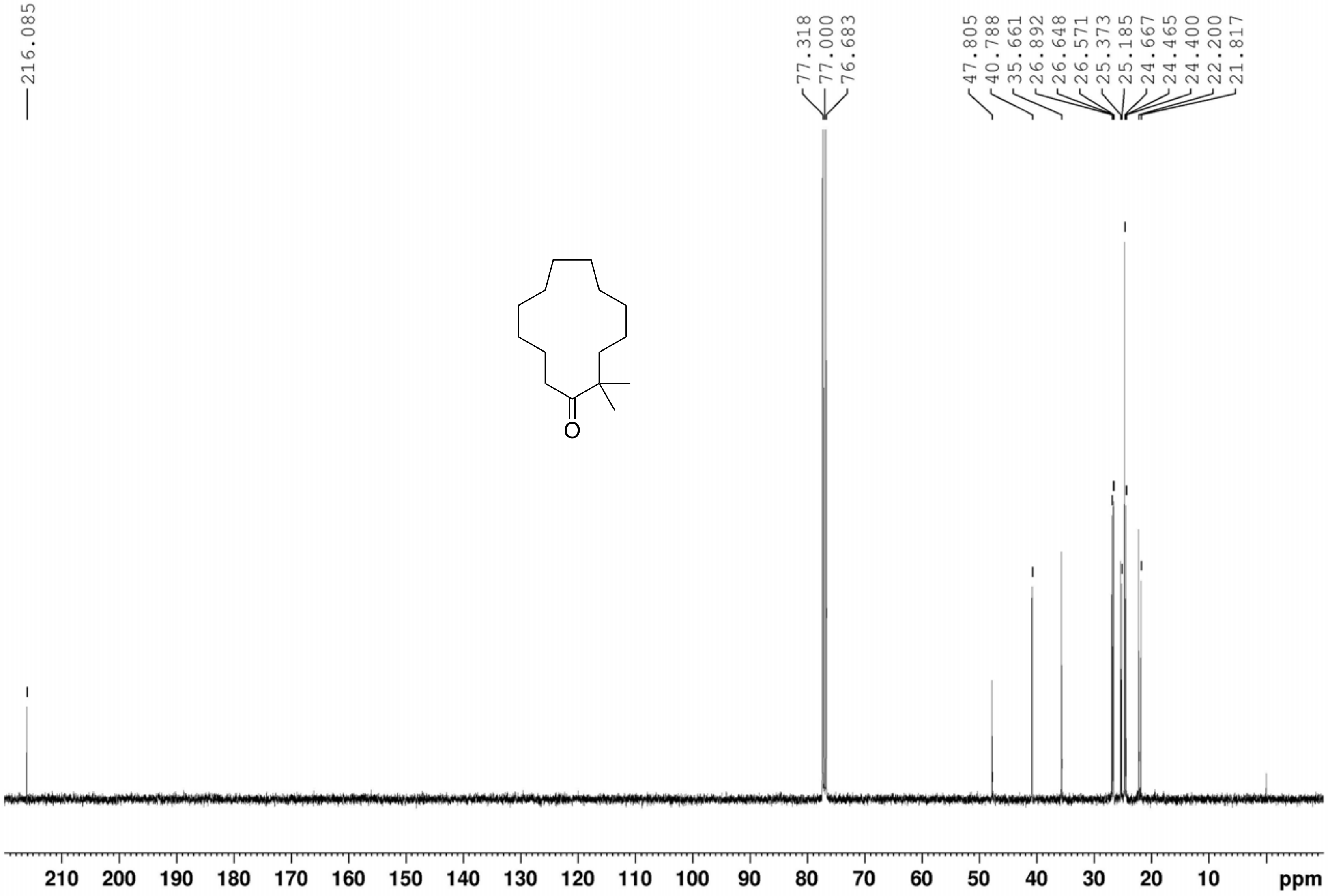

Supplementary Figure 133. ^{13}C NMR spectrum of **2A** (100.6 MHz, $CDCl_3$)

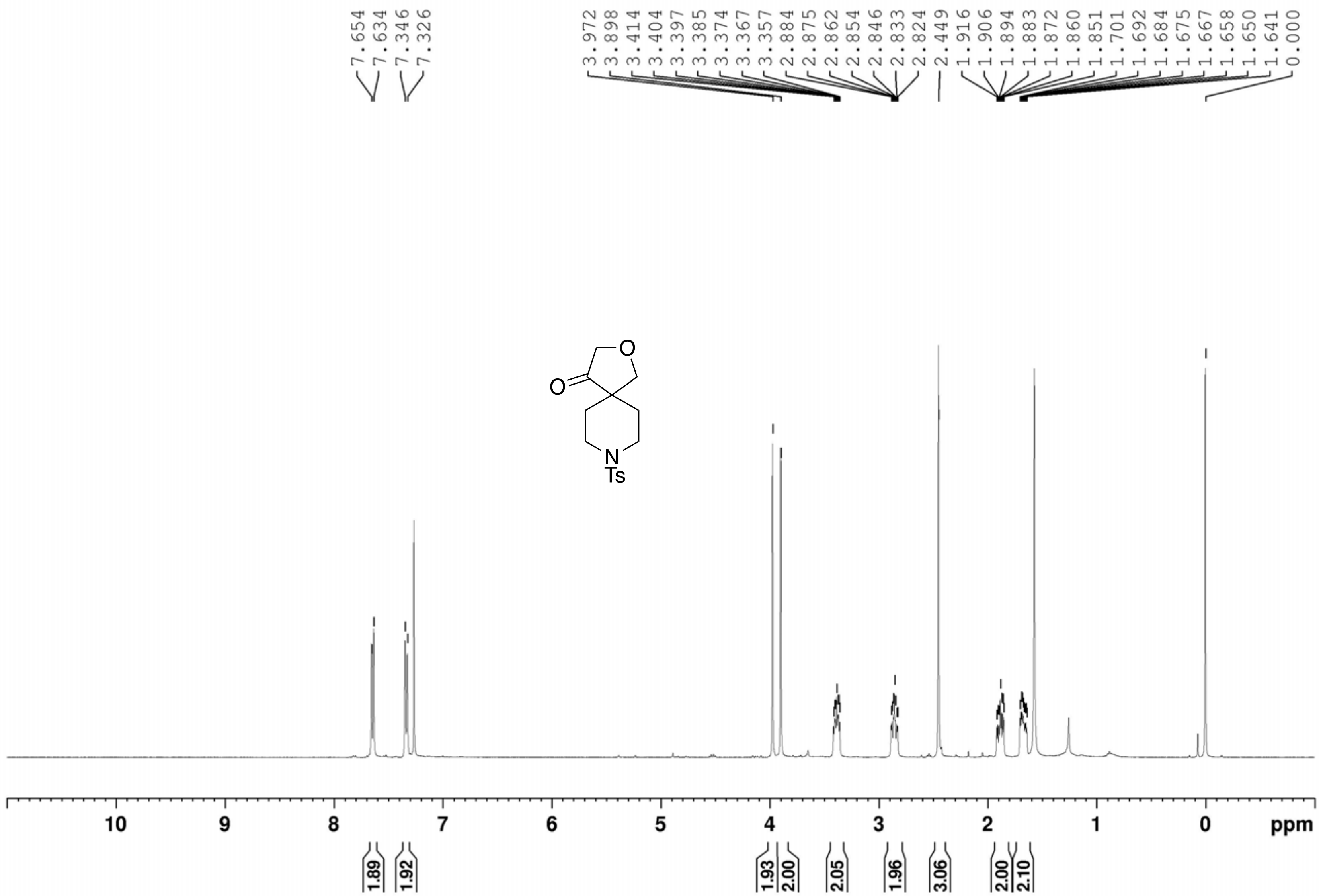

Supplementary Figure 134. ^{1}H NMR spectrum of **2B** (400 MHz, $CDCl_3$)

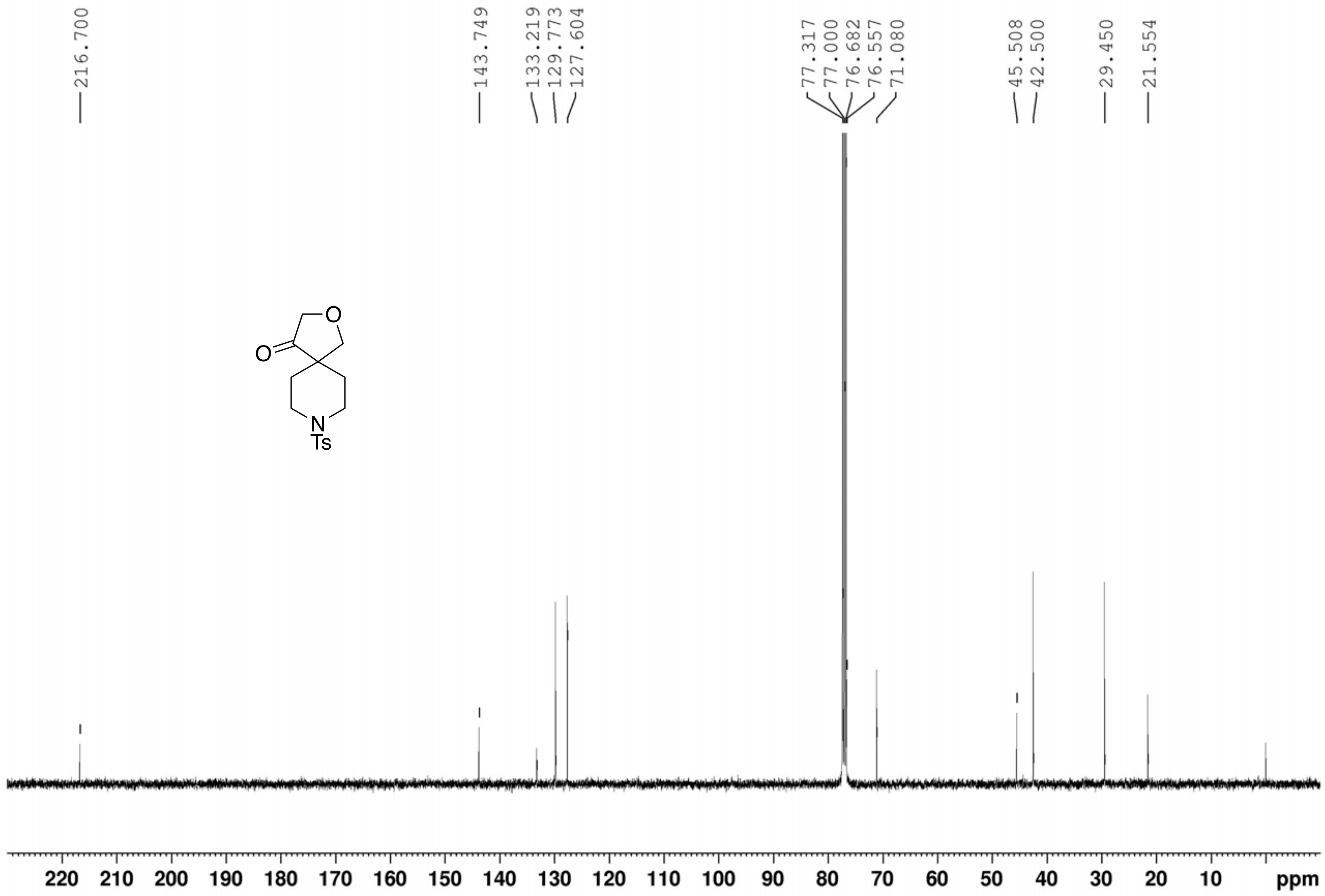

Supplementary Figure 135. ^{13}C NMR spectrum of **2B** (100.6 MHz, $CDCl_3$)

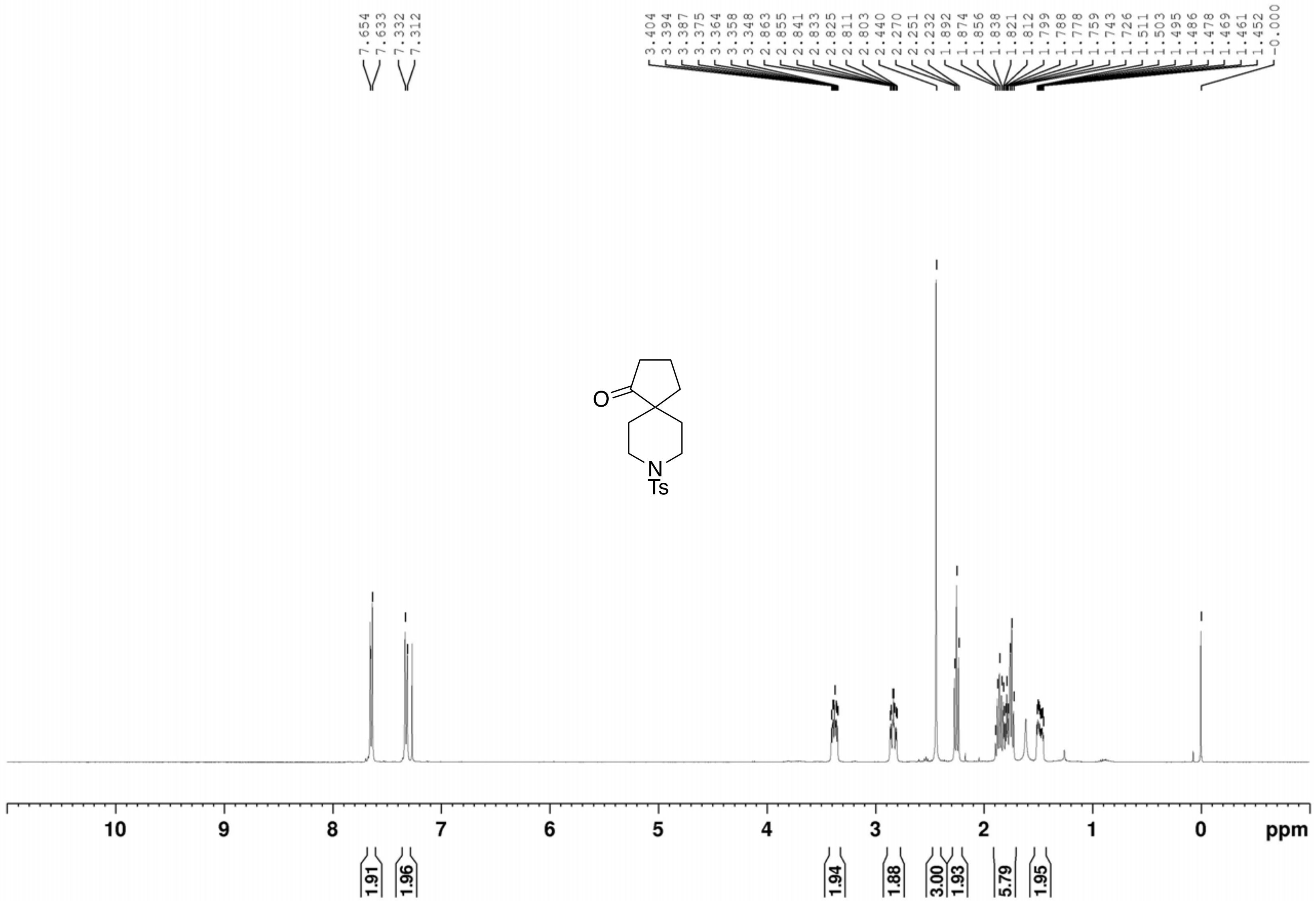

Supplementary Figure 136. ^{1}H NMR spectrum of **2C** (400 MHz, $CDCl_3$)

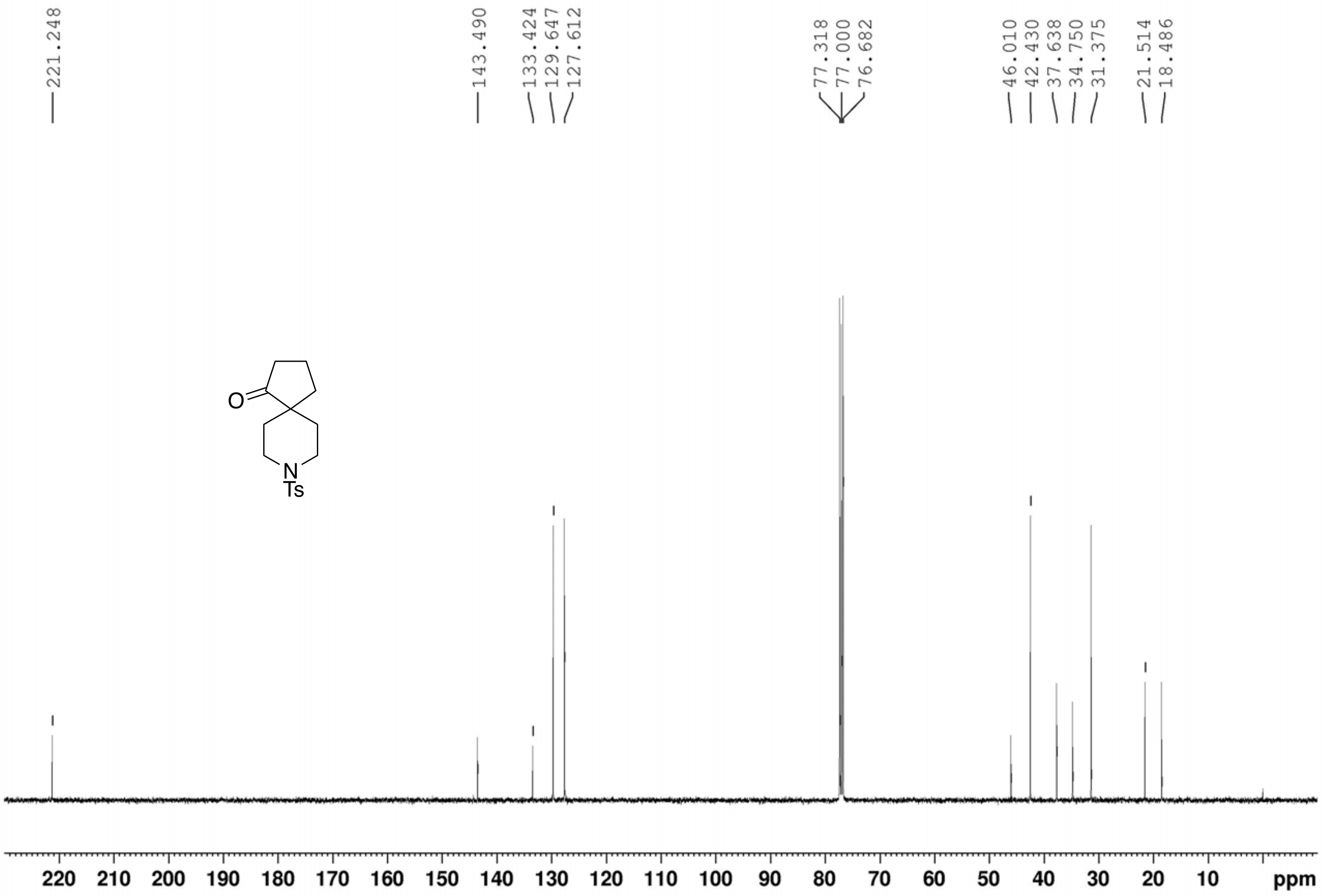

Supplementary Figure 137. ^{13}C NMR spectrum of **2C** (100.6 MHz, $CDCl_3$)

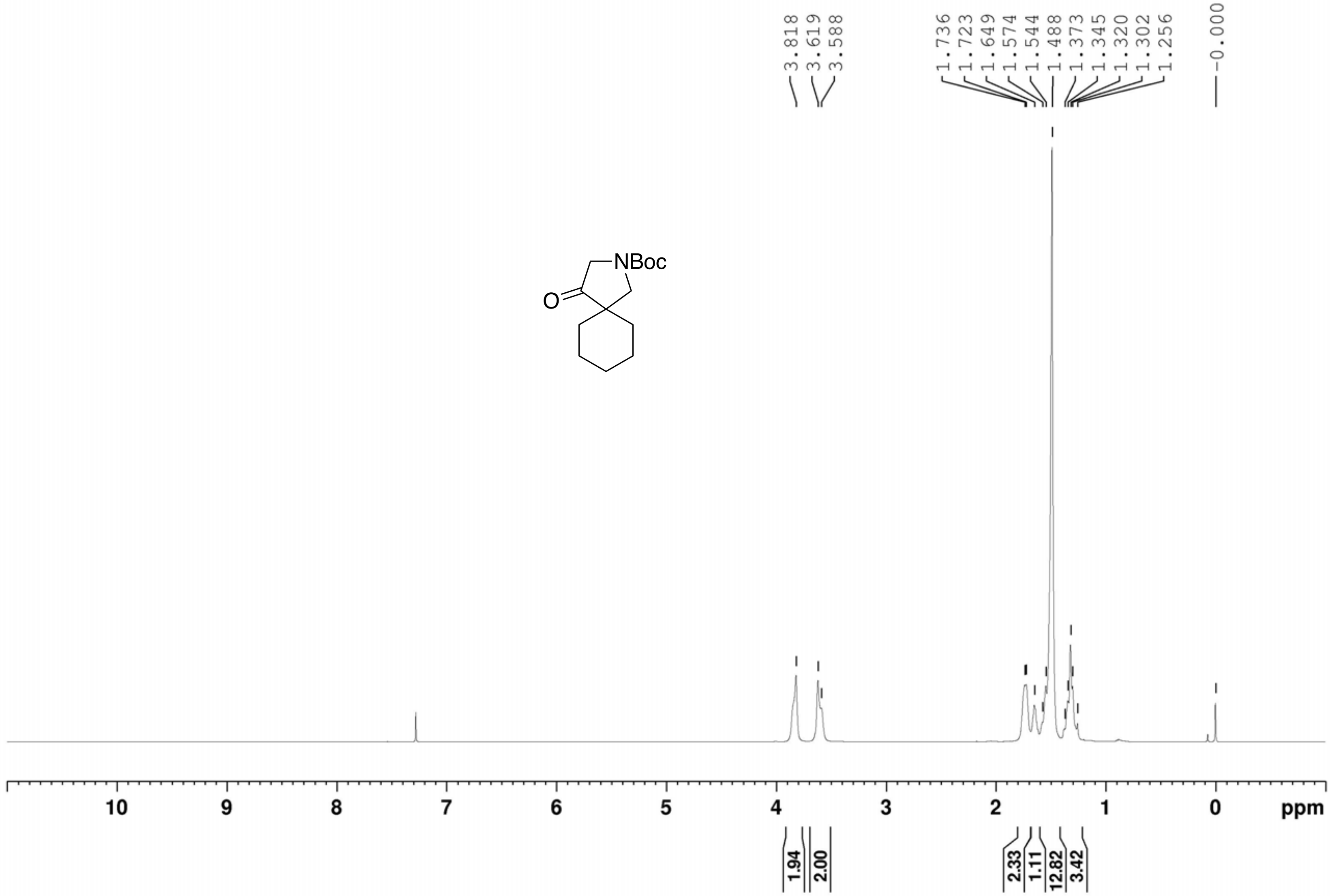

Supplementary Figure 138. ^{1}H NMR spectrum of **2D** (400 MHz, $CDCl_3$)

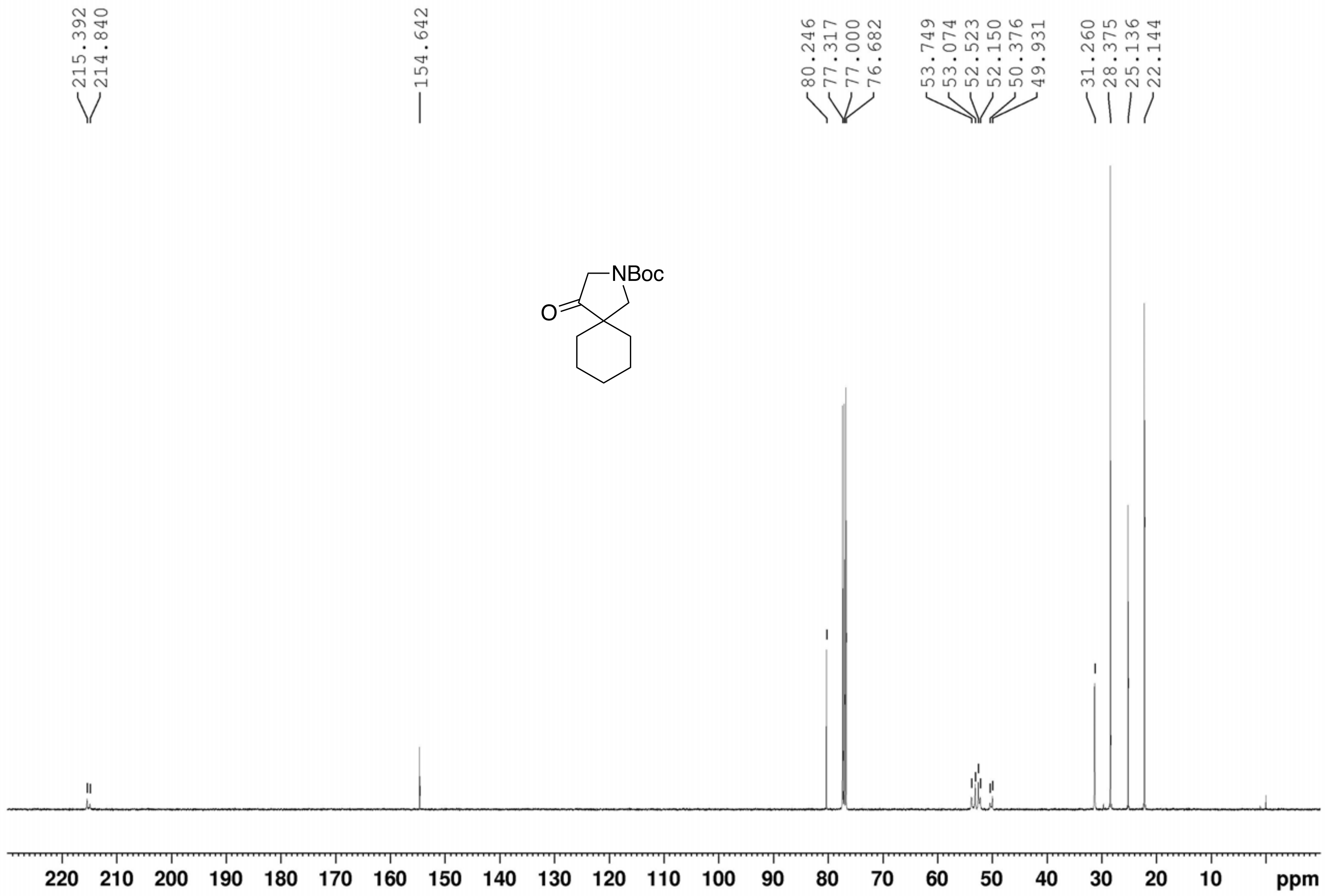

Supplementary Figure 139. ^{13}C NMR spectrum of **2D** (100.6 MHz, $CDCl_3$)

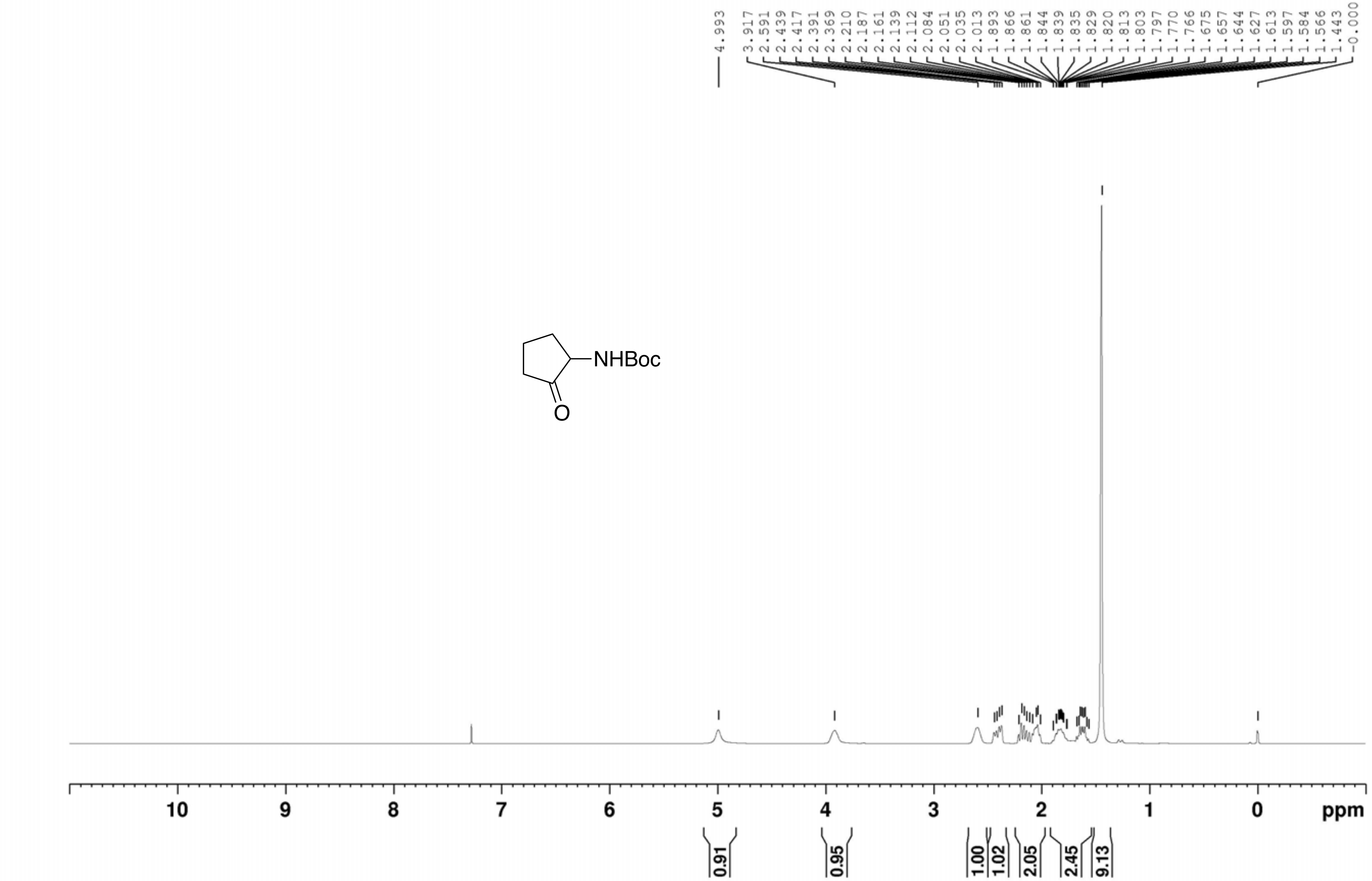

Supplementary Figure 140. ^{1}H NMR spectrum of **2E** (400 MHz, $CDCl_3$)

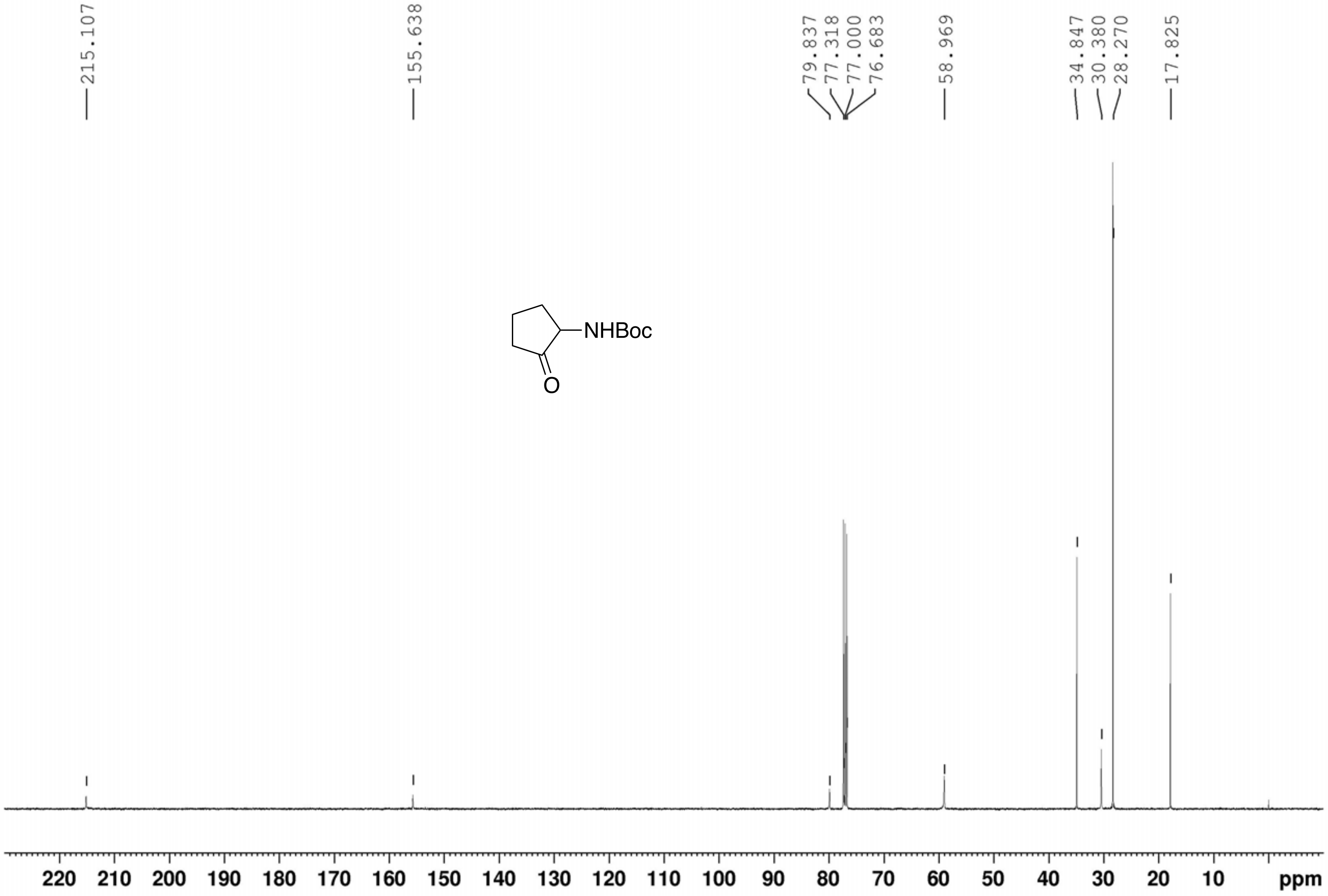

Supplementary Figure 141. ^{13}C NMR spectrum of **2E** (150.9 MHz, $CDCl_3$)

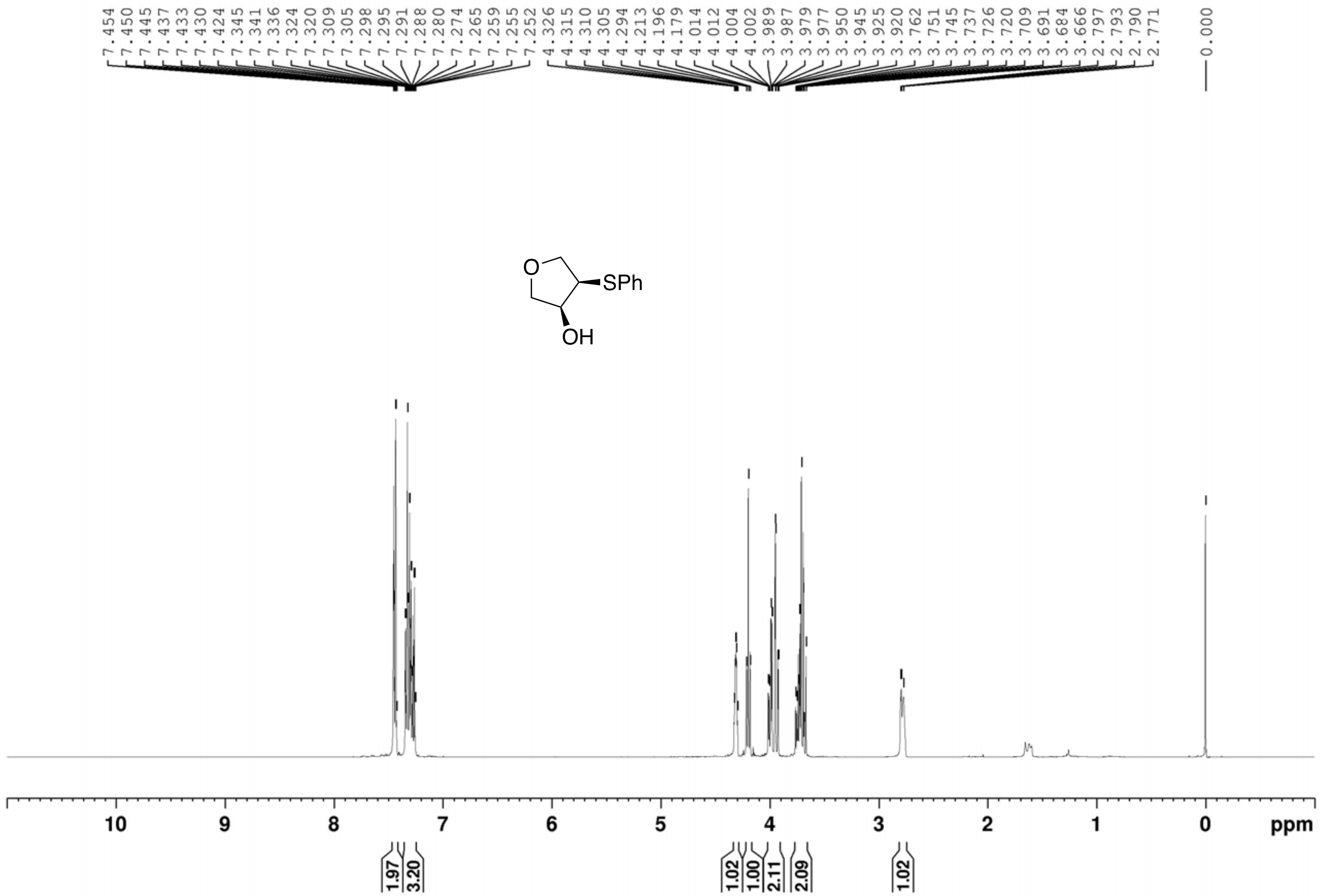

Supplementary Figure 142. ^{1}H NMR spectrum of **2F** (400 MHz, $CDCl_3$)

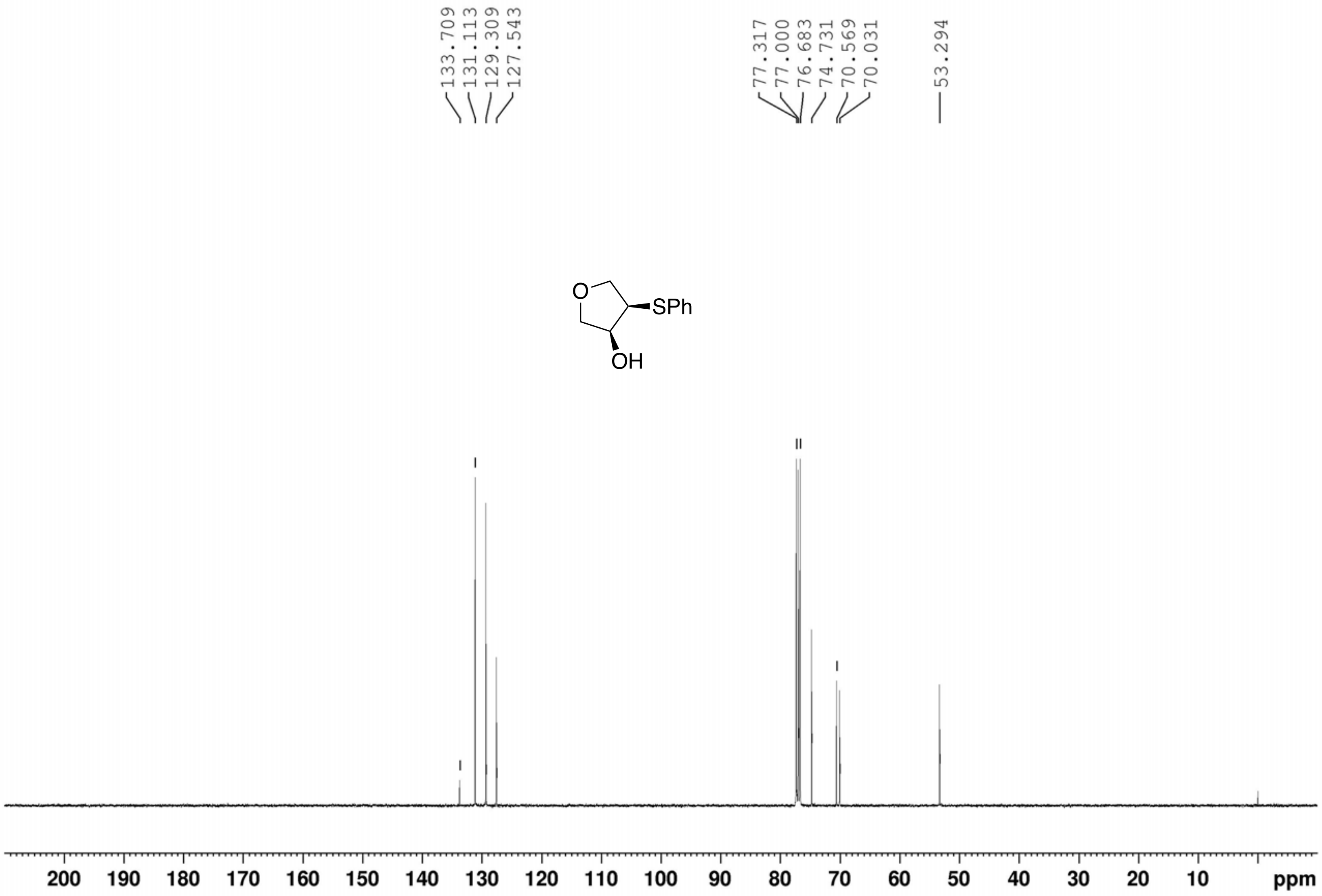

Supplementary Figure 143. ^{13}C NMR spectrum of **2F** (100.6 MHz, $CDCl_3$)

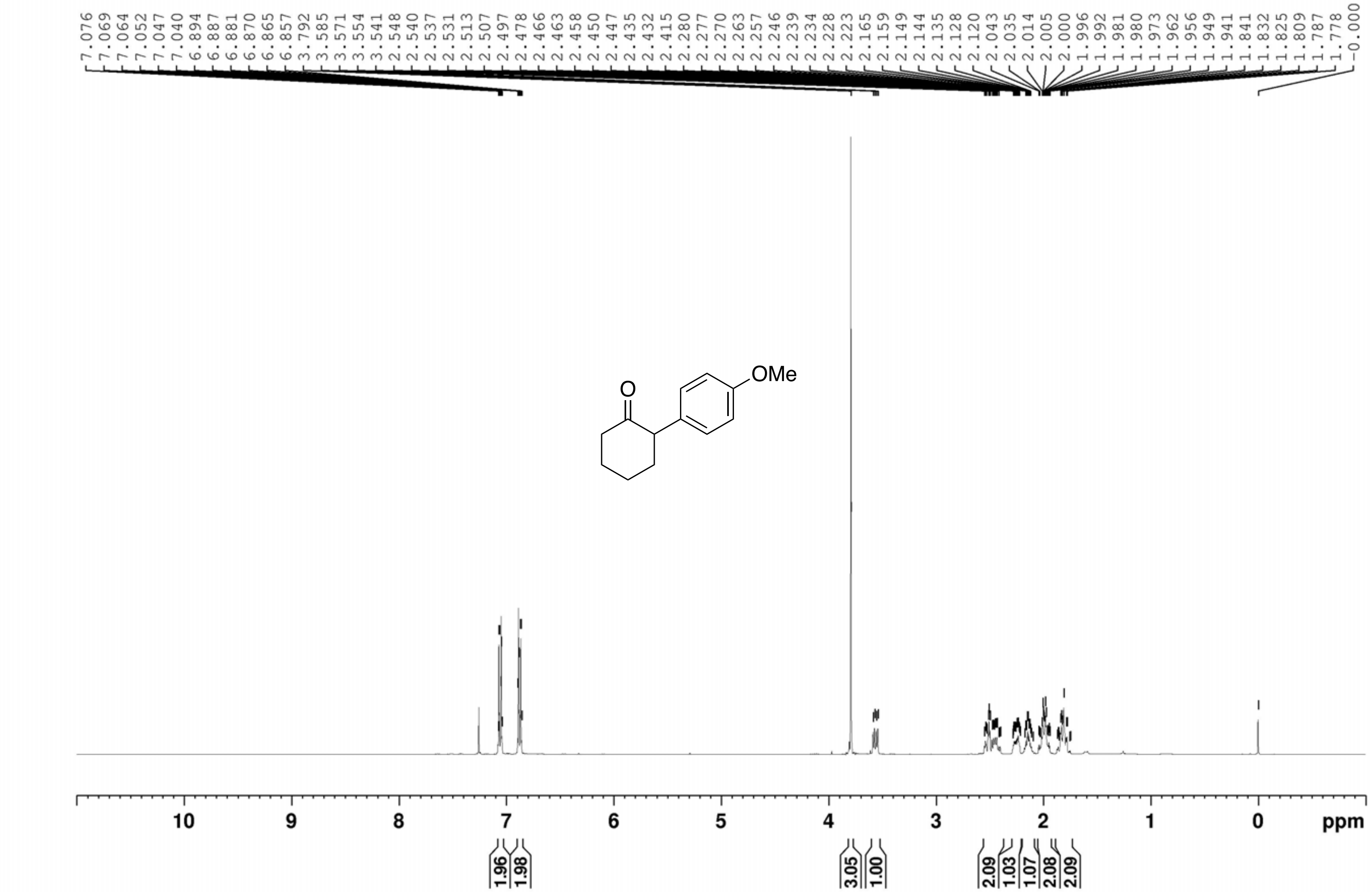

Supplementary Figure 144. ^{1}H NMR spectrum of **2G** (400 MHz, $CDCl_3$)

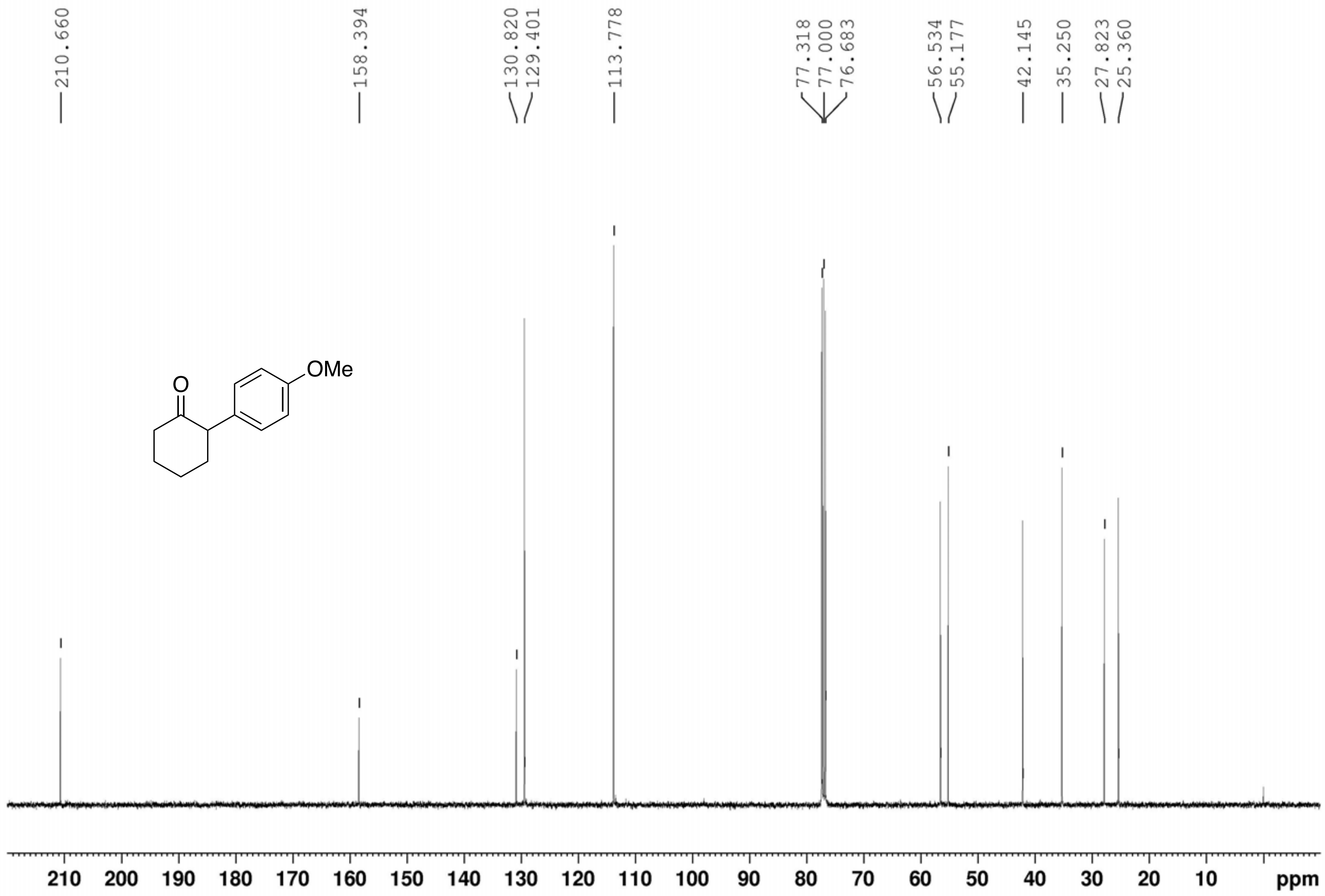

Supplementary Figure 145. ^{13}C NMR spectrum of **2G** (100.6 MHz, $CDCl_3$)

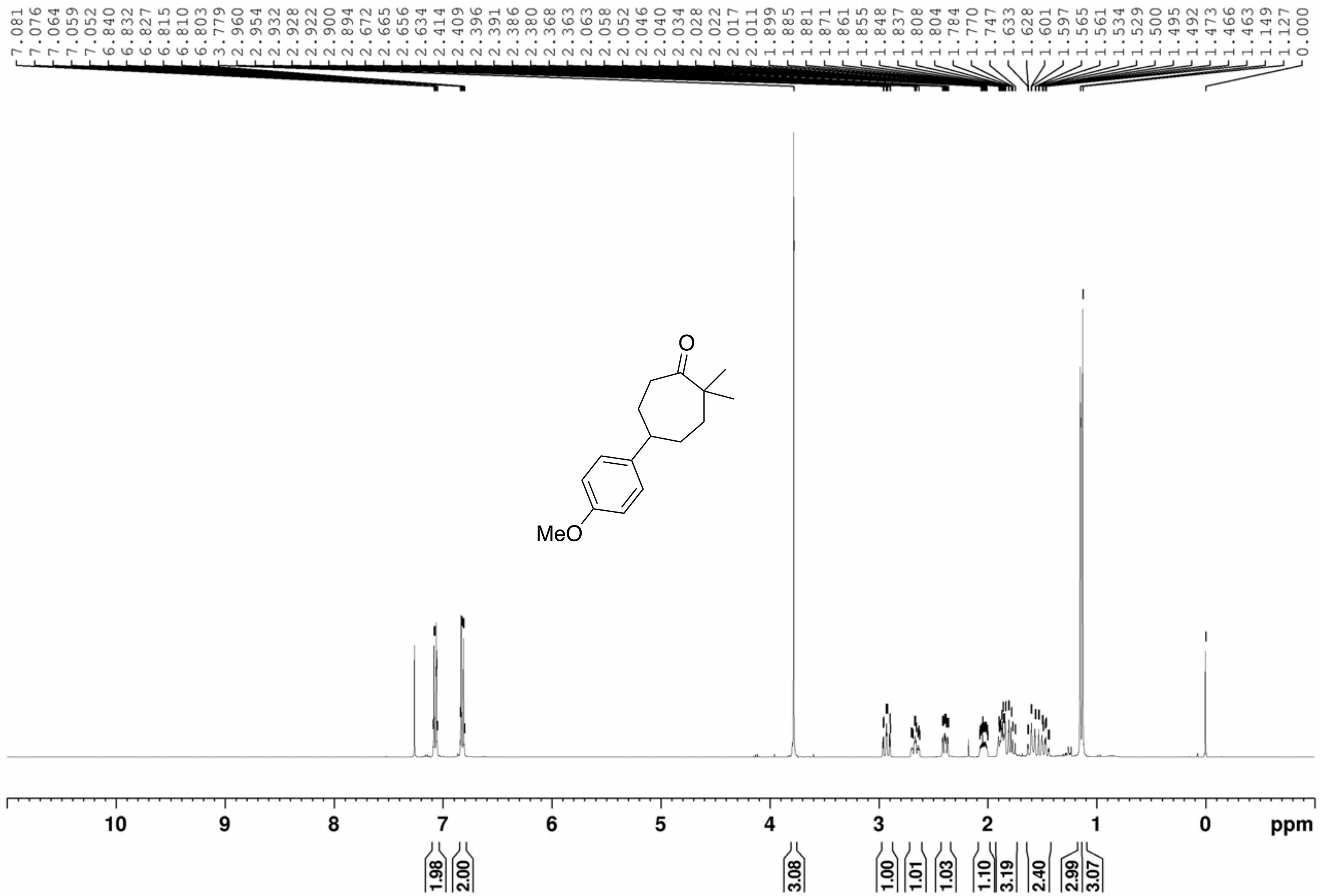

Supplementary Figure 146. ^{1}H NMR spectrum of **2H** (400 MHz, $CDCl_3$)

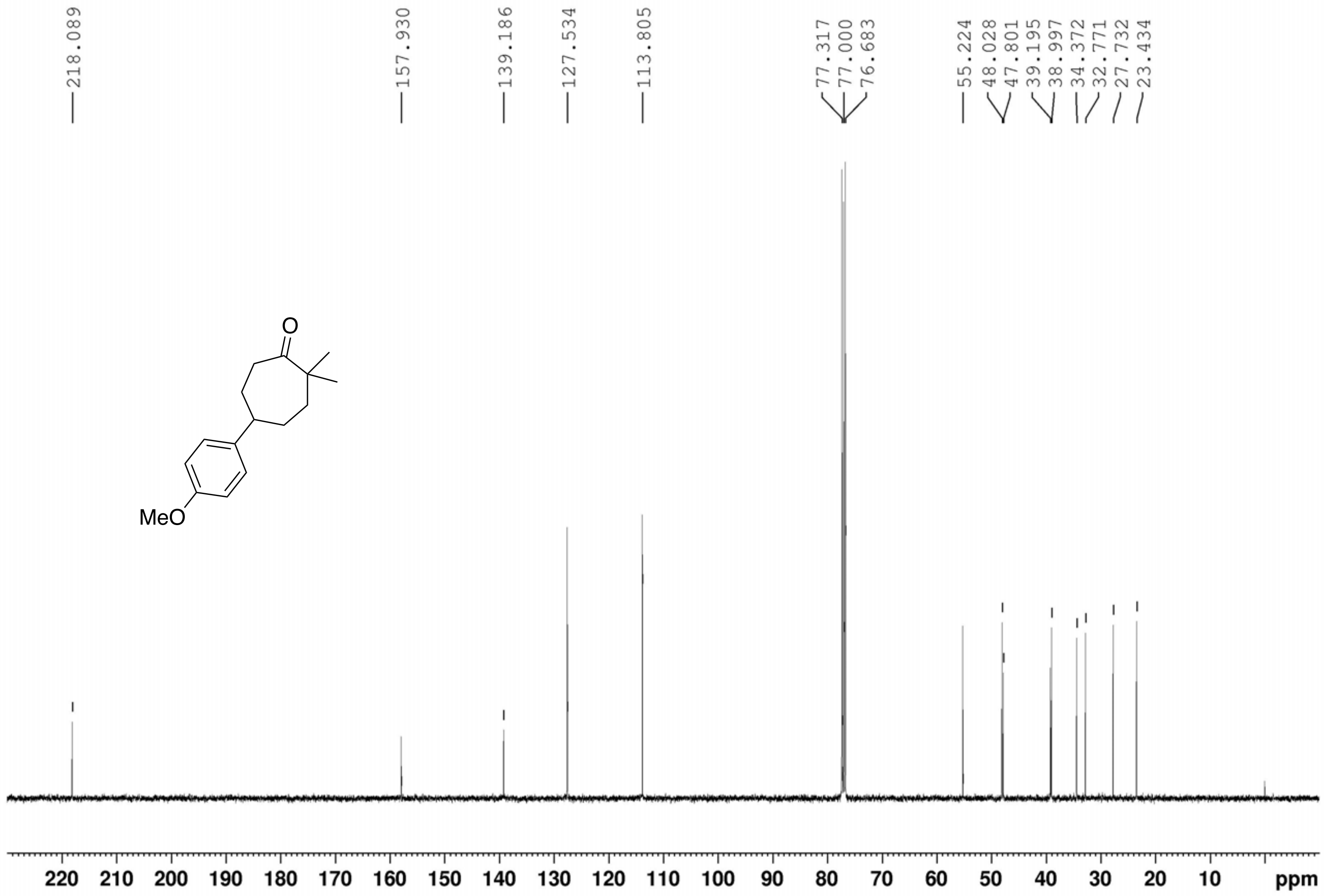

Supplementary Figure 147. ^{13}C NMR spectrum of **2H** (100.6 MHz, $CDCl_3$)

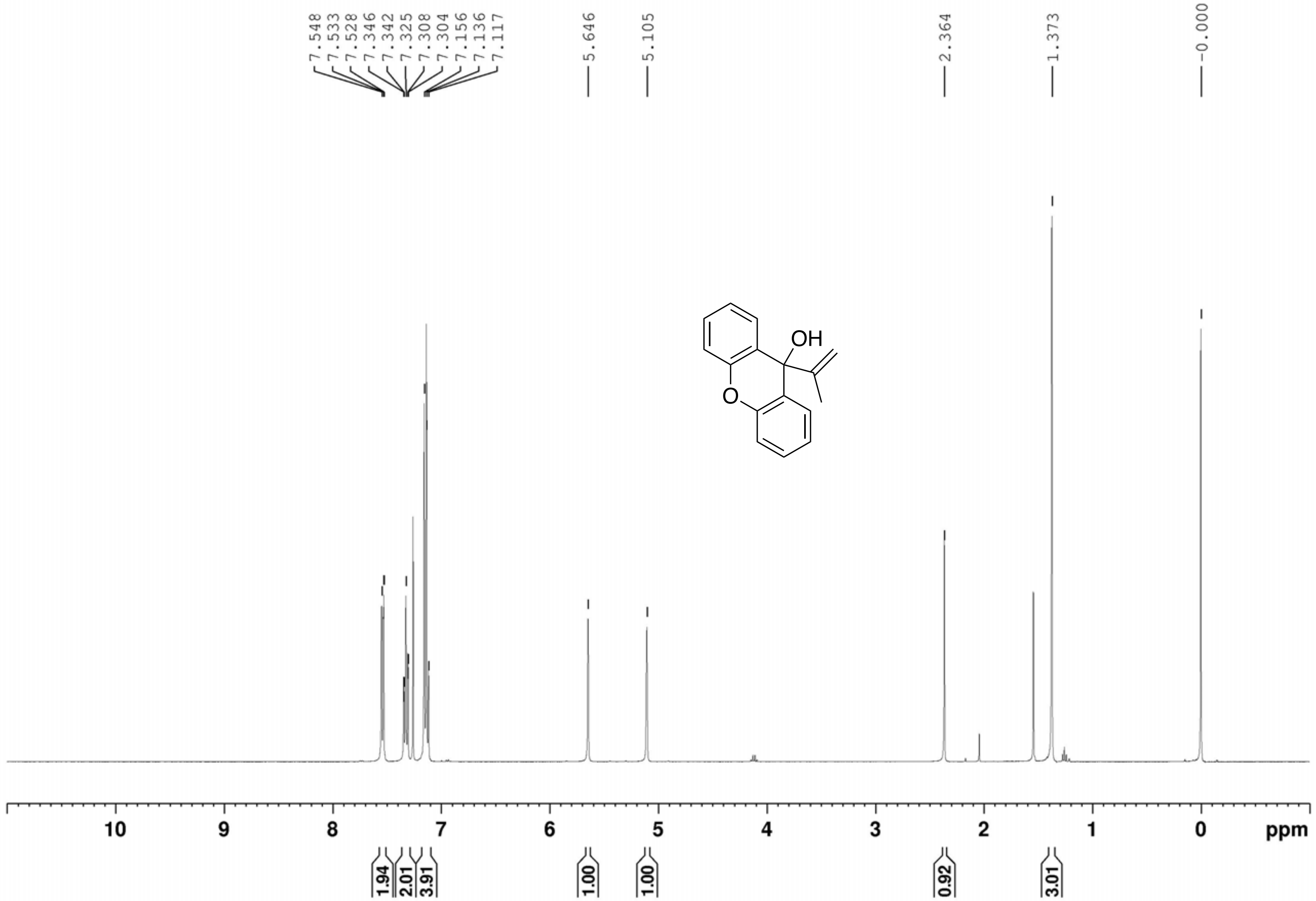

Supplementary Figure 148. ^{1}H NMR spectrum of **4a** (400 MHz, $CDCl_3$)

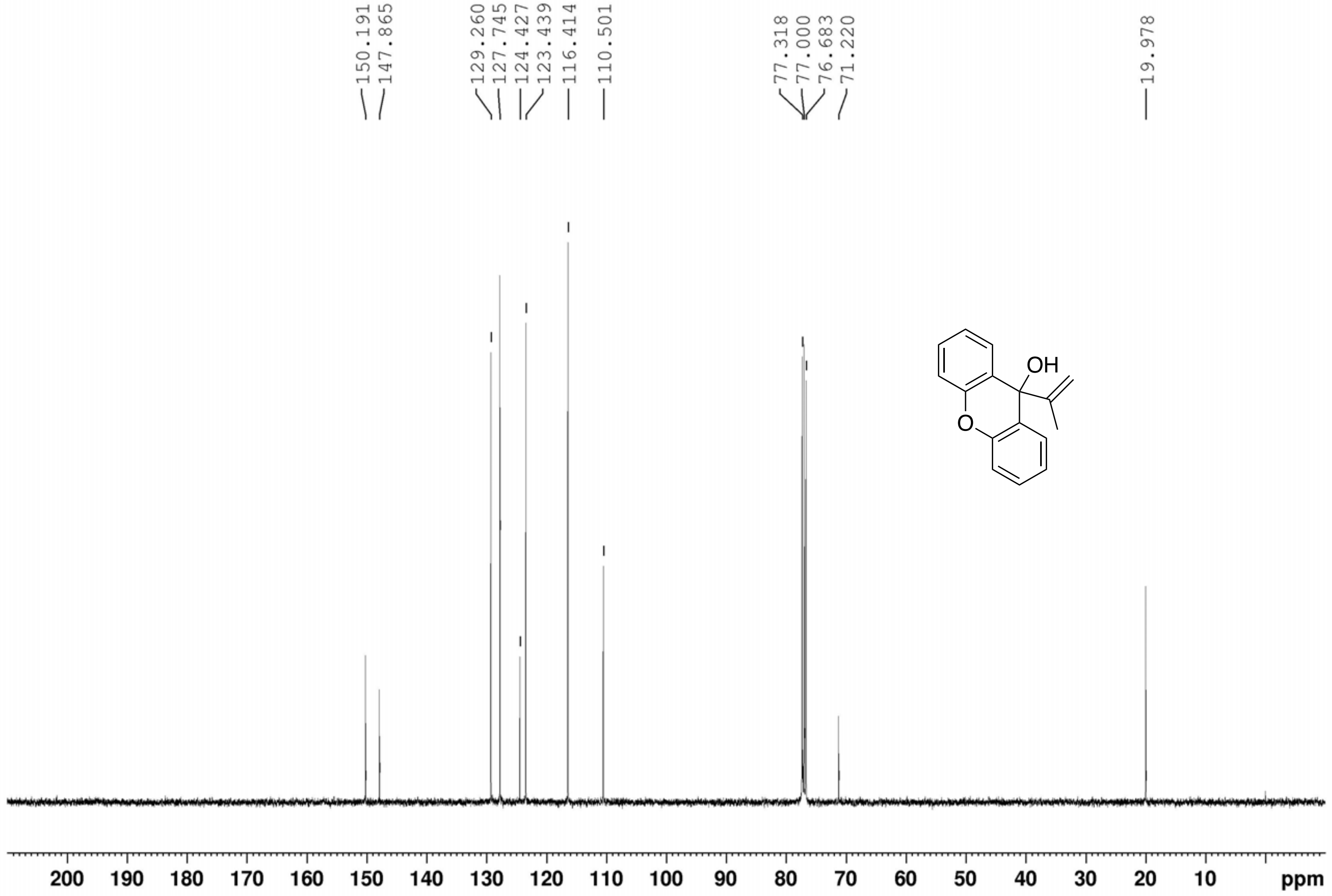

Supplementary Figure 149. ^{13}C NMR spectrum of **4a** (100.6 MHz, $CDCl_3$)

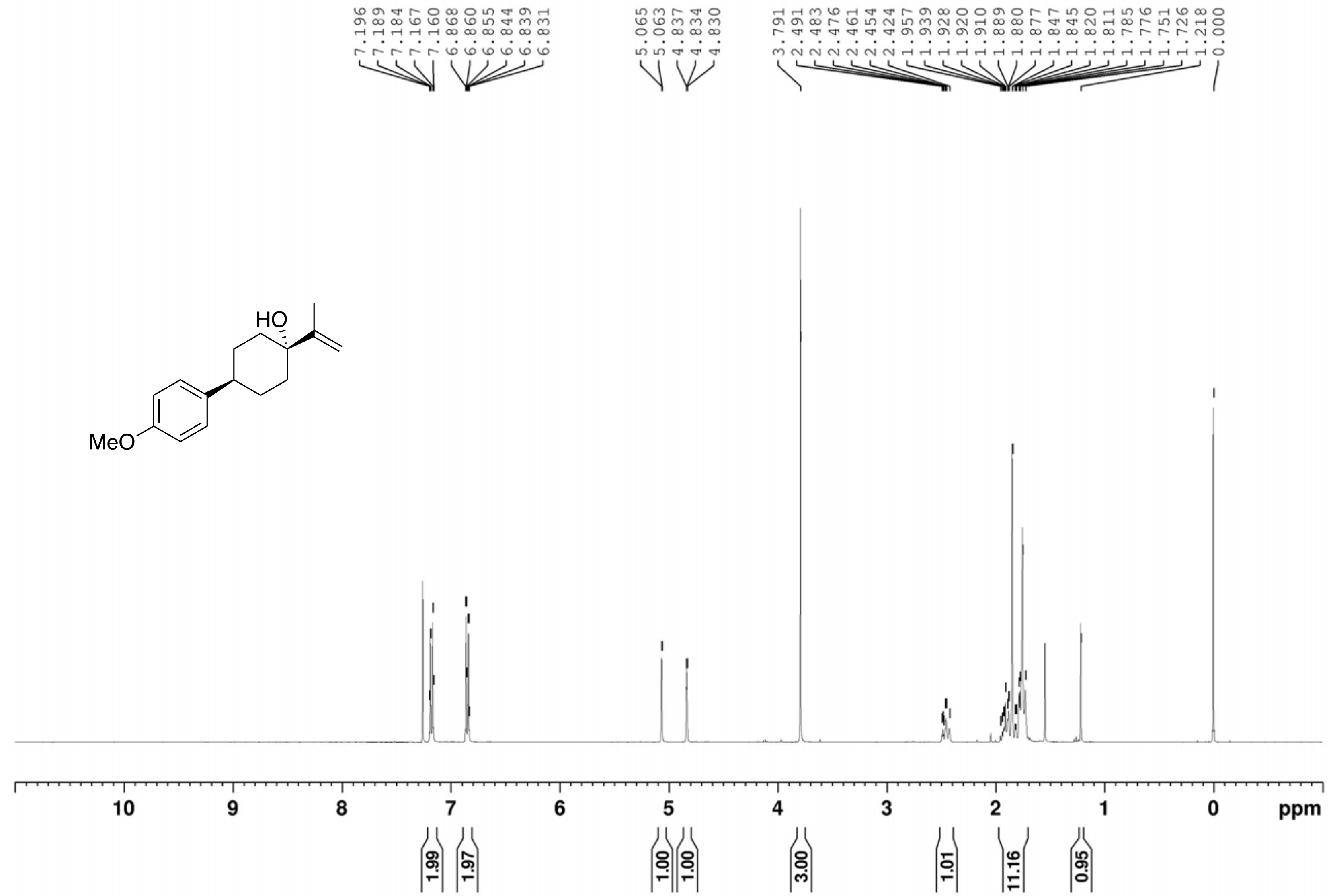

Supplementary Figure 150. ^{1}H NMR spectrum of **4c** (400 MHz, $CDCl_3$)

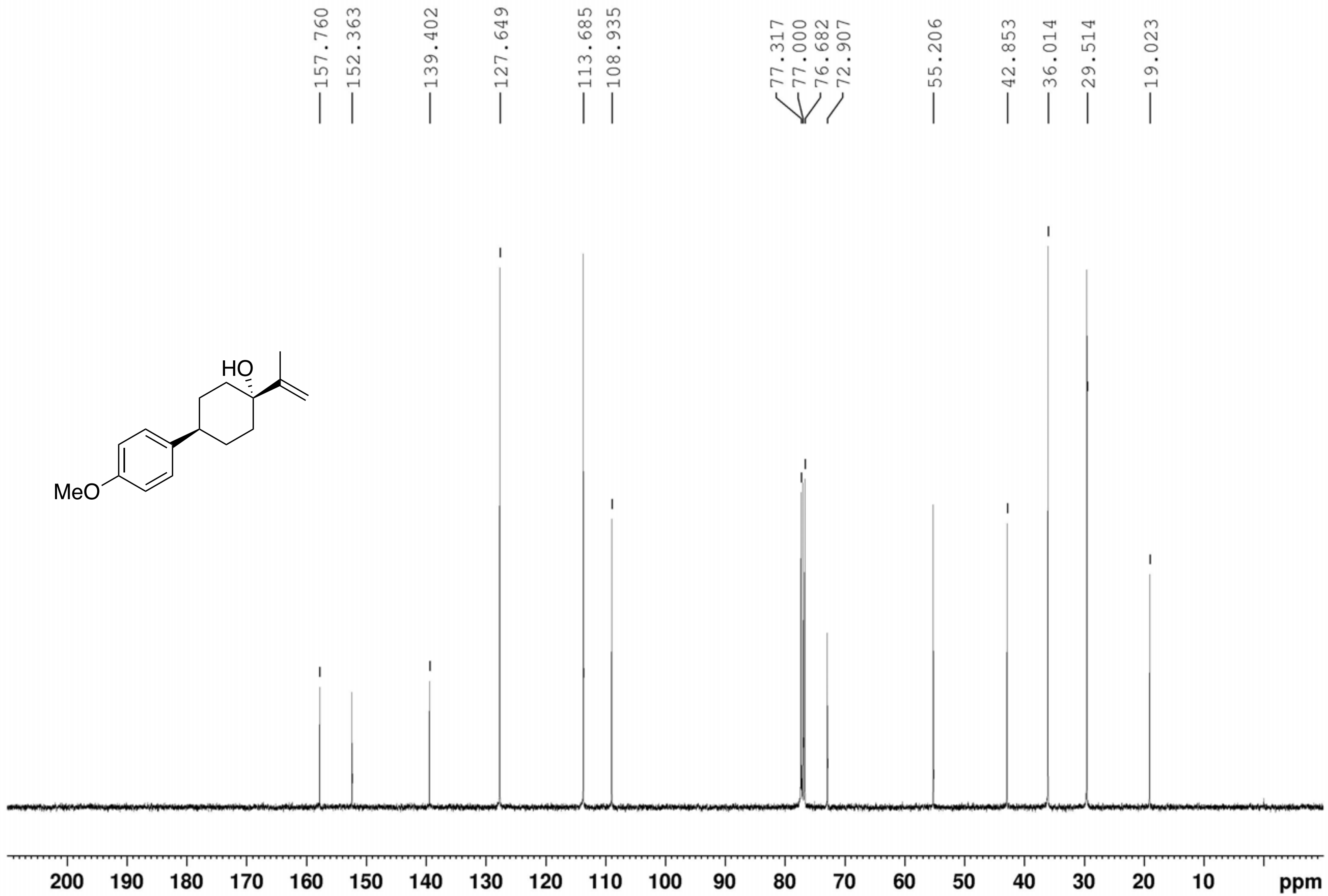

Supplementary Figure 151. ^{13}C NMR spectrum of **4c** (100.6 MHz, $CDCl_3$)

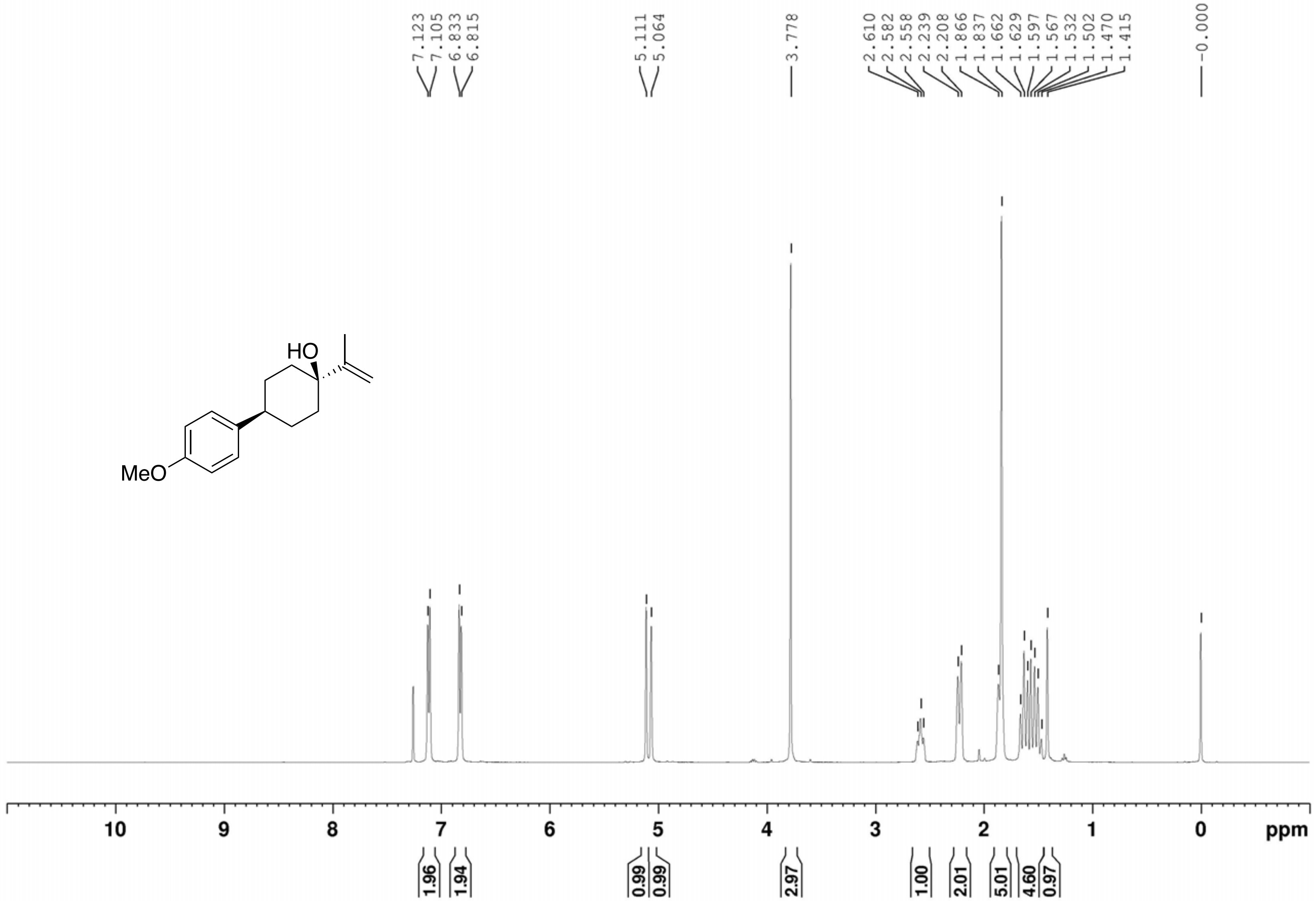

Supplementary Figure 152. ^{1}H NMR spectrum of **4c’** (400 MHz, $CDCl_3$)

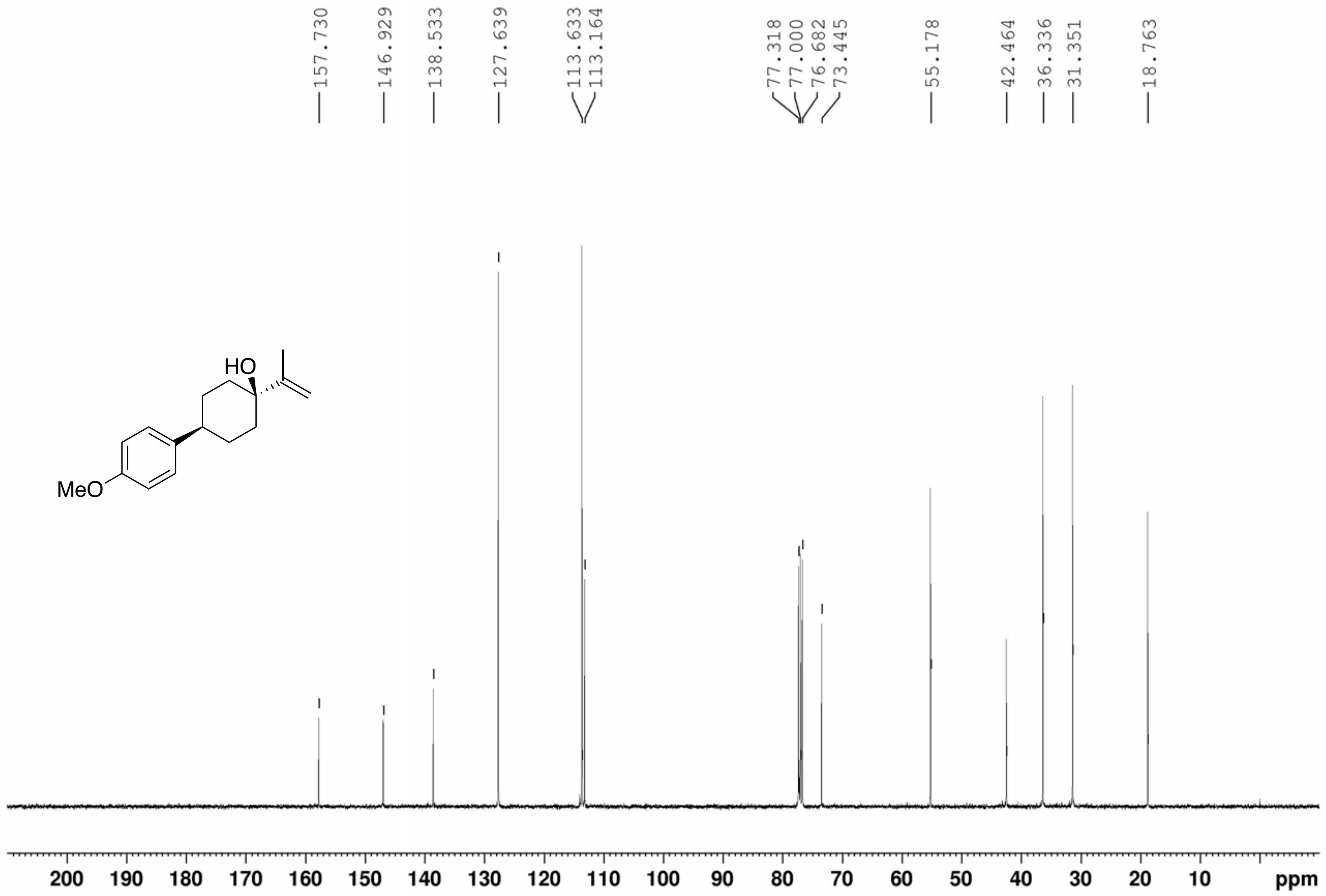

Supplementary Figure 153. ^{13}C NMR spectrum of **4c'** (100.6 MHz, $CDCl_3$)

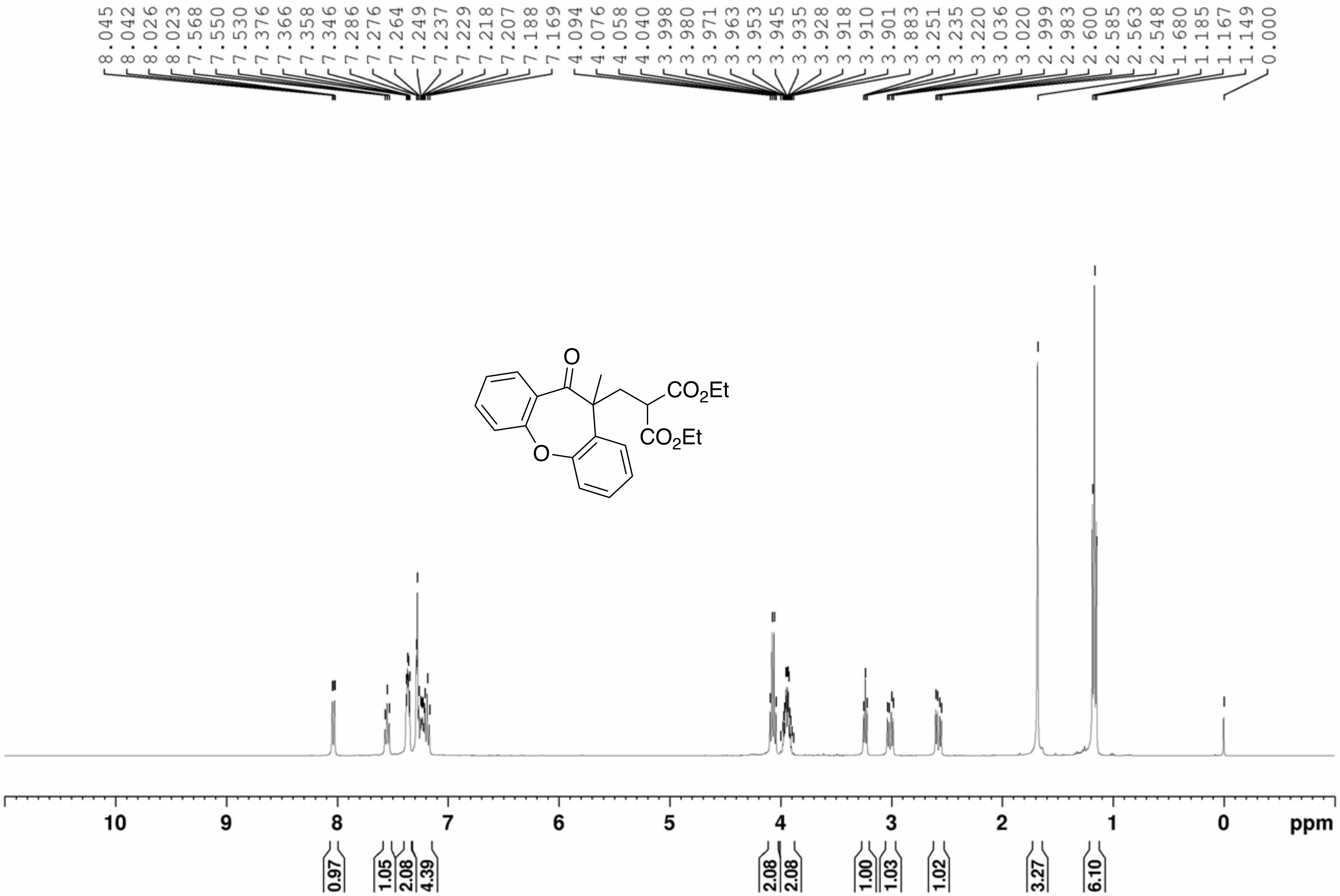

Supplementary Figure 154. ^{1}H NMR spectrum of **6aa** (400 MHz, $CDCl_3$)

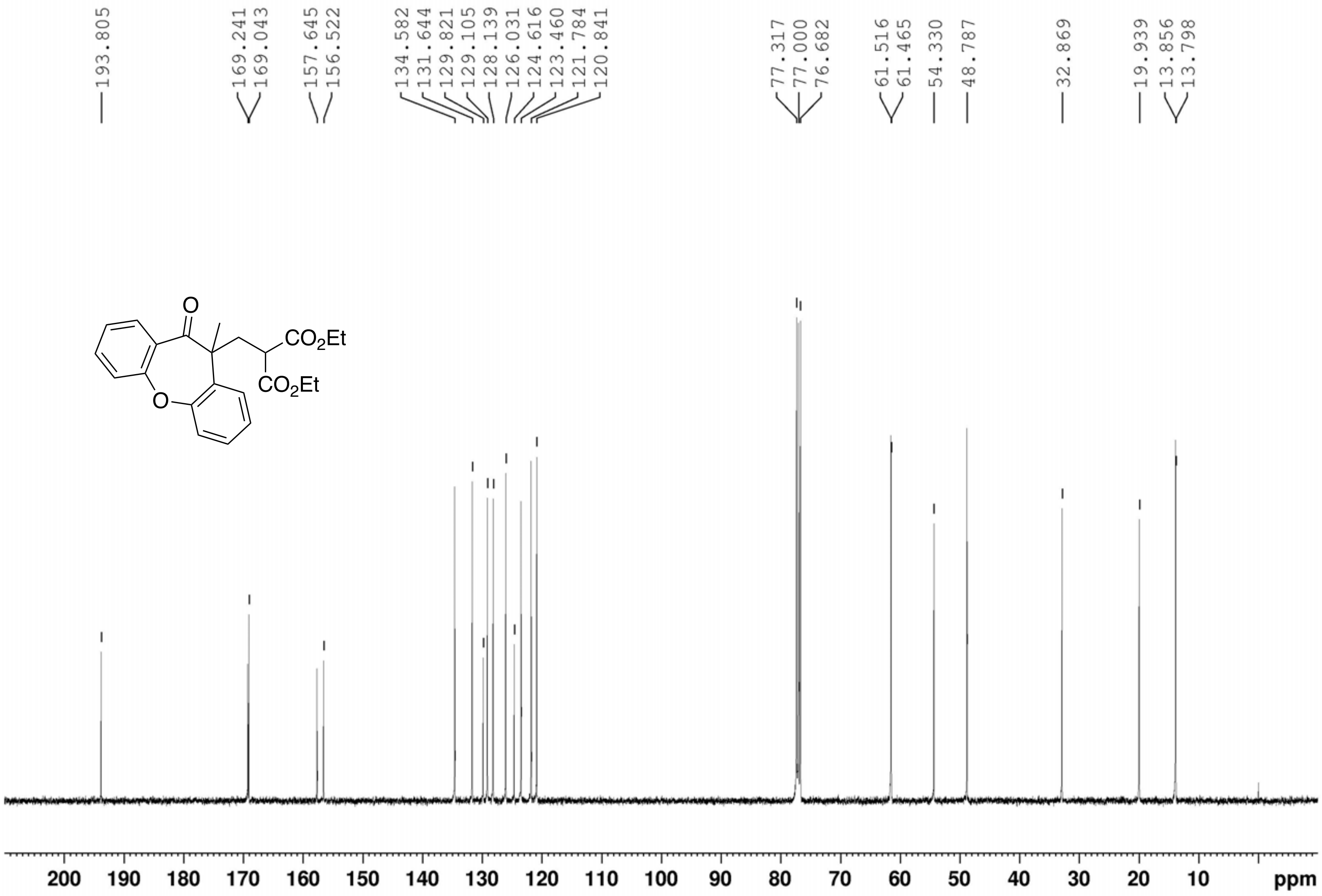

Supplementary Figure 155. ^{13}C NMR spectrum of **6aa** (100.6 MHz, $CDCl_3$)

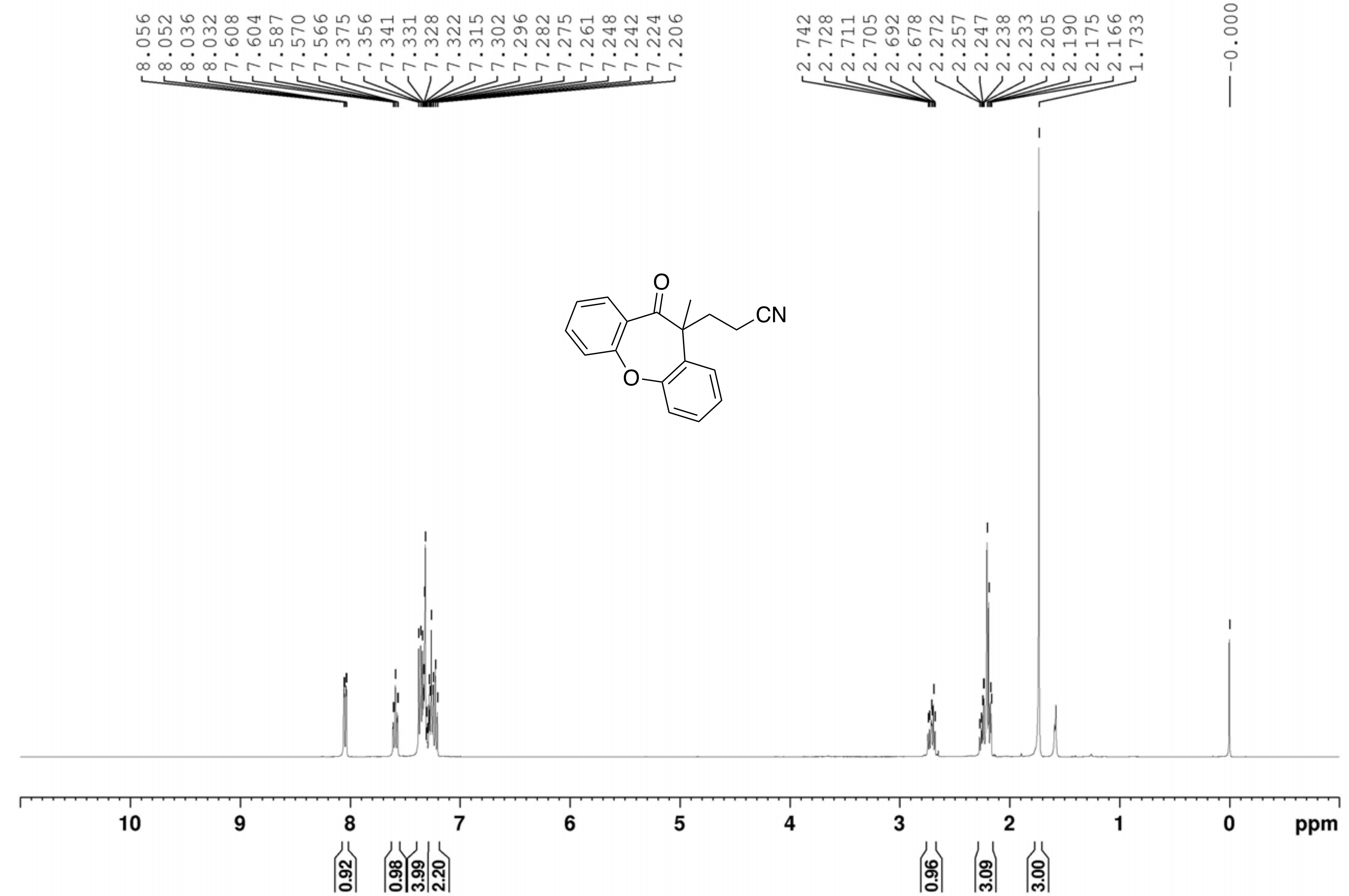

Supplementary Figure 156. ^{1}H NMR spectrum of **6ab** (400 MHz, $CDCl_3$)

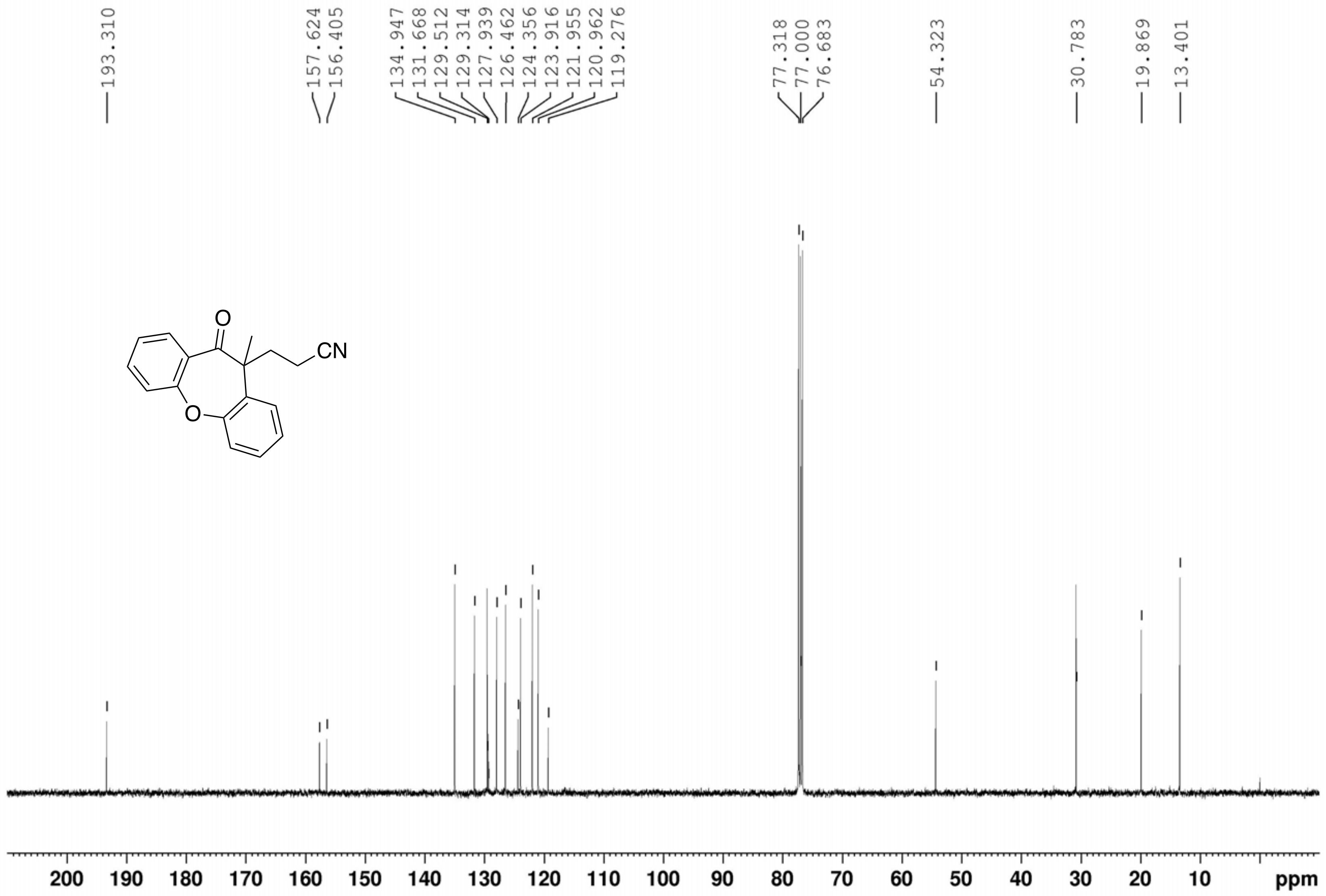

Supplementary Figure 157. ^{13}C NMR spectrum of **6ab** (100.6 MHz, $CDCl_3$)

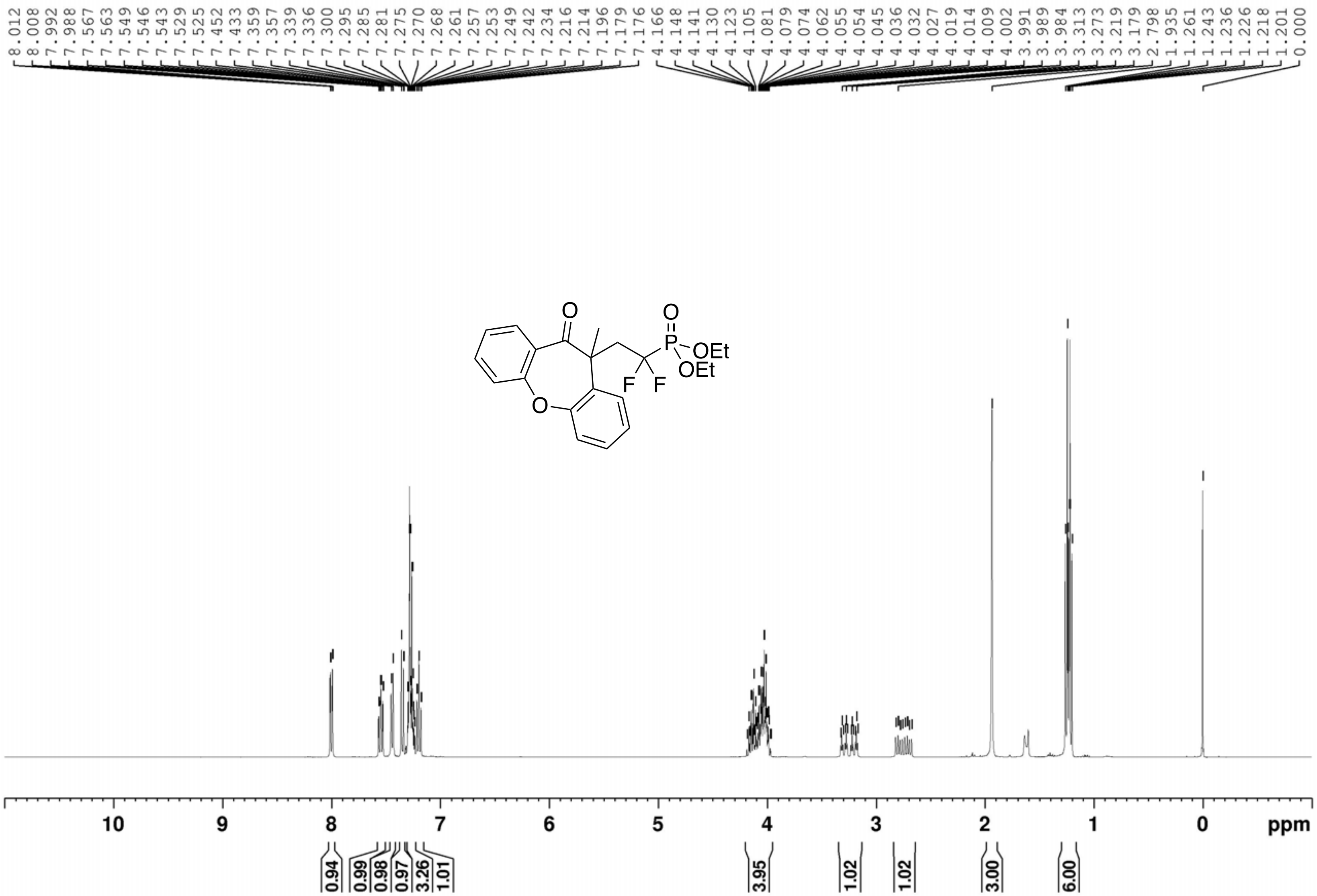

Supplementary Figure 158. ^{1}H NMR spectrum of **6ac** (400 MHz, $CDCl_3$)

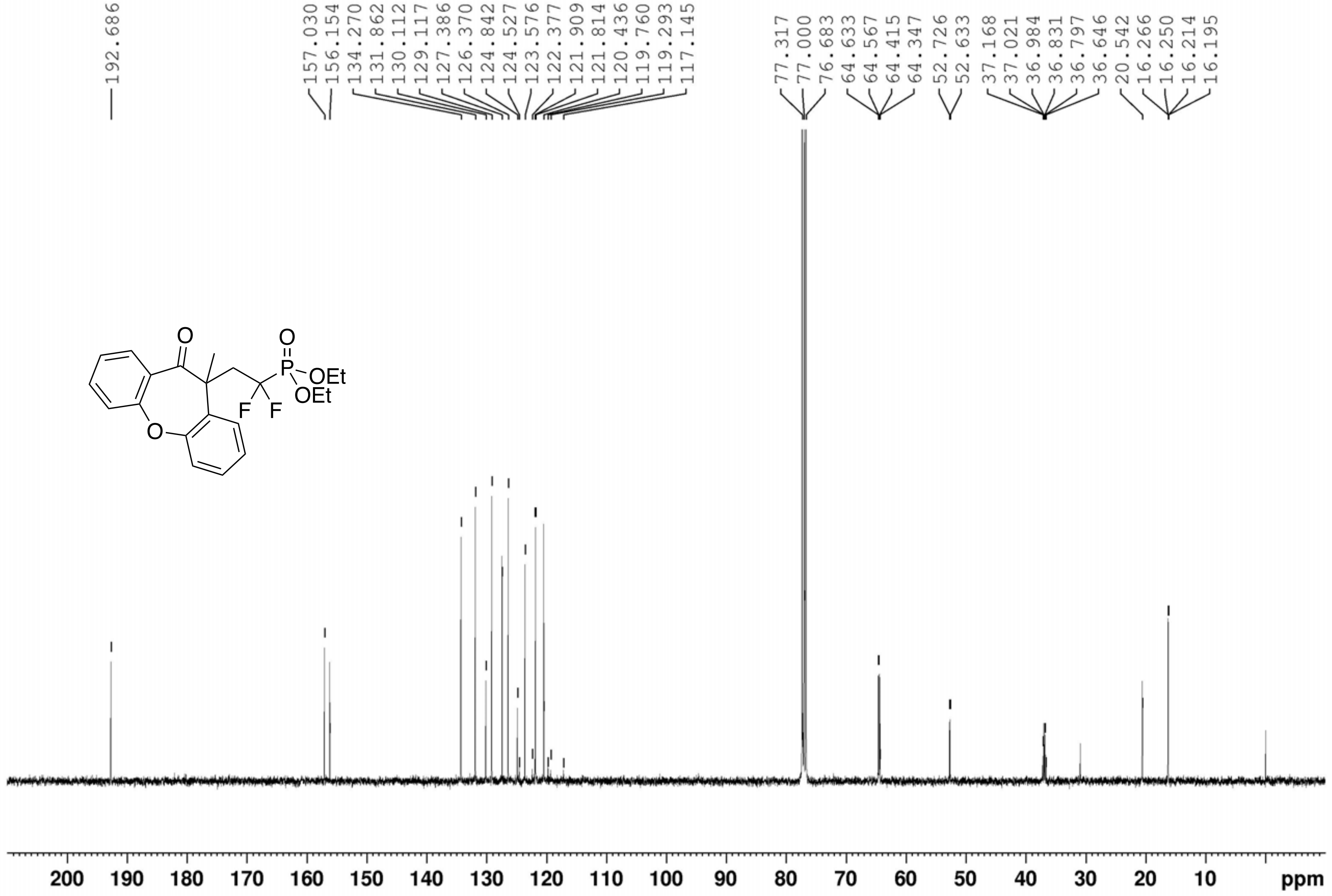

Supplementary Figure 159. ^{13}C NMR spectrum of **6ac** (100.6 MHz, $CDCl_3$)

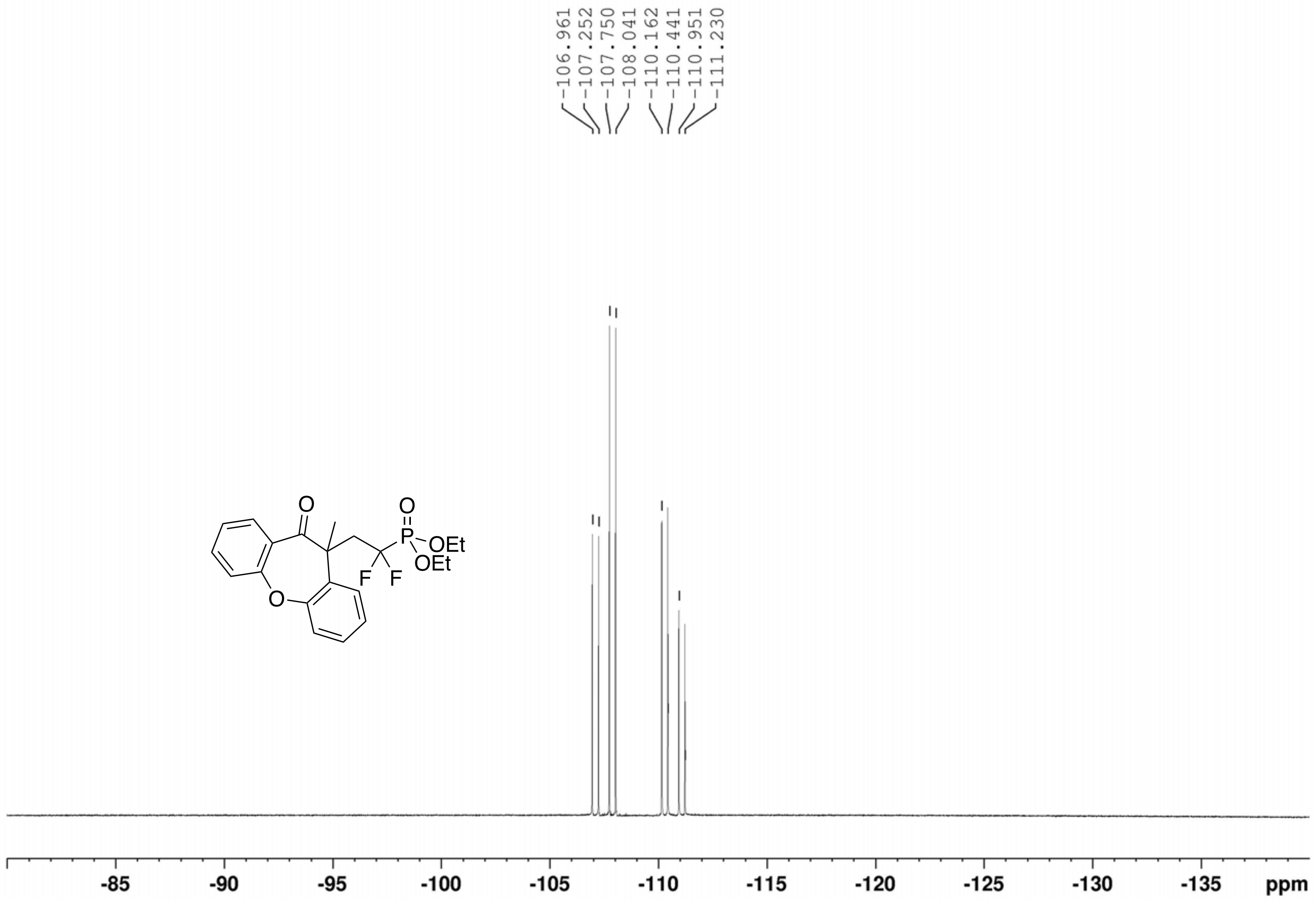

Supplementary Figure 160. ^{19}F NMR spectrum of **6ac** (376 MHz, $CDCl_3$)

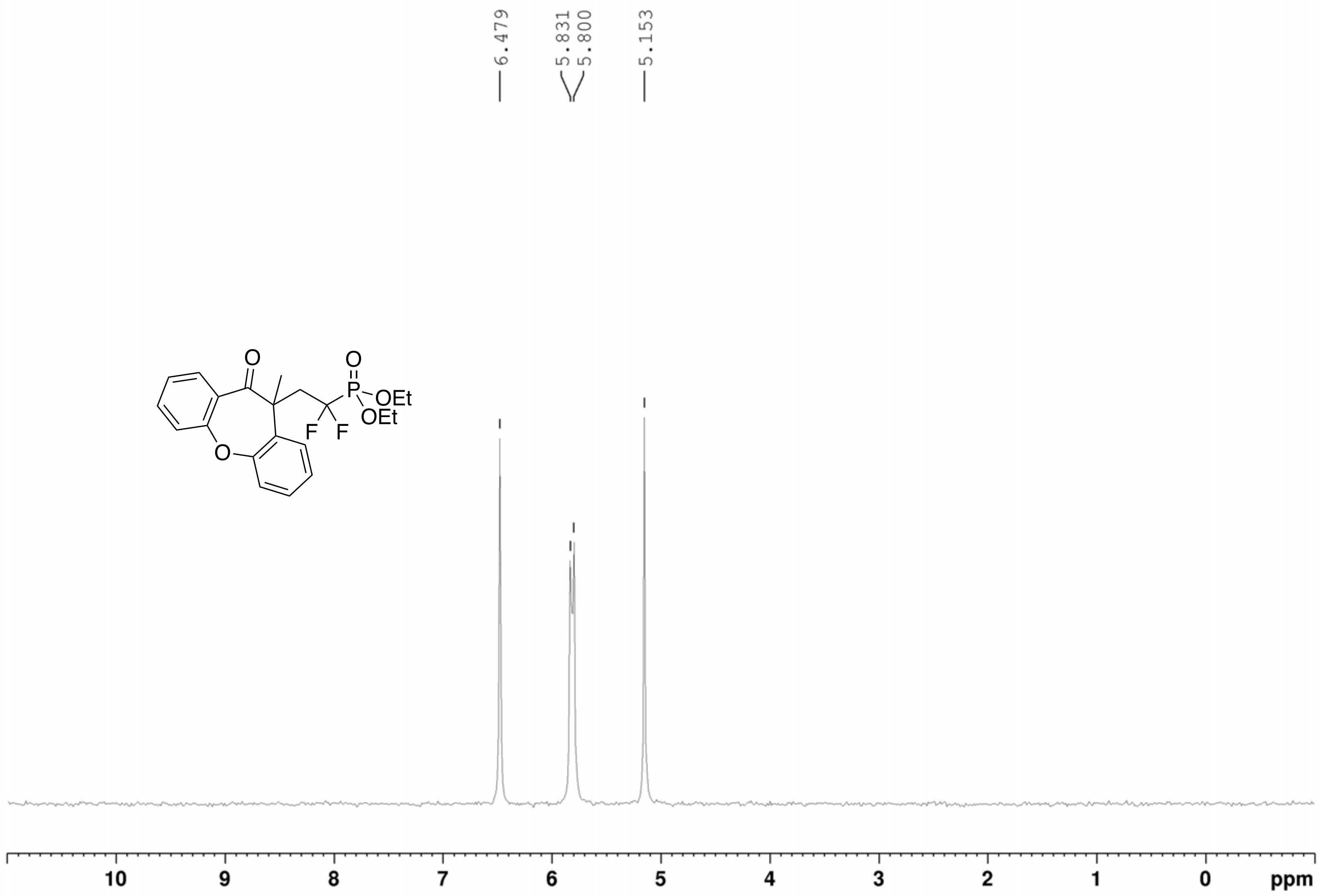

Supplementary Figure 161. ^{31}P NMR spectrum of **6ac** (162 MHz, $CDCl_3$)

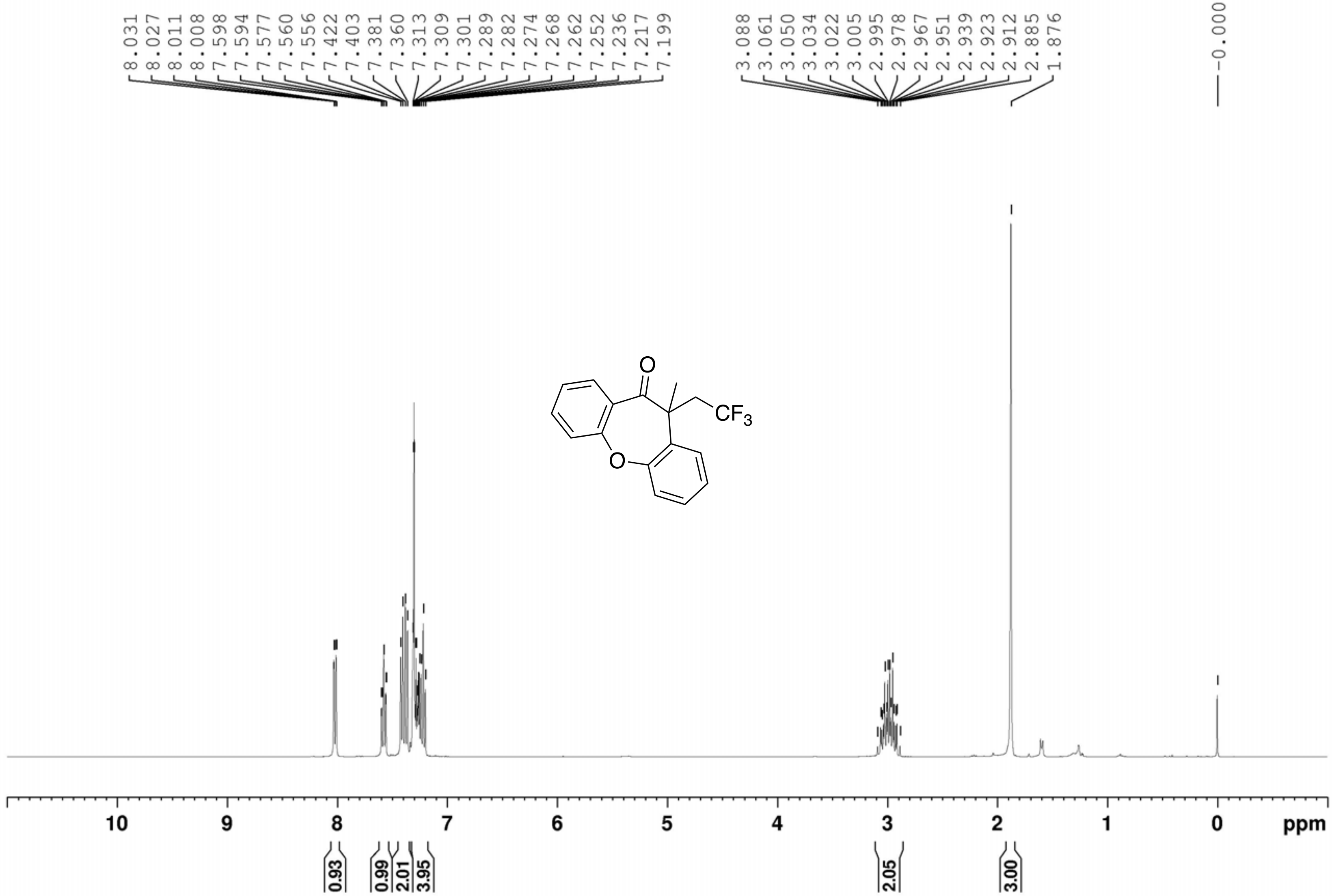

Supplementary Figure 162. ^{1}H NMR spectrum of **6ad** (400 MHz, $CDCl_3$)

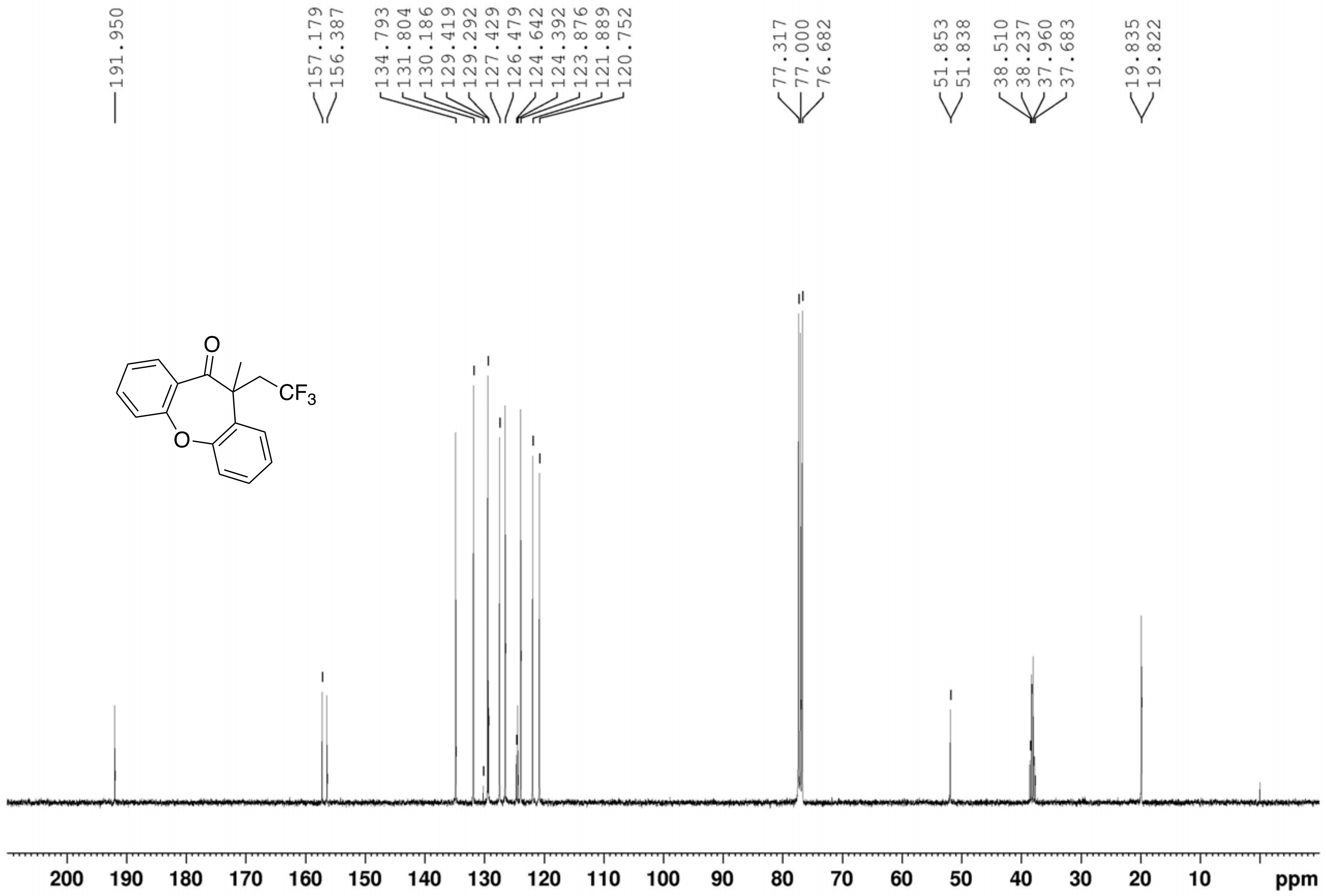

Supplementary Figure 163. ^{13}C NMR spectrum of **6ad** (100.6 MHz, $CDCl_3$)

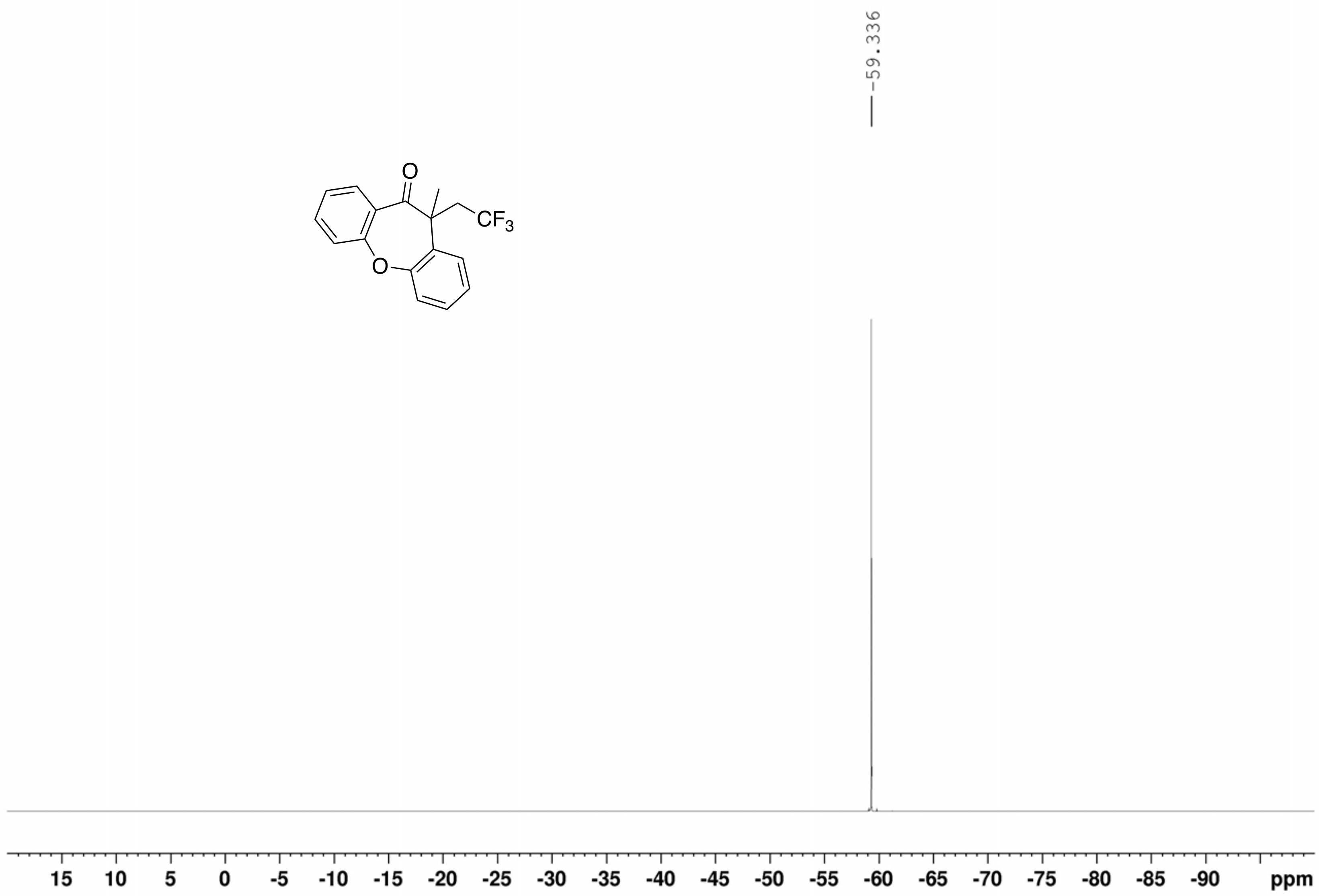

Supplementary Figure 164. ^{19}F NMR spectrum of **6ad**

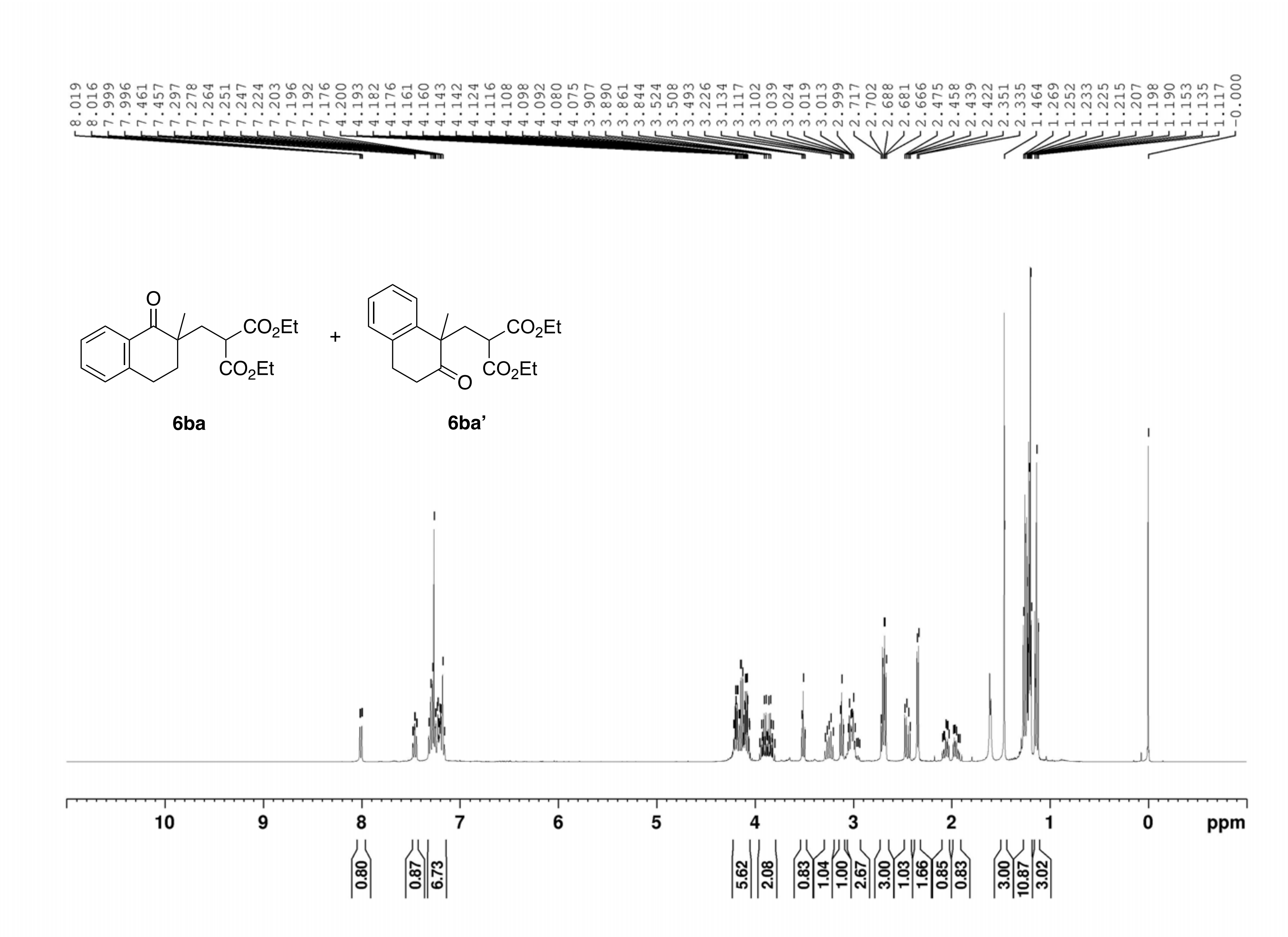

Supplementary Figure 165. ^{1}H NMR spectrum of **6ba + 6ba’** (400 MHz, $CDCl_3$)

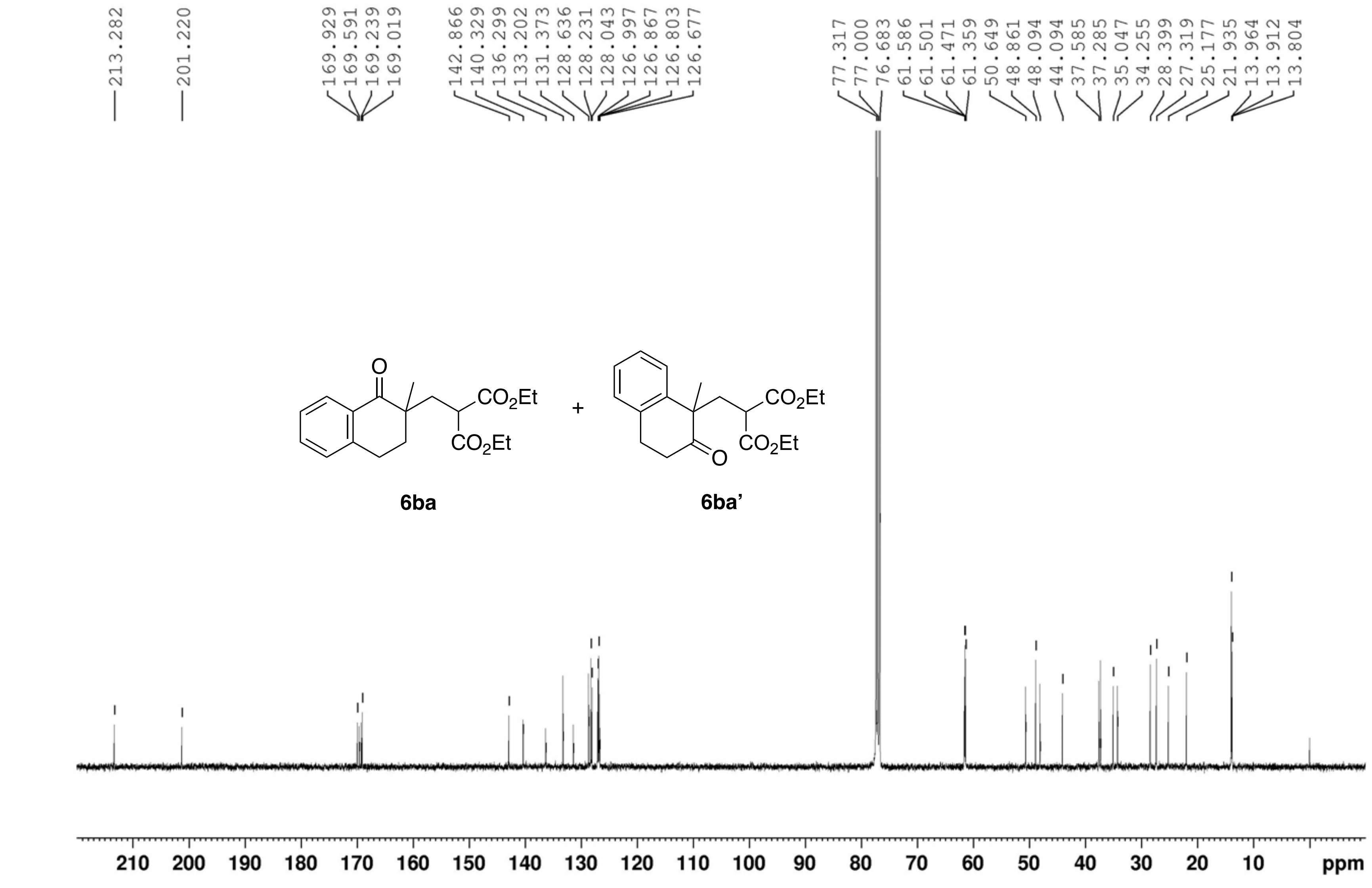

Supplementary Figure 166. ^{13}C NMR spectrum of **6ba + 6ba’** (100.6 MHz, $CDCl_3$)

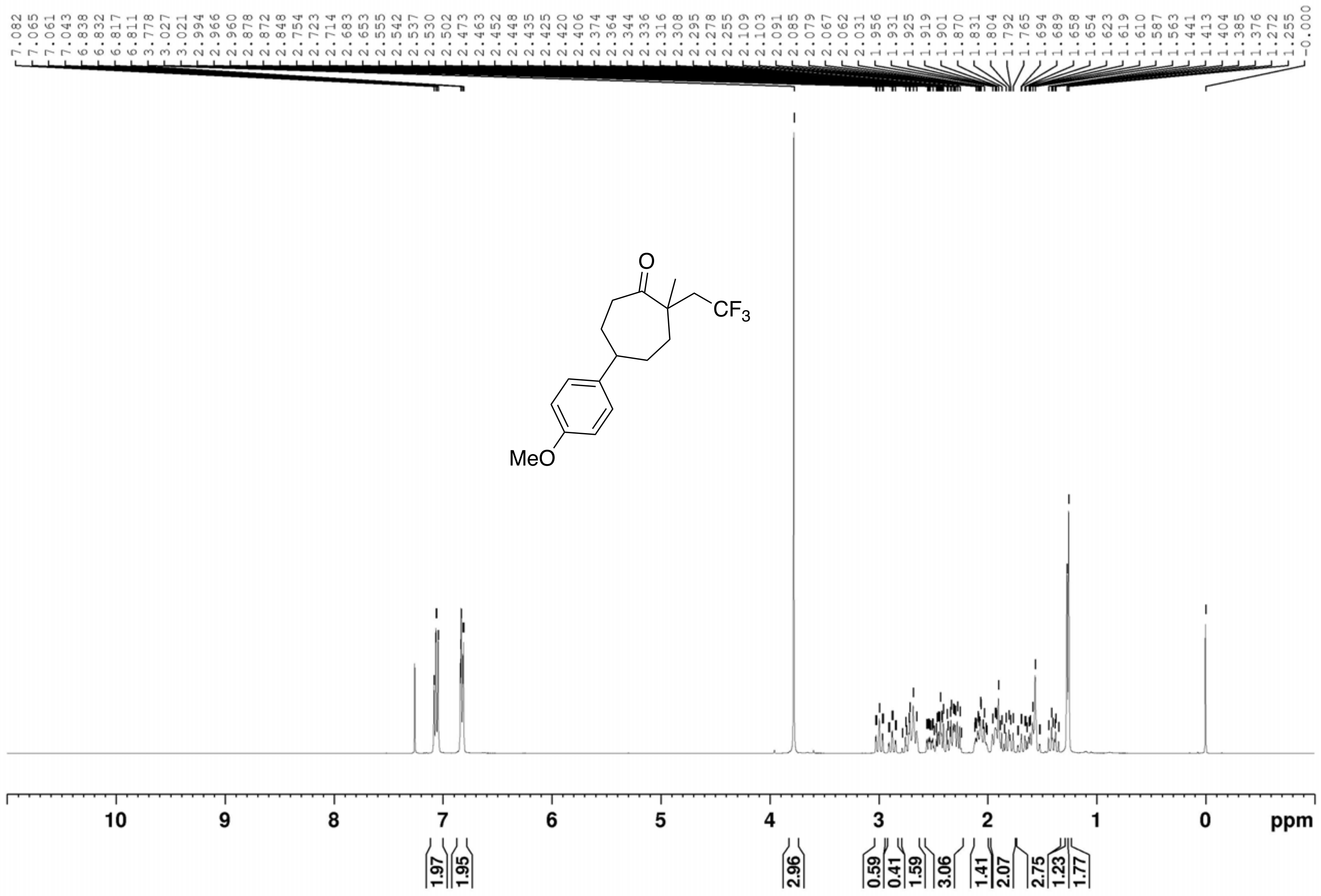

Supplementary Figure 167. ^{1}H NMR spectrum of **6cd** (400 MHz, $CDCl_3$)

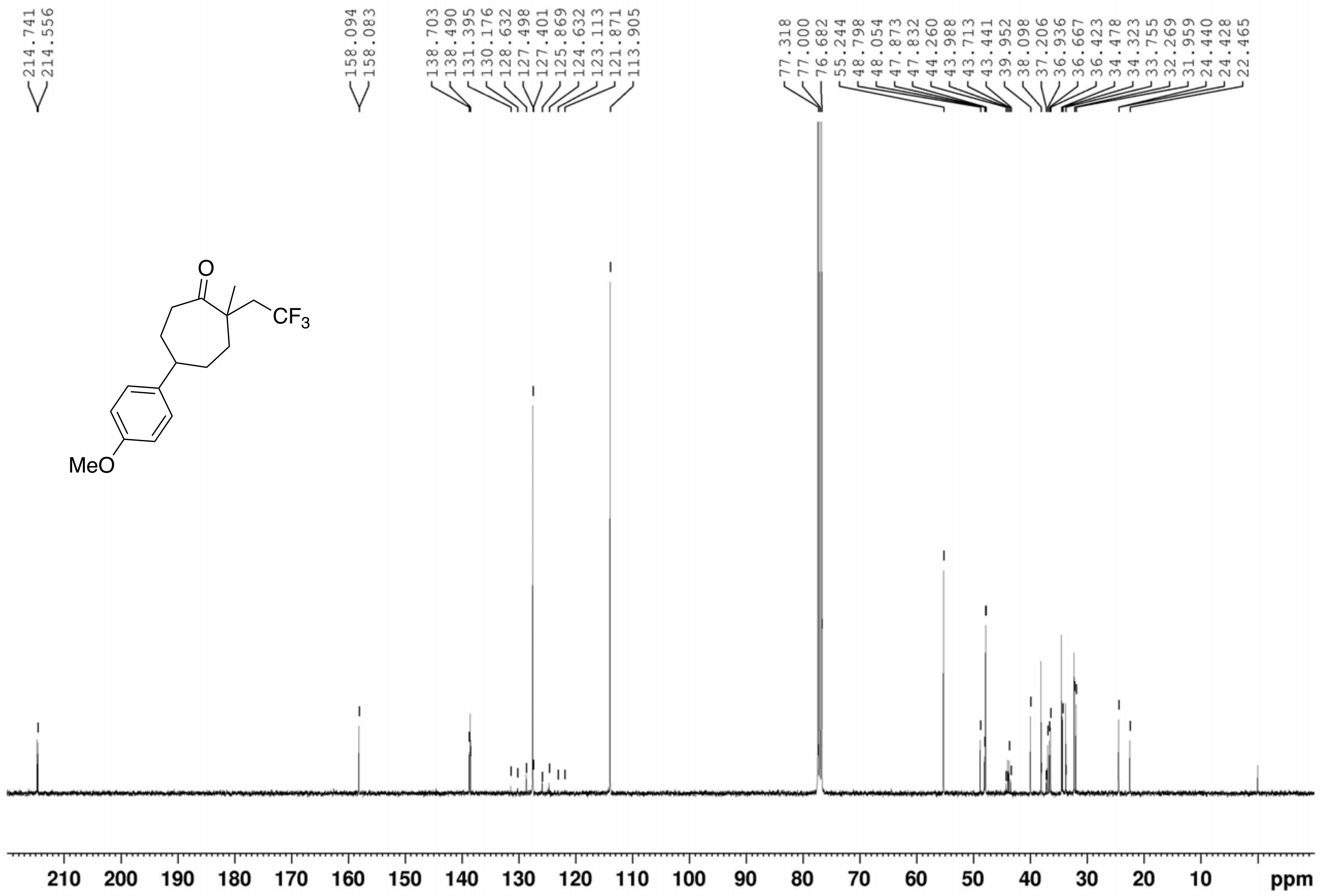

Supplementary Figure 168. ^{13}C NMR spectrum of **6cd** (100.6 MHz, $CDCl_3$)

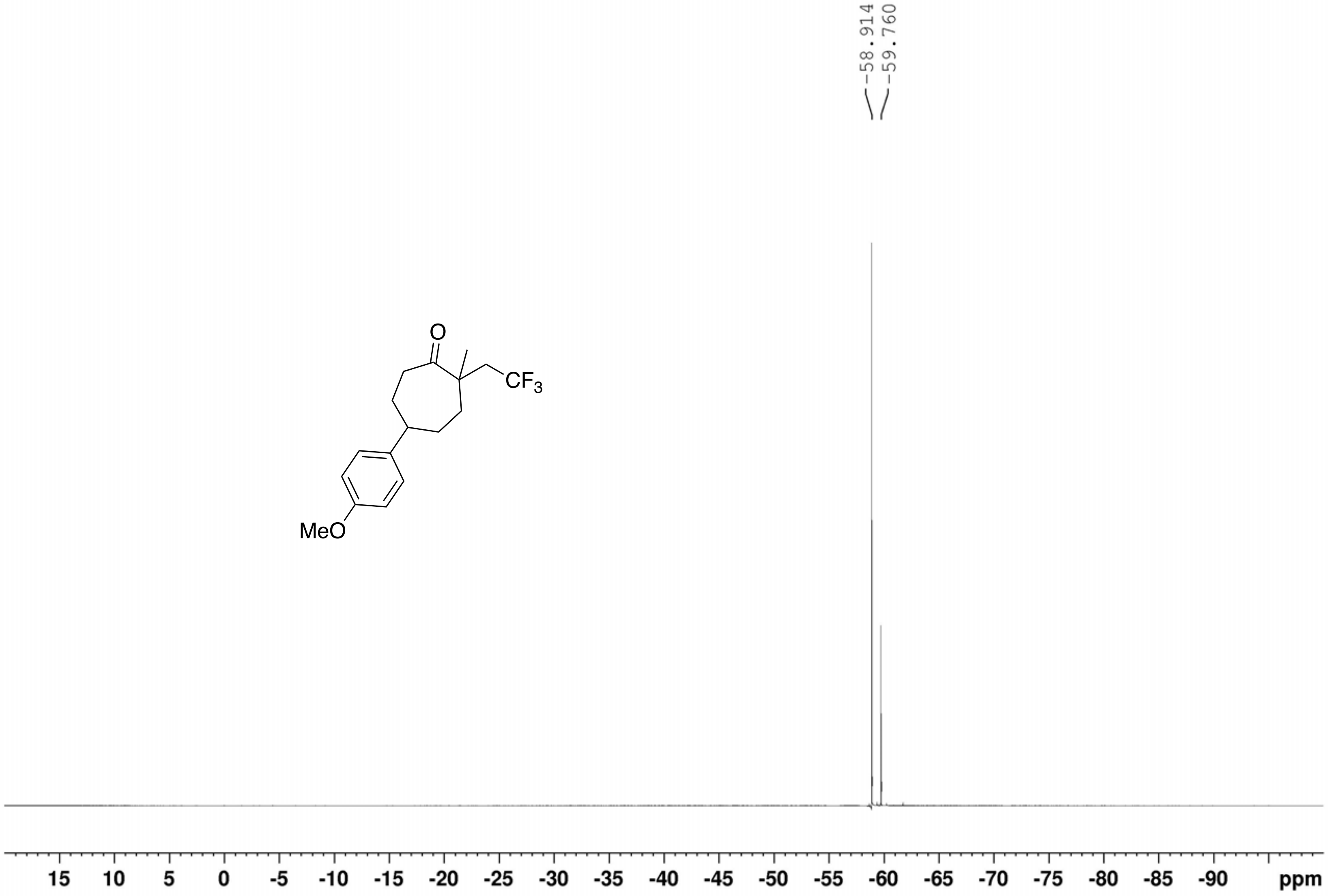

Supplementary Figure 169. ^{19}F NMR spectrum of **6cd** (376 MHz, $CDCl_3$)

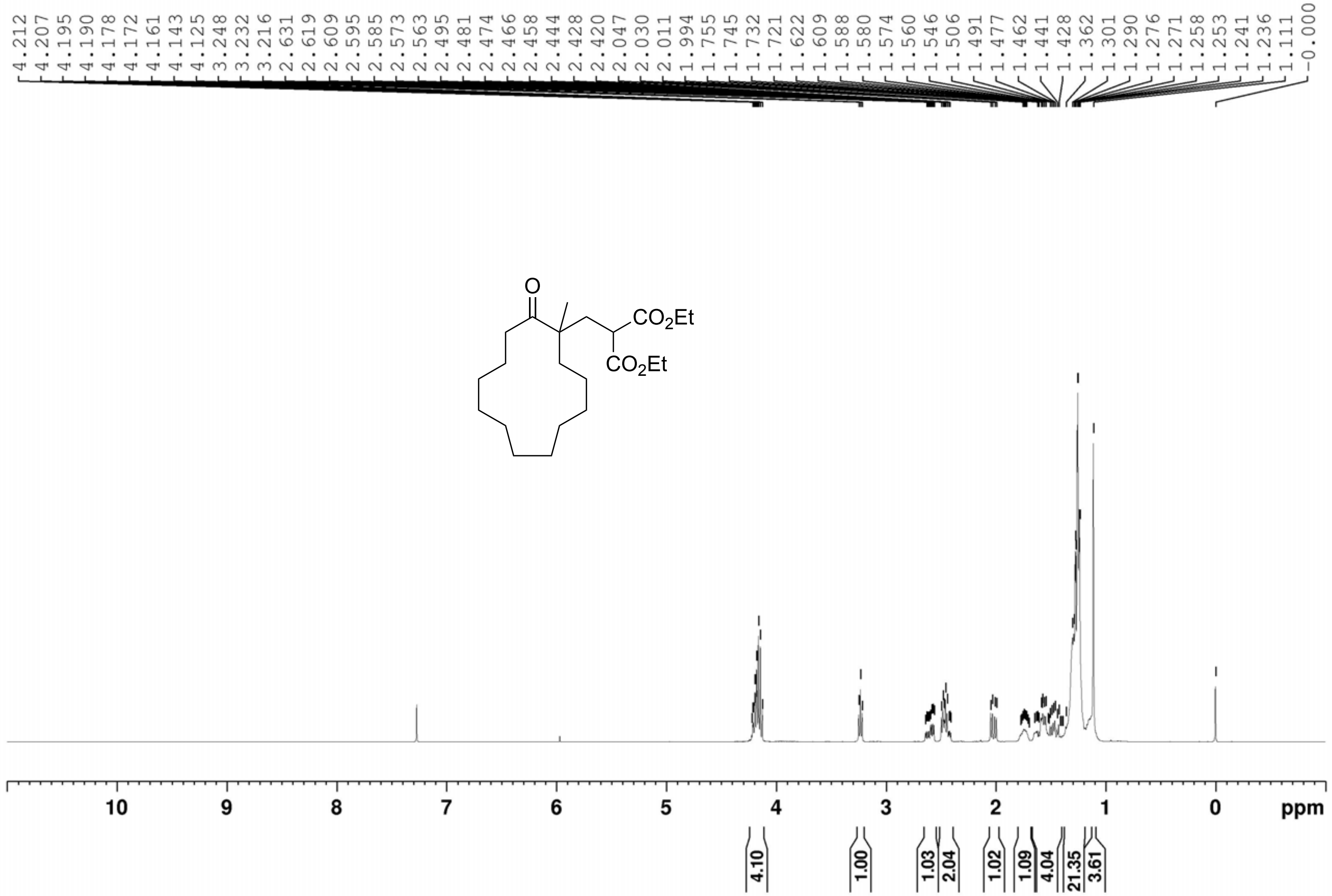

Supplementary Figure 170. ^{1}H NMR spectrum of **6da** (400 MHz, $CDCl_3$)

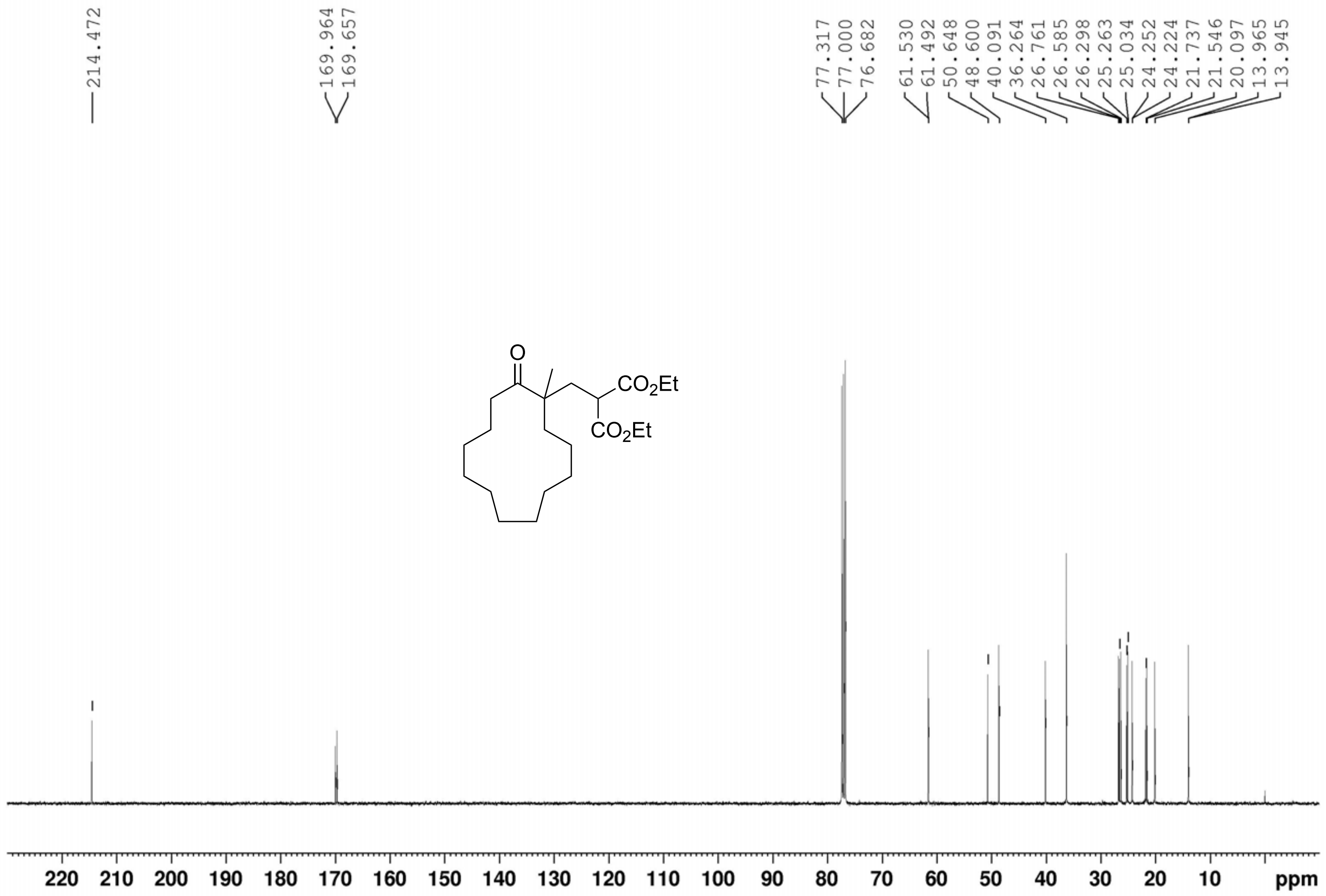

Supplementary Figure 171. ^{13}C NMR spectrum of **6da** (100.6 MHz, $CDCl_3$)

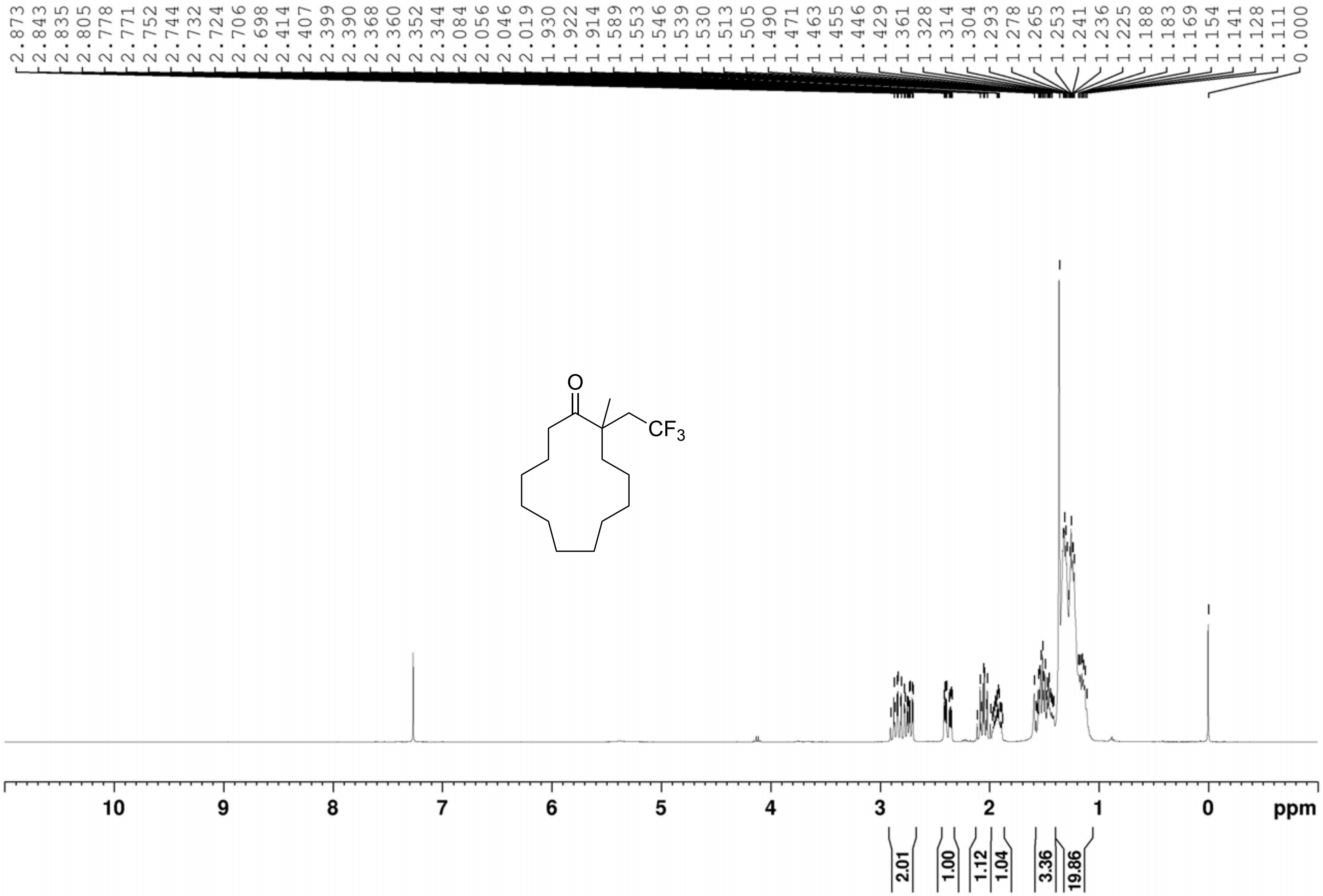

Supplementary Figure 172. ^{1}H NMR spectrum of **6dd** (400 MHz, $CDCl_3$)

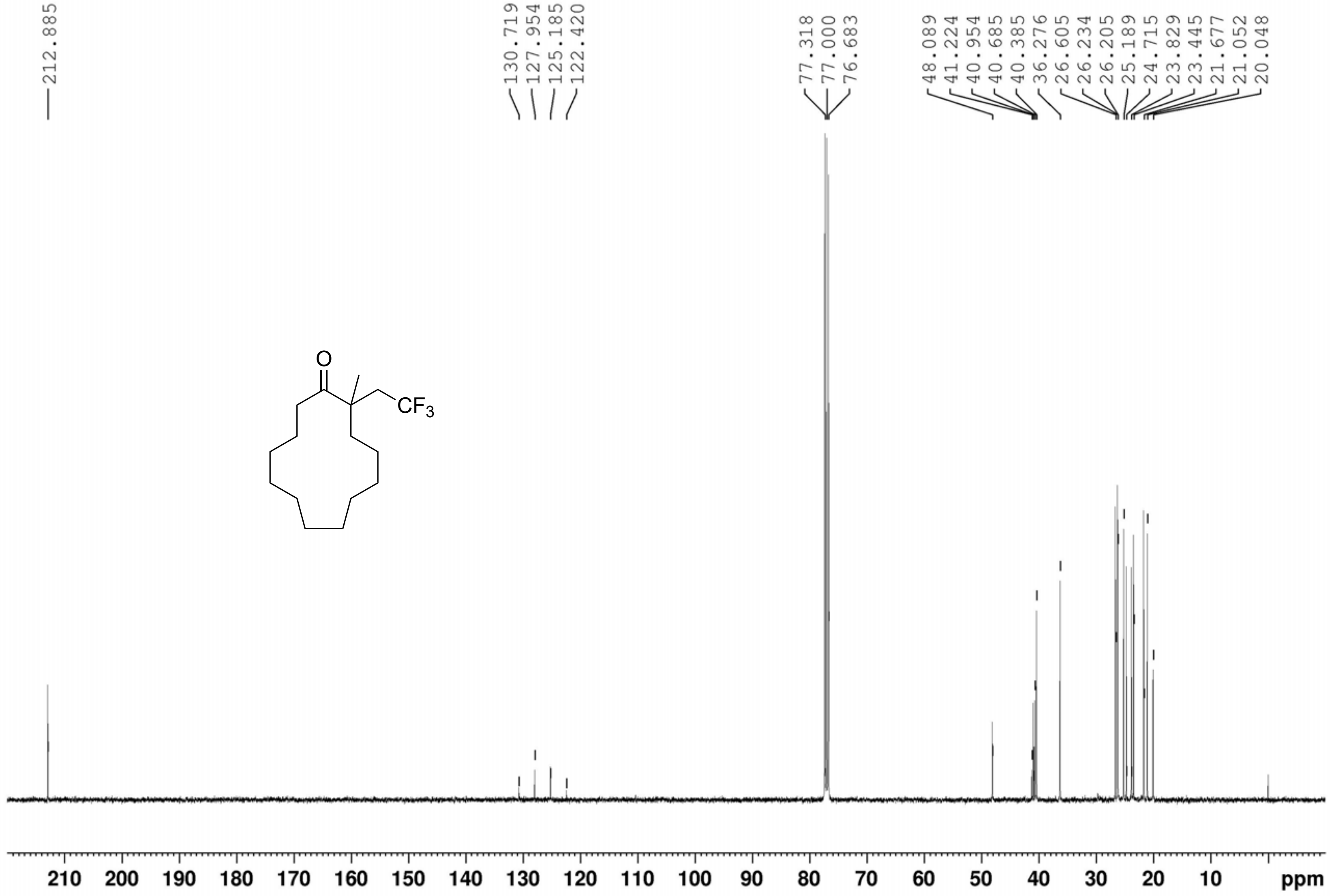

Supplementary Figure 173. ^{13}C NMR spectrum of **6dd** (100.6 MHz, $CDCl_3$)

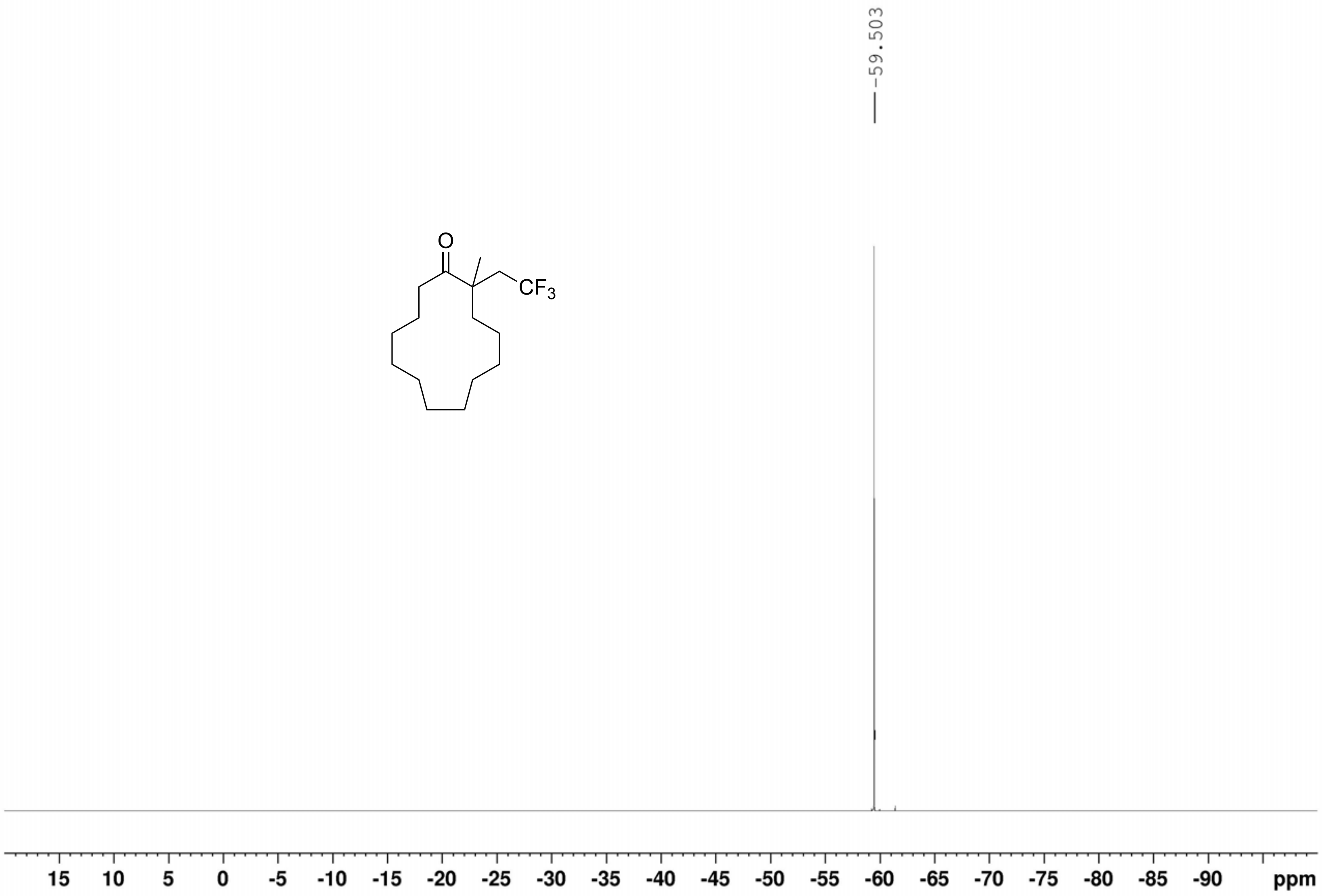

Supplementary Figure 174. ^{19}F NMR spectrum of **6dd** (376 MHz, $CDCl_3$)
